# Dynamically Generated Carbenium Species via Photoisomerization of Cyclic Alkenes: Mild Friedel–Crafts Alkylation

**DOI:** 10.1021/acs.joc.5c00061

**Published:** 2025-03-05

**Authors:** Timothy Schoch, Osaid Alkhamayseh, Nathan Herndon, Erik Lantz, Tyler Fleske, Jimmie D. Weaver

**Affiliations:** † Department of Chemistry, Oklahoma State University, Stillwater, Oklahoma 74078, United States

## Abstract

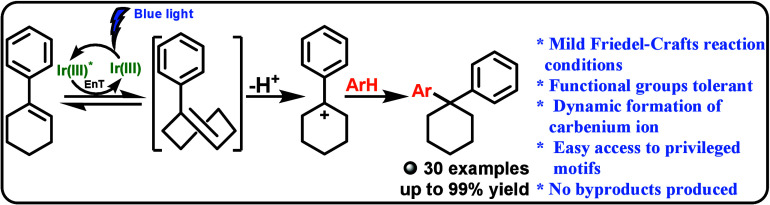

The torsional strain of *trans*-configured medium-sized (6–8) cycloalkenes imparts substantial potential energy efficiently toward ionic additions through generated reactive carbenium species. These reactions have been underexplored due to a historical necessity for harsh ultraviolet irradiation. We report here the Friedel–Crafts (FC) type reactivity of arylcycloalkenes (ACs) and π-nucleophiles for the first time with weak Brønsted acid and visible light energy transfer catalysis. Following optimizations using *p*-fluorophenyl cyclohexene as the AC and 2-methylfuran as the nucleophile, model conditions were obtained to probe the respective influence of the acid catalyst, aryl component of AC, nucleophile, and alicyclic component of AC on the desired FC reactivity. Each parameter was found to critically influence the course of the reaction. Ultimately, a mild, visible light-driven method for the preparation of a variety of 1,1-diarylcyclohexane and 4,4-diarylpiperidine derivatives that is mechanistically distinct from and complementary to other methods of preparation is outlined.

## Introduction

The release of molecular strain is a established technique for the efficient and selective provision of potential energy for organic reactions of both saturated^1−4^ and unsaturated systems.^5−7^ In these contexts, geometrically distorted bonds incorporated into the reactants can be considered alternatives to the forcing conditions necessary to access high-energy transition states of the desired transformations. A rich and nuanced subset of such transformations is exemplified by strained alkenes,^7−9^ which have carved out a niche in bioorthogonal “click” chemistry applications^10^ and synthesis.^11−15^ A core challenge to consider when designing such reactions, however, is how to incorporate the strained bonds into reactants in the first place. This still requires a large energy input, often from poorly discriminating reagents (such as Br_2_ for oxidation–elimination strategies),^5,11^ and may entail the irreversible formation of coproducts like CO_2_,^16^ N_2_,^17^ or Si–F species^18^ from preactivated starting materials.^19^ Moreover, strained alkenes are reactive by design, which has negative implications for their ability to be stored. An ideal solution would operate *in situ* with readily available and stable alkenes using the highly selective introduction of the strain in a dynamic fashion. Alkenes that can be efficiently and selectively strained when needed *in situ* address storage, safety, and functional group compatibility concerns. The dynamic transition between lower- and higher-potential energy states without arduous synthetic sequences is a hallmark of photochemistry.

**Figure 1:**
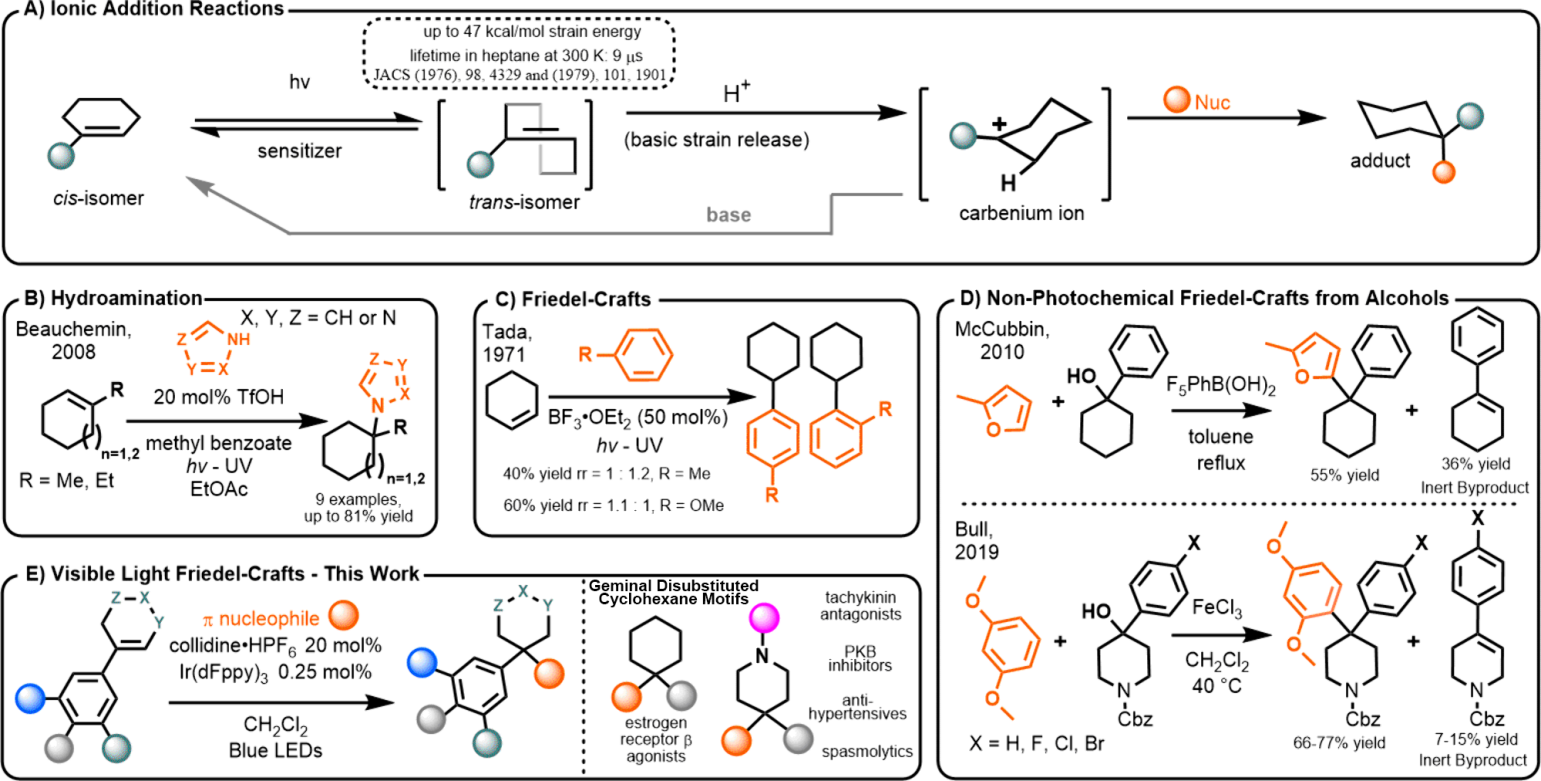
Cycloalkenes. Transiently strainable molecules for FC alkylation.

Our laboratory has taken inspiration from the seminal work of researchers like Paul Kropp^20,21^ and James Marshall,^22,23^ who pioneered the generation and utilization of small ring (6–8) *trans*-alkenes with ultraviolet (UV) irradiation. As much as 47 kcal/mol^24^ of potential energy as torsional strain has been measured for *trans*-ACs, which exist as shallow-welled ground state singlets. This strain has been used for pericyclic reactions^25−27^ and ionic addition reactions (Figure 1A).^21,28,29^ Critically, the ionic addition reactions require only weak Brønsted acids to proceed.^30^ The generated reactive carbenium species readily intercept water,^31^ alcohols,^28,32^ carboxylic acids,^33,34^ and carbamic acids.^35^ The Beauchemin laboratory demonstrated in 2008 that relatively mild hydroamination could be effected through the carbenium ions generated from simple *trans*-cycloalkenes and azolium triflates (Figure 1B).^36^ Moreover, albeit in limited fashion under extended UV irradiation with strong Lewis acid catalysis, aromatic nucleophiles have been demonstrated to undergo FC alkylation with *trans*-ACs (Figure 1C).^37^

1,1-Diaryl carbocycles constitute a known pharmacophore for estrogen receptor β agonism^38,39^ and proteasome inhibition,^40^ while related 4,4-disubstituted piperidines are a privileged medicinal structure pertaining to tachykinin antagonism,^41,42^ protein kinase b inhibition,^43^ and treatments for Parkinson’s disease^44^ and hypertension.^45^ These motifs are conventionally prepared from cyclohexanones or their corresponding alcohols under harshly acidic FC conditions.^46^ Somewhat gentler conditions were reported by McCubbin^47^ and Bull,^48^ but these were still limited to substrates with a favorable bifurcation of the carbenium reactivity between FC and inert elimination products, given the nondynamic nature of ion formation (Figure 1D). Considering the distinct mechanism for photochemical carbenium formation, which instead utilizes recursive, selective, and *in situ* alkene activation, we postulated that visible light energy transfer catalysis^49^ would be a complementary alternative to FC alkylations of alcohols (Figure 1E)

## Results and Discussion

**Scheme 1:**
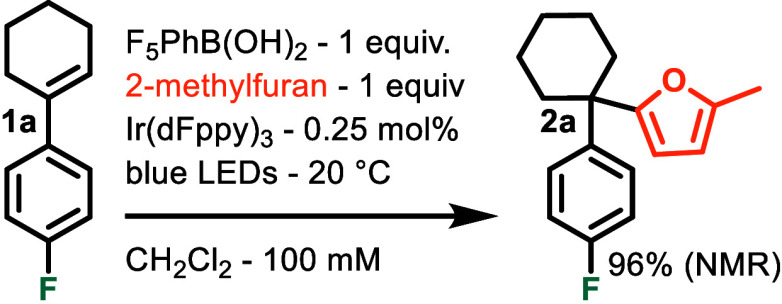
Proof of Concept

Prior work from our laboratory established cyclometalated iridium as an efficient class of energy transfer catalysts for alkene geometrical isomerization in CH_2_Cl_2_.^35,50^ Based on this and McCubbin’s work, we conducted a proof-of-concept reaction (Scheme 1) monitoring the conversion of 1-(4-fluorophenyl)cyclohexene **1a** to FC adduct **2a**. Although a qualified success, reaching up to 96% conversion as determined by ^19^F NMR, when scaled up for isolation, 1-(4-fluorophenyl)cyclohexanol was often observed as a photohydration byproduct, unmitigated by using an anhydrous solvent and glassware. Tentatively concerned that the pentafluorophenylboronic acid itself may be the source of water, we looked toward ionic Brønsted acid salts as alternatives. We hypothesized that cationic Brønsted acids with poorly coordinating counterions could be conducive to interception of carbenium ions by neutral π-nucleophiles. Carbenium ion formation would concomitantly produce an uncharged conjugate base rather than an anionic one and potentially render the carbenium more accessible. A broad screening of ammonium and pyridinium PF_6_^–^ salts [and a variety of other optimizations (see the Supporting Information)] revealed the use of 2,3,6-collidium PF_6_^–^ to be ideal.

Although there are many reports on the ionic reactivity mode for photon-generated *trans*-cycloalkenes,^28,32,35,36,51,52^ there remains a paucity involving the systematic probing of homologous derivatives. A lone 1987 report suggested that the nature of the aromatic ring has little influence on the *cis–trans*–AC isomerization barrier,^53^ though we expected it would have a significant impact on the *trans*–AC basicity. To demonstrate the role of the aromatic component, we examined a small series of ACs holding the nucleophile, 2-methylfuran, constant (Table 1**)**.

**Table 1:**
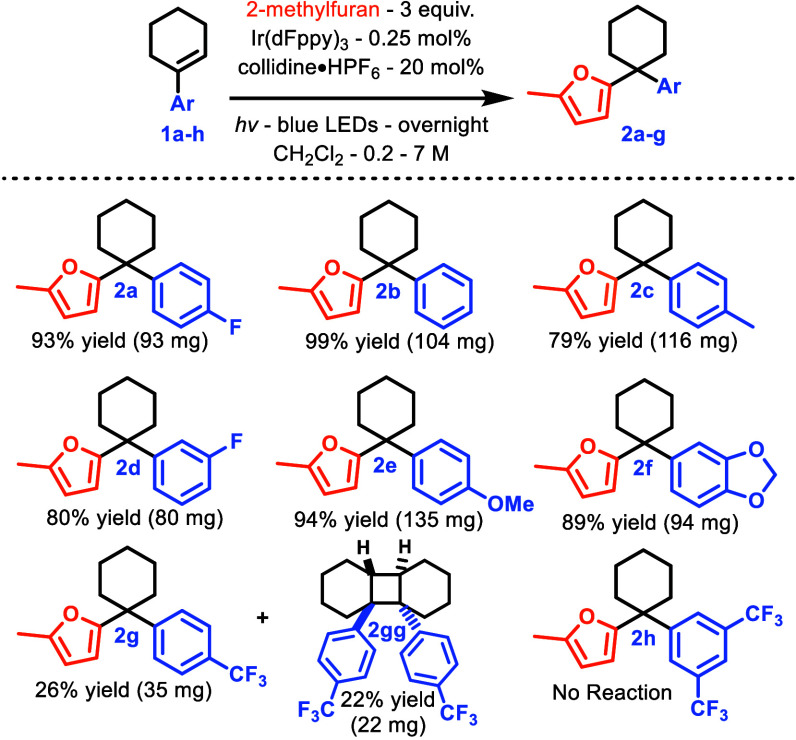
Variation of the Aromatic Part of Arylcyclohexenes

Electron-rich and neutral arenes **1a**–**1f** underwent efficient Friedel–Crafts alkylation, yielding products **2a**–**2f**, respectively. Surprisingly, 4-CF_3_-substituted arene **1g** underwent competitive [2+2] cyclodimerization, forming dimer **2gg** alongside the desired product **2g**. Although precedented,^35,54−56^ this form of cycloaddition was initially expected to be mitigated by the presence of a weak acid and a nucleophile. 3,5-Bis-CF_3_**1h** failed to yield FC adduct **2h** or pericyclic reactivity, apparently unable to support a reactive carbenium species. We next investigated the nucleophile scope.

Our initial studies of viable addition partners were shaped in part by the Mayr–Patz *N* parameters for π-nucleophiles according to the relationship described in refs :^57−59^.

Several less nucleophilic arenes failed to yield FC products with AC **1b** under our conditions [toluene, *m*-xylene, and 3-methylanisole (*N* = −4.36, −3.57, and 0.13, respectively)], while other more nucleophilic arenes succeeded in forming FC products (Table 2A, **3a**–**6b**). However, the clear electrophilicity of the carbenium species was complicated by the successful formation of FC product **4b** with less reactive arenes, such as thiophene, and by challenges in controlling monoalkylation versus dialkylation. In the case of pyrrole (*N* = 4.63), the reaction was easily controlled to yield monoalkylated products **5b** and **5b′**. In contrast, dimethoxybenzene (*N* = 2.48) led predominantly to dialkylated FC product **8b**, similar to thiophene. The heterocycles (**3a**–**7b**) followed established patterns of C1/C2 regioselectivity for electrophilic additions.^60^ Pyridines, obviously unsuitable as FC nucleophiles, were accessed through one additional step via a Ciamician–Dennstedt rearrangement^61^ of **5b**–**5bII**. Anilines, which are uncommon substrates for FC reactions, proved to be viable under our conditions. *N,N*-Dimethylaniline exhibited strict C4 selectivity, yielding FC product **9b**, while unsubstituted aniline favored N-alkylation, producing **10b′** as the major regioisomer and **10b** as the minor regioisomer. No C2-alkylated anilines were detected, in contrast with ordinary regioselectivity under stronger acid catalysis.^62−64^**11b**, a nucleophilic alkene entry, notably demonstrates the productive activation of **1b** in the presence of styrenes and the formation of a kinetic FC adduct regioisomer.

**Table 2:**
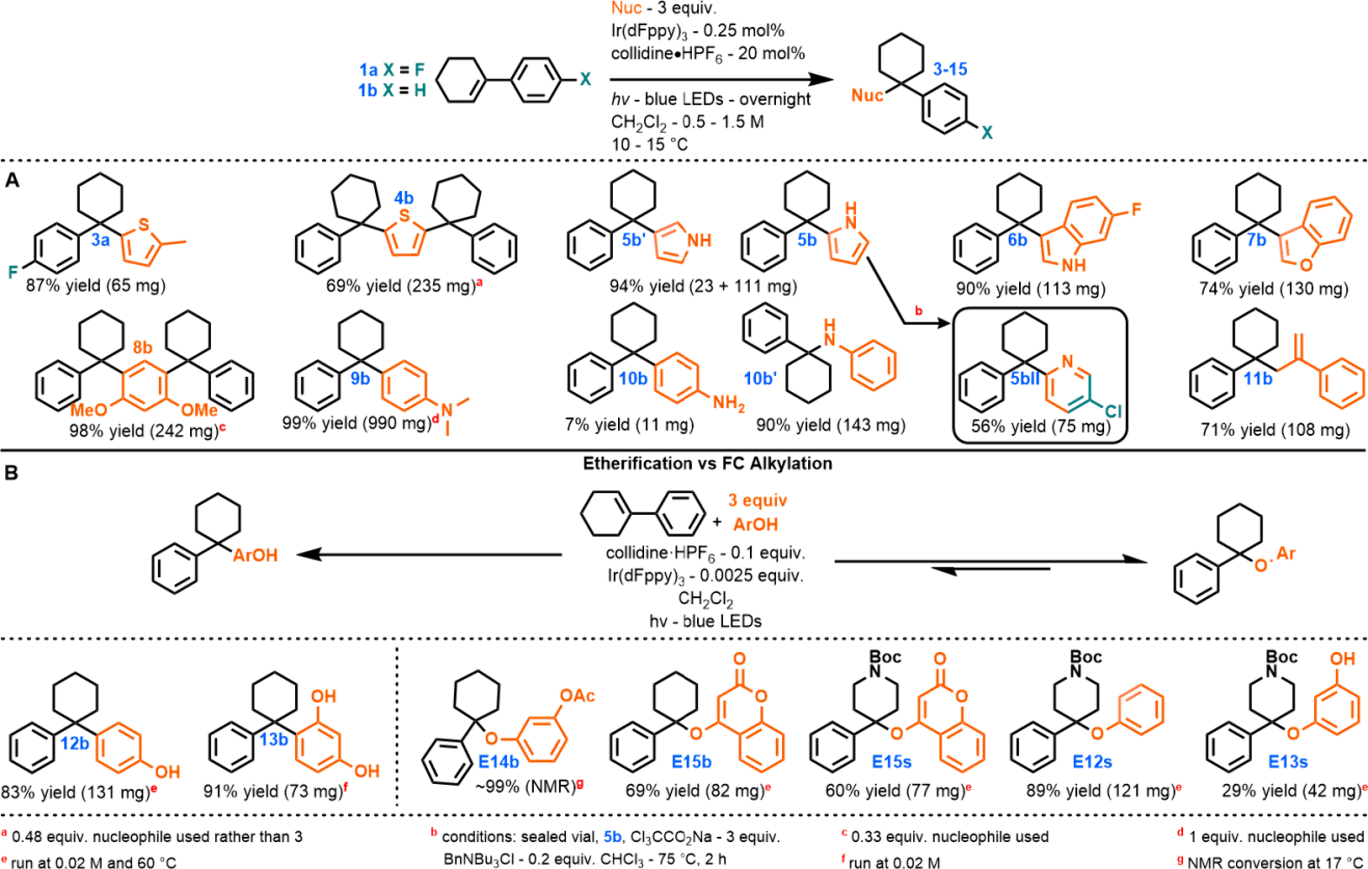
Variation of Nucleophiles for FC Alkylation

Consistent with our previous findings, some nucleophiles bearing hydroxy groups^28^ (Table 2B) tended to kinetically form a C–O bond but were ultimately able to convert to the favored C–C bond (**12b**–**13b**) while others were not (**E13s**–**E14b**). In the case of phenol and resorcinol, an equilibrium of etherification with **1b**, which could be accelerated by heating, was disrupted by irreversible FC reactivity, resulting in the formation of **12b** and **13b**, respectively. Resorcinol monoacetate was unable to interrupt the etherification to **E14b**, despite a demonstrated reversibility (Figure S5), while 4-hydroxycoumarin was found to irreversibly form ethers **E15b** and **E15s**. Phenol and resorcinol ethers **E12s** and **E13s**, respectively, of piperidine derivative **1s** were also unable to revert to **1s**, presumably due to the interaction of the carbamate with the proton.

The final aspect of variation within the project scope was the structure of the strainable ring. A series of derivatives were subjected to FC conditions to address this, as shown in Table 3. The formation of FC products **2i**–**2k** demonstrates the stereoselectivity of the reaction on the cyclohexane system, with only a single diastereomer, **2k**, observed for the *syn*-3,5-dimethylphenyl cyclohexene. The respective diastereomeric ratios, apparently independent of temperature, are consistent with a preferred equatorial approach of 2-methylfuran, affording the kinetic products over the thermodynamic ones. This is consistent with the stereoselectivity observed in organometallic methylations of related cyclohexanones^65^ and contrasts with the axial preference of metal hydride reductions in such systems.^66−69^ Selectivity for equatorial addition as seen here is usually observed for cases in which steric hindrance is a consideration,^70,71^ although it has also been demonstrated in the addition of HCl to **1j**.^72^

**Table 3:**
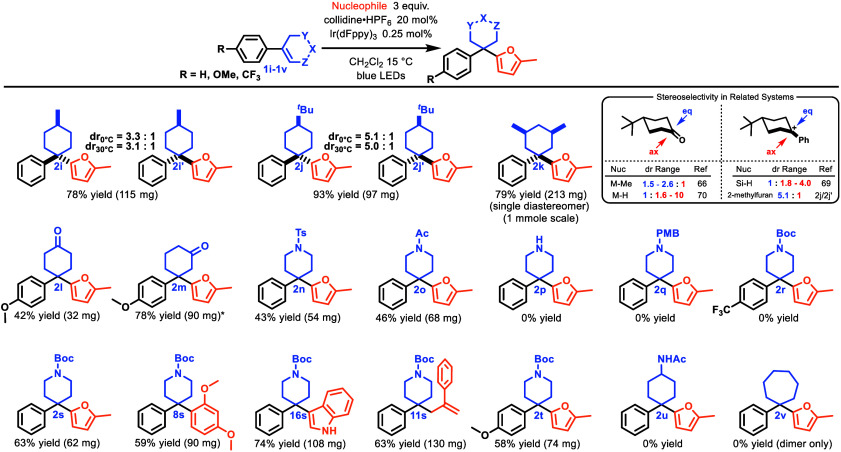
Variation of the Cyclohexene Ring

E. J. Corey^73^ and others have suggested that 2-cyclohexenones are unable to isomerize to their *trans* forms, due to the strain associated with their three contiguous sp^2^ centers. Under our FC conditions, this manifested as a simple lack of reactivity in the case of 3-arylcyclohex-2-en-1-ones. Interestingly, cyclohexene **1I** with three noncontiguous sp^2^ centers successfully formed FC product **2I**. A workaround for 3-arylcyclohex-2-en-1-ones involves using an ethylene ketal as a masking group, which yields **2m** after unmasking. This represents a mechanistically distinct, potentially complementary path to 3,3-diarylcyclohexanone motifs, previously reported by the Stanley group using Pd and arylboronic acids.^74^

Piperidyl alkenes were investigated as precursors to the medicinally relevant 4,4-diarylpiperidine structure. FC products **2n** and **2o**, protected with sulfonamide and acetamide groups, were obtained in moderate yields. However, piperidyl alkenes **1p** and **1q**, lacking electron-withdrawing N substituents, did not produce expected FC products **2p** and **2q**, respectively, instead leading to complex mixtures through oligomerization via N-alkylation. Additionally, piperidyl alkene **1r**, which contains the electron-withdrawing CF_3_ group, did not give FC product **2r**. The formation of products **2s** and **2t** demonstrated that Boc is the superior N-protecting group. Acetamide AC **1u** appeared to be photostable under the reaction conditions and did not yield FC product **2u**. This may be due to a form of intramolecular deactivation through the intermediacy of an unstable and reversible carboximidate. Seven-membered analogue **1v** exclusively formed the known [2+2] cyclodimer^54^ without any detectable FC adduct **2v**. It is not yet clear to us why pericyclic reactivity is so favored in our hands for **1v** over protonation, considering that ionic reactivity has been reported from it before, albeit under direct UV irradiation in acetic acid.^33^

## Mechanism

**Figure 2:**
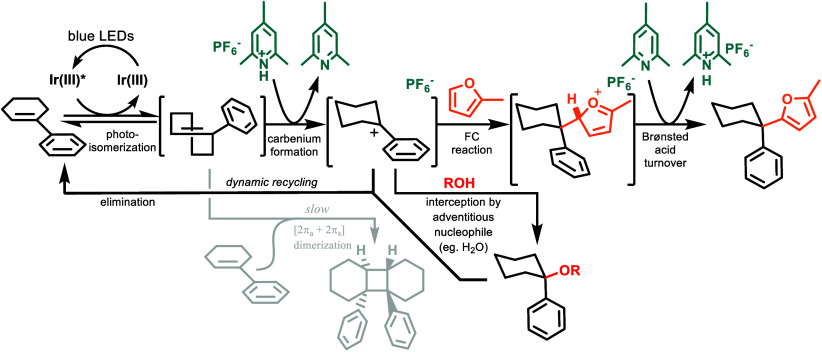
Proposed mechanism of the FC reaction.

Key aspects of our mechanistic understanding are listed in Figure 2. The reaction is initiated by absorption of a blue photon by the Ir photocatalyst, which gives rise to a long-lived triplet. This performs energy transfer with the *cis*-AC, giving rise to an electronically excited triplet biradical and returning the photocatalyst to the ground state. This biradical then relaxes, twisting toward a perpendicular geometry, eventually intersystem crossing, taking the substrate to the transition state. From this high-energy position, the path bifurcates between relaxation to the *cis* isomer or the twisted *trans* isomer. The *trans* isomer can undergo either spontaneous isomerization (back to the *cis* isomer), [2+2] cyclodimerization with the *cis* isomer, or protonation by the collidinium cation to generate a carbenium ion. The carbenium ion itself also partitions between several pathways depending on its environment. In the absence of a π-nucleophile, the carbenium can proceed with E1 elimination to regenerate *cis*-AC or react with an adventitious nucleophile. Water or alcohols, for example, may form alcohol and ether side products, respectively. Fortunately, these unproductive paths are both part of a dynamic process, allowing them to be recycled at the cost of more photons. After productive C–C bond formation, the loss of a proton rearomatizes the substrate to give the final product.

A common weakness associated with FC chemistry derived from addition to carbenium ions is the competition of undesired elimination reactions, leading to alkenes. This represents a decisive mechanistic path that erodes the yield for carbenium species not generated from alkene. The strategy described in Figure 2, conversely, dynamically regenerates the desired carbenium ions from the alkene, so rather than suffering yield loss to elimination, the loss is merely of energy efficiency.

**Scheme 2:**
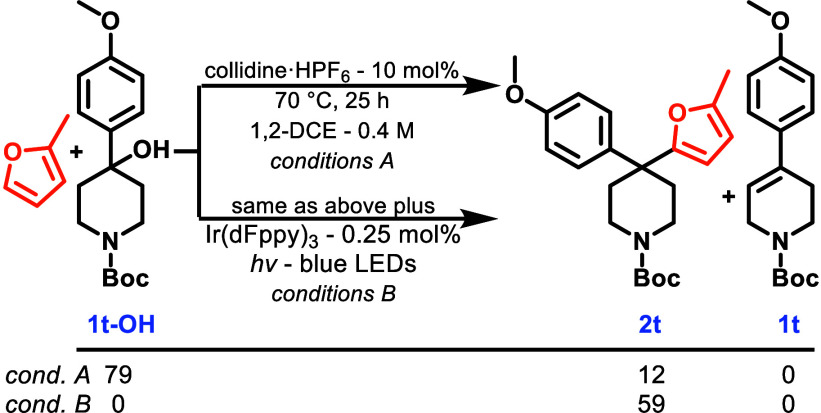
Exploiting the Dynamic Nature of Arylcyclohexenes

To illustrate this, we conducted two parallel reactions: one with a photocatalyst and blue light and one without, in which we started with the corresponding alcohol [**1t-OH** (Scheme 2)] instead of alkene. In both reactions, *in situ* ionization occurs via acid-catalyzed dehydration of **1t-OH**, and the resulting carbenium can then react with either water (to regenerate **1t-OH**) or 2-methylfuran. In the dark reaction (condition A), Freidel–Crafts product **2t** was produced in 12% yield while 79% of the starting material (**1t-OH**) remained. In contrast, when the photocatalyst was present (condition B), **2t** was formed in 59% yield and neither alkene nor starting material was detected in the reaction mixture. While it is clear that the greater yield of **2t** produced under condition B can be attributed to the photocatalyzed reaction, the exact cause is not clear at this time. Potentially, it may be attributed to the dynamic ability of the photocatalyst to re-engage cyclohexenes in productive bond formation, or alternatively, the formation of *trans*-cyclohexene may prevent decomposition of the collidinium hexafluorophosphate by water, which is needed to form the carbenium ion.

## Conclusion

In summary, a method has been developed for the preparation of geminal diarylcyclohexane derivatives. The mild nature of viable acid catalysts, compared to the Brønsted and Lewis catalysts/additives of most conventional FC reactions, is remarkable and provides substantial expansion of the scope of compatible functional groups, including carbamates, ketones, and dioxolanes. This was made possible by the unique ability of cyclohexenes to act as molecular transducers, converting the energy of selective electronic excitation to that of torsional strain, which can be exploited for the generation of carbenium ions under nonforcing conditions. We believe that much chemical space remains for transiently strainable cycloalkenes, holding promise for novel ways to orthogonally transduce chemical potential.

## Data Availability

The data underlying this study are available in the published article and its Supporting Information.

## Supporting Information Available

Optimizations, substrate scope investigations, and copies of ^1^H and ^13^C{^1^H} NMR spectra of all compounds (PDF)
FAIR data, including the primary NMR FID files, for compounds **2a–2g**, **2gg**, **3a**, **4b**, **5b**/**5b′**, **6b–9b**, **10b**/**10b′**, **11b–13b**, **2i**/**2i′**, **2j**/**2j′**, **2k–2o**, **2s**, **8s**, **16s**, **11s**, **2t**, **E12s**, **E13s**, **E15b**, and **E15s** (ZIP)
