# Supplementary material for: Dynamically Generated Carbenium Species via Photoisomerization of Cyclic Alkenes: Mild Friedel–Crafts Alkylation
Source: J Org Chem. 2025 Mar 5;90(10):3762–8. doi: 10.1021/acs.joc.5c00061 (PMC11915380; doi:10.1021/acs.joc.5c00061)

Supplementary Materials for

Dynamically Generated Carbenium Species via Photoisomerization of Cyclic Alkenes; Mild Friedel-Crafts Alkylation

Timothy Schoch [a,ǀ], Osaid Alkhamayseh [a,ǀ], Nathan Herndon [a], Erik Lantz [a], Tyler Fleske [a], Jimmie D. Weaver [a*]
[a] Department of Chemistry, Oklahoma State University, Stillwater, Oklahoma 74078, United States.
* Email: jimmie.weaver@okstate.edu.
ǀO.A. and ǀT.S. contributed equally as first-authors

The PDF file includes:

**General Experimental**	S2
**Scheme S1 – Preparation of 1-Arylcyclohexenes**	S3
**Scheme S2 – Preparation of Tetrahydropyridines**	S5
**Characteristic Datasheets for 1-Arylcyclohexenes**	S6-S10
**Scheme S3 – Preparation of PF_6^- Brønsted Acids**	S11
**Scheme S3.1 Preparation of Pyridinium salts with counter ions other than PF6-**	S12
**Scheme S4 – Representative Photo-FC Alkylation**	S13
**Figure S2 – Counterion Screening**	S14
**Characteristic Datasheets for FC Products**	S15-S26
**1 mmole scale synthesis of 2k**	S22
**Characteristic Datasheets for Ether Products**	S27-28
**Table S1 Screening of Initial Conditions**	S29
**Table S2 Bronsted Acid Cation Screening**	S30
**Table S3 The Effect of Acid Loading**	S31
**Table S4 The Effect of Temperature**	S32
**Figure S3 Gradual Degradation of Collidine•HPF_6 Under FC Conditions**	S33
**Figure S4 FC and Cyclodimerization of 1g**	S34
**Figure S5 Etherification of Resorcinol Monoacetate**	S35
**Figure S6 Assignment of Diastereomers by Selective Gradient NOESY**	S36
**Scheme S5 Ciamician-Dennstedt Rearrangement of 5b**	S37
**Scheme S6 Exploiting the Dynamic Nature of Arylcyclohexenes**	S38-40
**References**	S41
**NMR Spectra**	S42-S148

Materials and Methods

General Experimental: - All reagents were obtained from commercial suppliers (Aldrich, VWR, TCI chemicals, Oakwood chemicals, Alfa Aesar) and used without further purification unless otherwise noted. Reactions were monitored by thin layer chromatography (TLC), obtained from sorbent technology Silica XHL TLC Plates, w/UV254, glass backed, 250 µm, 20 x 20 cm visualized with ultraviolet light, by assay GCMS using Shimadzu QP2020 NX, and/or by ^{1}H/^{19}F NMR. Solvents were used as received. Flash chromatography was carried out with Merck 60 Å, mesh 230-400 silica gel on a Teledyne ISCO CombiFlash NEXTGEN 300. All compounds that were purified by flash chromatography utilized a gradient of hexanes and ethyl acetate (EtOAc) unless otherwise noted. NMR spectra were obtained on 400 MHz Bruker Avance III spectrometer, or a Bruker NEO800 MHz spectrometer equipped with cryoprobe. 1H, 19F and 13C NMR chemical shifts are reported in ppm relative to the residual proteo solvent peak. High resolution mass spectra (HRMS) analysis was performed on LTQ-OrbitrapXL by Thermo Scientific ltd.
Photochemistry was carried out in one of three reactors depending on the temperature:

A. a columnar reactor bath equipped with a linear vertical array of 1 W 447 nm LEDs and a recirculating chiller maintaining 20 °C or less,
B. a reactor fitted with Blue Waterproof 5050 36W LED Strip Lights (Solid Apollo) spiral-wrapped around the inside of a metal canister fitted with a cooling fan at the base, maintaining between 20 and 30 °C,
C. a reactor composed of a recrystallization dish spiral-wrapped with the same lights on the exterior, secured with masking tape, and wrapped in aluminum foil. Water in the dish was maintained at given temperatures above 30 °C by a heated and thermostatted magnetic stir plate.

Figure S1 - Blue LED Photoreactors

A <= 20 °C   20 °C < **B** < 30 °C   **C** >= 30 °C

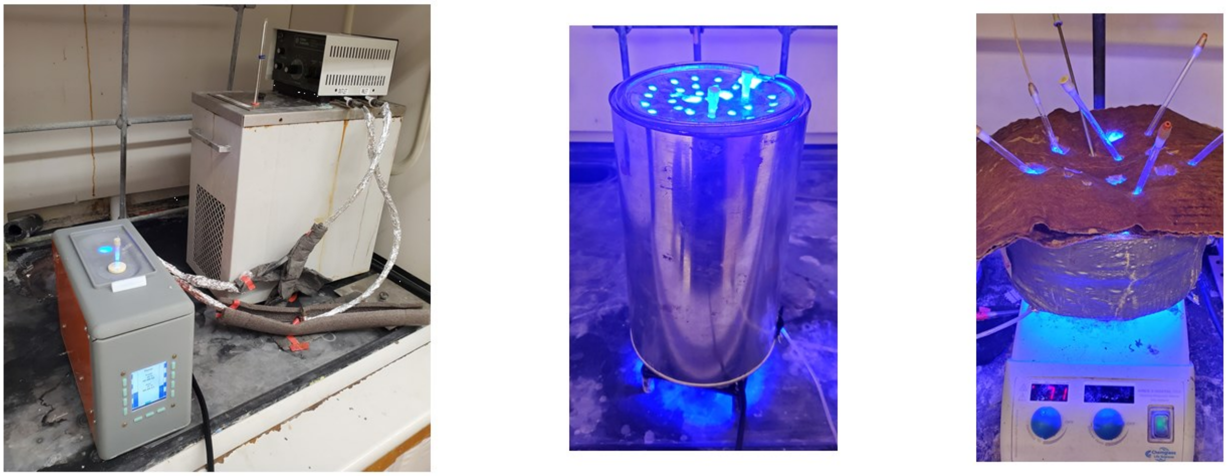

Scheme S1 Preparation of 1-Arylcyclohexenes

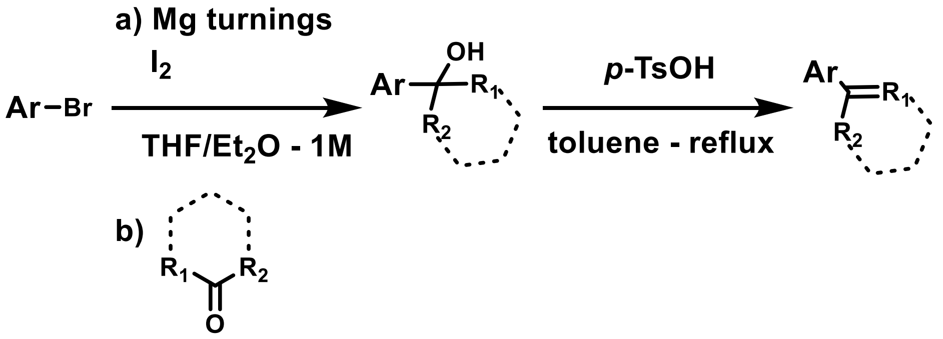

The general procedure for the synthesis of 1-arylcyclohexenes was as follows: a round-bottom flask with 2.5 equivalents of Mg turnings and a magnetic stir bar was flame-dried and placed under an atmosphere of argon along with 1 mol% of I_2. To this was added a 1 M solution of aryl bromide (1.2 equivalents relative to cyclohexanone derivative) in THF or Et_2O with vigorous stirring until an hour elapsed past the disappearance of brown I_2 color. The aryl magnesium bromide solution was then cooled to 0 °C and the corresponding cyclohexanone was added (as the limiting reagent). Once all the cyclohexanone compound was added, the reaction mixture was allowed to warm to room temperature with stirring before quenching with 1 M HCl solution when deemed complete by TLC (usually overnight). The reaction mixture was extracted with Et_2O twice. The organic layers were combined, washed with brine and water then dried over $MgSO_4$. The tertiary alcohol product mixture was usually purified by crystallization from hexanes prior to treatment to 10 mol% *p*-TsOH in refluxing toluene. Once the elimination reaction was complete, the reaction was

quenched with a 1 M NaOH solution. The quenched mixture was then transferred to a separatory funnel and extracted twice with DCM. The organic layers were combined, washed with brine, and dried over $MgSO_4$. After the workup, the desired arylcyclohexene was purified by silica gel column chromatography using 100% hexane as the eluent.

Scheme S2 Preparation of Tetrahydropyridines

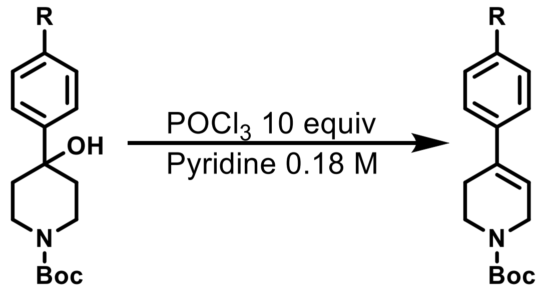

R = H, MeO, CF_3

R	Alcohol starting material (mass in gram, mmole)	Alkene product % yield (mass in gram)
H	(2 g, 7.22 mmole)	59% (1.1 g)
MeOH	(5.5 g, 17.9 mmole)	72% (3.7 g)
CF_3	(5.5 g, 16.1 mmole)	93% (4.89 g)

Rather than exposing the 4-azacycloalkanols to *p*-TsOH●H_2O as in the previous procedure, they were treated to alternative elimination conditions to preserve their carbamate functionality. Phosphorus oxychloride was added slowly to a solution of the corresponding alcohol in pyridine at room temperature. This mixture was stirred overnight, then quenched with aqueous $NaHCO_3$. The aqueous mixture was extracted with CH_2Cl_2 which was then washed with brine and dried over $MgSO_4$ before concentrating *in vacuo*. All products were obtained as yellow solids without further purification.

Characteristic Datasheets for Arylcyclohexenes

[1a] 4'-fluoro-2,3,4,5-tetrahydro-1,1'-biphenyl

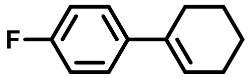

The alkene was prepared in accordance with Scheme S1. Characteristic data were found consistent with Nakamura's 2010 report.[1]

^{1}H NMR (800 MHz, $CDCl_3$) δ 7.35 – 7.30 (m, 2H), 7.01 – 6.96 (m, 2H), 6.05 (tt, *J* = 3.9, 1.7 Hz, 1H), 2.40 – 2.35 (m, 2H), 2.22 – 2.17 (m, 2H), 1.80 – 1.75 (m, 2H), 1.68 – 1.63 (m, 2H).

^{13}C{1H} NMR (201 MHz, $CDCl_3$) δ 162.5, 138.9 (d, *J* = 3.5 Hz), 135.8, 126.5 (d, *J* = 7.7 Hz), 124.8, 115.0 (d, *J* = 21.4 Hz), 27.7, 26.0, 23.2, 22.2.

[1b] 2,3,4,5-tetrahydro-1,1'-biphenyl

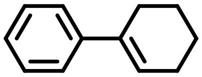

The alkene was prepared in accordance with Scheme S1. **1b** is commercially available.

^{1}H NMR (800 MHz, $CDCl_3$) δ 7.39 (dq, *J* = 8.3, 1.4 Hz, 2H), 7.31 (tt, *J* = 8.2, 1.5 Hz, 2H), 7.22 (tt, *J* = 7.3, 1.4 Hz, 1H), 6.13 (tt, *J* = 4.0, 1.9 Hz, 1H), 2.42 (tdd, *J* = 7.0, 2.6, 1.2 Hz, 2H), 2.22 (dtq, *J* = 8.0, 3.9, 2.2 Hz, 2H), 1.82 – 1.76 (m, 2H), 1.68 (dddt, *J* = 10.7, 6.1, 4.8, 2.2 Hz, 2H).

^{13}C{1H} NMR (201 MHz, $CDCl_3$) δ 142.8, 136.7, 128.3, 126.6, 125.1, 124.9, 27.5, 26.0, 23.2, 22.3.

[1c] 4'-methyl-2,3,4,5-tetrahydro-1,1'-biphenyl

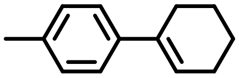

The alkene (a clear oil) was prepared in accordance with Scheme S1. Characteristic data were found consistent with Nakamura's 2010 report.[1]

^{1}H NMR (800 MHz, $CDCl_3$) δ 7.30 – 7.27 (m, 2H), 7.14 – 7.11 (m, 2H), 6.09 (tt, *J* = 3.9, 1.7 Hz, 1H), 2.42 – 2.39 (m, 2H), 2.34 (s, 3H), 2.23 – 2.18 (m, 2H), 1.82 – 1.76 (m, 2H), 1.69 – 1.64 (m, 2H).

^{13}C{1H} NMR (201 MHz, $CDCl_3$) δ 140.0, 136.5, 136.2, 129.0, 124.9, 124.1, 27.6, 26.0, 23.2, 22.3, 21.2.

[1d] 3'-fluoro-2,3,4,5-tetrahydro-1,1'-biphenyl

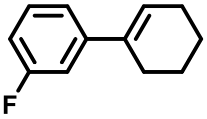

The alkene was prepared in accordance with Scheme S1. Data were consistent with Ruben Martin's 2011 report.[2]

[1e] 4'-methoxy-2,3,4,5-tetrahydro-1,1'-biphenyl

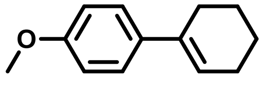

The compound was isolated as a colorless crystalline solid following Scheme S1. Characteristic data were consistent with Nakamura's 2010 report.[1]

^{1}H NMR (800 MHz, $CDCl_3$) δ 7.34 – 7.31 (m, 2H), 6.88 – 6.84 (m, 2H), 6.04 (tt, *J* = 3.9, 1.7 Hz, 1H), 3.81 (s, 3H), 2.41 – 2.38 (m, 2H), 2.23 – 2.18 (m, 2H), 1.81 – 1.76 (m, 2H), 1.69 – 1.64 (m, 2H).

^{13}C{1H} NMR (201 MHz, $CDCl_3$) δ 158.5, 136.0, 135.5, 126.0, 123.3, 113.7, 55.4, 27.6, 26.0, 23.3, 22.4.

[1f] 5-(cyclohex-1-en-1-yl)benzo[d][1,3]dioxole

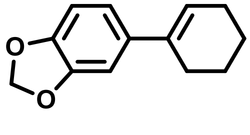

The compound was isolated in 97% yield (1.78 g) as a colorless oil following Scheme S1. Characteristic data were found consistent with Knowle's 2013 report.[3]

^{1}H NMR (800 MHz, $CDCl_3$) δ 6.90 (d, *J* = 1.9 Hz, 1H), 6.85 (dd, *J* = 8.1, 1.8 Hz, 1H), 6.76 (d, *J* = 8.1 Hz, 1H), 6.01 (tt, *J* = 3.9, 1.7 Hz, 1H), 5.93 (s, 2H), 2.37 – 2.33 (m, 2H), 2.21 – 2.16 (m, 2H), 1.79 – 1.74 (m, 2H), 1.67 – 1.62 (m, 2H).

^{13}C{1H} NMR (201 MHz, $CDCl_3$) δ 147.7, 146.3, 137.4, 136.2, 123.9, 118.3, 108.0, 105.8, 101.0, 27.8, 26.0, 23.2, 22.3.

GC/MS – EI (m/z, relative intensity) 202 (M^+, 100), 187 (12), 174 (28), 144 (45), 116 (50), 115 (40)

[1g] 4'-(trifluoromethyl)-2,3,4,5-tetrahydro-1,1'-biphenyl

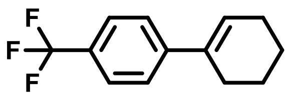

The compound was obtained in 92% yield (1.05 g) as a colorless oil following Scheme S1. Characteristic data were consistent with Knowles 2013 report.[3]

^{1}H NMR (800 MHz, CDCl$_3$) δ 7.55 (d, *J* = 8.2 Hz, 1H), 7.46 (d, *J* = 8.2 Hz, 1H), 6.21 (tt, *J* = 3.9, 1.8 Hz, 1H), 2.43 – 2.38 (m, 1H), 2.26 – 2.21 (m, 1H), 1.83 – 1.77 (m, 1H), 1.71 – 1.65 (m, 1H).

^{13}C{1H} NMR (201 MHz, CDCl$_3$) δ 146.1, 135.7, 128.4 (q, *J* = 32.2 Hz), 127.1, 125.1, 125.1 (q, *J* = 4.1 Hz), 124.4 (q, *J* = 271.9 Hz), 27.3, 25.9, 22.9, 22.0.

^{19}F NMR (753 MHz, CDCl$_3$) δ -62.3.

[1h] 3',5'-bis(trifluoromethyl)-2,3,4,5-tetrahydro-1,1'-biphenyl

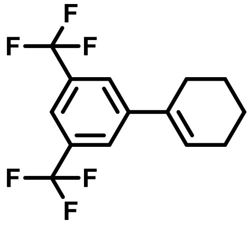

The compound was obtained in 93% yield (880 mg) as a colorless oil following Scheme S1. Characteristic data was consistent with Hartwig's 2014 report.[4]

^{1}H NMR (800 MHz, CDCl$_3$) δ 7.78 (d, *J* = 1.7 Hz, 2H), 7.71 (s, 1H), 6.27 (tt, *J* = 3.9, 1.7 Hz, 1H), 2.45 – 2.40 (m, 2H), 2.28 – 2.23 (m, 2H), 1.85 – 1.79 (m, 2H), 1.72 – 1.66 (m, 2H).

^{13}C{1H} NMR (201 MHz, CDCl$_3$) δ 144.8, 134.7, 131.5 (q, *J* = 32.8 Hz), 128.6, 125.1 (q, *J* = 3.3 Hz), 123.7 (q, *J* = 272.8 Hz), 120.2 (q, *J* = 3.9 Hz), 27.3, 26.0, 22.8, 21.9.

^{19}F NMR (753 MHz, CDCl$_3$) δ -62.9.

[1i] 4-methyl-2,3,4,5-tetrahydro-1,1'-biphenyl

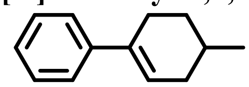

The compound was isolated in 95% yield (1.9 g) as a beige transparent liquid following Scheme S1. The characteristic data are consistent with Yamamoto's 1994 report.[5]

^{1}H NMR (800 MHz, CDCl$_3$) δ 7.43 – 7.38 (m, 2H), 7.34 – 7.29 (m, 2H), 7.25 – 7.20 (m, 1H), 6.11 (ddt, *J* = 4.8, 3.1, 1.8 Hz, 1H), 2.48 (dddq, *J* = 9.0, 5.2, 3.7, 1.9 Hz, 2H), 2.32 (dddd, *J* = 17.8, 6.9, 3.4, 1.7 Hz, 1H), 1.88 (dtdd, *J* = 11.8, 4.4, 3.0, 1.6 Hz, 1H), 1.84 (dtd, *J* = 18.0, 6.3, 3.1 Hz, 1H), 1.80 – 1.71 (m, 1H), 1.40 (dddd, *J* = 12.8, 11.0, 8.8, 7.2 Hz, 1H), 1.03 (d, *J* = 6.6 Hz, 3H).

^{13}C{1H} NMR (201 MHz, CDCl$_3$) δ 142.5, 136.3, 128.3, 126.6, 125.1, 124.4, 34.6, 31.4, 28.3, 27.6, 21.9.

[1j] 4-(*tert*–butyl)-2,3,4,5-tetrahydro-1,1'-biphenyl

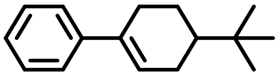

The compound was isolated according to Scheme S1 as a colorless oil (894 mg, 96% yield). Spectral characterization was consistent with Ackermann's 2011 report.[6]

^{1}H NMR (800 MHz, CDCl$_3$) δ 7.39 (dq, *J* = 8.1, 1.5 Hz, 2H), 7.31 (ddd, *J* = 9.2, 5.0, 2.0 Hz, 2H), 7.21 (tdd, *J* = 7.5, 2.7, 1.3 Hz, 1H), 6.14 (dt, *J* = 5.5, 2.7 Hz, 1H), 2.53 (ddt, *J* = 16.7, 4.7, 2.5 Hz, 1H), 2.44 (ttt, *J* = 13.9, 4.5, 2.1 Hz, 1H), 2.26 (dtt, *J* = 15.3, 4.5, 2.3 Hz, 1H), 2.02 – 1.94 (m, 2H), 1.37 (tdd, *J* = 11.2, 3.8, 2.0 Hz, 1H), 1.35 – 1.29 (m, 1H), 0.92 (dd, *J* = 3.2, 1.2 Hz, 9H).

^{13}C{1H} NMR (201 MHz, CDCl$_3$) δ 142.4, 136.5, 128.3, 126.6, 125.1, 125.1, 43.9, 32.4, 29.0, 27.6, 27.4, 24.6.

GC/MS (m/z, relative intensity) 214 (M^+, 50), 158 (50), 143 (65), 130 (100), 115 (60), 104 (25), 91 (95)

[1k] *cis*–3,5-dimethyl-2,3,4,5-tetrahydro-1,1'-biphenyl

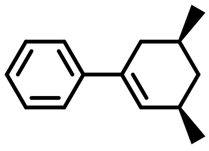

The compound was prepared from the corresponding alcohol (2 g, 9.8 mmol) according to Scheme S1, obtained as a colorless oil (1.82 g, 99% yield). This compound has been previously prepared using an alternative method by Mikhailov and Cherkasova.[7]

^{1}H NMR (800 MHz, CDCl$_3$) δ 7.39 (dq, *J* = 8.1, 1.5 Hz, 2H), 7.31 (ddd, *J* = 9.2, 5.0, 2.0 Hz, 2H), 7.21 (tdd, *J* = 7.5, 2.7, 1.3 Hz, 1H), 6.14 (dt, *J* = 5.5, 2.7 Hz, 1H), 2.53 (ddt, *J* = 16.7, 4.7, 2.5 Hz, 1H), 2.44 (ttt, *J* = 13.9, 4.5, 2.1 Hz, 1H), 2.26 (dtt, *J* = 15.3, 4.5, 2.3 Hz, 1H), 2.02 – 1.94 (m, 2H), 1.37 (tdd, *J* = 11.2, 3.8, 2.0 Hz, 1H), 1.35 – 1.29 (m, 1H), 0.92 (dd, *J* = 3.2, 1.2 Hz, 9H).

^{13}C{1H} NMR (201 MHz, CDCl$_3$) δ 142.4, 136.5, 128.3, 126.6, 125.1, 125.1, 43.9, 32.4, 29.0, 27.6, 27.4, 24.6.

GC/MS (m/z, relative intensity) 214 (M^+, 50), 158 (50), 143 (65), 130 (100), 115 (60), 104 (25), 91 (95)

[1l] 4'-methoxy-2,5-dihydro-[1,1'-biphenyl]-4(3H)-one

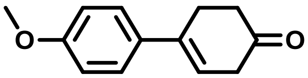

The compound was prepared previously by Jon Day from cyclohexanedione monoethylene ketal.[8]

mp: 68-70 °C

^{1}H NMR (400 MHz, $CDCl_3$) δ 7.37 – 7.30 (m, 2H), 6.93 – 6.85 (m, 2H), 6.00 (tt, *J* = 3.9, 1.3 Hz, 1H), 3.82 (s, 3H), 3.05 (dt, *J* = 3.9, 1.9 Hz, 2H), 2.87 (tq, *J* = 6.7, 1.7 Hz, 2H), 2.64 (t, *J* = 6.9 Hz, 2H).

^{13}C{1H} NMR (101 MHz, $CDCl_3$) δ 210.3, 159.0, 137.1, 133.3, 126.3, 119.3, 113.8, 55.3, 39.9, 38.7, 28.0.

[1m] 7-(4-methoxyphenyl)-1,4-dioxaspiro[4.5]dec-6-ene

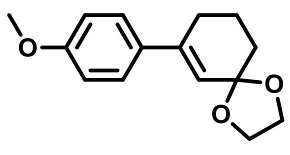

1m was obtained by treating the known compound 3-(4-methoxyphenyl)cyclohex-2-enone (1 g, 4.94 mmol)[9] to ethylene glycol (276 microliters, 1 equiv.) and *p*-toluenesulfonic acid monohydrate (100 mg, 0.1 equiv.) in refluxing toluene (100 mL) for 6 hours. The reaction, when deemed complete by TLC, was cooled, quenched with aqueous $NaHCO_3$, diluted with EtOAc (50 mL), and washed consecutively with water and saturated brine solution. The organic layer was dried over $MgSO_4$ and purified by flash column chromatography. The product was obtained as a white powder (96 mg, 8% yield).

mp: 101-102 °C

^{1}H NMR (800 MHz, $CDCl_3$) δ 7.32 – 7.28 (m, 2H), 6.86 – 6.82 (m, 2H), 6.04 (tt, *J* = 3.9, 1.8 Hz, 1H), 4.06 – 3.99 (m, 4H), 3.80 (s, 3H), 2.63 (q, *J* = 2.1 Hz, 2H), 2.42 (tdt, *J* = 6.4, 4.3, 2.3 Hz, 2H), 1.81 (tt, *J* = 6.5, 0.8 Hz, 2H).

^{13}C{1H} NMR (201 MHz, $CDCl_3$) δ 158.8, 134.1, 134.0, 126.2, 121.8, 113.7, 108.7, 64.6, 55.4, 38.0, 30.7, 24.8.

GC/MS (m/z, relative intensity) 246 (M^+, 70), 160 (100), 145 (40), 129 (50), 115 (25), 86 (35)

[1n] 4-phenyl-1-tosyl-1,2,3,6-tetrahydropyridine

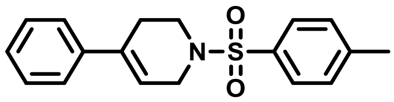

1n was prepared by dissolving **1p** (258 mg, 1.6 mmol) and triethyl amine (246 mg, 1.2 equiv) in CH_2Cl_2 (3 mL). The resulting mixture was cooled to 0 ºC then p-toluenesulfonyl chloride (309 mg, 1 equiv) was added portion-wise. The ice bath was removed after the addition of sulfonyl chloride was completed. The reaction was stirred for 4 h before transferring it into a separatory funnel. The organic layer was washed with water and brine sequentially then dried over $MgSO_4$. The product was concentrated *in vacuo* and was recrystallized in isopropanol to afford pure yellow crystals. The obtained yield is (369 mg, 73%).

mp: 205 – 207 ºC.

^{1}H NMR (800 MHz, $CDCl_3$) δ 7.76 – 7.72 (m, 2H), 7.37 – 7.30 (m, 6H), 7.29 – 7.25 (m, 2H), 5.98 (tt, *J* = 3.5, 1.6 Hz, 1H), 3.79 (q, *J* = 2.9 Hz, 2H), 3.35 (t, *J* = 5.7 Hz, 2H), 2.66 – 2.62 (m, 2H), 2.46 (s, 3H).

^{13}C{1H} NMR (201 MHz, $CDCl_3$) δ 143.8, 140.3, 135.6, 133.3, 129.8, 128.6, 127.9, 127.7, 125.1, 119.1, 45.4, 43.2, 27.7, 21.7.

GC/MS (m/z, relative intensity) 313 (M^+, 6), 158 (60), 143 (2), 131 (100), 115 (25), 103 (34), 91 (62), 80 (2)

[1o] 1-(4-phenyl-3,6-dihydropyridin-1(2H)-yl)ethan-1-one

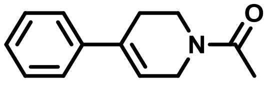

The amine precursor **1p** (252 mg, 1.57 mmole), dissolved in CH_2Cl_2 (13 mL) was set stirring in a 50 mL round-bottom flask before adding acetic anhydride (194 mg, 2.4 equiv) at room temperature. After stirring overnight, it was quenched with 5% aqueous NaOH solution and transferred to a separatory funnel. The aqueous layer was extracted twice with CH_2Cl_2 and combined with the original organic layer before washing with brine and drying over $MgSO_4$. The product was concentrated *in vacuo* and was obtained in 81% yield (256 mg) as a yellow solid mixture of rotamers without further purification.

mp: 59 – 63 ºC

^{1}H NMR (800 MHz, $CDCl_3$) δ 7.36 – 7.31 (m, 4H), 7.33 – 7.27 (m, 4H), 7.26 – 7.21 (m, 2H), 6.05 – 6.02 (m, 1H), 5.99 – 5.97 (m, 1H), 4.20 (q, *J* = 3.00 Hz, 2H), 4.08 (q, *J* = 2.98 Hz, 2H), 3.78 (t, *J* = 5.82 Hz, 2H), 3.61 (t, *J* = 5.78 Hz, 2H), 2.56 – 2.53 (m, 2H), 2.52 – 2.48 (m, 2H), 2.13 (s, 3H), 2.10 (s, 3H).

^{13}C{1H} NMR (201 MHz, $CDCl_3$) δ 169.2, 169.1, 140.2, 140.1, 136.6, 134.8, 128.4, 128.4, 127.4, 127.3, 124.8, 124.8, 120.9, 119.3, 45.7, 43.3, 42.1, 38.2, 27.8, 27.0, 21.8, 21.4.

GC/MS (m/z, relative intensity) 201 (M^+, 100), 143 (25), 130 (60), 115 (52), 103 (21), 91 (50), 82 (63)

[1p] 4-phenyl-1,2,3,6-tetrahydropyridine

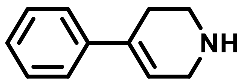

The corresponding Boc-alcohol **1s** (8.17 g, 29.5 mmol) was refluxed in TFA (33 mL) for an hour. After the reaction was completed, it was quenched by saturated 1M NaOH and extracted with DCM. The combined organic layers were washed with brine. The organic layer was separated, dried over MgSO4, and concentrated. The product was purified by flash column chromatography using 10% EtOAC: hexane. **1p** was obtained as a yellow liquid (3.42 g, 73% yield).

^{1}H NMR (800 MHz, $CDCl_3$) δ 7.42 – 7.38 (m, 2H), 7.37 – 7.33 (m, 2H), 7.30 – 7.26 (m, 1H), 6.12 (s, 1H), 5.14 (s, 2H), 3.65 (q, *J* = 2.9 Hz, 2H), 3.23 (t, *J* = 5.8 Hz, 2H), 2.60 (tq, *J* = 5.1, 2.3 Hz, 2H).

^{113}**C{1H} NMR** (201 MHz, $CDCl_3$) δ 140.8, 135.7, 128.5, 127.5, 125.0, 121.4, 44.6, 42.7, 26.8.

GC/MS (m/z, relative intensity) 159 (M^+, 100), 130 (60), 115 (60), 91 (40), 82 (60)

[1q] 1-(4-methoxybenzyl)-4-phenyl-1,2,3,6-tetrahydropyridine

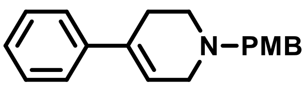

Acetic acid (94 mg, 1 equiv), sodium triacetoxyborohydride (666 mg, 1.5 equiv) and 4-methoxybenzaldehyde (235 mg, 1.1 equiv) were added to a solution of **1p** (250 mg, 1.57 mmol) in THF (5 mL). The reaction mixture was stirred and monitored by TLC for the disappearance of **1p**. When completely consumed, the reaction mixture was quenched with 1 M NaOH until pH of 9, concentrated under reduced pressure, and extracted into EtOAc. The organic layers were extracted with 1 M HCl, and the combined aqueous layers treated to saturated NaOH to reach a pH of 9. The basic solutions are then extracted with EtOAc, which is then dried over $MgSO_4$ and concentrated *in vacuo*. The amine product was purified by flash column chromatography using 10% EtOAC: hexane. **1q** was afforded as a yellow solid (147 mg, 34%)

mp: 77-79 °C

^{1}H NMR (800 MHz, $CDCl_3$) δ 7.38 (dd, *J* = 8.2, 1.5 Hz, 2H), 7.34 – 7.28 (m, 4H), 7.25 – 7.21 (m, 1H), 6.90 – 6.86 (m, 2H), 6.07 (t, *J* = 1.5 Hz, 1H), 3.82 (s, 3H), 3.59 (s, 2H), 3.16 (q, *J* = 3.0 Hz, 2H), 2.70 (t, *J* = 5.7 Hz, 2H), 2.59 – 2.54 (m, 2H).

^{13}C{1H} NMR (201 MHz, $CDCl_3$) δ 158.9, 141.1, 135.1, 130.5, 128.4, 127.0, 125.1, 122.1, 113.8, 62.2, 55.4, 53.4, 50.0, 28.2.

GC/MS (m/z, relative intensity) 279 (M^+, 10), 121(100), 91(20)

[1r] tert-butyl 4-(4-(trifluoromethyl)phenyl)-3,6-dihydropyridine-1(2H)-carboxylate

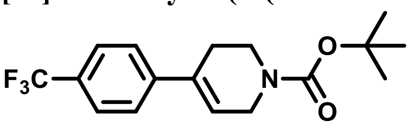

1r was prepared according to Scheme S2, the obtained yield is 93 % (4.89 g) as a yellow solid.

mp: 59 °C

^{1}H NMR (800 MHz, $CDCl_3$) δ 7.56 (d, *J* = 8.2 Hz, 2H), 7.45 (d, *J* = 8.2 Hz, 2H), 6.11 (s, 1H), 4.19 – 3.99 (m, 2H), 3.70 – 3.56 (m, 2H), 2.61 – 2.37 (m, 2H), 1.49 (s, 9H).

^{13}C{1H} NMR (201 MHz, $CDCl_3$) δ 154.9, 144.2, 134.7, 129.2 (q, *J* = 32.4 Hz), 125.5 (q, *J* = 3.8 Hz), 125.3, 124.3 (q, *J* = 271.8 Hz), 123.0, 79.9, 43.8 (d, *J* = 116.2 Hz), 40.4 (d, *J* = 260.2 Hz), 28.5, 27.4.

^{19}F NMR (753 MHz, $CDCl_3$) δ -62.5.

GC/MS (m/z, relative intensity) 227(M-($CO_2C(CH_3)_3^+$, 100), 198(25), 159(27), 129 (72), 82(75)

[1s] *tert*-butyl 4-phenyl-3,6-dihydropyridine-1(2H)-carboxylate

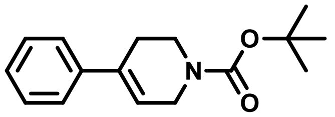

1s was prepared according to Scheme S2, obtained in 59% yield (1.1 g) as a yellow solid. The characteristic data are consistent with Daniel Rich's 2001 report.[10]

mp: 51-53 °C

^{1}H NMR (800 MHz, $CDCl_3$) δ 7.41 – 7.38 (m, 2H), 7.35 (dd, J = 8.7, 6.9 Hz, 2H), 7.28 (t, J = 7.1 Hz, 1H), 6.04 (s, 1H), 4.10 (s, 2H), 3.70 – 3.61 (m, 2H), 2.55 (s, 2H), 1.53 (s, 10H).

^{13}C{1H} NMR (201 MHz, $CDCl_3$) δ 154.9, 140.7, 135.4 (d, J = 51.5 Hz), 128.4, 127.2, 124.9, 120.9 (d, J = 110.9 Hz), 79.6, 43.7 (d, J = 117.2 Hz), 40.5 (d, J = 259.7 Hz), 28.5, 27.4.

GC/MS (m/z, relative intensity) 202 (M-$C(CH_3)_3^+$, 100), 159 (85), 142 (40), 130 (75), 115 (65), 91 (50), 82 (50)

[1t] tert-butyl 4-(4-methoxyphenyl)-3,6-dihydropyridine-1(2H)-carboxylate

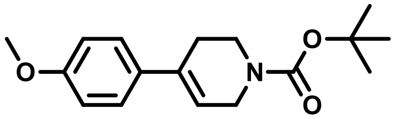

1t was prepared according to Scheme S2, obtained in 72% yield (3.7 g) as a yellow solid.

mp: 65 °C

^{1}H NMR (800 MHz, $CDCl_3$) δ 7.35 – 7.31 (m, 2H), 6.90 – 6.87 (m, 2H), 5.95 (s, 1H), 4.13 – 4.03 (m, 2H), 3.82 (s, 3H), 3.70 – 3.59 (m, 2H), 2.57 – 2.44 (m, 2H), 1.51 (s, 9H).
^{13}C{1H} NMR (201 MHz, $CDCl_3$) δ 159.0, 155.0, 134.9, 133.4, 126.1, 119.6, 113.8, 79.7, 55.4, 43.7 (d, J = 116.7 Hz), 40.6 (d, J = 264.0 Hz), 28.6, 27.5.
GC/MS (m/z, relative intensity) 189(M-($CO_2C(CH_3)_3^+$, 100), 174(25), 160(60), 145(45), 129(21), 121(30), 115 (35), 103(10), 91(25), 89(15), 82(35)

[1u] N-(2,3,4,5-tetrahydro-[1,1'-biphenyl]-4-yl)acetamide

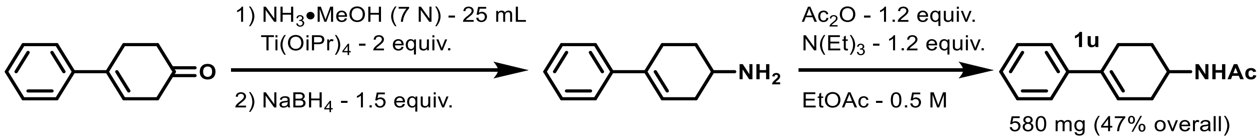

2,5-dihydro-[1,1'-biphenyl]-4(3H)-one, prepared according to the literature,[8] was subjected to $Ti(OiPr)_4$ in methanolic ammonia and subsequently $NaBH_4$ following a previous report.[11] The corresponding amine (500 mg, 2.9 mmol) was treated to acetic anhydride (360 mg, 3.5 mmol) and triethylamine (360 mg, 3.5 mmol), stirring for 30 minutes. The reaction was quenched and purified analogously to the procedure reported by Lapkin,[12] affording **1u** as a waxy brown solid (580 mg, 47% yield over 2 steps).

mp: 99-100 °C

^{1}H NMR (800 MHz, CDCl3) δ 7.38 – 7.36 (m, 2H), 7.31 (t, J = 7.8 Hz, 2H), 7.25 – 7.22 (m, 1H), 6.03 (tt, J = 3.7, 1.8 Hz, 1H), 5.69 (d, J = 8.1 Hz, 1H), 4.21 – 4.15 (m, 1H), 2.58 (dddd, J = 20.3, 10.8, 4.8, 2.4 Hz, 2H), 2.53 – 2.48 (m, 1H), 2.11 – 2.05 (m, 1H), 2.03 – 1.97 (m, 4H), 1.80 – 1.73 (m, 1H).

^{13}C{1H} NMR (201 MHz, $CDCl_3$) δ 169.8, 141.4, 136.6, 128.4, 127.1, 125.1, 121.7, 44.4, 32.3, 28.4, 25.6, 23.7.

GC/MS (m/z, relative intensity) 216 (M+H^+, 5), 156 (100), 141 (30), 129 (30), 128 (25), 115 (25), 91 (20)

[1v] 1-phenylcyclohept-1-ene

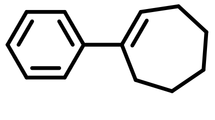

1v was obtained in 90% yield (2.45 g) following the general procedure as a colorless oil. It is well documented in the literature, and the characteristic 1H NMR spectrum was a match.[13]

^{1}H NMR (400 MHz, $CDCl_3$) δ 7.36 – 7.17 (m, 5H), 6.11 (t, J = 6.8 Hz, 1H), 2.67 – 2.59 (m, 2H), 2.35 – 2.26 (m, 2H), 1.91 – 1.81 (m, 2H), 1.71 – 1.62 (m, 2H), 1.62 – 1.53 (m, 2H)

^{13}C{1H} NMR (201 MHz, $CDCl_3$) δ 145.1, 145.1, 130.6, 128.2, 126.4, 125.8, 32.9, 29.0, 27.1, 26.9.

Scheme S3 Preparation of PF_6^-
Brønsted Acid Salts

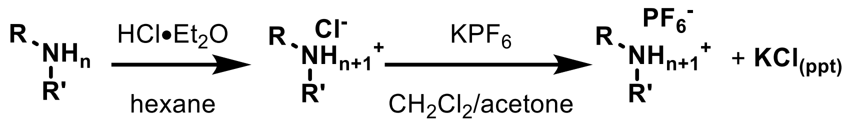

Dry ethereal HCl was added dropwise to a stirring solution of amine or basic heterocycle in hexane (4:1 v/v) until visible precipitate no longer formed. The precipitate was isolated by centrifugation and the hexanes/ether supernatant was decanted. The resultant white powder was then thrice cycled through washing with anhydrous ether, centrifugation, and decanting before dissolving in CH_2Cl_2 to a saturated solution. This was added to a stirring saturated solution of 1.1 equivalents of potassium hexafluorophosphate in acetone, which precipitated KCl while leaving the desired PF_6^- salt in solution. The precipitate was subjected to the same cycle of washing and centrifugation, except with CH_2Cl_2 instead of ether. The supernatant was concentrated in vacuo to afford the target acidic hexafluorophosphate salt.

Scheme S3.1 Preparation of Pyridinium salts with counter ions other than PF_6^-

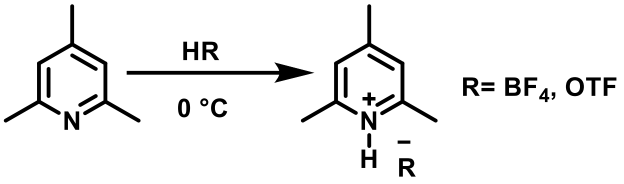

To synthesize 2,4,6-trimethylpyridinium tetrafluoroborate, 300 mg (2.48 mmole, 1 equiv) of collidine was added to a small round-bottom flask with a stirring bar and placed in an ice bath. While stirring, 1088 mg (5.95 mmole) of 48% HBF4 was added dropwise to the collidine. The mixture was stirred for 10 minutes, during which a white precipitate formed. After 10 minutes, the water was decanted, and the resulting white solid was washed twice with ether, yielding 518 mg of product (99% yield). The synthesis of collidinium triflate was performed similarly, using 2 g (16.52 mmole) of collidine and 2.48 g (16.52 mmole) of HOTf, resulting in a yield of 3.9 g (87%). The characteristic data for collidine●HBF_4 and collidine●HOTf were consistent with Nagao and Ohmiya's 2022 report.[14]

Scheme S4 Representative Photochemical FC Reaction

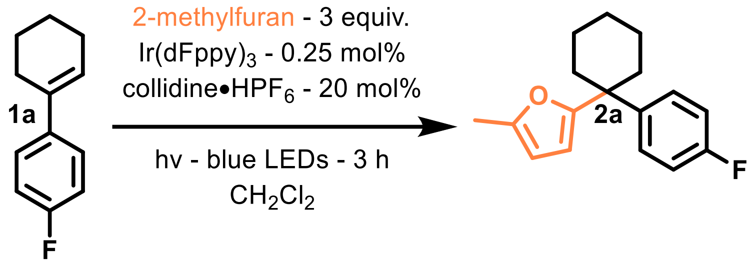

A generalized procedure for the FC arylcyclohexylation can be understood from the example of adduct 2-(1-(4-fluorophenyl)cyclohexyl)-5-methylfuran (**2a**). Although not all reactions were prepared on the exact same scale, the proportions for most FC reactions were maintained as specified. However, in a few cases, the proportions deviated slightly from those in Scheme S4. Into an NMR tube charged with a C_6D_6 capillary was measured collidine●HPF_6 (21 mg, 77 µmol) followed by sealing the tube with a rubber septum and parafilm. The rubber septum was pierced with an exit needle and a long needle connected to argon flow before briefly heating with a flame to remove water vapor. The tube was then cooled to 0 °C by immersion into an ice bath while maintaining argon flow. Through the septum was then injected 1-(4-fluorophenyl)cyclohexene (**1a**, 69 mg, 390 µmol), 2-methylfuran (96 mg, 1.17 mmol), and a stock solution of Ir(dFppy)$_3$ in CH_2Cl_2 (1.13 mM, 870 µL –0.0025 equiv.) before diluting the mixture to 1 mL total volume with CH_2Cl_2. Argon was bubbled through the cooled solution for 10 minutes before removing the needles, further sealing the septum with more parafilm, and shaking to ensure homogeneity in the reaction mixture. NMR spectra (^{1}H, ^{13}C, and ^{19}F) were collected prior to and sporadically over the course of irradiation with the LEDs depicted in **Figure S1** at ca. 10 °C. When deemed complete by ^{1}H NMR (3 hours in this case, yet often 8-12 hours for others) the reaction mixture was removed from the tube and eluted through a silica gel plug with hexane before concentrating under vacuum to afford the product **2a.** In instances where phenyl cyclohexenes such as **1i**, **1j**, and **1k** were used, two diastereomeric FC products could be formed, namely **2i**/**2i'**, **2j**/**2j'**, and **2k**. The stereochemistry of these products was determined through structural assignments, which were supported by additional information from gHSQC experiments. Substrate **2k** was synthesized on a 1 mmol scale (S22).

Figure S2 Counterion Screening

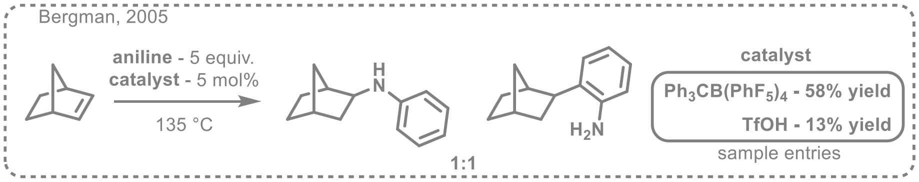

Entry	X	time (min)	conversion to 2b
1	PF_6^-	10	11
		30	30
		60	59
		120	100
2	BF_4^-	10	11
		30	21
		60	33
		120	48
		780	100
3	TfO^-	10	7
		30	8
		60	9
		120	9
		780	34
4	Cl^-	10	7
		30	7
		60	7
		120	7
		780	10

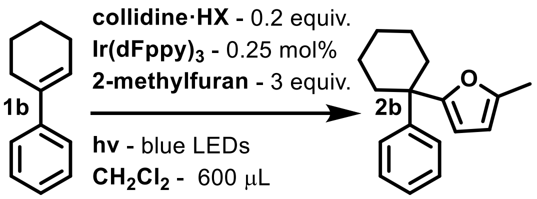

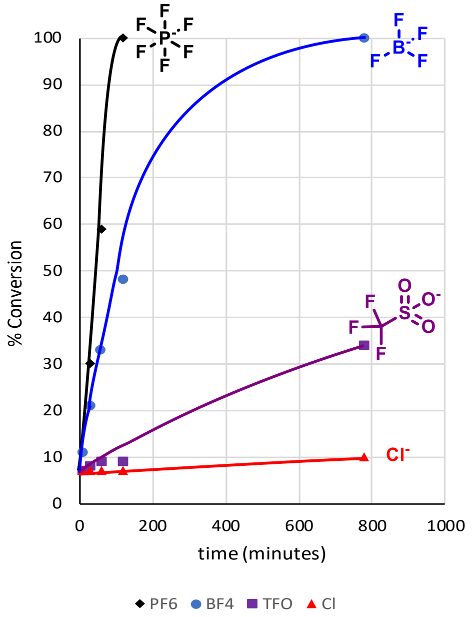

In 2005 Bergman reported a significant influence on yields of related acid-mediated alkene hydroamination reactions involving carbenium ions when comparing their counter anions in terms of coordinative ability.[15] He observed yield variation from 0 to 60% for norbornene hydroamination/FC arylation with seven anilinium-anion salts. The least coordinating anions led to the highest yields. To observe the extent of influence the counter-anion would have on our reactivity, a series of four parallel experiments were carried out, keeping the acidic cation, 2,4,6-collidinium, constant (Figure 2). The poorly soluble chloride anion limited concentration to 0.21 M and performed poorly. While hexafluorophosphate was clearly the superior option in terms of reaction rate, tetrafluoroborate was not far behind. Triflate was significantly slower, though led to similar yields in time. Collidine●HPF_6 remained the Bronsted acid catalyst of choice for subsequent reactions.

Characteristic Datasheets for FC Products

[2a] 2-(1-(4-fluorophenyl)cyclohexyl)-5-methylfuran

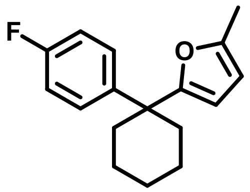

The reaction was conducted following the procedure outlined in Scheme S4, using 1a cyclohexene (69 mg, 0.39 mmol, 1 equiv), identified as 4'-fluoro-2,3,4,5-tetrahydro-1,1'-biphenyl, along with 2-methylfuran (96 mg, 1.17 mmol, 3 equiv), a stock solution of $Ir(dFppy)_3$ in CH_2Cl_2 (0.68 μM, 1.43 mL, 0.0025 equiv), and collidine•HPF_6 (21 mg, 0.08 mmol, 0.2 equiv), maintaining a reaction mixture concentration of 115 mM. After completion, the reaction mixture was purified by silica gel column chromatography with 100% hexane, yielding 2a as a beige oil (93 mg, 93% yield).

^{1}H NMR (800 MHz, $CDCl_3$) δ 7.23 – 7.18 (m, 2H), 6.98 – 6.92 (m, 2H), 5.93 (d, *J* = 3.1 Hz, 1H), 5.88 (dq, *J* = 3.2, 1.1 Hz, 1H), 2.29 (dt, *J* = 13.1, 4.8 Hz, 2H), 2.23 (d, *J* = 1.1 Hz, 3H), 2.00 – 1.95 (m, 2H), 1.61 – 1.54 (m, 3H), 1.54 – 1.49 (m, 2H), 1.42 – 1.31 (m, 1H).

^{13}C{1H} NMR (201 MHz, $CDCl_3$) δ 161.1 (d, *J* = 244.1 Hz), 158.3, 150.6, 143.7, 128.1 (d, *J* = 7.7 Hz), 114.9 (d, *J* = 20.8 Hz), 106.9, 105.9, 44.0, 36.1, 26.2, 23.0, 13.7.

^{19}F NMR (753 MHz, $CDCl_3$) δ -118.0 (tt, *J* = 9.0, 5.3 Hz).

GC/MS (m/z, relative intensity) 258 (M^+, 75), 215 (100), 202 (30), 189 (40), 159 (25), 133 (25), 109 (25), 95 (35)

[2b] 2-methyl-5-(1-phenylcyclohexyl)furan

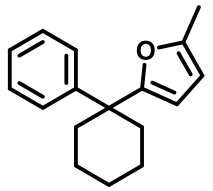

The reaction followed the procedure in Scheme S4, using **1b** cyclohexene (69 mg, 0.42 mmol, 1 equiv), identified as 2,3,4,5-tetrahydro-1,1'-biphenyl, along with 2-methylfuran (103 mg, 1.26 mmol, 3 equiv), a stock solution of $Ir(dFppy)_3$ in CH_2Cl_2 (0.65 μM, 1.6 mL, 0.0025 equivalents), and collidine•HPF_6 (22 mg, 0.08 mmol, 0.2 equivalents), with the reaction mixture concentration set to 0.1 M. Upon completion, the reaction mixture was purified using a silica gel plug with 100% hexane, yielding **2b** as a colorless crystalline solid (104 mg, 99% yield). Characteristic data was consistent with McCubbins' report.[16]

mp: 48 °C

^{1}H NMR (800 MHz, $CDCl_3$) δ 7.31 – 7.23 (m, 4H), 7.16 (tt, *J* = 6.6, 1.6 Hz, 1H), 5.95 (d, *J* = 3.1 Hz, 1H), 5.88 (dq, *J* = 3.1, 1.1 Hz, 1H), 2.35 – 2.29 (m, 2H), 2.23 (d, *J* = 1.0 Hz, 3H), 2.02 (ddd, *J* = 13.7, 10.3, 3.3 Hz, 2H), 1.62 – 1.57 (m, 3H), 1.56 – 1.49 (m, 2H), 1.39 – 1.33 (m, 1H).

^{13}C{1H} NMR (201 MHz, $CDCl_3$) δ 158.4, 150.4, 148.0, 128.3, 126.5, 125.9, 106.9, 105.8, 44.5, 35.9, 26.2, 23.1, 13.8.

GC/MS (m/z, relative intensity) 240 (M^+, 90), 197 (100), 184 (25), 171 (40), 155 (30), 141 (40), 115 (45), 95 (35), 91 (35)

[2c] 2-methyl-5-(1-(p-tolyl)cyclohexyl)furan

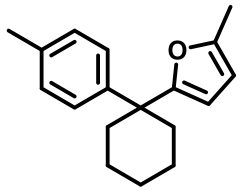

The reaction was set up following the procedure outlined in Scheme S4, starting with **1c** cyclohexene (100 mg, 0.58 mmol), identified as 4'-methyl-2,3,4,5-tetrahydro-1,1'-biphenyl, along with 2-methylfuran (143 mg, 1.74 mmol, 3 equiv), a stock solution of $Ir(dFppy)_3$ in CH_2Cl_2 (1.32 mM, 1.1 mL, 0.0025 equiv), and collidine•HPF_6 (31 mg, 0.12 mmol, 0.2 equiv). With a reaction mixture concentration of 529 mM, the product **2c** was obtained as a white crystalline solid (116 mg, 79% yield) after purification using a small silica gel plug with 100% hexane.

mp: 52-53 °C

^{1}H NMR (800 MHz, $CDCl_3$) δ 7.31 – 7.24 (m, 2H), 7.23 – 7.19 (m, 2H), 6.06 (d, *J* = 3.3 Hz, 1H), 6.01 – 5.97 (m, 1H), 2.46 – 2.40 (m, 4H), 2.35 (d, *J* = 1.7 Hz, 3H), 2.14 (ddd, *J* = 14.0, 10.5, 3.4 Hz, 2H), 1.75 – 1.62 (m, 6H), 1.53 – 1.46 (m, 1H).

^{13}C{1H} NMR (201 MHz, $CDCl_3$) δ 158.8, 150.5, 145.2, 135.4, 129.1, 126.6, 106.9, 106.0, 44.3, 36.1, 26.4, 23.2, 21.1, 13.9.

GC/MS (m/z, relative intensity) 254 (M^+, 80), 239 (50), 211 (100), 185 (40)

[2d] 2-(1-(3-fluorophenyl)cyclohexyl)-5-methylfuran

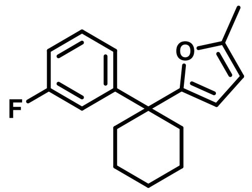

The reaction was conducted following the procedure in Scheme S4, using **1d** cyclohexene (69 mg, 0.39 mmol, 1 equiv), identified as 3'-fluoro-2,3,4,5-tetrahydro-1,1'-biphenyl, along with 2-methylfuran (96 mg, 1.17 mmol, 3 equiv), a stock solution of $Ir(dFppy)_3$ in CH_2Cl_2 (0.68 µM, 1.43 mL, 0.0025 equivalents), and collidine•HPF_6 (21 mg, 0.08 mmol, 0.2 equivalents), with the reaction mixture concentration set to 0.1 M. The product, **2d**, was obtained as a colorless crystalline solid (80 mg, 80% yield) following purification by silica gel column chromatography using 100% hexane.

mp: 47 °C

^{1}H NMR (800 MHz, $CDCl_3$) δ 7.22 (td, *J* = 8.0, 6.3 Hz, 1H), 7.03 (dt, *J* = 7.9, 0.9 Hz, 1H), 6.95 (dt, *J* = 11.3, 2.2 Hz, 1H), 6.85 (tdd, *J* = 8.2, 2.7, 0.9 Hz, 1H), 5.96 (d, *J* = 3.1 Hz, 1H), 5.89 (dq, *J* = 3.2, 1.0 Hz, 1H), 2.33 – 2.27 (m, 2H), 2.23 (d, *J* = 1.1 Hz, 3H), 1.97 (ddd, *J* = 13.8, 10.3, 3.1 Hz, 2H), 1.62 – 1.55 (m, 3H), 1.55 – 1.48 (m, 2H), 1.40 – 1.33 (m, 1H).

^{13}C{1H} NMR (201 MHz, $CDCl_3$) δ 162.9 (d, *J* = 243.8 Hz), 157.6, 150.6 (d, *J* = 0.7 Hz), 129.4 (d, *J* = 8.2 Hz), 125.9, 122.0 (d, *J* = 2.0 Hz), 113.6 (d, *J* = 22.1 Hz), 112.6 (d, *J* = 21.4 Hz), 106.9, 105.8, 44.3, 35.7, 26.0, 22.8, 13.6.

GC/MS (m/z, relative intensity) 258 (M^+, 95), 215 (100), 202 (30), 189 (30), 133 (25), 95 (30)

[2e] 2-(1-(4-methoxyphenyl)cyclohexyl)-5-methylfuran

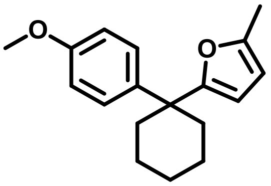

The reaction was set up according to the procedure in Scheme S4, using **1e** cyclohexene (100 mg, 0.53 mmol), identified as 4'-methoxy-2,3,4,5-tetrahydro-1,1'-biphenyl, along with 2-methylfuran (131 mg, 1.6 mmol, 3 equiv), a stock solution of $Ir(dFppy)_3$ in CH_2Cl_2 (1.33 µM, 1 mL, 0.0025 equiv), and collidine•HPF_6 (28 mg, 0.11 mmol, 0.2 equiv), with the reaction mixture concentration set to 530 mM. Product **2e** was obtained as a beige powder (135 mg, 94% yield) after purification through a silica gel plug with hexane.

mp: 68-69 °C

^{1}H NMR (800 MHz, $CDCl_3$) δ 7.24 – 7.20 (m, 2H), 6.88 – 6.84 (m, 2H), 5.96 (d, *J* = 3.2 Hz, 1H), 5.93 – 5.89 (m, 1H), 3.80 (s, 3H), 2.35 – 2.31 (m, 2H), 2.27 (d, *J* = 1.5 Hz, 3H), 2.04 (ddd, *J* = 13.9, 10.2, 3.5 Hz, 2H), 1.65 – 1.58 (m, 4H), 1.60 – 1.52 (m, 2H), 1.45 – 1.37 (m, 1H).

^{13}C{1H} NMR (201 MHz, $CDCl_3$) δ 158.8, 157.6, 150.3, 140.1, 127.5, 113.5, 106.6, 105.8, 55.2, 43.8, 36.1, 26.2, 23.0, 13.7.

GC/MS (m/z, relative intensity) 270 (M^+, 80), 227 (100), 201 (40), 95 (25)

[2f] 5-(1-(5-methylfuran-2-yl)cyclohexyl)benzo[d][1,3]dioxole

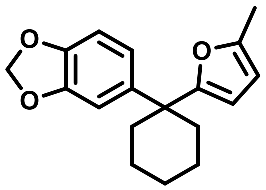

The reaction was set up according to the procedure in Scheme S4, using **1f** cyclohexene (75 mg, 0.37 mmol), identified as 5-(cyclohex-1-en-1-yl)benzo[d][1,3]dioxole, along with 2-methylfuran (91 mg, 1.11 mmol, 3 equiv), a stock solution of $Ir(dFppy)_3$ in CH_2Cl_2 (0.56 mM, 1.3 mL, 0.0025 equiv), and collidine•HPF_6 (20 mg, 0.075 mmol, 0.2 equiv). With a reaction mixture concentration of 280 mM, compound **2f** was obtained as colorless crystals (94 mg, 89% yield).

mp: 96-98 °C

^{1}H NMR (800 MHz, $CDCl_3$) δ 6.79 (d, *J* = 2.0 Hz, 1H), 6.74 – 6.73 (m, 2H), 5.95 (d, *J* = 3.1 Hz, 1H), 5.91 (s, 2H), 5.89 (dd, *J* = 3.0, 1.2 Hz, 1H), 2.31 – 2.27 (m, 2H), 2.24 (d, *J* = 1.3 Hz, 3H), 1.96 (ddd, *J* = 13.8, 10.5, 3.4 Hz, 2H), 1.63 – 1.48 (m, 5H), 1.41 – 1.32 (m, 1H).

^{13}C{1H} NMR (201 MHz, $CDCl_3$) δ 158.3, 150.3, 147.5, 145.4, 142.2, 119.2, 107.8, 107.4, 106.6, 105.8, 100.8, 44.1, 36.1, 26.1, 23.0, 13.7.

GC/MS (m/z, relative intensity) 284 (M^+, 95), 241 (100), 228 (25), 215 (30), 211 (40), 183 (30)

[2g] 2-methyl-5-(1-(4-(trifluoromethyl)phenyl)cyclohexyl)furan

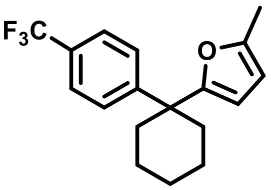

The reaction was set up following the procedure in Scheme S4, using **1g** cyclohexene (100 mg, 0.442 mmol, 1 equiv), specifically 4'-(trifluoromethyl)-2,3,4,5-tetrahydro-1,1'-biphenyl, along with 2-methylfuran (108 mg, 1.3 mmol, 3 equiv), a stock solution of $Ir(dFppy)_3$ in CH_2Cl_2 (1 μM, 1 mL, 0.0025 equiv), and collidine•HPF_6 (48 mg, 0.176 mmol, 0.4 equiv), with the reaction mixture concentration adjusted to 0.1 M. The total volume was diluted to 4 mL in a glass culture tube (instead of an NMR tube). Flash column chromatography on the crude reaction mixture, eluting with pure hexane, separated out the cyclodimer **2gg**, yielding **2g** as a colorless oil (35 mg, 26% yield).

^{1}H NMR (800 MHz, $CDCl_3$) δ 7.54 – 7.51 (m, 2H), 7.37 (d, *J* = 8.3 Hz, 2H), 6.00 (d, *J* = 3.1 Hz, 1H), 5.90 (dq, *J* = 3.1, 1.1 Hz, 1H), 2.33 (dq, *J* = 15.2, 4.5 Hz, 2H), 2.23 (d, *J* = 1.2 Hz, 3H), 2.06 – 1.99 (m, 2H), 1.62 – 1.58 (m, 3H), 1.58 – 1.52 (m, 2H), 1.42 – 1.35 (m, 1H).

^{13}C{1H} NMR (201 MHz, $CDCl_3$) δ 157.4, 152.1, 150.9, 128.2 (q, *J* = 33.0 Hz), 126.9, 125.2 (q, *J* = 3.7 Hz), 124.5 (q, *J* = 272.0 Hz), 107.3, 106.0, 44.6, 35.8, 26.1, 22.9, 13.7.

GC/MS - CI (m/z, relative intensity) 308 (M^+, 80), 289 (100), 265 (20), 227 (40), 163 (35)

[2gg] 4a,4b-bis(4-(trifluoromethyl)phenyl)dodecahydrobiphenylene

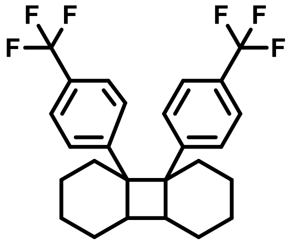

As described for entry **2g** above, cyclodimer **2gg** was obtained as a colorless oil (22 mg, 22% of the yield).

^{1}H NMR (800 MHz, $CDCl_3$) δ 7.65 (d, *J* = 8.1 Hz, 4H), 7.56 (d, *J* = 8.1 Hz, 4H), 2.95 – 2.88 (m, 2H), 1.75 – 1.65 (m, 4H), 1.63 – 1.58 (m, 2H), 1.53 (dt, *J* = 13.8, 3.4 Hz, 2H), 1.44 (td, *J* = 13.9, 3.2 Hz, 2H), 1.38 (qdd, *J* = 13.1, 5.4, 3.1 Hz, 2H), 1.28 (dt, *J* = 13.6, 3.6 Hz, 2H), 0.73 (qt, *J* = 13.4, 2.8 Hz, 2H).

^{13}C{1H} NMR (201 MHz, $CDCl_3$) δ 150.7, 128.2 (q, *J* = 32.3 Hz), 128.1, 125.1 (q, *J* = 3.7 Hz), 124.5 (q, *J* = 271.8 Hz), 51.3, 34.8, 33.2, 23.4, 21.8, 21.1.

^{19}F NMR (753 MHz, $CDCl_3$) δ -62.3.

GC/MS - CI (m/z, relative intensity) 479 (M+Et^+, 5), 465 (M+Me^+, 40), 447 (50), 431 (50), 287 (10), 269 (10), 259 (40), 241 (40), 227 (60), 225 (100), 221 (25), 207 (60), 131 (25), 117 (20), 105 (10)

[3a] 2-(1-(4-fluorophenyl)cyclohexyl)-5-methylthiophene

The reaction was carried out according to the procedure in Scheme S4, using **1a** cyclohexene (48 mg, 0.27 mmol, 1 equivalent), identified as 4'-fluoro-2,3,4,5-tetrahydro-1,1'-biphenyl, along with 2-methylthiophene (72 mg, 0.82 mmol, 3 equivalents), a stock solution of $Ir(dFppy)_3$ in CH_2Cl_2 (0.68 μmol, 0.0025 equivalents), and collidine•HPF_6 (15 mg, 0.05 mmol, 0.2 equivalents), with the reaction mixture concentration set to 248 mM. The product, **3a**, was obtained as a colorless oil (65 mg, 87% yield) following purification with a silica plug using 100% hexane.

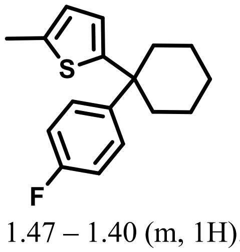

^{1}H NMR (800 MHz, $CDCl_3$) δ 7.31 – 7.26 (m, 2H), 6.99 – 6.94 (m, 2H), 6.56 – 6.52 (m, 2H), 2.39 (d, *J* = 1.1 Hz, 3H), 2.26 – 2.22 (m, 2H), 2.21 – 2.16 (m, 2H), 1.69 – 1.59 (m, 2H), 1.57 – 1.49 (m, 3H), 1.47 – 1.40 (m, 1H).

^{13}C{1H} NMR (201 MHz, $CDCl_3$) δ 161.0 (d, *J* = 244.6 Hz), 152.4, 144.0, 137.9, 128.2 (d, *J* = 7.7 Hz), 124.4, 123.3, 114.9 (d, *J* = 20.7 Hz), 45.0, 38.9, 26.1, 22.9, 15.3.

^{19}F NMR (753 MHz, $CDCl_3$) δ -117.8 (dt, *J* = 14.7, 7.5 Hz).

GC/MS (m/z, relative intensity) 274 (M^+, 90), 259 (30), 231 (100), 205 (30), 133 (70), 111 (40)

[4b] 2,5-bis(1-phenylcyclohexyl)thiophene

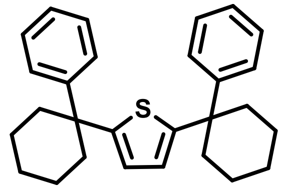

The reaction was conducted following the procedure in Scheme S4, using **1b** cyclohexene (332 mg, 2.1 mmol, 2.1 equiv), specifically 2,3,4,5-tetrahydro-1,1'-biphenyl, along with thiophene (84 mg, 1 mmol, 1 equiv), a stock solution of Ir(dFppy)$_3$ in CH_2Cl_2 (1.07 µM, 700 µL, 0.0025 equiv), and collidine•HPF_6 (27 mg, 0.1 mmol, 0.1 equiv), with the reaction mixture concentration set to 1 M. After irradiating the reaction for 30 hours, the crude mixture was purified by flash column chromatography with pure hexane as the eluent, yielding **4b** as a white solid (235 mg, 69% yield).

mp: 113-114 °C

^{1}H NMR (800 MHz, $CDCl_3$) δ 7.36 – 7.32 (m, 4H), 7.32 – 7.27 (m, 4H), 7.18 (tdt, *J* = 7.2, 2.3, 1.2 Hz, 2H), 6.48 (s, 2H), 2.35 – 2.30 (m, 4H), 2.17 (ddd, *J* = 13.6, 8.1, 3.5 Hz, 4H), 1.67 – 1.59 (m, 4H), 1.56 – 1.43 (m, 8H).

^{13}C{1H} NMR (201 MHz, $CDCl_3$) δ 152.8, 147.9, 128.3, 126.9, 125.8, 122.9, 45.6, 38.9, 26.2, 23.0.

GC/MS (m/z, relative intensity) 400 (M^+, 90), 323 (55), 159 (100)

[5b/b'] 2/3-(1-phenylcyclohexyl)-1H-pyrrole

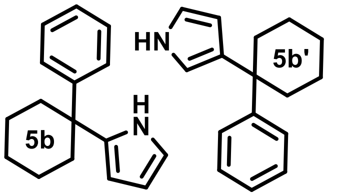

The reaction was set up according to the procedure in Scheme S4, using **1b** cyclohexene (100 mg, 0.633 mmol), identified as 2,3,4,5-tetrahydro-1,1'-biphenyl, along with pyran (127 mg, 1.9 mmol, 3 equiv), a stock solution of Ir(dFppy)$_3$ in CH_2Cl_2 (1.32 µM, 1.2 mL, 0.0025 equiv), and collidine•HPF_6 (34 mg, 0.13 mmol, 0.2 equiv), with the reaction mixture concentration set to 541 mM. Products **5b** and **5b'** were obtained as a beige powder and dark brown solid, respectively (111 mg and 23 mg, 94% combined yield). Purification was carried out using silica gel flash chromatography with 10% EtOAc in hexanes, rather than a simple silica gel plug.

mp: 60-61 °C

^{1}H NMR (800 MHz, $CDCl_3$) δ 7.64 (s, 1H), 7.35 – 7.30 (m, 2H), 7.30 – 7.28 (m, 2H), 7.21 (tt, *J* = 7.2, 1.1 Hz, 1H), 6.64 (td, *J* = 2.6, 1.5 Hz, 1H), 6.20 (q, *J* = 2.9 Hz, 1H), 6.15 (td, *J* = 3.1, 1.6 Hz, 1H), 2.25 (ddd, *J* = 13.0, 8.9, 3.2 Hz, 2H), 2.21 – 2.15 (m, 2H), 1.69 – 1.62 (m, 2H), 1.62 – 1.56 (m, 3H), 1.53 – 1.45 (m, 1H).

^{13}C{1H} NMR (201 MHz, $CDCl_3$) δ 147.9, 138.6, 128.5, 126.8, 126.0, 116.5, 107.9, 105.4, 43.5, 37.4, 26.3, 23.0.

GC/MS (m/z, relative intensity) 225 (M^+, 85), 182 (100), 167 (25), 156 (30), 154 (32), 115 (90), 80 (45)

5b'

mp: 71-72 °C

^{1}H NMR (800 MHz, $CDCl_3$) δ 7.97 (s, 1H), 7.38 – 7.35 (m, 2H), 7.30 – 7.27 (m, 2H), 7.14 (tt, *J* = 7.3, 1.3 Hz, 1H), 6.71 (q, *J* = 2.5 Hz, 1H), 6.45 (q, *J* = 2.1 Hz, 1H), 6.07 (td, *J* = 2.7, 1.6 Hz, 1H), 2.24 (ddd, *J* = 13.0, 8.4, 3.1 Hz, 2H), 2.14 (ddd, *J* = 13.3, 7.9, 3.5 Hz, 2H), 1.65 – 1.58 (m, 2H), 1.57 – 1.49 (m, 3H), 1.49 – 1.43 (m, 1H).

^{13}C{1H} NMR (201 MHz, $CDCl_3$) δ 149.9, 132.9, 128.1, 126.9, 125.3, 117.7, 115.0, 107.5, 42.3, 38.0, 26.6, 23.1.

GC/MS (m/z, relative intensity) 225 (M^+, 85), 182 (90), 167 (25), 156 (30), 154 (20), 129 (20), 115 (100), 91 (20), 80 (40)

[6b] 6-fluoro-3-(1-phenylcyclohexyl)-1H-indole

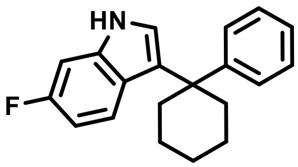

The reaction was set up following the procedure in Scheme S4, using **1b** cyclohexene (75 mg, 0.47 mmol, 1.1 equiv), identified as 2,3,4,5-tetrahydro-1,1'-biphenyl, along with 6-fluoro-1H-indole (58 mg, 0.43 mmol, 1 equiv), a stock solution of Ir(dFppy)$_3$ in CH_2Cl_2 (1 µM, 1 mL, 0.0025 equiv), and triethylamine•HPF_6 (20 mg, 0.086 mmol, 0.2 equiv), with the reaction mixture concentration set to 500 mM. Product **6b** was obtained as a beige powder (113 mg, 90% yield) after purification by silica gel flash chromatography using 10% EtOAc in hexanes, rather than a simple silica gel plug.

mp: 90 °C

^{1}H NMR (800 MHz, $CDCl_3$) δ 7.92 (s, 1H), 7.40 – 7.37 (m, 2H), 7.28 – 7.23 (m, 2H), 7.14 (tt, *J* = 7.2, 1.3 Hz, 1H), 7.02 (d, *J* = 2.4 Hz, 1H), 6.97 (dd, *J* = 9.5, 2.4 Hz, 1H), 6.67 (td, *J* = 9.2, 2.4 Hz, 1H), 2.40 (ddd, *J* = 13.7, 7.1, 4.1 Hz, 2H), 2.30 (ddd, *J* = 13.3, 8.2, 4.2 Hz, 2H), 1.67 – 1.54 (m, 5H), 1.53 – 1.46 (m, 1H).

^{13}C{1H} NMR (201 MHz, $CDCl_3$) δ 159.6 (d, *J* = 237.7 Hz), 148.8, 137.1 (d, *J* = 12.4 Hz), 128.2, 127.2, 125.6, 124.2, 122.8, 122.5 (d, *J* = 3.5 Hz), 122.1 (d, *J* = 10.0 Hz), 107.7 (d, *J* = 23.8 Hz), 97.3 (d, *J* = 25.8 Hz), 43.2, 37.4, 26.7, 23.1.

^{19}F NMR (753 MHz, $CDCl_3$) δ -122.0 (td, *J* = 9.4, 5.6 Hz).

GC/MS (m/z, relative intensity) 293 (M^+, 95), 274 (35), 250 (15), 216 (35), 159 (100)

[7b] 3-(1-phenylcyclohexyl)benzofuran

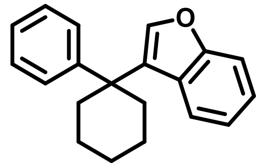

The reaction was set up according to the procedure in Scheme S4, using **1b** cyclohexene (100 mg, 0.633 mmol), identified as 2,3,4,5-tetrahydro-1,1'-biphenyl, along with benzofuran (224 mg, 1.9 mmol, 3 equiv), a stock solution of Ir(dFppy)$_3$ in CH_2Cl_2 (1.32 µM, 1.2 mL, 0.0025 equiv), and collidine•HPF_6 (39 mg, 0.13 mmol, 0.2 equiv), with the reaction mixture concentration set to 541 mM. After performing silica gel column chromatography with 100% hexane as the eluent, product **7b** was obtained as a white powder (130 mg, 74% yield).

mp: 86-89 °C

^{1}H NMR (800 MHz, $CDCl_3$) δ 7.53 – 7.50 (m, 1H), 7.42 – 7.39 (m, 1H), 7.37 – 7.33 (m, 2H), 7.32 – 7.27 (m, 2H), 7.22 – 7.17 (m, 3H), 6.52 (s, 1H), 2.53 – 2.48 (m, 2H), 2.16 (ddd, *J* = 14.1, 10.4, 3.8 Hz, 2H), 1.72 – 1.65 (m, 2H), 1.65 – 1.56 (m, 2H), 1.47 – 1.39 (m, 1H).

^{13}C{1H} NMR (201 MHz, $CDCl_3$) δ 163.4, 154.5, 146.5, 128.7, 128.2, 126.4, 126.1, 123.1, 122.3, 120.3, 111.0, 103.2, 44.8, 35.6, 25.9, 22.8.

GC/MS (m/z, relative intensity) 276 (M^+, 95), 247 (25), 233 (100), 220 (50), 207 (45), 131 (35), 115 (50)

[8b] ((4,6-dimethoxy-1,3-phenylene)bis(cyclohexane-1,1-diyl))dibenzene

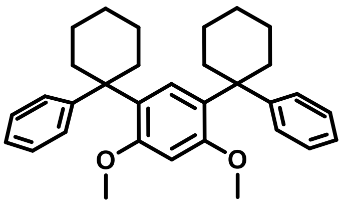

The reaction was set up following the procedure in Scheme S4, using **1b** cyclohexene (260 mg, 1.6 mmol, 3 equiv), identified as 2,3,4,5-tetrahydro-1,1'-biphenyl, along with 1,3-dimethoxybenzene (75 mg, 0.543 mmol, 1 equiv), Ir(dFppy)$_3$ (1 mg, 1.4 µmol, 0.0025 equiv), and collidine•HPF_6 (20 mg, 54 µmol, 0.1 equiv). With a reaction mixture concentration of 543 mM, product **8b** was obtained as a colorless crystalline solid (242 mg, 98% yield). Purification was performed using silica gel column chromatography with 100% hexane as the eluent.

mp: 89-89 °C

^{1}H NMR (800 MHz, $CDCl_3$) δ 7.53 (s, 1H), 7.31 – 7.28 (m, 4H), 7.26 – 7.23 (m, 4H), 7.11 (tt, *J* = 7.2, 1.3 Hz, 2H), 6.26 (s, 1H), 3.38 (s, 6H), 2.52 (ddd, *J* = 10.9, 7.5, 3.6 Hz, 4H), 2.23 (ddd, *J* = 13.2, 8.8, 3.6 Hz, 4H), 1.67 – 1.56 (m, 10H), 1.52 – 1.45 (m, 2H).

^{13}C{1H} NMR (201 MHz, $CDCl_3$) δ 157.4, 150.0, 128.8, 127.8, 127.5, 127.3, 124.8, 100.0, 55.6, 45.8, 36.7, 26.9, 23.6.

GC/MS (m/z, relative intensity) 454 (M^+, 80), 411 (15), 295 (20), 253(15), 159 (20), 117 (40), 103 (20), 91 (100)

[9b] N,N-dimethyl-4-(1-phenylcyclohexyl)aniline

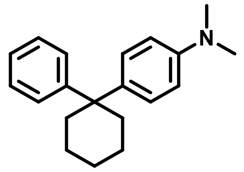

The reaction was conducted according to the procedure in Scheme S4, using **1b** cyclohexene (565 mg, 3.6 mmol, 1 equiv), identified as 2,3,4,5-tetrahydro-1,1'-biphenyl, together with N,N-dimethylaniline (433 mg, 3.6 mmol, 1 equiv), Ir(dFppy)$_3$ (3 mg, 3.6 µmol, 0.0025 equiv), and collidine•HPF_6 (190 mg, 0.7 mmol, 0.2 equiv), with the reaction mixture concentration set to 1 M. The product, **9b**, was obtained as a white solid (990 mg, 99% yield) and purified by flash chromatography on silica gel using 5% EtOAc in hexane as the eluent.

mp: 96 to 97 °C

^{1}H NMR (800 MHz, $CDCl_3$) δ 7.33 – 7.30 (m, 2H), 7.28 (dd, *J* = 8.61, 6.98 Hz, 2H), 7.20 – 7.17 (m, 2H), 7.14 (tt, *J* = 7.15, 1.35 Hz, 1H), 6.73 (d, *J* = 8.42 Hz, 2H), 2.93 (s, 6H), 2.28 (t, *J* = 5.96 Hz, 4H), 1.64 – 1.55 (m, 5H), 1.54 – 1.49 (m, 2H).

^{13}C{1H} NMR (201 MHz, $CDCl_3$) δ 149.4, 148.1, 137.1, 128.2, 128.0, 127.2, 125.3, 112.9, 45.5, 40.9, 37.3, 26.6, 23.1.

GC/MS (m/z, relative intensity) 279 (M^+, 100), 280 (60), 202(40), 159(40)

The reaction was conducted according to the procedure in Scheme S4, using **1b** cyclohexene (100 mg, 0.633 mmol, 1 equiv), identified as 2,3,4,5-tetrahydro-1,1'-biphenyl, along with aniline (177 mg, 1.9 mmol, 3 equiv), Ir(dFppy)$_3$ (1 mg, 1.6 µmol, 0.0025 equiv), and collidine•HPF_6 (34 mg, 127 µmol, 0.2 equiv), with the reaction mixture concentration set to 630 mM. Compound **10b'** was obtained as pale-yellow crystals (143 mg, 90% yield), and **10b** as a pale-yellow oil (11 mg, 7% yield). The purification involved flash chromatography on silica gel, using 5% EtOAc in hexane as the eluent to separate **10b'** and **10b**.

[10b'] N-(1-phenylcyclohexyl)aniline

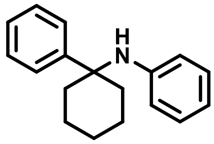

mp: 100-101 °C

^{1}H NMR (800 MHz, $CDCl_3$) δ 7.56 (d, *J* = 7.8 Hz, 2H), 7.35 (t, *J* = 7.6 Hz, 2H), 7.24 (t, *J* = 7.6 Hz, 1H), 7.01 (t, *J* = 7.7 Hz, 2H), 6.62 (t, *J* = 7.4 Hz, 1H), 6.37 (d, *J* = 8.0 Hz, 2H), 4.20 (s, 1H), 2.19 (d, *J* = 13.6 Hz, 2H), 1.83 (td, *J* = 13.4, 4.0 Hz, 2H), 1.76 (dt, *J* = 13.3, 3.8 Hz, 1H), 1.66 (dt, *J* = 13.9, 3.7 Hz, 2H), 1.60 (ddt, *J* = 16.9, 13.4, 6.7 Hz, 2H), 1.33 (qt, *J* = 12.8, 4.3 Hz, 1H).

^{13}C{1H} NMR (201 MHz, $CDCl_3$) δ 148.6, 145.6, 128.8, 128.7, 126.4, 125.9, 117.1, 115.5, 57.6, 36.3, 25.6, 22.1.

GC/MS (m/z, relative intensity) 252 ($M+1^+$, 45), 251 (M^+, 30), 159 (30), 122 (20), 94 (100)

[10b] 4-(1-phenylcyclohexyl)aniline

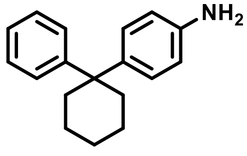

^{1}H NMR (800 MHz, $CDCl_3$) δ 7.29 – 7.22 (m, 4H), 7.11 (tt, *J* = 6.9, 1.7 Hz, 1H), 7.07 – 7.01 (m, 2H), 6.63 – 6.58 (m, 2H), 3.58 (br s, 2H), 2.23 (t, *J* = 5.8 Hz, 4H), 1.57 – 1.52 (m, 4H), 1.51 – 1.43 (m, 2H).

^{13}C{1H} NMR (201 MHz, $CDCl_3$) δ 149.5, 143.8, 138.8, 128.2, 128.2, 127.2, 125.3, 115.2, 45.7, 37.3, 26.6, 23.1.

GCMS (m/z, relative intensity) 252 ($M+1^+$, 60), 251 (M^+, 40), 174 (100), 159 (70), 94 (15)

[11b] (1-(2-phenylallyl)cyclohexyl)benzene

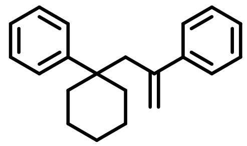

The reaction was conducted according to the procedure in Scheme S4, using **1b** cyclohexene (100 mg, 0.633 mmol, 1 equivalent), identified as 2,3,4,5-tetrahydro-1,1'-biphenyl. This was combined with prop-1-en-2-ylbenzene (82 mg, 0.7 mmol, 1.1 equivalents), a stock solution of Ir(dFppy)$_3$ in CH_2Cl_2 (1.6 µM, 1.5 mL, 0.0025 equivalents), and collidine•HPF_6 (34 mg, 0.13 mmol, 0.2 equivalents), resulting in a reaction mixture concentration of 253 mM. The product, 11b, was obtained as a colorless liquid (108 mg, 71% yield). Purification was performed with a silica plug using 100% hexane as the eluent.

^{1}H NMR (800 MHz, $CDCl_3$) δ 7.32 – 7.28 (m, 4H), 7.29 – 7.24 (m, 4H), 7.25 – 7.22 (m, 1H), 7.18 – 7.13 (m, 1H), 5.13 (d, *J* = 2.0 Hz, 1H), 4.65 (d, *J* = 2.1 Hz, 1H), 2.81 (s, 2H), 2.21 – 2.11 (m, 2H), 1.63 – 1.49 (m, 5H), 1.42 – 1.31 (m, 4H).

^{13}C{1H} NMR (201 MHz, $CDCl_3$) δ 146.1, 145.7, 143.6, 128.0, 127.9, 127.4, 126.7, 126.5, 125.3, 117.1, 50.7, 43.0, 36.4, 26.6, 22.6.

GCMS (m/z, relative intensity) 276 (M^+, ~1), 159 (60), 115 (10), 91 (100), 81 (30)

[12b] 4-(1-phenylcyclohexyl)phenol

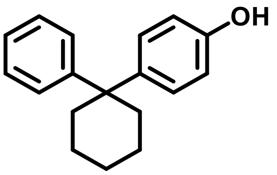

The reaction was set up following the procedure in Scheme S4, using **1b** cyclohexene (100 mg, 0.633 mmol), identified as 2,3,4,5-tetrahydro-1,1'-biphenyl, along with phenol (179 mg, 1.9 mmol, 3 equiv), a stock solution of Ir(dFppy)$_3$ in DCE (1.32 µM, 1.2 mL, 0.0025 equiv), and collidine•HPF_6 (34 mg, 0.13 mmol, 0.2 equiv), with the reaction mixture concentration adjusted to 541 mM using DCE instead of DCM. After adding all reagents, the NMR tube was placed in a blue light bath at 60 °C. Product **12b** was obtained as a white powder (131 mg, 83% yield) and was purified by filtration through a silica gel plug using hexane.

mp: 123-124 °C

^{1}H NMR (800 MHz, $CDCl_3$) δ 7.32 – 7.27 (m, 4H), 7.19 – 7.14 (m, 3H), 6.78 – 6.73 (m, 2H), 4.73 (s, 1H), 2.32 – 2.24 (m, 4H), 1.62 – 1.57 (m, 4H), 1.56 – 1.49 (m, 2H).

^{13}C{1H} NMR (201 MHz, $CDCl_3$) δ 153.3, 149.2, 141.2, 128.7, 128.5, 127.4, 125.6, 115.3, 45.9, 37.5, 26.7, 23.2.

GC/MS (m/z, relative intensity) 252 (M^+, 80), 209 (100), 183 (35), 131 (30), 115 (50), 107 (35), 91 (30)

[13b] 4-(1-phenylcyclohexyl)benzene-1,3-diol

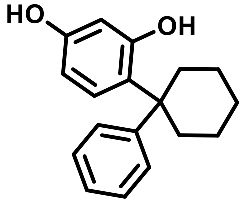

The reaction followed the procedure in Scheme S4, using **1b** cyclohexene (24 mg, 0.3 mmol, 1 equivalent), identified as 2,3,4,5-tetrahydro-1,1'-biphenyl, together with resorcinol (100 mg, 0.9 mmol, 3 equivalents), a stock solution of Ir(dFppy)$_3$ in CH_2Cl_2 (1.13 µM, 500 µL, 0.0025 equivalents), and collidine•HPF_6 (16 mg, 60 µmol, 0.2 equivalents) at a reaction mixture concentration of 30 mM. Due to resorcinol's low solubility in CH_2Cl_2, the mixture was diluted to 30 mM with respect to 1b, resulting in a reaction volume of over 10 mL, which exceeded the capacity of an NMR tube. A test tube was used instead, with TLC monitoring to observe the disappearance of the high Rf spot for 1b. Purification was achieved by flash chromatography using 20% EtOAc in hexane. The product, **13b**, was obtained as a white powder (73 mg, 91% yield).

mp: 75-76 °C

^{1}H NMR (800 MHz, $CDCl_3$) δ 7.39 – 7.35 (m, 3H), 7.33 – 7.30 (m, 2H), 7.20 (tt, *J* = 7.2, 1.3 Hz, 1H), 6.44 (dd, *J* = 8.6, 2.7 Hz, 1H), 6.19 (d, *J* = 2.7 Hz, 1H), 5.11 (s, 1H), 4.69 (s, 1H), 2.35 – 2.30 (m, 2H), 2.18 (m, 2H), 1.64 – 1.52 (m, 5H), 1.51 – 1.44 (m, 1H).

^{13}C{1H} NMR (201 MHz, $CDCl_3$) δ 155.2, 155.2, 148.4, 129.1, 129.0, 127.2, 127.0, 126.5, 107.3, 105.4, 44.9, 36.6, 26.5, 23.2.

GC/MS (m/z, relative intensity) 268 (M^+, 100), 225 (95), 211 (40), 199 (35), 198 (30), 147 (25), 123 (40), 115 (50), 91 (45)

[2i/2i'] 2-methyl-5-(4-methyl-1-phenylcyclohexyl)furan

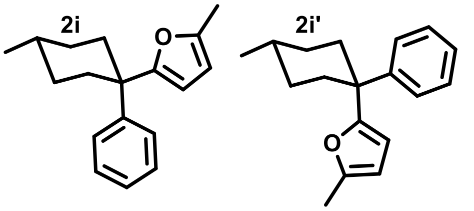

The reaction was performed according to the procedure in Scheme S4, using **1i** cyclohexene (100 mg, 0.58 mmol, 1 equivalent), identified as 4-methyl-2,3,4,5-tetrahydro-1,1'-biphenyl, along with 2-methylfuran (147 mg, 1.74 mmol, 3 equivalents), $Ir(dFppy)_3$ (1 mg, 1.5 µmol, 0.0025 equivalents), and collidine•HPF_6 (31 mg, 116 µmol, 0.2 equivalents), at a reaction mixture concentration of 580 mM. The products, **2i**/**2i'**, were obtained as a mixture of diastereomers in the form of yellow crystals (115 mg, 78% yield, dr = 77:23).

mp: 40-42 °C

^{1}H NMR (800 MHz, $CDCl_3$) δ 7.4 – 7.4 (m, 7H), 7.3 (dd, *J* = 8.37, 7.23 Hz, 7H), 7.3 – 7.2 (m, 2H), 7.2 (tt, *J* = 7.63, 1.18 Hz, 6H), 7.1 (td, *J* = 7.12, 1.22 Hz, 1H), 6.1 (d, *J* = 3.01 Hz, 1H), 5.9 (dq, *J* = 3.02, 1.04 Hz, 1H), 5.8 (dt, *J* = 3.07, 1.19 Hz, 3H), 5.6 (d, *J* = 3.08 Hz, 3H), 2.6 – 2.5 (m, 7H), 2.5 (ddt, *J* = 13.86, 4.30, 2.82 Hz, 3H), 2.2 (d, *J* = 1.10 Hz, 3H), 2.2 (d, *J* = 1.12 Hz, 11H), 2.0 (ddd, *J* = 13.81, 12.38, 3.46 Hz, 8H), 1.9 (td, *J* = 13.42, 3.68 Hz, 3H), 1.6 (dddd, *J* = 18.25, 13.29, 6.05, 2.52 Hz, 9H), 1.6 – 1.5 (m, 3H), 1.2 (tdd, *J* = 13.36, 11.58, 3.15 Hz, 2H), 1.1 (tdd, *J* = 13.26, 10.80, 3.08 Hz, 8H), 0.9 (d, *J* = 6.48 Hz, 4H), 0.8 (d, *J* = 6.57 Hz, 11H).

Composite ^{13}C{1H} NMR (201 MHz, $CDCl_3$) δ 161.7, 157.0, 150.5, 150.4, 149.6, 143.8, 128.3, 128.2, 127.7, 126.1, 125.9, 125.9, 107.9, 105.9, 105.6, 104.4, 44.4, 43.3, 36.1, 34.3, 32.5, 32.3, 32.0, 30.8, 22.5, 22.0, 13.8, 13.7.

GC/MS (m/z, relative intensity) 254 (M^+, 50), 197 (100), 184 (25), 115 (25)

[2j/2j'] 2-methyl-5-(4-*tert*-butyl-1-phenylcyclohexyl)furan

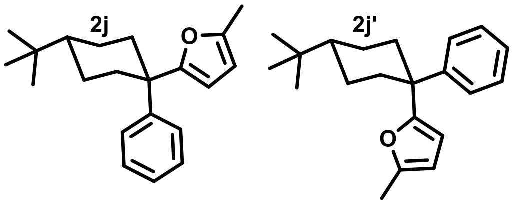

The reaction was conducted following the procedure in Scheme S4, beginning with **1j** cyclohexene (75 mg, 0.35 mmol), identified as 4-(tert-butyl)-2,3,4,5-tetrahydro-1,1'-biphenyl, along with 2-methylfuran (86 mg, 1.05 mmol, 3 equiv), a stock solution of $Ir(dFppy)_3$ in CH_2Cl_2 (0.56 mM, 1.2 mL, 0.0025 equiv), and collidine•HPF_6 (19 mg, 0.07 mmol, 0.2 equiv). With a reaction mixture concentration of 300 mM, products **2j**/**2j'** were obtained as a colorless diastereomeric oil mixture (97 mg, 93% yield, dr = 83:17). Purification was performed using flash chromatography with hexane instead of simple elution through a silica gel plug. Refer to Figure S6 for NOE experimental details.

^{1}H NMR (800 MHz, $CDCl_3$) δ 7.51 – 7.48 (m, 1H), 7.36 (dd, *J* = 8.4, 7.3 Hz, 1H), 7.31 – 7.27 (m, 0H), 7.24 – 7.21 (m, 1H), 6.14 (d, *J* = 3.1 Hz, 0H), 5.96 (dd, *J* = 3.0, 1.3 Hz, 0H), 5.78 (dd, *J* = 3.1, 1.3 Hz, 0H), 5.58 (d, *J* = 3.2 Hz, 0H), 2.77 – 2.71 (m, 1H), 2.58 (ddt, *J* = 13.9, 4.5, 2.9 Hz, 0H), 2.27 (d, *J* = 1.1 Hz, 0H), 2.22 (d, *J* = 1.5 Hz, 1H), 2.00 (td, *J* = 13.6, 3.3 Hz, 1H), 1.87 (td, *J* = 13.3, 3.6 Hz, 0H), 1.79 – 1.74 (m, 0H), 1.74 – 1.69 (m, 1H), 1.31 (qd, *J* = 12.7, 3.1 Hz, 0H), 1.25 – 1.13 (m, 2H), 0.87 (s, 1H), 0.81 (s, 5H).

^{13}C{1H} NMR (201 MHz, $CDCl_3$) δ 162.4, 156.9, 150.4, 150.3, 149.7, 142.9, 128.3, 128.2 – 128.1 (m), 127.9, 126.1, 125.9, 125.8, 107.9, 105.9, 105.6, 103.9, 48.4, 48.1, 44.6, 43.2, 36.7, 35.4, 32.5, 32.5, 27.7, 27.6, 24.3, 23.3, 13.8, 13.7.

GC/MS (m/z, relative intensity) 296 (M^+, 40), 197 (100)

[2k] 2-((1s,3R,5S)-3,5-dimethyl-1-phenylcyclohexyl)-5-methylfuran

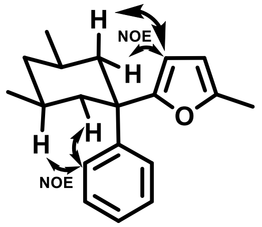

The reaction was carried out as per the procedure in Scheme S4, using **1k** cyclohexene (186 mg, 1 mmol, 1 equivalent), identified as cis–3,5-dimethyl-2,3,4,5-tetrahydro-1,1'-biphenyl, along with 2-methylfuran (246 mg, 3 mmol, 3 equivalents), Ir(dFppy)$_3$ (2 mg, 2.5 μmol, 0.0025 equivalents), and collidine•HPF_6 (50 mg, 200 μmol, 0.2 equivalents), with the reaction mixture concentration set to 1 M. The product, **2k**, was obtained as a colorless solid (213 mg, 79% yield). Purification was achieved by flash chromatography on silica gel using hexane instead of simple elution. The diastereomer was confirmed through NOE experiments, selectively enhancing the resonances at 7.43 ppm (ortho-phenyl C–H), 5.55 ppm (furyl), and 1.55 ppm (methine). See S163.

^{1}H NMR (800 MHz, $CDCl_3$) δ 7.47 – 7.43 (m, 2H), 7.36 – 7.32 (m, 2H), 7.21 (ddt, *J* = 8.5, 7.2, 1.2 Hz, 1H), 5.75 (dd, *J* = 3.0, 1.3 Hz, 1H), 5.55 (d, *J* = 3.1 Hz, 1H), 2.61 – 2.58 (m, 2H), 2.21 (d, *J* = 1.4 Hz, 3H), 1.63 (ddq, *J* = 14.2, 3.5, 1.9 Hz, 1H), 1.61 – 1.54 (m, 4H), 0.95 (d, *J* = 6.4 Hz, 6H), 0.70 (q, *J* = 11.5 Hz, 1H).

^{13}C{1H} NMR (201 MHz, $CDCl_3$) δ 162.2, 150.3, 143.7, 128.3, 127.7, 125.9, 105.6, 103.9, 44.6, 44.2, 43.2, 28.1, 22.8, 13.7.

GC/MS (m/z, relative intensity) 268 (M^+, 100), 253 (20), 211 (25), 191 (60), 187 (25) **2k':** 268 (M^+, 90), 211 (25), 191 (100), 187 (25), 105 (20)

[2l] 4-(4-methoxyphenyl)-4-(5-methylfuran-2-yl)cyclohexan-1-one

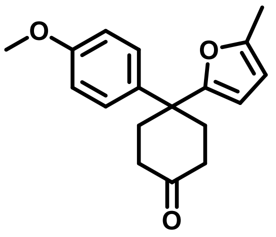

The reaction was conducted according to the procedure in Scheme S4, using **1l** cyclohexene (53 mg, 0.26 mmol, 1 equiv), identified as 4'-methoxy-2,5-dihydro-[1,1'-biphenyl]-4(3H)-one, along with 2-methylfuran (65 mg, 0.79 mmol, 3 equiv), a stock solution of Ir(dFppy)$_3$ in CH_2Cl_2 (0.33 μM, 2 mL, 0.0025 equiv), and collidine•HPF_6 (13 mg, 0.05 mmol, 0.2 equiv), with the reaction mixture concentration set to 50 mM. After completion, the reaction mixture was purified using a silica gel column with a gradient of 30% EtOAc to 70% hexane, yielding **2l** as yellow crystals (30 mg, 42% yield).

mp: 109-111 °C

^{1}H NMR (800 MHz, $CDCl_3$) δ 7.22 – 7.18 (m, 2H), 6.87 – 6.83 (m, 2H), 6.03 (d, *J* = 3.1 Hz, 1H), 5.92 (dq, *J* = 3.1, 1.0 Hz, 1H), 3.79 (s, 3H), 2.65 – 2.60 (m, 2H), 2.51 – 2.42 (m, 4H), 2.41 – 2.36 (m, 2H), 2.25 (d, *J* = 1.1 Hz, 3H).

^{13}C{1H} NMR (201 MHz, $CDCl_3$) δ 211.2, 158.1, 156.3, 151.2, 136.9, 127.3, 113.8, 107.3, 106.1, 55.2, 42.9, 38.5, 35.6, 13.6.

GC/MS (m/z, relative intensity) 284 (M^+, 40), 227 (100), 214 (25)

[2m] 3-(4-methoxyphenyl)-3-(5-methylfuran-2-yl)cyclohexan-1-one

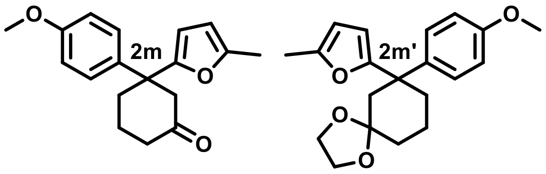

The reaction was conducted acc(ording to the procedure in Scheme S4, using **1m** cyclohexene (100 mg, 0.41 mmol, 1 equiv), identified as 7-(4-methoxyphenyl)-1,4-dioxaspiro[4.5]dec-6-ene, along with 2-methylfuran (100 mg, 1.22 mmol, 3 equiv), a stock solution of Ir(dFppy)$_3$ in CH_2Cl_2 (1.1 mM, 0.95 mL, 0.0025 equiv), and collidine•HPF_6 (22 mg, 0.08 mmol, 0.2 equiv), with the reaction mixture concentration set to 200 mM. Compounds **2m** and **2m'** were formed and separated by basic alumina column chromatography (10% EtOAc in hexane), yielding 50 mg of **2m** and 70 mg of **2m'** (combined yield: 96%). The **2m'** was then converted to **2m** by treatment with TFA (25 mL) at room temperature for 30 minutes, followed by flash column chromatography and combination with the **2m** fraction from the column. This resulted in a 90 mg yield (78%) of yellow liquid.

2m:

^{1}H NMR (800 MHz, $CDCl_3$) δ 7.19 – 7.14 (m, 2H), 6.86 – 6.82 (m, 2H), 5.97 (d, *J* = 3.1 Hz, 1H), 5.84 (dd, *J* = 3.1, 1.2 Hz, 1H), 3.78 (s, 3H), 2.93 (dt, *J* = 14.9, 1.8 Hz, 1H), 2.77 (dd, *J* = 15.0, 1.3 Hz, 1H), 2.59 – 2.54 (m, 1H), 2.38 – 2.27 (m, 3H), 2.21 (d, *J* = 1.1 Hz, 3H), 1.87 – 1.80 (m, 1H), 1.63 – 1.57 (m, 1H).

^{13}C{1H} NMR (201 MHz, $CDCl_3$) δ 210.1, 158.3, 156.1, 151.6, 137.3, 127.4, 113.9, 108.6, 106.0, 55.4, 52.1, 47.4, 40.8, 34.4, 21.8, 13.7.

GC/MS (m/z, relative intensity) 284 (M^+, 91), 241 (100), 227 (82), 214 (50), 199 (33), 128 (30)

[2n] 4-(5-methylfuran-2-yl)-4-phenyl-1-tosylpiperidine

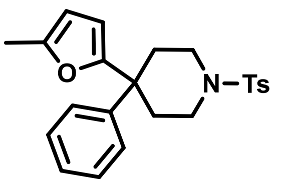

The reaction was conducted following the procedure outlined in Scheme S4, utilizing **1n** piperidine alkene (100 mg, 0.32 mmol, 1 equiv), identified as 4-phenyl-1-tosyl-1,2,3,6-tetrahydropyridine, in combination with 2-methylfuran (79 mg, 0.96 mmol, 3 equiv), a stock solution of Ir(dFppy)$_3$ in CH_2Cl_2 (0.57 µM, 1.4 mL, 0.0025 equiv), and collidine•HPF_6 (17 mg, 0.064 mmol, 0.2 equiv), with the concentration of the reaction mixture precisely adjusted to 228 mM. Product **2n** was isolated as a brown solid (54 mg, 43% yield). Purification was carried out via flash chromatography using a gradient of 10% EtOAc in hexane, as opposed to simple elution through a silica gel plug.

mp: 107-113 ºC

^{1}H NMR (800 MHz, $CDCl_3$) δ 7.63 – 7.61 (m, 2H), 7.28 (d, *J* = 7.9 Hz, 2H), 7.27 – 7.24 (m, 4H), 7.18 – 7.15 (m, 1H), 7.12 – 7.09 (m, 2H), 5.86 (d, *J* = 3.1 Hz, 1H), 5.82 (dd, *J* = 3.0, 1.3 Hz, 1H), 3.57 (dt, *J* = 12.3, 4.2 Hz, 2H), 2.75 (ddd, *J* = 11.7, 11.6, 2.6 Hz, 2H), 2.45 – 2.40 (m, 5H), 2.27 (ddd, *J* = 14.3, 10.9, 4.0 Hz, 2H), 2.17 (s, 3H).

^{13}C{1H} NMR (201 MHz, $CDCl_3$) δ 155.2, 151.2, 146.2, 143.4, 134.3, 129.7, 128.5, 127.6, 126.6, 126.2, 108.0, 106.1, 43.4, 42.7, 34.6, 21.6, 13.7.

GC/MS (m/z, relative intensity) 395 (M^+, 5), 312(20), 240(35), 158(75), 155(32), 131(85), 91(100)

[2o] 1-(4-(5-methylfuran-2-yl)-4-phenylpiperidin-1-yl)ethan-1-one

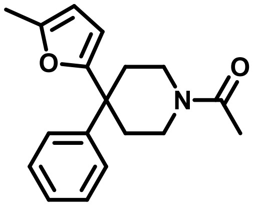

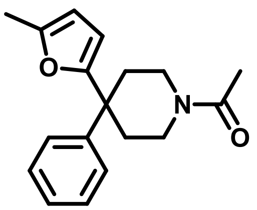

The reaction was set up according to the procedure in Scheme S4, using **1o** piperidine alkene (106 mg, 0.53 mmol, 1 equiv), identified as 1-(4-phenyl-3,6-dihydropyridin-1(2H)-yl)ethan-1-one, along with 2-methylfuran (130 mg, 1.6 mmol, 3 equiv), a stock solution of Ir(dFppy)$_3$ in CH_2Cl_2 (0.56 µM, 2.4 mL, 0.0025 equiv), and collidine•HPF_6 (28 mg, 0.1 mmol, 0.2 equiv), with the reaction mixture concentration set to 220 mM. Product **2o** was obtained as a yellow liquid (68 mg, 46% yield). Purification was performed by flash chromatography using 20% EtOAc in hexane, instead of simple elution through a silica gel plug. Limited rotation around the amide bond appeared to create an inseparable mixture of rotamers.

Composite ^{1}H NMR (800 MHz, $CDCl_3$) δ 7.41 – 7.32 (m, 1H), 7.29 (t, *J* = 7.70 Hz, 2H), 7.21 – 7.17 (m, 2H), 6.10 – 6.07 (m, 0H), 6.01 (d, *J* = 3.12 Hz, 1H), 5.92 (dd, *J* = 2.97, 1.28 Hz, 1H), 4.29 – 4.21 (m, 1H), 4.19 – 4.11 (m, 0H), 3.82 (t, *J* = 5.75 Hz, 0H), 3.69 – 3.62 (m, 1H), 3.33 (t, *J* = 12.88 Hz, 1H), 3.09 (t, 1H), 2.62 – 2.58 (m, 0H), 2.57 – 2.53 (m, 0H), 2.41 (dd, *J* = 45.39, 13.76 Hz, 2H), 2.24 (s, 2H), 2.10 (s, 2H), 1.25 (s, 2H), 1.11 (s, 1H).

Composite ^{13}C{1H} NMR (201 MHz, $CDCl_3$) δ 169.4, 169.0, 155.7, 151.3, 146.2, 140.4, 137.0, 135.0, 128.6, 128.6, 127.5, 126.6, 126.2, 125.0, 121.2, 119.5, 107.9, 106.2, 59.7, 46.0, 43.9, 43.3, 38.9, 38.3, 35.7, 34.6 (d, *J* = 64.7 Hz), 31.4, 29.8, 22.8, 21.6, 14.3, 13.7.

GC/MS (m/z, relative intensity) 283(M^+, 45), 201(100), 171(35), 159(72), 115(30), 91(25)

[2s] *tert*-butyl 4-(5-methylfuran-2-yl)-4-phenylpiperidine-1-carboxylate

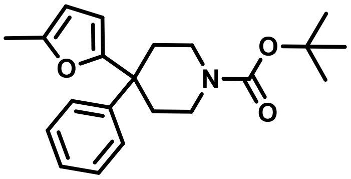

The reaction was performed according to the procedure in Scheme S4, using **1s** piperidine alkene (75 mg, 0.29 mmol, 1 equiv), identified as tert-butyl 4-phenyl-3,6-dihydropyridine-1(2H)-carboxylate, along with 2-methylfuran (71 mg, 0.87 mmol, 3 equiv), a stock solution of Ir(dFppy)$_3$ in CH_2Cl_2 (1.36 µM, 0.5 mL, 0.0025 equiv), and collidine•HPF_6 (15 mg, 0.058 mmol, 0.2 equiv), with the reaction mixture concentration adjusted to exactly 290 mM. The product, **2s**, was obtained as a beige oil (62 mg, 63% yield). Purification was carried out by flash chromatography using a gradient of 15% EtOAc to 85% hexane, instead of simple silica gel plug elution.

^{1}H NMR (800 MHz, $CDCl_3$) δ 7.30 – 7.27 (m, 2H), 7.22 – 7.20 (m, 2H), 7.20 – 7.17 (m, 1H), 6.00 (d, *J* = 3.2 Hz, 1H), 5.92 (dq, *J* = 3.1, 1.0 Hz, 1H), 3.85 (d, *J* = 12.9 Hz, 2H), 3.12 (ddd, *J* = 13.8, 10.8, 2.8 Hz, 2H), 2.39 – 2.34 (m, 2H), 2.23 (d, *J* = 1.3 Hz, 3H), 2.12 (ddd, *J* = 14.4, 10.8, 4.2 Hz, 2H), 1.47 (s, 9H).

^{13}C{1H} NMR (201 MHz, $CDCl_3$) δ 156.0, 155.1, 151.1, 146.7, 128.5, 126.4, 126.3, 107.7, 106.1, 79.5, 43.1, 41.0, 34.9, 28.6, 13.7.

GC/MS (m/z, relative intensity) 341 (M^+, 10), 285 (35), 268 (30), 258 (30), 240 (25), 203 (100), 196 (35), 159 (70), 142 (35), 131 (35), 91 (30)

[8s] *tert*-butyl 4-(2,4-dimethoxyphenyl)-4-phenylpiperidine-1-carboxylate

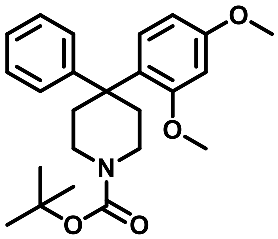

The reaction was conducted according to the procedure outlined in Scheme S4, using **1s** piperidine alkene (100 mg, 0.39 mmol, 1 equiv), specifically tert-butyl 4-phenyl-3,6-dihydropyridine-1(2H)-carboxylate, along with dimethoxybenzene (183 mg, 1.2 mmol, 3 equiv), a stock solution of Ir(dFppy)$_3$ in CH_2Cl_2 (1.04 µM, 0.92 mL, 0.0025 equiv), and collidine•HPF_6 (21 mg, 0.08 mmol, 0.2 equiv). The reaction mixture concentration was adjusted to precisely 200 mM. Compound **8s** was obtained as a white powder (90 mg, 59% yield) and purified by flash chromatography on silica gel, eluting with 10% EtOAc in hexane

mp: 133-135 °C

^{1}H NMR (800 MHz, $CDCl_3$) δ 7.28 (d, *J* = 8.6 Hz, 1H), 7.23 – 7.20 (m, 4H), 7.12 – 7.08 (m, 1H), 6.49 (dd, *J* = 8.6, 2.6 Hz, 1H), 6.36 (d, *J* = 2.6 Hz, 1H), 3.78 (s, 3H), 3.59 – 3.50 (m, 2H), 3.42 (s, 3H), 3.40 (ddd, *J* = 12.9, 8.6, 3.2 Hz, 2H), 2.56 – 2.44 (m, 2H), 2.38 – 2.28 (m, 2H), 1.45 (s, 9H).

^{13}C{1H} NMR (201 MHz, $CDCl_3$) δ 159.6, 159.3, 155.2, 147.6, 128.5, 127.7, 127.4, 127.2, 125.4, 103.8, 100.8, 79.3, 55.4, 55.3, 43.8, 41.1 (d, *J* = 204.2 Hz), 35.5, 28.6.

GC/MS (m/z, relative intensity) 297(M-($CO_2C(CH_3)_3^+$, 17), 159(100), 158(25), 130(15), 91(17), 82(15)

[16s] *tert*-butyl 4-(1H-indol-3-yl)-4-phenylpiperidine-1-carboxylate

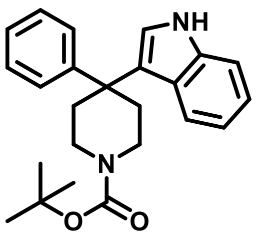

The reaction was carried out following the procedure outlined in Scheme S4, using **1s** piperidine alkene (100 mg, 0.39 mmol, 1 equiv), specifically tert-butyl 4-phenyl-3,6-dihydropyridine-1(2H)-carboxylate, along with 1H-indole (135 mg, 1.2 mmol, 3 equiv), a stock solution of Ir(dFppy)$_3$ in CH_2Cl_2 (1.04 µM, 0.92 mL, 0.0025 equiv), and collidine•HPF_6 (21 mg, 0.08 mmol, 0.2 equiv). The concentration of the reaction mixture was precisely adjusted to 132 mM. Compound **16s** was obtained as a yellow solid (108 mg, 74% yield) and purified by flash chromatography on silica gel, using 20% EtOAc in hexane as the eluent.

mp: 61-65 °C

^{1}H NMR (800 MHz, $CDCl_3$) δ 8.25 (s, 1H), 7.39 – 7.37 (m, 2H), 7.37 – 7.34 (m, 2H), 7.31 – 7.27 (m, 2H), 7.20 – 7.16 (m, 1H), 7.16 – 7.12 (m, 1H), 7.07 (d, *J* = 2.6 Hz, 1H), 6.98 – 6.94 (m, 1H), 3.77 – 3.64 (m, 2H), 3.51 – 3.38 (m, 2H), 2.57 – 2.52 (m, 2H), 2.44 – 2.37 (m, 2H), 1.50 (s, 9H).

^{13}C{1H} NMR (201 MHz, $CDCl_3$) δ 155.1, 147.3, 137.1, 128.3, 126.9, 125.9, 125.8, 122.3, 121.8, 121.4, 121.0, 119.1, 111.3, 79.4, 41.7, 41.0 (d, *J* = 203.0 Hz), 36.2, 28.5.

GC/MS (m/z, relative intensity) 276(M-($CO_2C(CH_3)_3^+$, 52), 232 (56), 220 (82), 217 (36), 159 (100), 144 (36), 130 (80), 115 (75), 103 (75), 91 (35), 82 (35)

[11s] *tert*-butyl 4-phenyl-4-(2-phenylallyl)piperidine-1-carboxylate

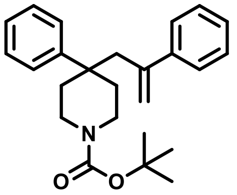

The reaction was conducted following the procedure outlined in Scheme S4. It involved **1s** piperidine alkene (142 mg, 0.54 mmol, 1 equivalent), specified as tert-butyl 4-phenyl-3,6-dihydropyridine-1(2H)-carboxylate, combined with prop-1-en-2-ylbenzene (128 mg, 1.1 mmol, 2 equivalents). Additionally, a stock solution of Ir(dFppy)$_3$ in CH_2Cl_2 (1.36 µM, 1 mL, 0.0025 equivalents) and collidine•HPF_6 (29 mg, 0.17 mmol, 0.2 equivalents) were added, maintaining a reaction mixture concentration of 181 mM. The product, **11s**, was obtained as a colorless liquid (130 mg, 63% yield). Purification was achieved using flash chromatography on silica gel with 10% EtOAc in hexane as the eluent.

^{1}H NMR (800 MHz, $CDCl_3$) δ 7.26 (s, 1H), 7.21 (t, *J* = 7.6 Hz, 2H), 7.19 – 7.15 (m, 2H), 7.15 – 7.11 (m, 4H), 7.10 (t, *J* = 7.3 Hz, 1H), 5.04 (s, 1H), 4.56 (s, 1H), 3.80 – 3.56 (m, 1H), 2.88 (t, *J* = 12.5 Hz, 2H), 2.76 (s, 2H), 2.13 – 2.00 (m, 2H), 1.69 – 1.60 (m, 2H), 1.40 (s, 10H).

^{13}C{1H} NMR (201 MHz, $CDCl_3$) δ 155.1, 145.4, 143.5, 143.1, 128.3, 128.2, 127.3, 127.0, 126.5, 125.9, 117.6, 79.3, 49.8, 41.6, 40.5 (d, *J* = 182.2 Hz), 35.4, 28.6.

GC/MS (m/z, relative intensity) 278((M-($CO_2C(CH_3)_3$ +H) $^+$,0.17), 244(5), 232(25), 204(100), 186(4), 160(10)

[2t] tert-butyl 4-(4-methoxyphenyl)-4-(5-methylfuran-2-yl)piperidine-1-carboxylate

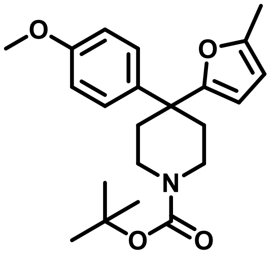

The reaction was conducted according to the procedure in Scheme S4, using **1t** piperidine alkene (100 mg, 0.35 mmol, 1 equiv), specifically tert-butyl 4-(4-methoxyphenyl)-3,6-dihydropyridine-1(2H)-carboxylate, along with 2-methylfuran (85 mg, 1 mmol, 3 equiv), a stock solution of Ir(dFppy)$_3$ in CH_2Cl_2 (1.1 μM, 0.81 mL, 0.0025 equiv), and collidine•HPF_6 (18 mg, 0.069 mmol, 0.2 equiv). The reaction mixture concentration was precisely adjusted to 200 mM. Compound **2t** was obtained as a pale-yellow oil (74 mg, 58% yield) and purified by flash chromatography using 10% EtOAc in hexane, rather than simple elution through a silica gel plug.

^{1}H NMR (800 MHz, $CDCl_3$) δ 7.14 – 7.10 (m, 2H), 6.84 – 6.80 (m, 2H), 5.96 (d, *J* = 3.1 Hz, 1H), 5.91 – 5.88 (m, 1H), 3.77 (s, 5H), 3.24 – 3.00 (m, 2H), 2.32 (d, *J* = 13.8 Hz, 2H), 2.23 (s, 3H), 2.14 – 2.00 (m, 2H), 1.45 (s, 9H).

^{13}C{1H} NMR (201 MHz, $CDCl_3$) δ 158.0, 156.4, 155.1, 151.0, 138.8, 127.3, 113.8, 107.5, 106.1, 79.5, 55.3, 42.5, 41.0 (d, *J* = 205.9 Hz), 35.1, 28.6, 13.7.

GC/MS (m/z, relative intensity) 271(M-($CO_2C(CH_3)_3$$^+$, 82), 188(100), 158(42)

Characteristic Datasheets for Ether Products

[E12s] *tert*-butyl 4-phenoxy-4-phenylpiperidine-1-carboxylate

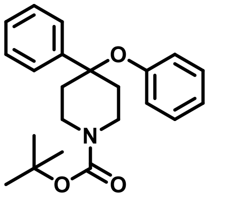

The reaction was conducted according to the procedure outlined in Scheme S4, using **1s** piperidine alkene (100 mg, 0.39 mmol, 1 equiv), identified as tert-butyl 4-phenyl-3,6-dihydropyridine-1(2H)-carboxylate, along with phenol (47 mg, 0.5 mmol, 1.3 equiv), a stock solution of Ir(dFppy)$_3$ in CH_2Cl_2 (1.04 µM, 0.92 mL, 0.0025 equivalents), and collidine•HPF_6 (21 mg, 0.08 mmol, 0.2 equiv). The reaction mixture concentration was precisely adjusted to 200 mM. The product, **E12s**, was obtained as a yellow liquid (121 mg, 89% yield) after purification by flash chromatography using 10% EtOAc in hexane.

^{1}H NMR (800 MHz, $CDCl_3$) δ 7.51 – 7.46 (m, 2H), 7.43 – 7.38 (m, 2H), 7.36 – 7.31 (m, 1H), 7.15 – 7.10 (m, 2H), 6.91 – 6.87 (m, 1H), 6.69 – 6.64 (m, 2H), 4.21 – 3.83 (m, 2H), 3.39 – 3.08 (m, 2H), 2.43 – 2.26 (m, 2H), 2.12 – 1.96 (m, 2H), 1.50 (s, 9H).

^{13}C{1H} NMR (201 MHz, $CDCl_3$) δ 155.2, 155.0, 145.4, 129.1, 128.9, 127.6, 125.3, 121.2, 119.1, 79.6, 78.7, 39.2, 35.9, 28.6.

GC/MS (m/z, relative intensity) 254 ((M-($CO_2C(CH_3)_3$ +H)$^+$, 5), 232 (10), 204 (100), 188 (5), 160 (60)

[E13s] *tert*-butyl 4-(3-hydroxyphenoxy)-4-phenylpiperidine-1-carboxylate

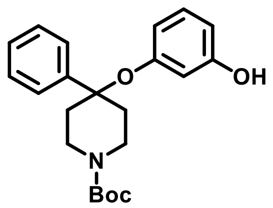

The reaction was carried out according to the procedure in Scheme S4, using **1s** piperidine alkene (100 mg, 0.39 mmol, 1 equiv), identified as tert-butyl 4-phenyl-3,6-dihydropyridine-1(2H)-carboxylate, along with resorcinol (127 mg, 1.2 mmol, 3 equiv), a stock solution of Ir(dFppy)$_3$ in CH_2Cl_2 (1.05 µM, 0.92 mL, 0.0025 equiv), and collidine•HPF_6 (21 mg, 0.08 mmol, 0.2 equiv), with the reaction mixture concentration adjusted to 20 mM. Due to the poor solubility of resorcinol in CH_2Cl_2, dilution with respect to **1s** was necessary, resulting in a reaction volume that exceeded the capacity of an NMR tube (20 mL). Therefore, the reaction was conducted in a test tube, with progress monitored by TLC (10% EtOAc in hexane) on aliquots, observing the disappearance of the higher Rf spot corresponding to **1s**. The product, **E13s**, was obtained as a colorless liquid (42 mg, 29% yield) following purification by flash chromatography using 30% EtOAc in hexane.

^{1}H NMR (800 MHz, CD_3CN) δ 7.47 – 7.45 (m, 2H), 7.41 – 7.38 (m, 2H), 7.34 – 7.30 (m, 1H), 6.90 (t, *J* = 8.2 Hz, 1H), 6.75 (s, 1H), 6.31 (ddd, *J* = 8.1, 2.3, 0.9 Hz, 1H), 6.16 (ddd, *J* = 8.3, 2.4, 0.9 Hz, 1H), 6.07 (t, *J* = 2.3 Hz, 1H), 3.93 (s, 2H), 3.31 – 3.01 (m, 2H), 2.26 (d, *J* = 13.9 Hz, 2H), 2.00 – 1.95 (m, 2H), 1.43 (s, 10H).

^{13}C{1H} NMR (201 MHz, $CDCl_3$) δ 162.7, 161.8, 153.6, 143.5, 132.1, 129.2, 128.2, 124.9, 124.0, 123.3, 117.0, 96.1, 85.3, 36.3, 25.2, 22.1.

GC/MS (m/z, relative intensity) 244 (5), 232 (20), 204 (75), 188 (7), 160(30), 139 (12), 111 (100)

[E15b] 4-((1-phenylcyclohexyl)oxy)-2H-chromen-2-one

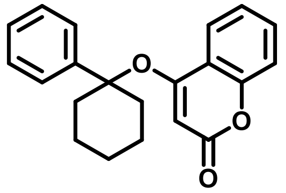

The reaction was carried out according to the procedure in Scheme S4, using **1b** cycloalkene (59 mg, 0.38 mmol, 1 equiv), identified as 2,3,4,5-tetrahydro-1,1'-biphenyl, along with 4-hydroxy-2H-chromen-2-one (92 mg, 0.57 mmol, 1.5 equiv), a stock solution of Ir(dFppy)$_3$ in CH_2Cl_2 (0.95 µM, 1 mL, 0.0025 equiv), and collidine•HPF_6 (20 mg, 0.08 mmol, 0.2 equivalents), with the reaction mixture concentration adjusted to 19 mM. Due to the poor solubility of 4-hydroxy-2H-chromen-2-one in CH_2Cl_2, additional dilution was necessary, resulting in a reaction volume that exceeded the capacity of an NMR tube (19 mL). Consequently, the reaction was conducted in a test tube, with progress monitored by TLC (10% EtOAc in hexane) on aliquots, observing the disappearance of the higher Rf spot corresponding to **1b**. The product, **E15b**,

was obtained as a white powder (82 mg, 69% yield) following purification by flash chromatography on silica gel using 10% EtOAc in hexane as eluent.

mp: 112-116°C

^{1}H NMR (800 MHz, $CDCl_3$) δ 8.05 (dd, *J* = 7.9, 1.6 Hz, 1H), 7.55 (ddd, *J* = 8.7, 7.4, 1.6 Hz, 1H), 7.38 – 7.34 (m, 4H), 7.33 (td, *J* = 7.6, 1.1 Hz, 1H), 7.31 – 7.27 (m, 2H), 5.06 (s, 1H), 2.61 – 2.56 (m, 2H), 1.95 – 1.87 (m, 2H), 1.83 – 1.78 (m, 1H), 1.78 – 1.72 (m, 4H), 1.42 – 1.33 (m, 1H).

^{13}C{1H} NMR (201 MHz, $CDCl_3$) δ 162.7, 161.8, 153.6, 143.5, 132.1, 129.2, 128.2, 124.9, 124.0, 123.3, 117.1, 117.0, 96.1, 85.3, 36.3, 25.2, 22.1.

GC/MS (m/z, relative intensity) 199 (2), 187 (10), 159 ((M-4-hydroxycoumarin)$^+$, 100), 158 (25), 157 (40), 119 (35), 105 (8)

[E15s] 2-oxo-2H-chromen-4-yl)oxy)-4-phenylpiperidine-1-carboxylate

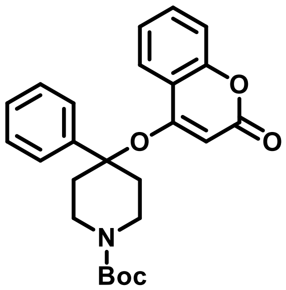

The reaction was performed according to the procedure in Scheme S4, using **1s** piperidine alkene (79 mg, 0.31 mmol, 1 equiv), identified as tert-butyl 4-phenyl-3,6-dihydropyridine-1(2H)-carboxylate, along with 4-hydroxy-2H-chromen-2-one (74 mg, 0.46 mmol, 1.5 equiv), a stock solution of $Ir(dFppy)_3$ in CH_2Cl_2 (1.04 µM, 0.73 mL, 0.0025 equiv), and collidine•HPF_6 (16 mg, 0.06 mmol, 0.2 equiv), with the reaction mixture concentration adjusted to 18 mM. Due to the poor solubility of 4-hydroxy-2H-chromen-2-one in CH_2Cl_2, additional dilution was required, resulting in a reaction volume exceeding the capacity of an NMR tube (16 mL). Thus, the reaction was conducted in a test tube, with progress monitored by TLC (10% EtOAc in hexane) on aliquots, observing the disappearance of the higher Rf spot corresponding to **1s**. The product, **E15s**, was obtained as a white solid (77 mg, 60% yield) after purification by flash chromatography on silica gel using 30% EtOAc in hexane as eluent.

mp: 73-76 °C

^{1}H NMR (800 MHz, $CDCl_3$) δ 7.99 (dd, *J* = 7.8, 1.6 Hz, 1H), 7.56 (ddd, *J* = 8.5, 7.3, 1.6 Hz, 1H), 7.41 – 7.38 (m, 2H), 7.37 – 7.35 (m, 2H), 7.35 – 7.32 (m, 2H), 7.29 (d, *J* = 1.1 Hz, 1H), 5.07 (s, 1H), 4.15 (s, 3H), 3.18 (t, 2H), 2.54 (dd, *J* = 14.7, 2.5 Hz, 2H), 2.14 (td, *J* = 13.5, 4.9 Hz, 2H), 1.47 (s, 9H).

^{13}C{1H} NMR (201 MHz, $CDCl_3$) δ 162.3, 161.3, 154.8, 153.6, 141.8, 132.5, 129.5, 128.8, 124.8, 124.2, 123.0, 117.1, 116.6, 96.6, 83.2, 80.2, 39.6, 35.5, 28.6.

GC/MS (m/z, relative intensity) 244(5), 232(12), 204(100), 160(20), 144(7), 100(5)

Table S1 Screening of Initial Conditions

Consumption of 1 monitored by ^{19}F NMR

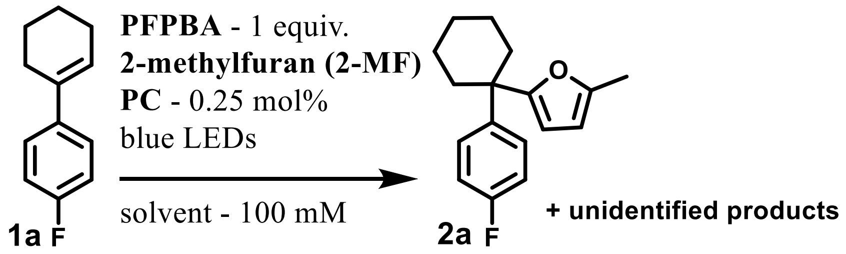

Entry	T (°C)	Solvent	Equiv. 2-MF	% Conv. to 2a	PC
1	20	MeCN	1	57	PC_B
2	20	C_6D_6	1	14	PC_B
3	20	toluene	1	11	PC_B
4	20	$CDCl_3$	1	0	PC_B
5	20	acetone	1	0	PC_B
6	20	DMSO	1	0	PC_B
7	20	DMF	1	0	PC_B
8	20	THF	1	0	PC_B
9	20	CH_2Cl_2	1	72	PC_B
10	0	CH_2Cl_2	1	80	PC_B
11	65	CH_2Cl_2	1	11	PC_B
12	20	CH_2Cl_2	2	79	PC_B
13	20	CH_2Cl_2	3	79	PC_B
14	20	CH_2Cl_2	1	9	PC_A
15	20	CH_2Cl_2	1	96	PC_C
16	20	CH_2Cl_2	1	45	PC_D

Photocatalysts (PC)

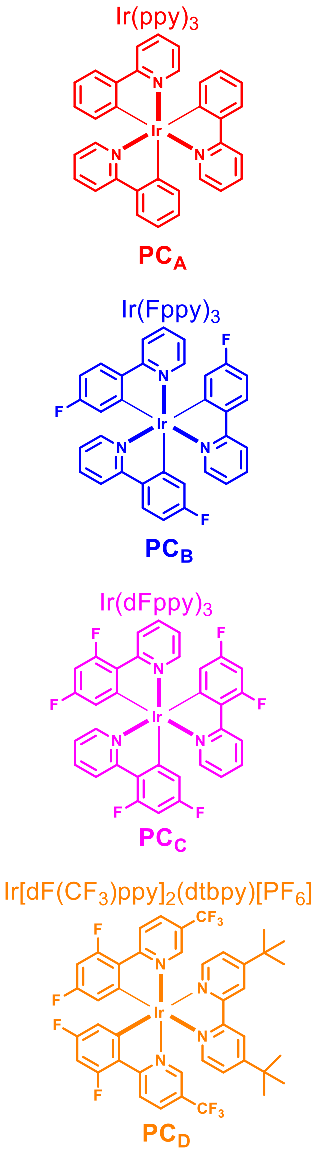

ds

Table S2 Bronsted Acid Salt Assessment, Cation Screening

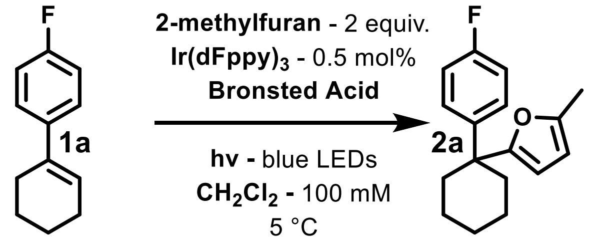

Entry	Acid (HPF_6 salt)	Mol% Acid	Time (h)	%Composition (^{19}F NMR) 1a	2a
1	*n*-Bu_2NH	9	19	55	22
2	Cy_2NH	9	19	2	78
3	2,2,6,6-tetramethylpiperidine	5	19	77	18
4	*i*-Pr_2NH	7	10	32	49
5	Et_3N	12	10	0	80
6	*i*-Pr_2EtN	11	10	55	33
7	N-Et-morpholine	4	10	27	45
8	2,4,6-collidine	7	6	2	87
9	3,5-lutidine	3	10	41	25
10	2,6-di*tert*-butyl-pyridine	8	8	76	13
11	quinoline	6	8	4	88

Table S3 The Effect of Acid Loading

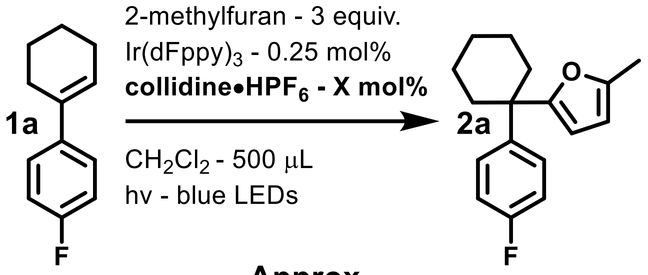

Entry	X	Approx. Initial rate (nmol/s)[a]	Terminal % Consversion[b]
1	1	5	90
2	5	11	90
3	10	~30	90
4	20	-	93
5	40	-	93

a: linear regression based off of conversion at 2, 10, and 20 minute irradiation timepoints

b: ^{19}F NMR, at 2-4 hours irradiation

Table S4 Effects of Temperature on Photo-FC Reaction

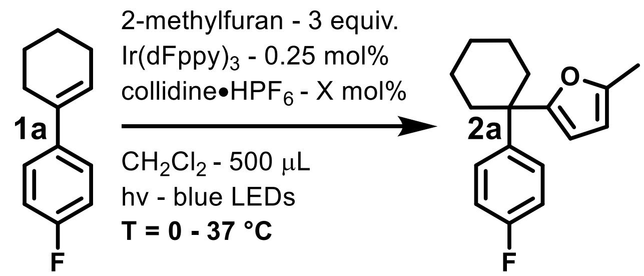

Entry	T (°C)	^{19}F NMR Conv. at 10 min. % 2a	^{19}F NMR Conv. at 2.5 h % 2a	Init. Rate (nmol/s)
1	0	41	98	36
2	10	28	99	27
3	20	20	98	19
4	30	14	98	13
5	37	8	88	8

Figure S3 Gradual Degradation of Collidine•HPF$_6$ Under FC Reaction Conditions

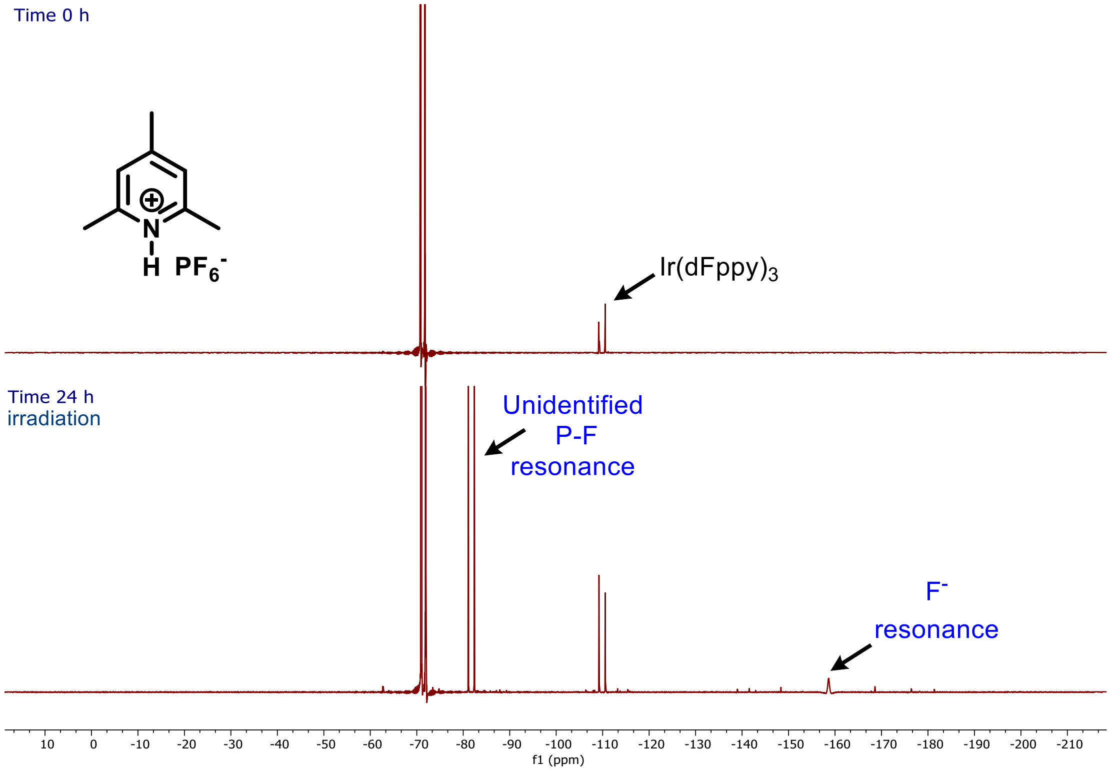

During optimization, for slower reactions, ^{19}F NMR was collected to assess the status of collidine·HPF$_6$ and Ir(dFppy)$_3$. By looking at ^{19}F NMR spectra, partial degradation of collidine HPF6 into primarily two new signals (a phosphorus bound species ca. 82 ppm and an F- species at -160 ppm) was observed. Seen at the end of most of our Friedel-Crafts reactions, these compounds were never demonstrated to hinder product formation at 20 mol% loading.

Figure S4 - FC and Cyclodimerization of 1g

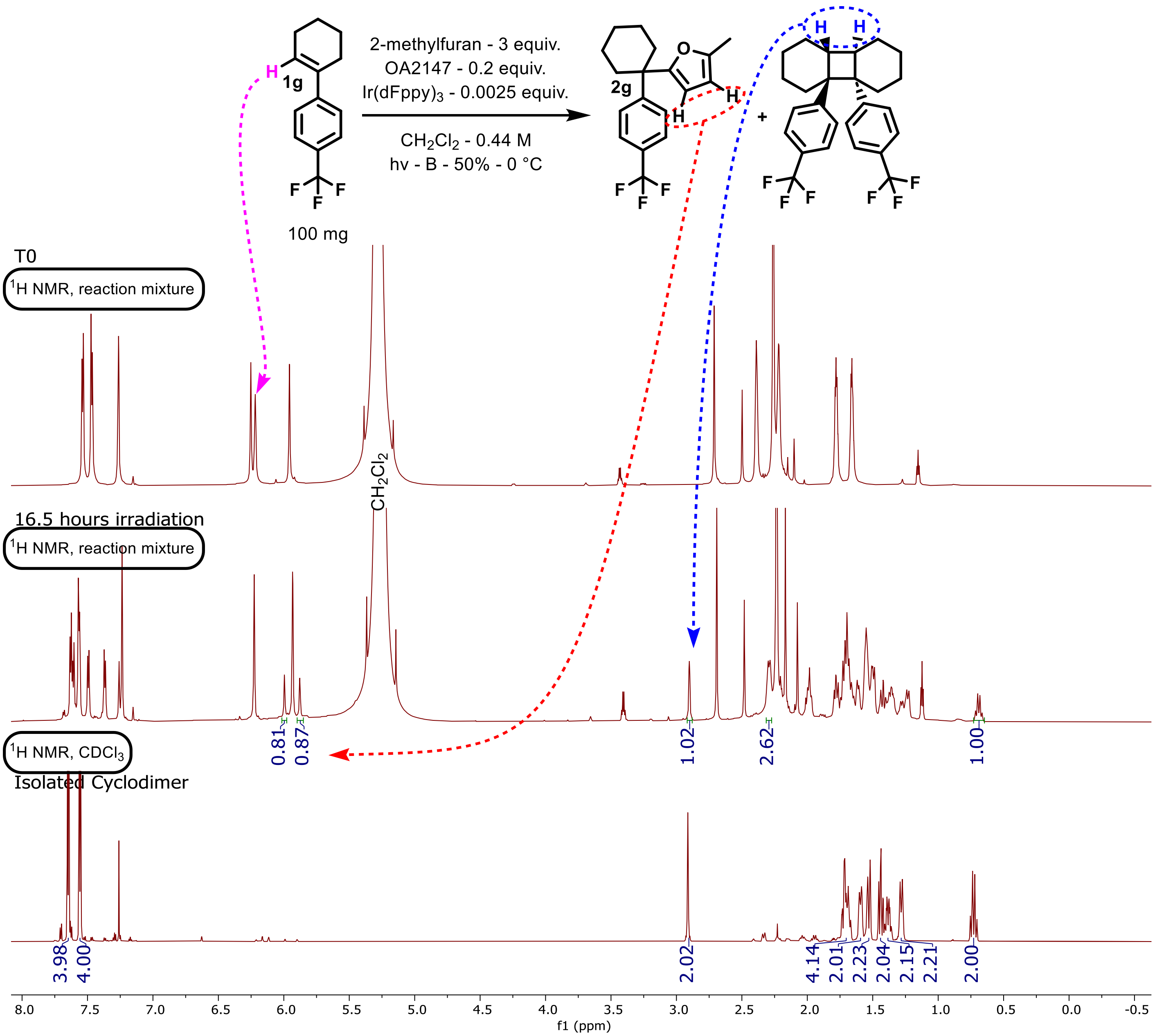

Figure S5 - Etherification of Resorcinol Monoacetate

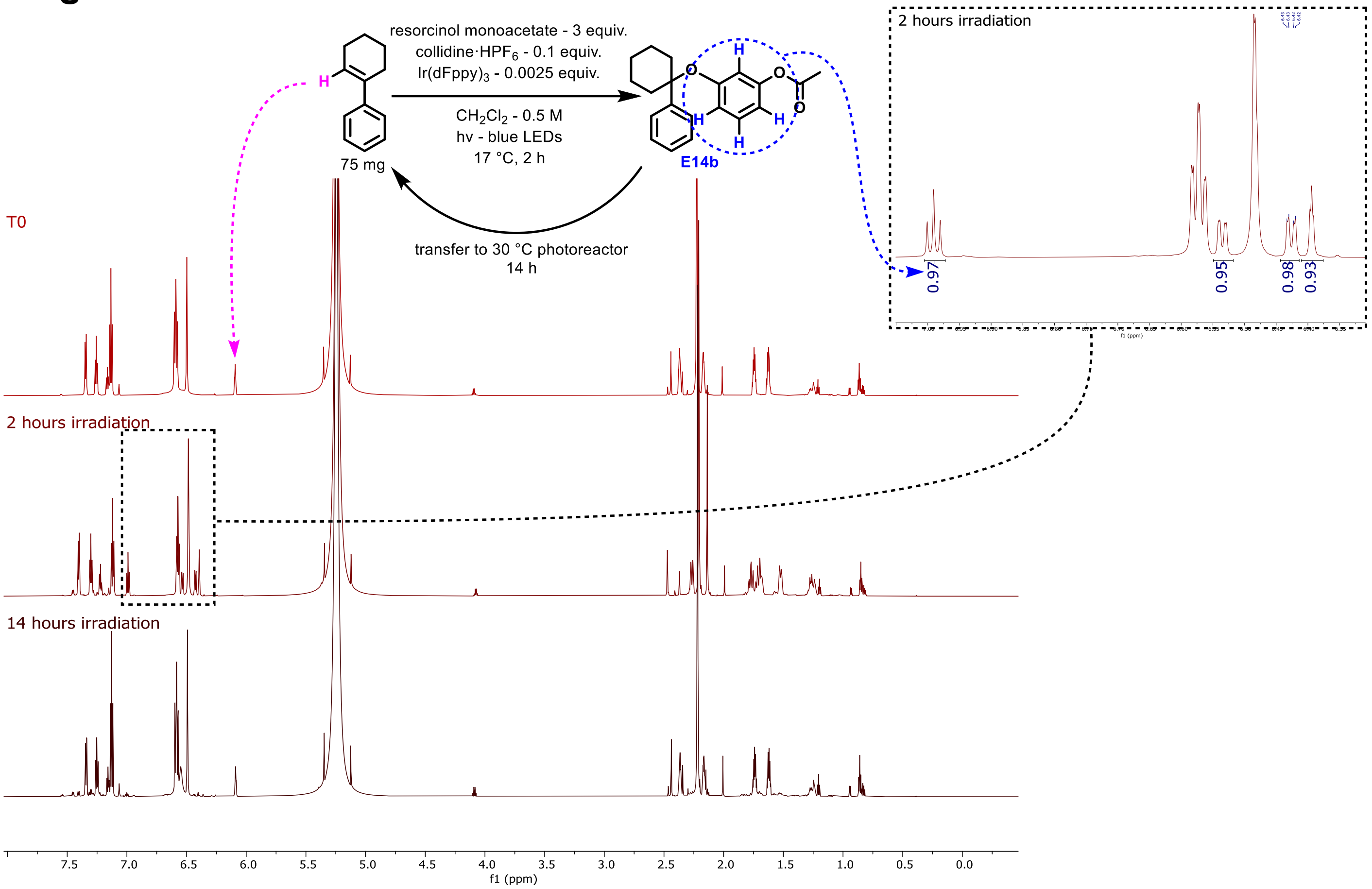

Into an NMR tube was placed 75 mg of **1b** (475 mmol), resorcinol monoacetate (216 mg, 1.42 mmol), collidine●HPF_6 (13 mg, 48 μmol), and 1 mg Ir(dFppy)$_3$ (1.2 μmol) in 1 mL of CH_2Cl_2. The NMR tube was sealed with a septum and sparged with argon through a long needle for 10 minutes at 0 °C prior to collection of a ^{1}H NMR spectrum. The tube was then immersed into a blue LED reactor at 17 °C for 2 hours before collecting another ^{1}H NMR spectrum. The disappearance of the vinylic proton resonance indicated full consumption of **1b** while the formation of a new set of 4 (rather than 3) resorcinyl signals indicated nearly quantitative ether formation over FC reactivity. The tube was transferred to a warmer blue LED reactor (30 °C) in hopes of aiding reversion to **1b** and allow for FC reactivity to occur. However, 14 hours irradiation revealed no formation of desired product according to ^{1}H NMR; only what appeared to be an equilibrium distribution of **1b** and **E14b**.

Figure S6 - Assignment of Diastereomers by Selective Gradient 1D NOESY

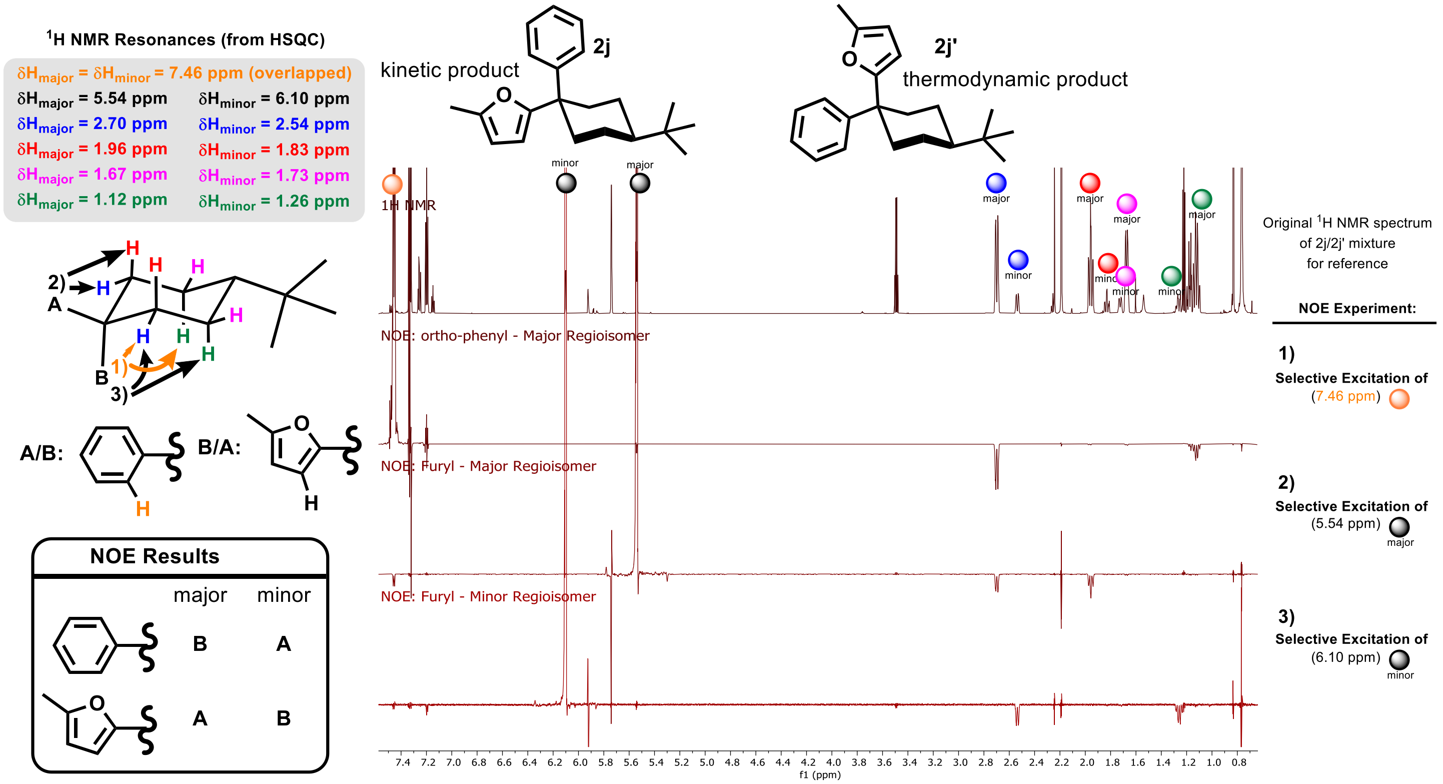

The diastereomeric assignments for **2i/2i'** (by analogy) and **2j/2j'** were carried out by identification of the alicyclic ring resonances from the HSQC spectra followed by selective gradient NOESY as exemplified above. Selective induction of the ***ortho*-phenyl proton resonance** (**Experiment 1**) clarified that the major diastereomer experienced cross-relaxation with the **axial β proton resonance**, consistent with the kinetic product being dominant. This was corroborated by selective induction of the **proximal furyl proton resonance** of the major diastereomer (**5.54 ppm**, **Experiment 2**) indicating cross-relaxation only with the **α methylene protons** at **2.70** and **1.96** ppm. Finally, selective induction of the corresponding proximal **furyl proton resonance** of the minor diastereomer **(6.10 ppm**, **Experiment 3**) indicated an expected cross relaxation with the **respective axial β protons** (1.26 ppm) consistent with **2j'** as the minor diastereomer.

Scheme S5 - Ciamician-Dennstedt Rearrangement of 5b to 5bII

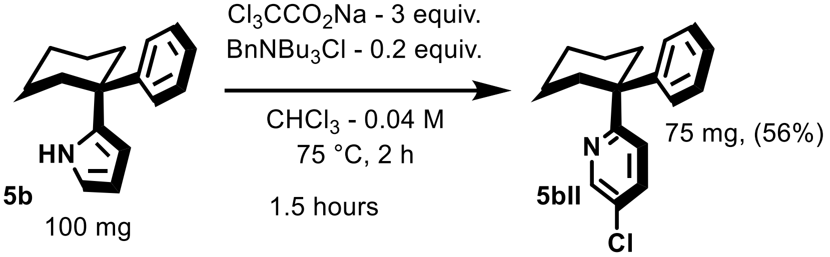

Into a 25 mL vial with a stir bar was added **5b** (100 mg , 444 µmol), sodium trichloroacetate (246 mg, 1.33 mmol), benzyltributylammonium chloride (28 mg, 88 µmol), and 10 mL of chloroform. The vial was sealed with a screwcap and set stirring in a heating bath at 75 °C for 2 hours. After the 2 hours had elapsed, approximately 4 g of silica was added, and the solvent was removed under reduced pressure in preparation for flash column chromatography. The material was eluted with pure hexane through 24 g of silica gel to obtain **5bII** as a yellow oil (75 mg, 56% yield).

^{1}H NMR: (800 MHz, $CDCl_3$) δ 8.52 (dd, J = 2.6, 0.8 Hz, 1H), 7.49 (dd, J = 8.6, 2.6 Hz, 1H), 7.34 – 7.31 (m, 2H), 7.30 – 7.26 (m, 2H), 7.15 (tt, J = 7.3, 1.3 Hz, 1H), 7.06 (d, J = 8.6 Hz, 1H), 2.56 – 2.49 (m, 2H), 2.24 – 2.17 (m, 2H), 1.61 – 1.57 (m, 2H), 1.54 – 1.48 (m, 3H), 1.48 – 1.43 (m, 1H).
^{13}C NMR: (201 MHz, $CDCl_3$) δ 165.3, 147.6, 136.0, 129.0, 128.5, 127.0, 126.0, 123.1, 48.6, 36.3, 26.3, 23.0.
GC/MS: (m/z, relative intensity) 300 (M + Et^+, 20), 272 (M^+, 100), 236 (35), 216 (10), 194 (10), 127 (10)

Scheme S6- Exploiting the Dynamic Nature of Arylcyclohexenes

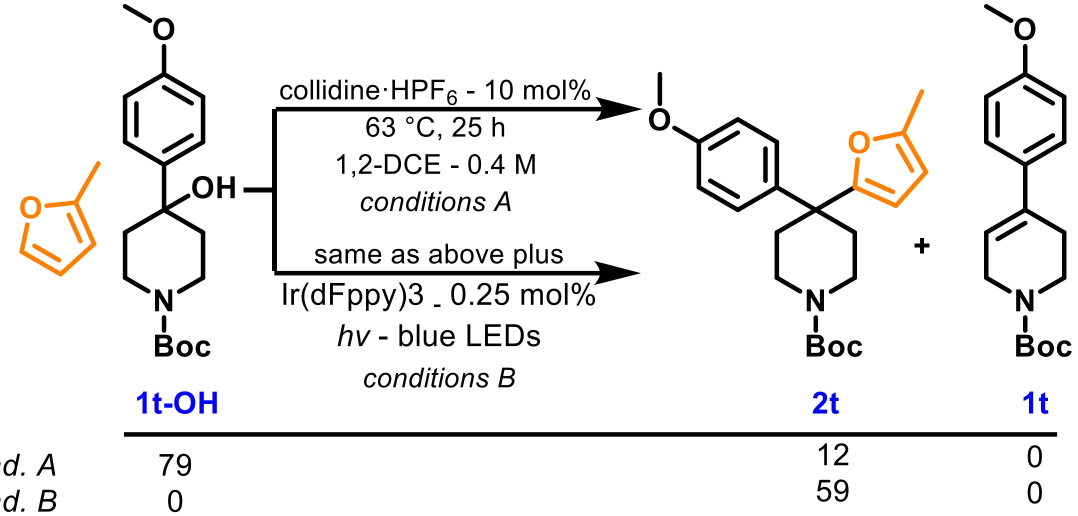

	1t-OH	2t	1t
cond. A	79	12	0
cond. B	0	59	0

The dark non-dynamic reaction (*cond*. A) began by weighing collidine●HPF6 (3.4 mg, 0.013 mmol) and adding it to an NMR tube that contained a capillary filled with C6D6. Next, 2-methyl furan (3 equivalents, 0.385 mmol, 32 mg) and alcohol 1t-OH (1 equivalent, 0.128 mmol, 39.4 mg) were measured and added to the NMR tube. Following this, 0.7 mL of DCE was introduced, and the tube was sealed with a rubber septum and parafilm. It was then placed in a water bath set to 63 °C for 32 hours.

For the dynamic photochemical reaction, a stock solution of the photocatalyst in DCM was prepared, with the required amount calculated from the stock concentration. The photocatalyst was added to an NMR tube, which was sealed with a septum. DCM was then removed using a flow of argon through two needles—one for argon introduction and the other for venting. After the DCM had completely evaporated, the remaining reagents—collidine●HPF6, 2-methyl furan, alcohol 1t-OH, and DCE—were added in the same order and amounts as in the dark reaction. A capillary tube filled with C6D6 was inserted into the NMR tube, which was sealed with a rubber septum and parafilm, and sparged with argon for 10 minutes. The NMR tube was then irradiated in a blue LED reactor at 63 °C for 32 hours, as shown in Figure S1.

Both the dark and photochemical reactions were conducted simultaneously and monitored by ^{1}H NMR. After completion, the yield of the FC product 2t for the photochemical reaction was assessed by adding 4-bromo-1-

butene (0.130 mmol, 17.5 mg) as an internal standard. To calculate the NMR yield of 2t, the vinyl alkene signal of 4-bromo-1-butene (internal standard, IS) was identified in the ^{1}H NMR spectrum, and its integration was normalized to 130 µmoles, the amount added. The methine proton of 2-methylfuran in 2t was also identified in the spectrum, and its integration was measured relative to that of the internal standard. The integration of the methine proton signal was divided by the initial mmol of 1t-OH, resulting in a 59% NMR yield of 2t for the photochemical reaction. A similar calculation was performed to determine the NMR yield of 2t for the dark reaction.

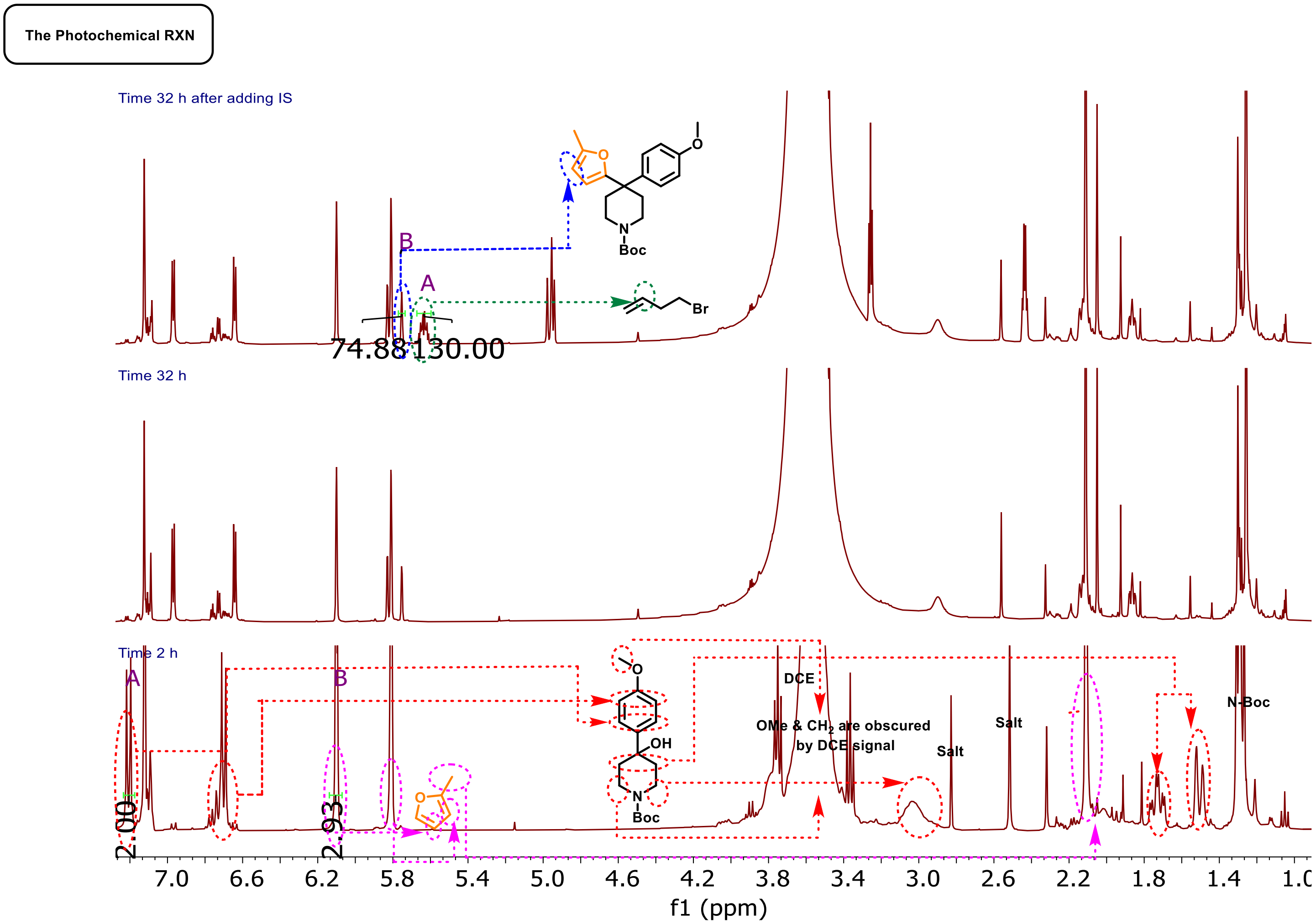

The Dark RXN

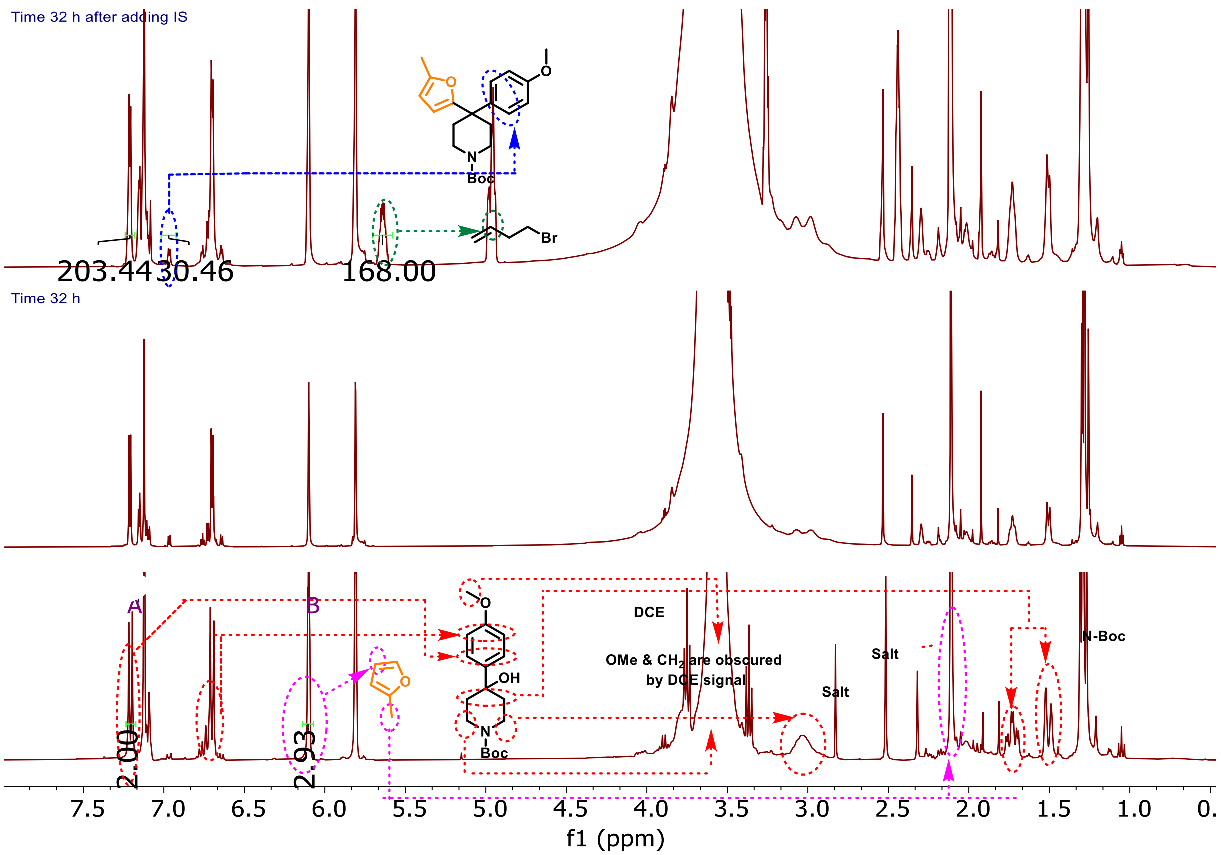
Time 32 h after adding IS
Boc
Br
203.44
30.46
168.00
Time 32 h
A
B
OH
N
Boc
DCE
OMe & CH_2 are obscured by DCE signal
Salt
Salt
N-Boc
2.00
2.93
7.5 7.0 6.5 6.0 5.5 5.0 4.5 4.0 3.5 3.0 2.5 2.0 1.5 1.0
f1 (ppm)

References

(1) Ishizuka, K.; Seike, H.; Hatakeyama, T.; Nakamura, M. Nickel-Catalyzed Alkenylative Cross-Coupling Reaction of Alkyl Sulfides. *J Am Chem Soc* **2010**, *132* (38), 13117–13119. https://doi.org/10.1021/ja104155f.

(2) Flores-Gaspar, A.; Martin, R. Mechanistic Switch via Subtle Ligand Modulation: Palladium-Catalyzed Synthesis of α,β-Substituted Styrenes via C-H Bond Functionalization. *Adv Synth Catal* **2011**, *353* (8), 1223–1228. https://doi.org/10.1002/adsc.201100140.

(3) Rono, L. J.; Yayla, H. G.; Wang, D. Y.; Armstrong, M. F.; Knowles, R. R. Enantioselective Photoredox Catalysis Enabled by Proton-Coupled Electron Transfer: Development of an Asymmetric Aza-Pinacol Cyclization. *J Am Chem Soc* **2013**, *135* (47), 17735–17738. https://doi.org/10.1021/ja4100595.

(4) Bair, J. S.; Schramm, Y.; Sergeev, A. G.; Clot, E.; Eisenstein, O.; Hartwig, J. F. Linear-Selective Hydroarylation of Unactivated Terminal and Internal Olefins with Trifluoromethyl-Substituted Arenes. *J Am Chem Soc* **2014**, *136* (38), 13098–13101. https://doi.org/10.1021/ja505579f.

(5) Ooi, T.; Maruoka, K.; Yamamoto, H. Unprecedented Stereochemical Control in the Intramolecular Ene-Reactions of δ,ε-Unsaturated Aldehydes Using Exceptionally Bulky Organoaluminum Reagents: Elucidation of the Transition State. *Tetrahedron* **1994**, *50* (22), 6505–6522. https://doi.org/10.1016/S0040-4020(01)89682-4.

(6) Ackermann, L.; Kapdi, A. R.; Fenner, S.; Kornhaab, C.; Schulzke, C. Well-Defined Air-Stable Palladium HASPO Complexes for Efficient Kumada-Corriu Cross-Couplings of (Hetero)Aryl or Alkenyl Tosylates. *Chemistry - A European Journal* **2011**, *17* (10), 2965–2971. https://doi.org/10.1002/chem.201002386.

(7) Mikhailov, B. M.; Cherkasova, K. L. Organoboron Compounds. *Bulletin of the Academy of Sciences of the USSR Division of Chemical Science* **1971**, *20* (6), 1150–1154. https://doi.org/10.1007/BF00855370.

(8) Day, J. I.; Singh, K.; Trinh, W.; Weaver, J. D. Visible Light Mediated Generation of *Trans* -Arylcyclohexenes and Their Utilization in the Synthesis of Cyclic Bridged Ethers. *J Am Chem Soc* **2018**, *140* (31), 9934–9941. https://doi.org/10.1021/jacs.8b04642.

(9) Izawa, Y.; Zheng, C.; Stahl, S. S. Aerobic Oxidative Heck/Dehydrogenation Reactions of Cyclohexenones: Efficient Access to Meta-Substituted Phenols. *Angewandte Chemie - International Edition* **2013**, *52* (13), 3672–3675. https://doi.org/10.1002/anie.201209457.

(10) Bursavich, M. G.; West, C. W.; Rich, D. H. From Peptides to Non-Peptide Peptidomimetics: Design and Synthesis of New Piperidine Inhibitors of Aspartic Peptidases. *Org Lett* **2001**, *3* (15), 2317–2320. https://doi.org/10.1021/ol016092u.

(11) Schoch, T.; Wyneken, H.; Despain, M.; Weaver, J. D. Probing the Visible Light-Driven Geometrical Isomerization of 4-Arylbut-3-ene-2-amines. *ChemCatChem* **2023**, *15* (22), e202301002. https://doi.org/10.1002/cctc.202301002.

(12) Zhang, J.; Sugisawa, N.; Felton, K. C.; Fuse, S.; Lapkin, A. A. Multi-Objective Bayesian Optimisation Using q-Noisy Expected Hypervolume Improvement (QNEHVI) for Schotten-Baumann Reaction. *ChemRxiv*. https://doi.org/10.26434/chemrxiv-2023-dlkgl.

(13) Olsson, V. J.; Szabó, K. J. Selective One-Pot Carbon-Carbon Bond Formation by Catalytic Boronation of Unactivated Cycloalkenes and Subsequent Coupling. *Angewandte Chemie - International Edition* **2007**, *46* (36), 6891–6893. https://doi.org/10.1002/anie.200702499.

(14) Nakagawa, M.; Matsuki, Y.; Nagao, K.; Ohmiya, H. A Triple Photoredox/Cobalt/Brønsted Acid Catalysis Enabling Markovnikov Hydroalkoxylation of Unactivated Alkenes. *J Am Chem Soc* **2022**. https://doi.org/10.1021/jacs.2c00527.

(15) Anderson, L. L.; Arnold, J.; Bergman, R. G. Proton-Catalyzed Hydroamination and Hydroarylation Reactions of Anilines and Alkenes: A Dramatic Effect of Counteranions on Reaction Efficiency. *J Am Chem Soc* **2005**, *127* (42), 14542–14543. https://doi.org/10.1021/ja053700i.

(16) McCubbin, J. A.; Krokhin, O. V. Organocatalyzed Friedel-Crafts Arylation of Benzylic Alcohols. *Tetrahedron Lett* **2010**, *51* (18), 2447–2449. https://doi.org/10.1016/j.tetlet.2010.02.151.

(17) Singh, K.; Trinh, W.; Weaver, J. D. An Elusive Thermal [2 + 2] Cycloaddition Driven by Visible Light Photocatalysis: Tapping into Strain to Access C 2-Symmetric Tricyclic Rings. *Org Biomol Chem* **2019**, *17* (7), 1854–1861. https://doi.org/10.1039/c8ob01273c.

[1a] 4'-fluoro-2,3,4,5-tetrahydro-1,1'-biphenyl
1H NMR collected at 800.34 MHz in CDCl3

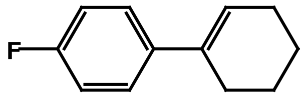

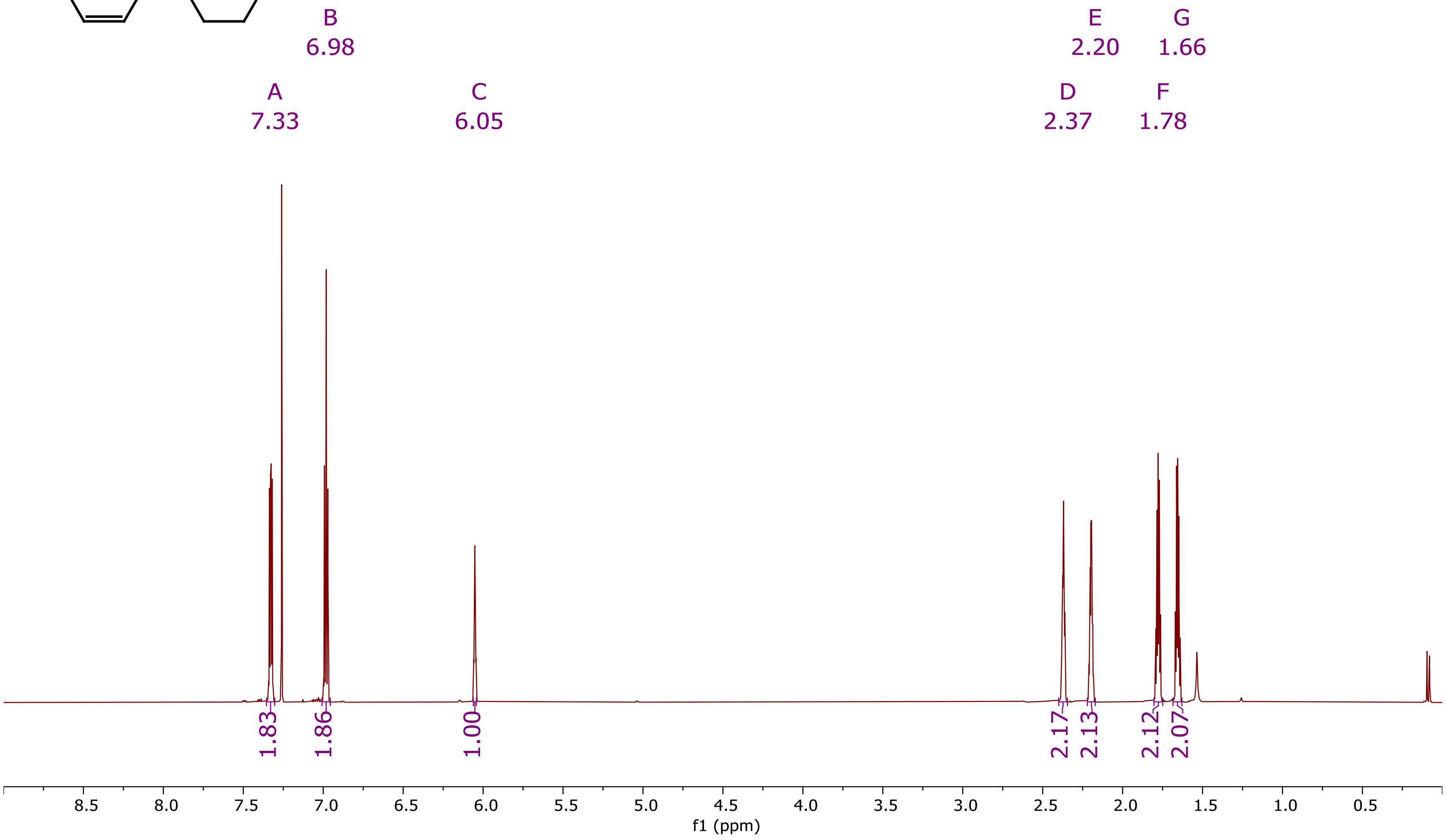

[1a] 4'-fluoro-2,3,4,5-tetrahydro-1,1'-biphenyl
13C NMR collected at 201.27 MHz in CDCl3

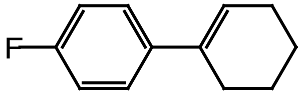

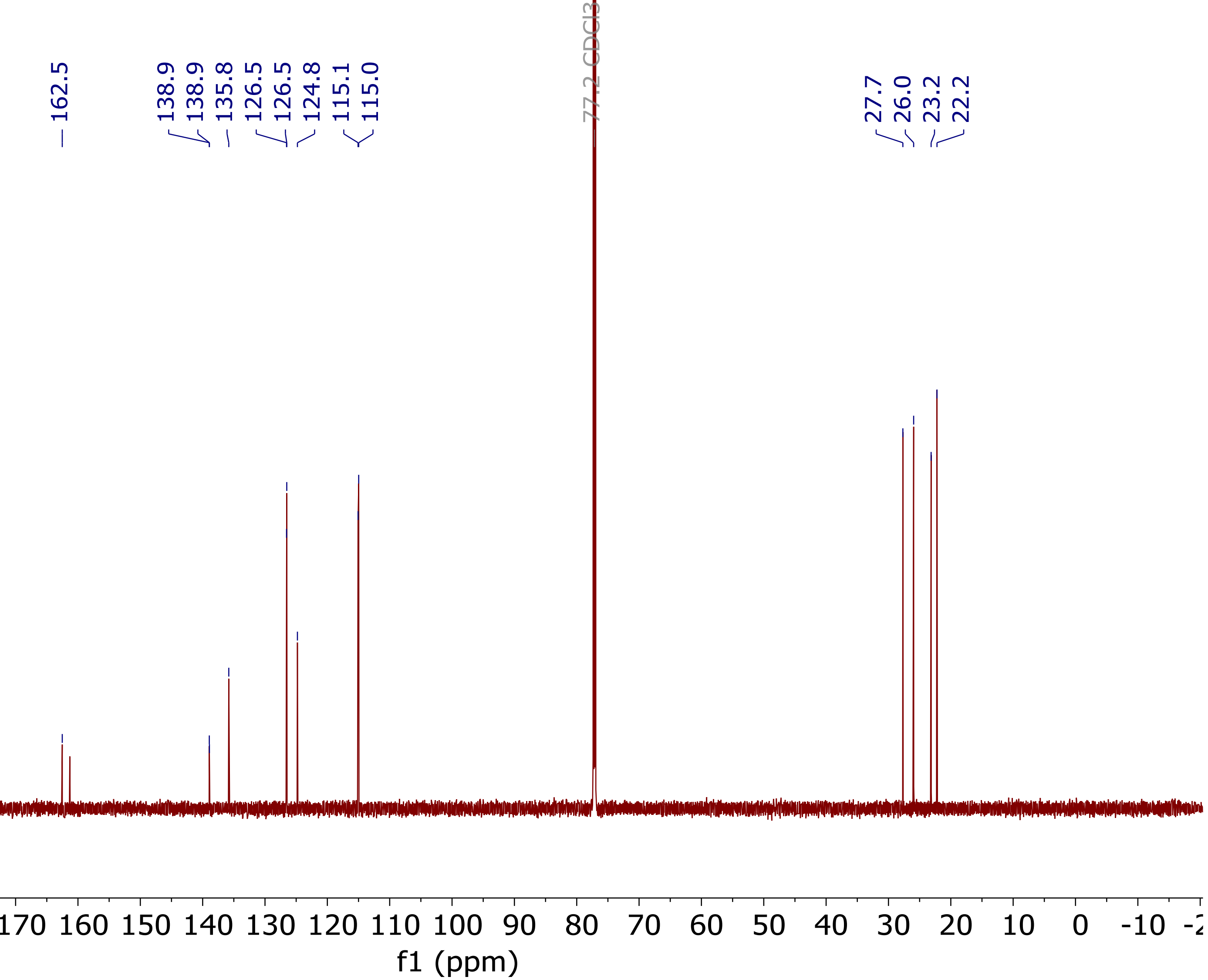

[1a] 4'-fluoro-2,3,4,5-tetrahydro-1,1'-biphenyl
19F NMR collected at 753.00 MHz in CDCl3

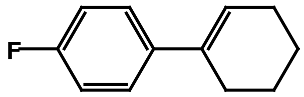

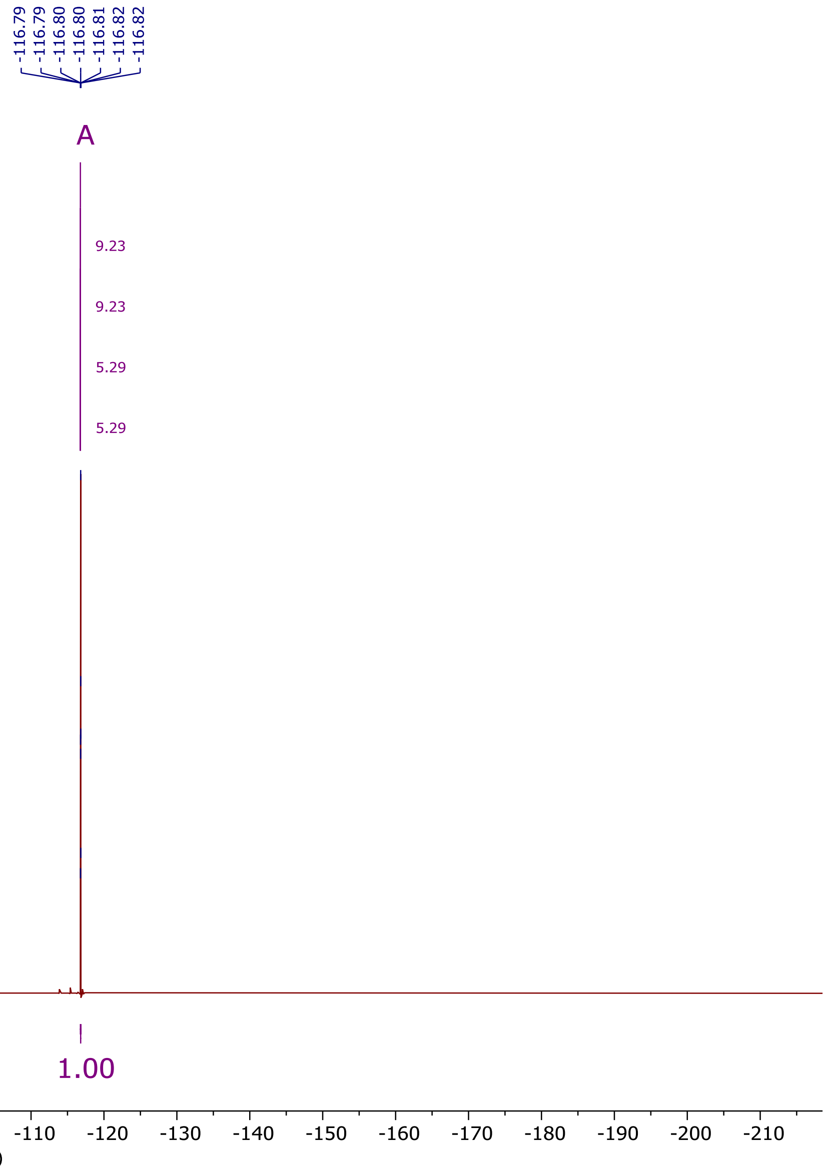

[1b] 2,3,4,5-tetrahydro-1,1'-biphenyl
1H NMR collected at 800.34 MHz in CDCl3

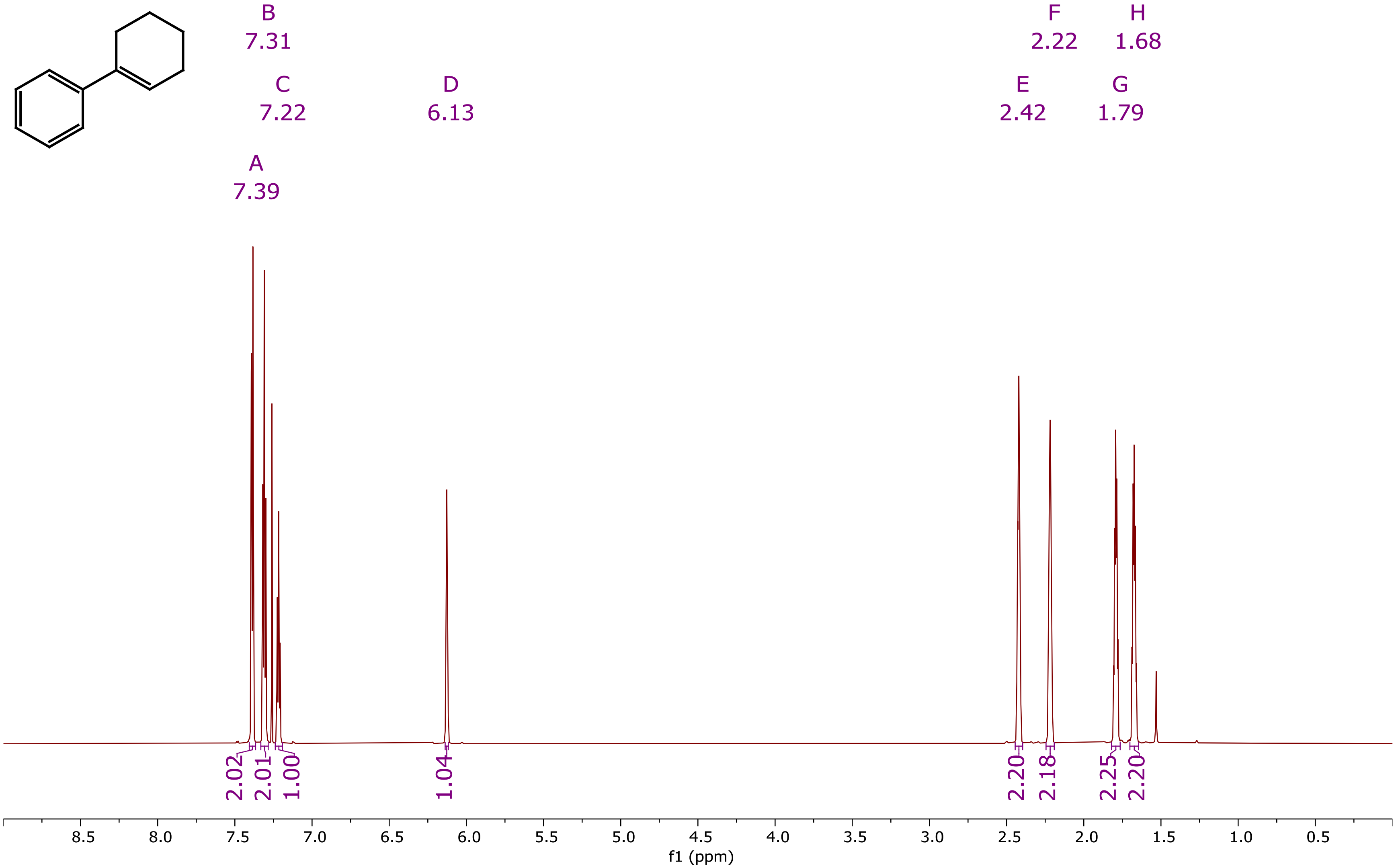

[1b] 2,3,4,5-tetrahydro-1,1'-biphenyl
13C NMR collected at 201.27 MHz in CDCl3

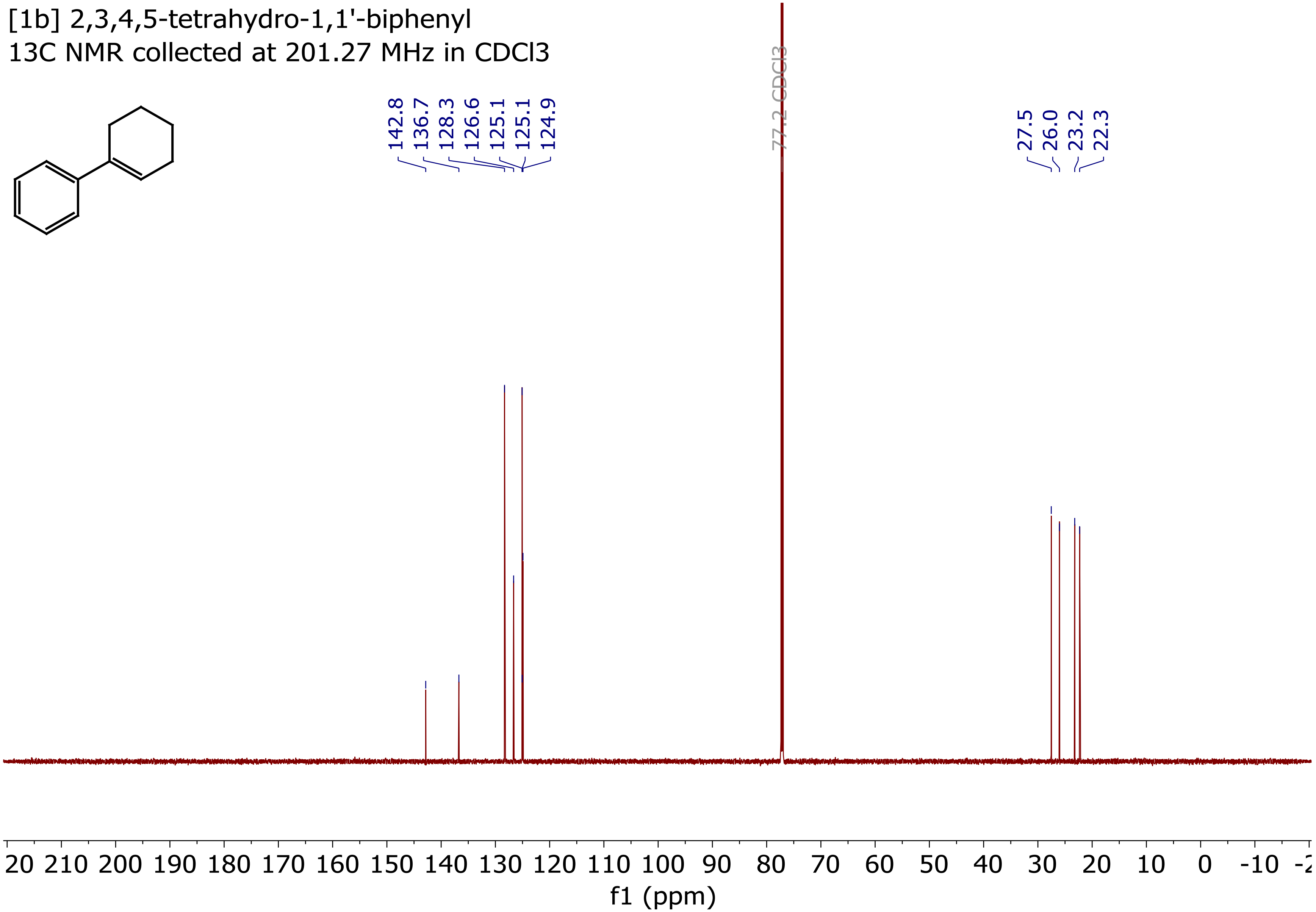

[1c] 4'-methyl-2,3,4,5-tetrahydro-1,1'-biphenyl
1H NMR collected at 800.34 MHz in CDCl3

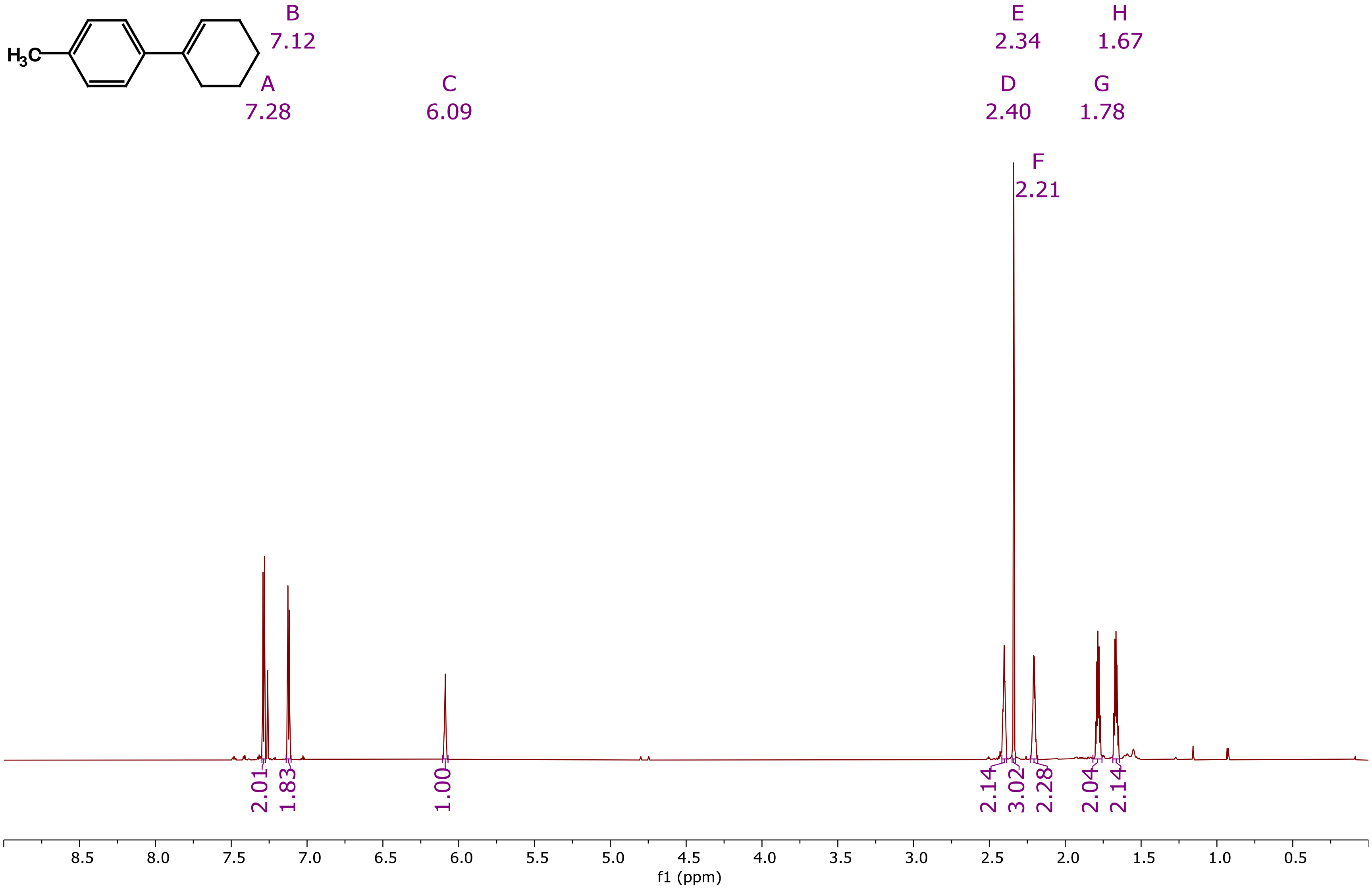

[1c] 4'-methyl-2,3,4,5-tetrahydro-1,1'-biphenyl
13C NMR collected at 201.27 MHz in CDCl3

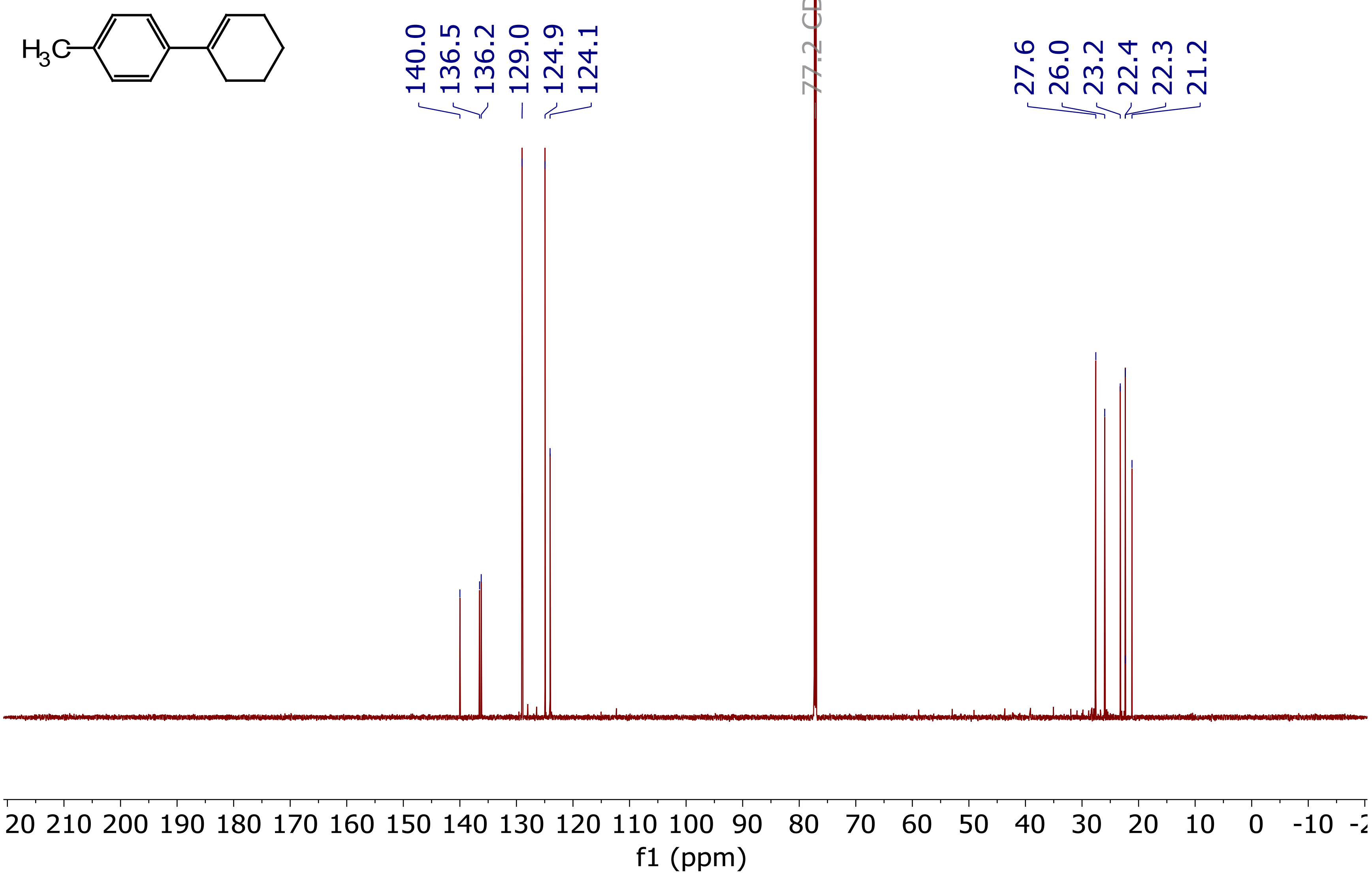

[1e] 4'-methoxy-2,3,4,5-tetrahydro-1,1'-biphenyl
1H NMR collected at 800.34 MHz in CDCl3

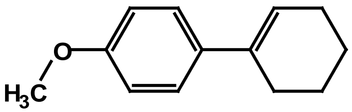

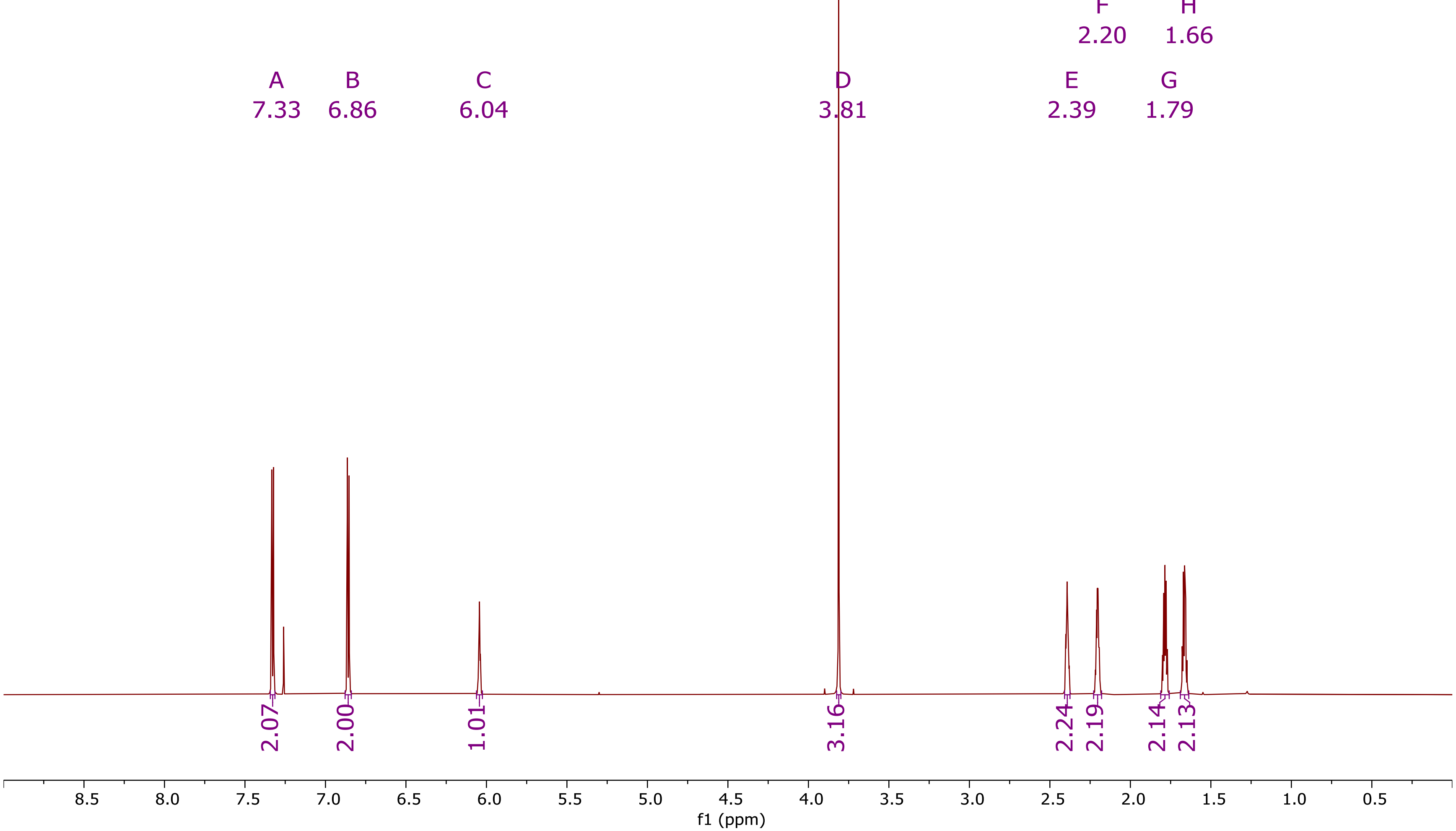

[1e] 4'-methoxy-2,3,4,5-tetrahydro-1,1'-biphenyl
13C NMR collected at 201.27 MHz in CDCl3

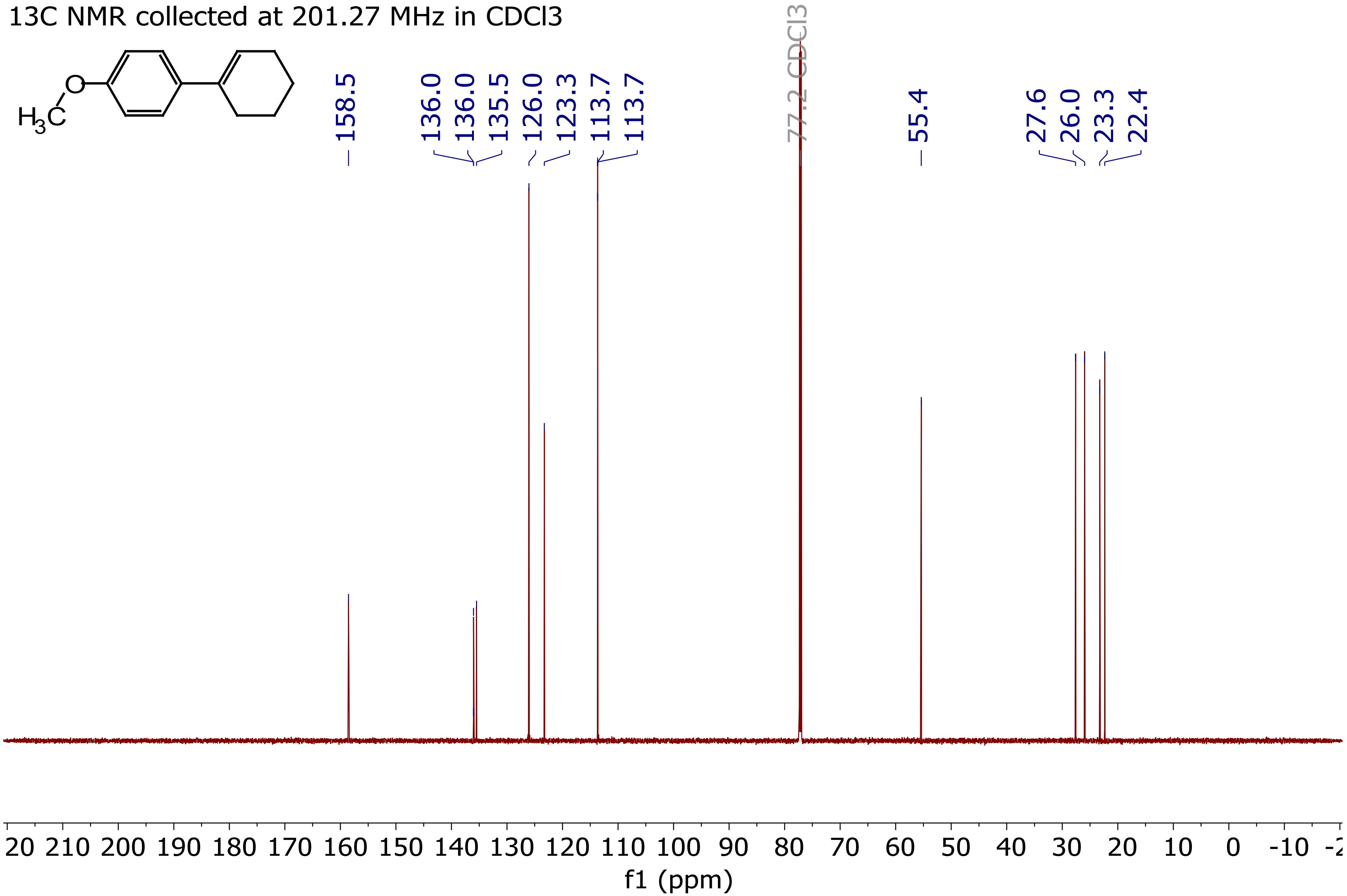

[1f] - 5-(cyclohex-1-en-1-yl)benzo[d][1,3]dioxole
1H NMR collected at 800.34 MHz in CDCl3

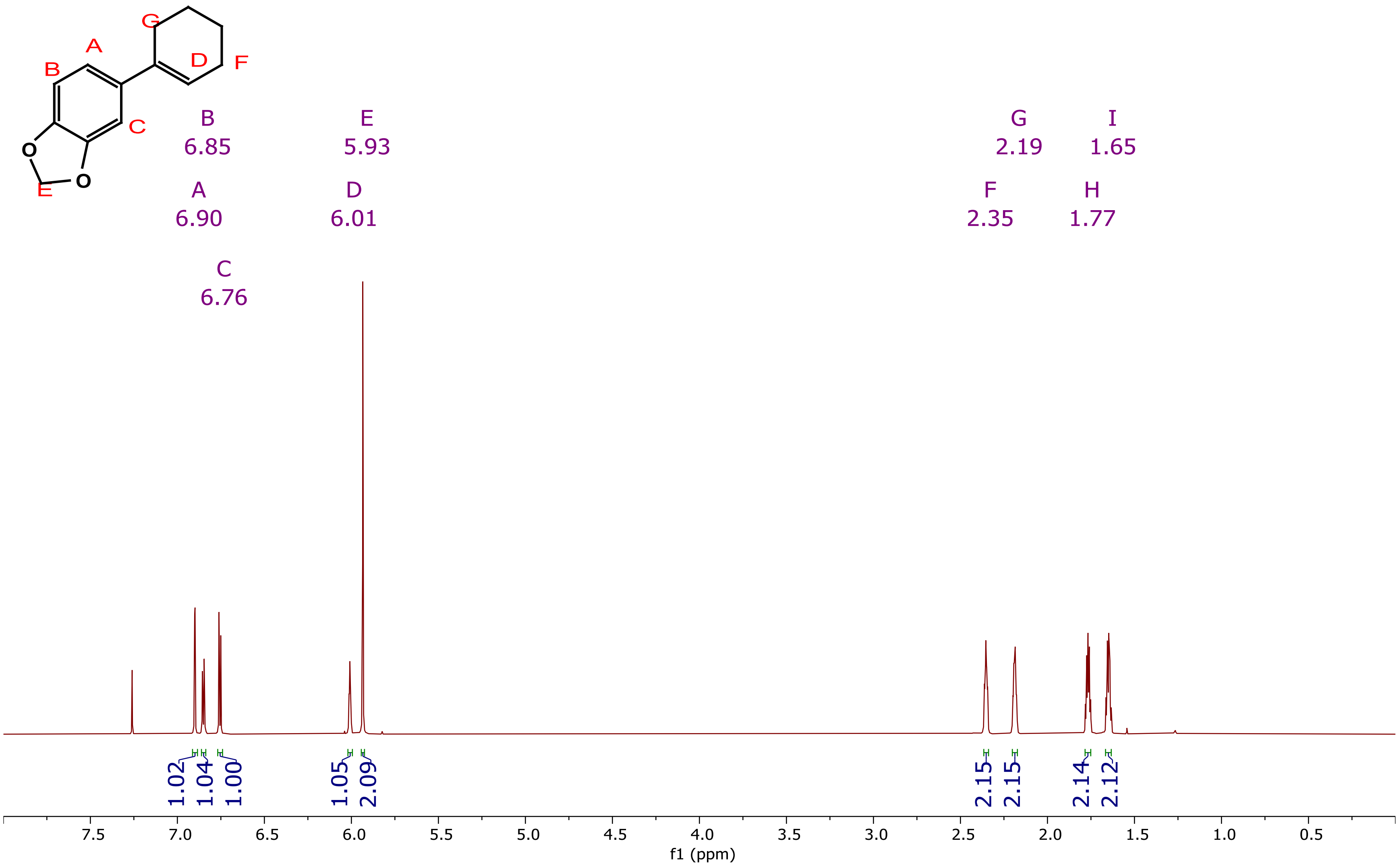

[1f] - 5-(cyclohex-1-en-1-yl)benzo[d][1,3]dioxole
13C NMR collected at 201.27 MHz in CDCl3

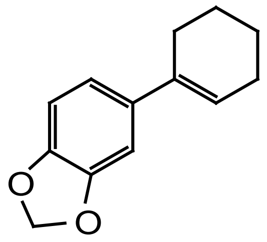

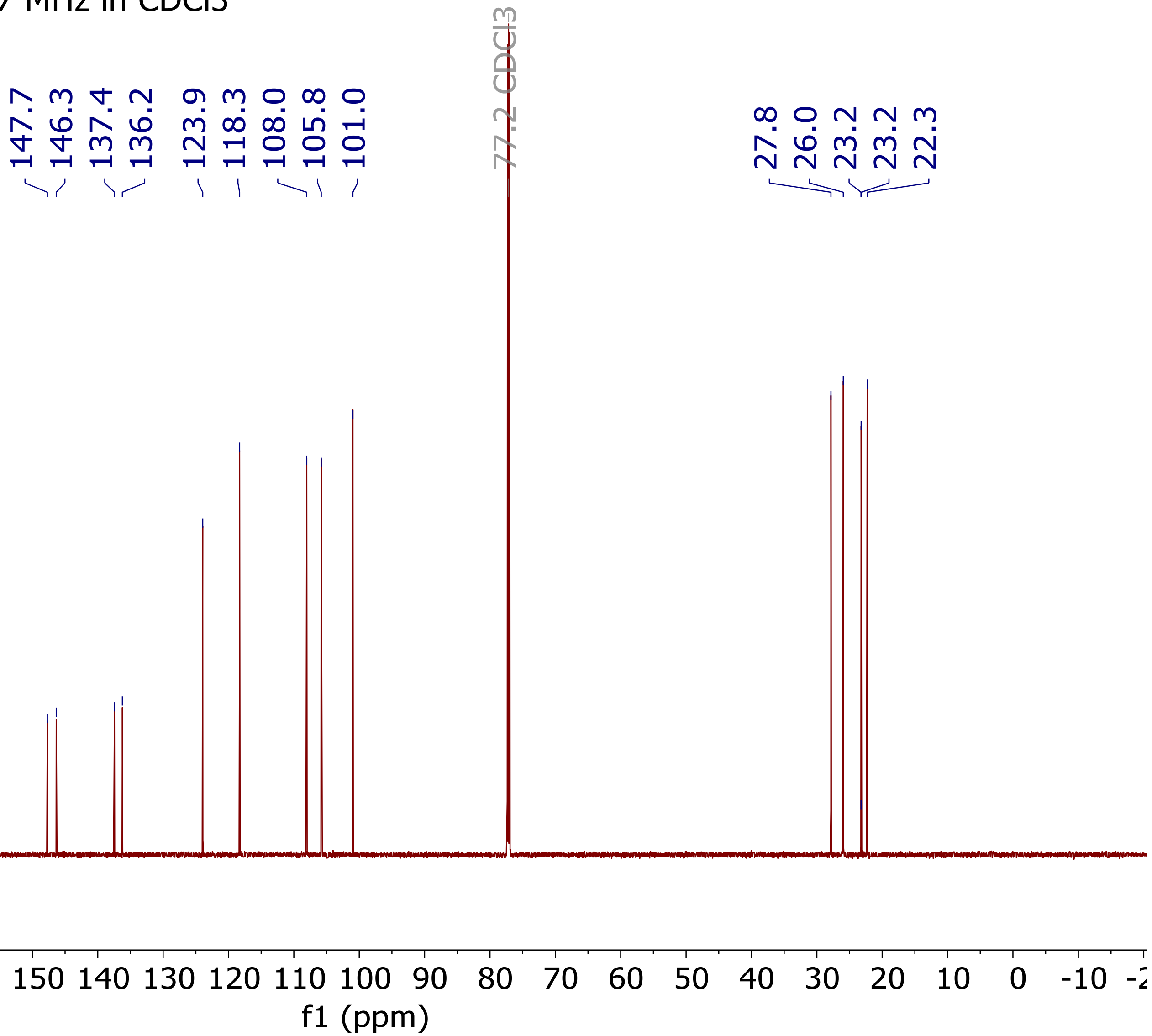

[1g] 4'-(trifluoromethyl)-2,3,4,5-tetrahydro-1,1'-biphenyl
1H NMR collected at 800.34 MHz in CDCl3

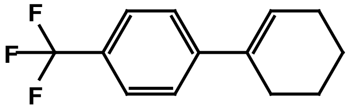

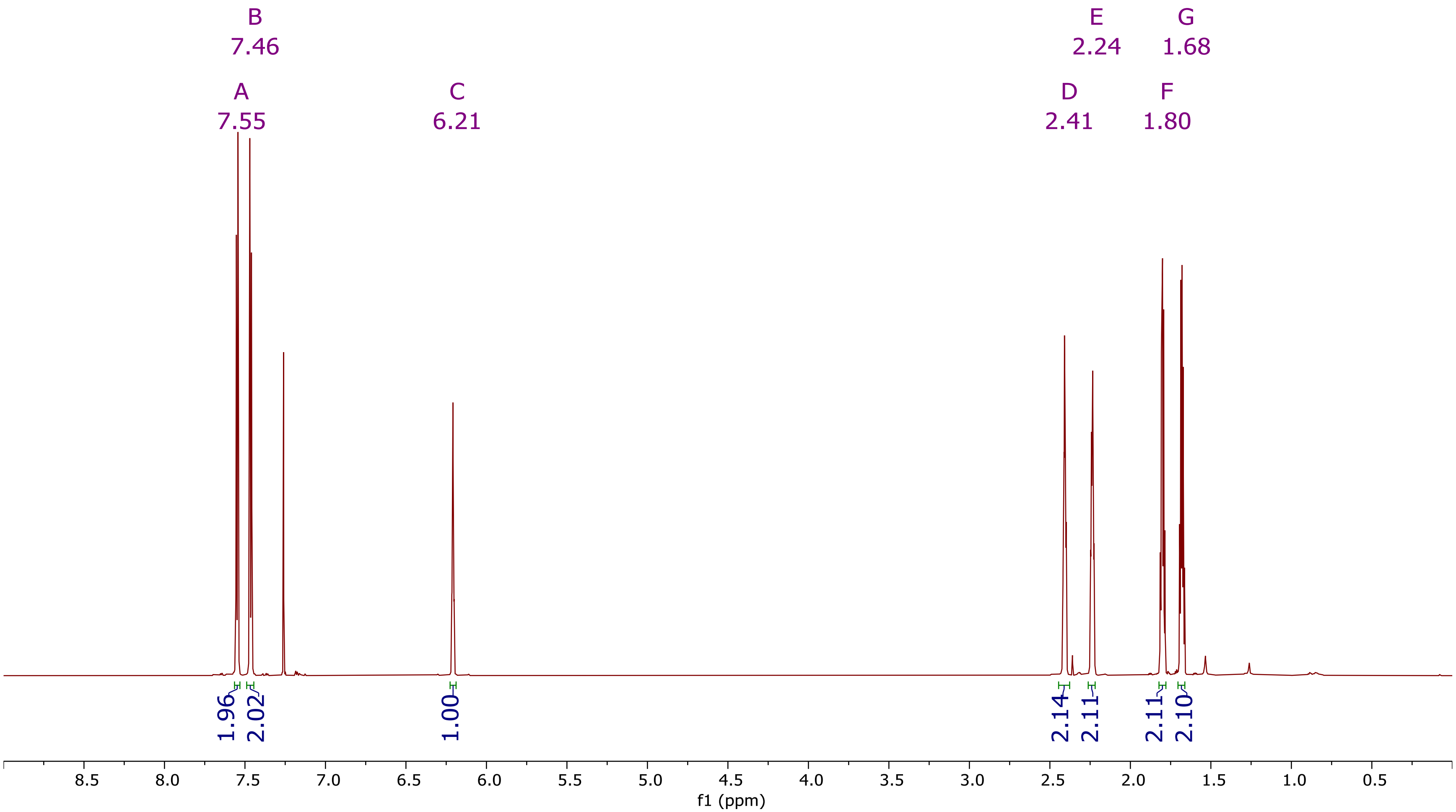

[1g] 4'-(trifluoromethyl)-2,3,4,5-tetrahydro-1,1'-biphenyl
13C NMR collected at 201.27 MHz in CDCl3

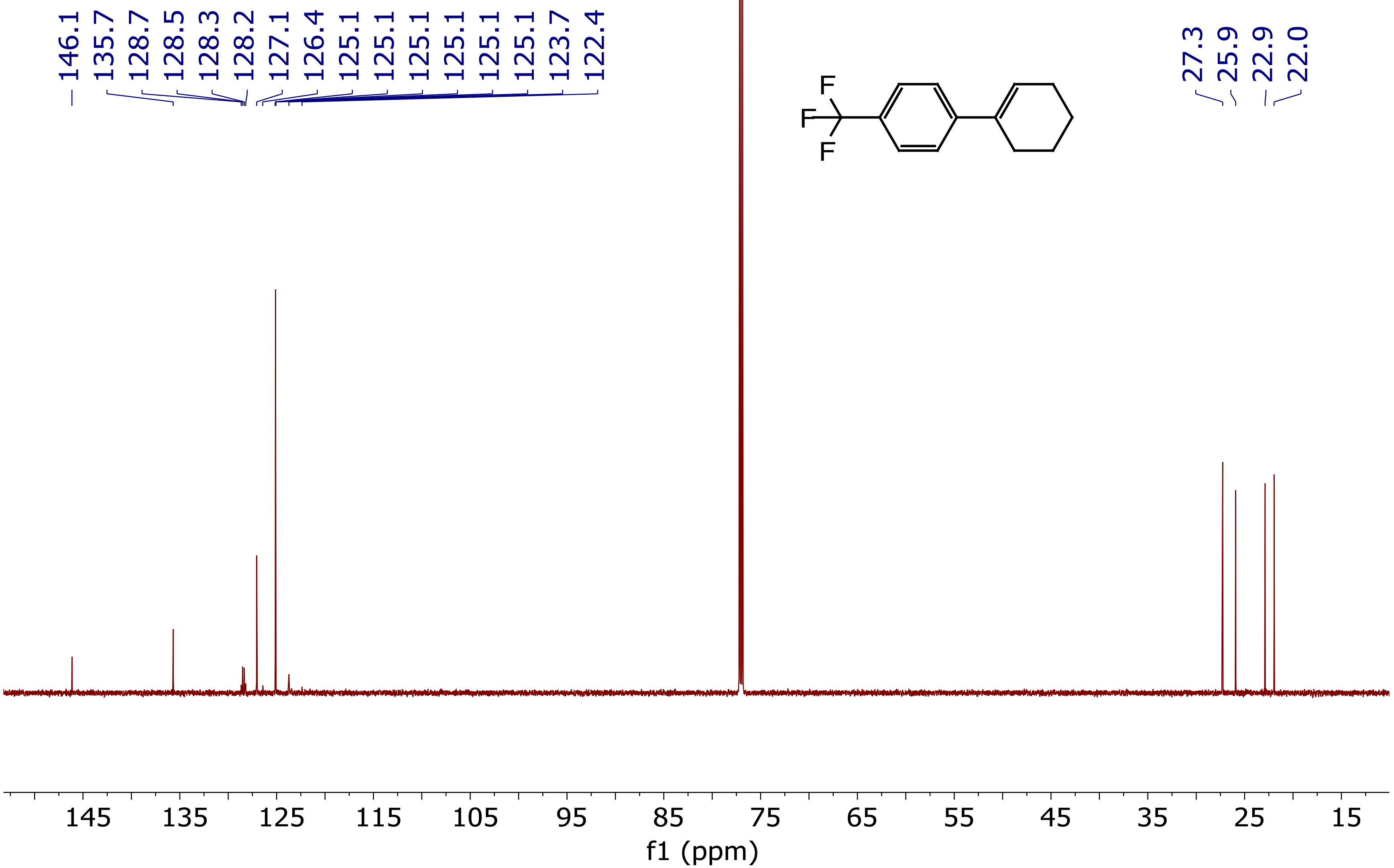

[1g] 4'-(trifluoromethyl)-2,3,4,5-tetrahydro-1,1'-biphenyl
19F NMR collected at 753.00 MHz in CDCl3

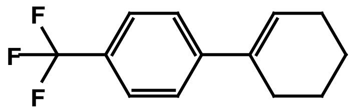

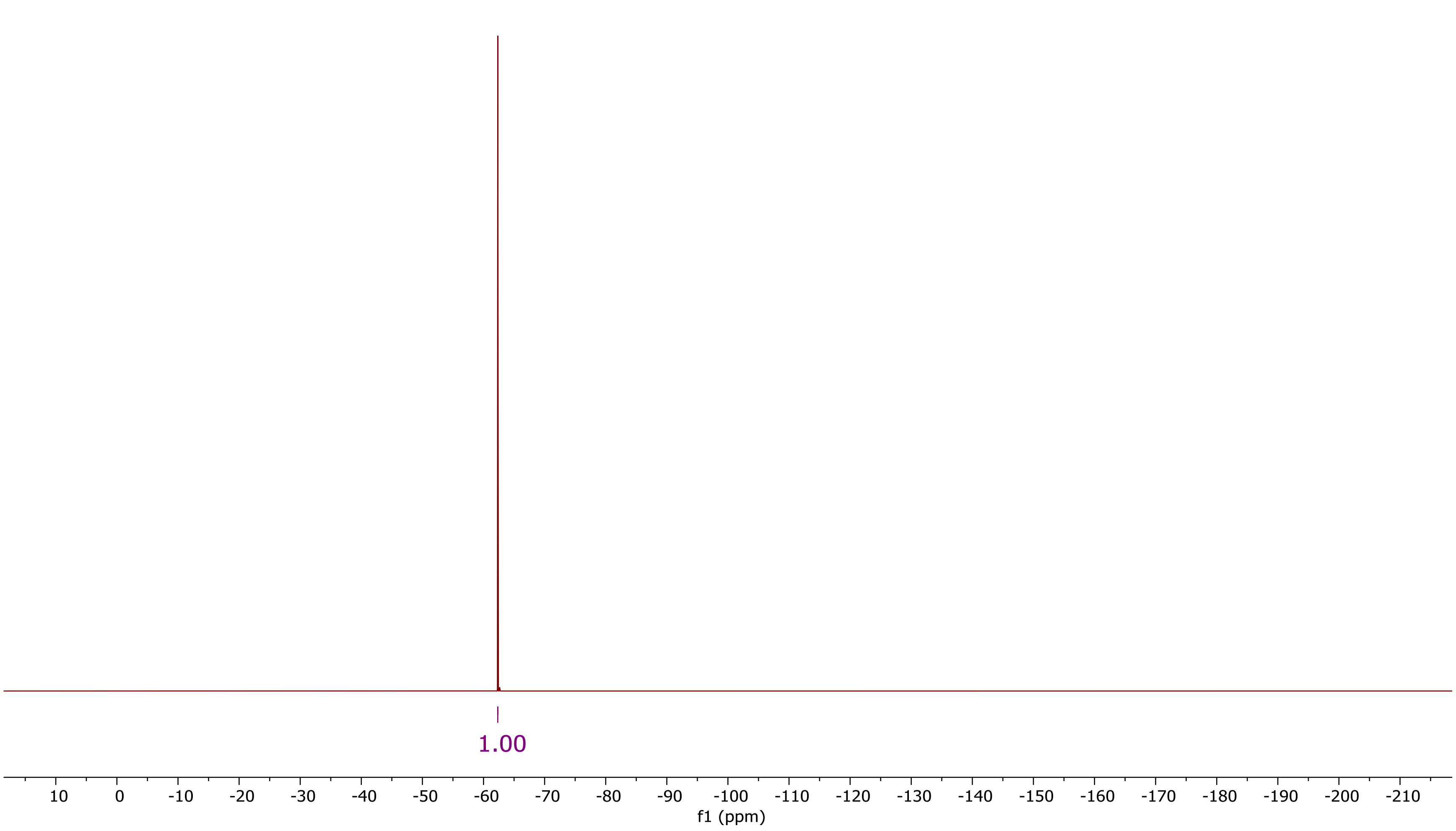

[1h] 3',5'-bis(trifluoromethyl)-2,3,4,5-tetrahydro-1,1'-biphenyl
1H NMR collected at 800.34 MHz in CDCl3

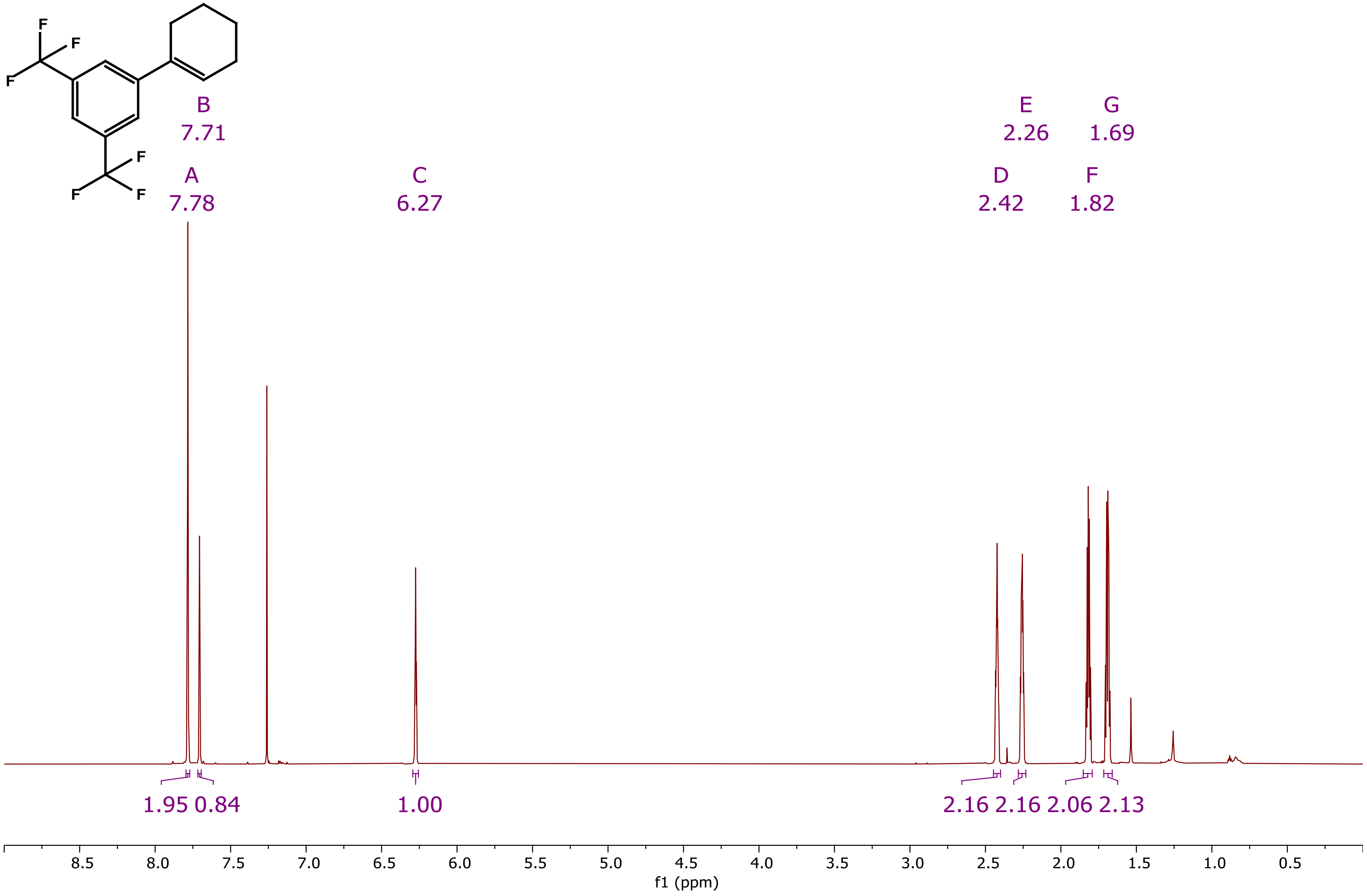

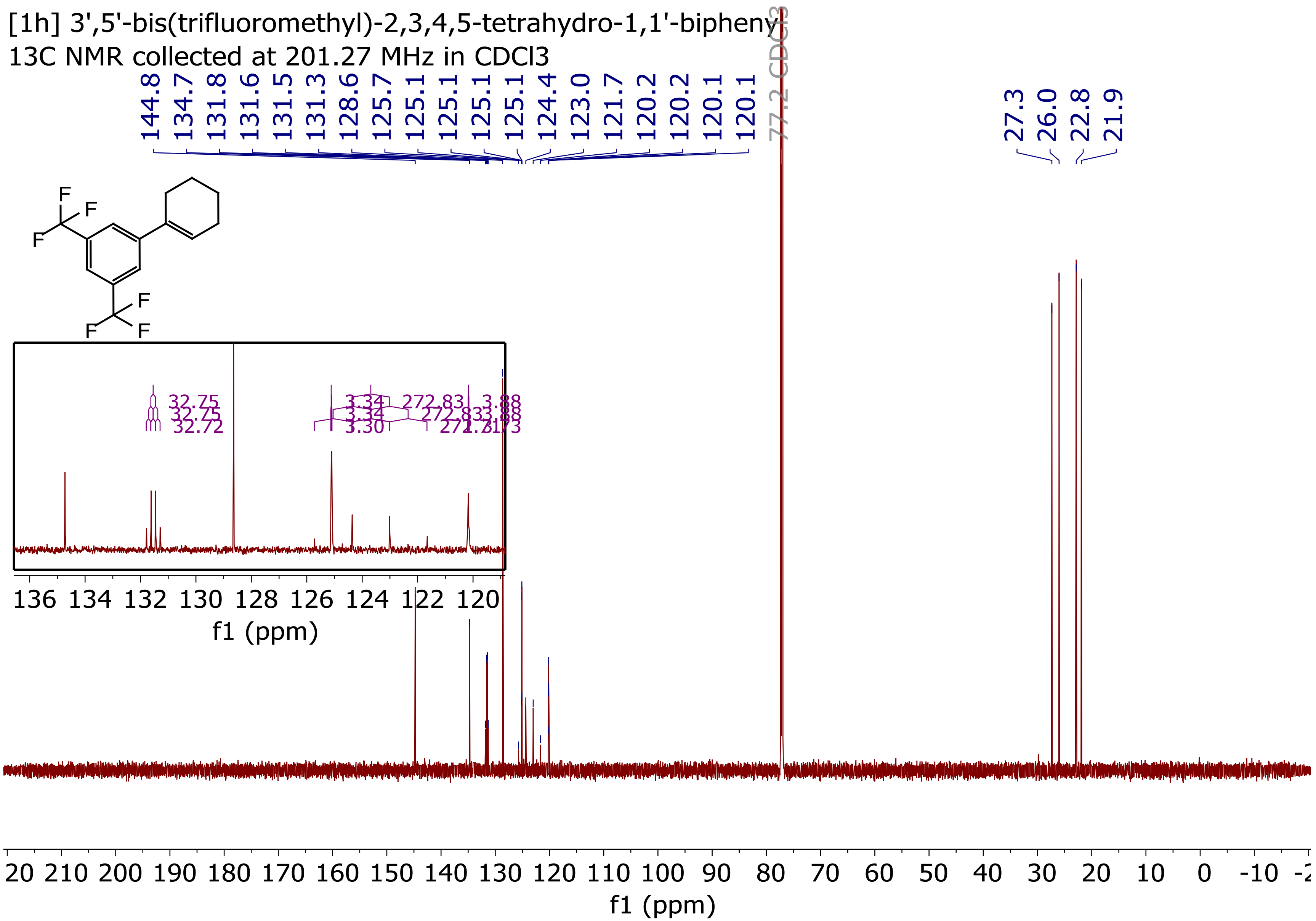
[1h] 3',5'-bis(trifluoromethyl)-2,3,4,5-tetrahydro-1,1'-biphenyl
13C NMR collected at 201.27 MHz in CDCl3
144.8
134.7
131.8
131.6
131.5
131.3
128.6
125.7
125.1
125.1
125.1
125.1
124.4
123.0
121.7
120.2
120.2
120.1
120.1
77.2 CDCl3
27.3
26.0
22.8
21.9
F
32.75
32.75
32.72
3.34
3.34
3.30
272.83
136 134 132 130 128 126 124 122 120
f1 (ppm)
210 200 190 180 170 160 150 140 130 120 110 100 90 80 70 60 50 40 30 20 10 0 -10
f1 (ppm)

[1h] 3',5'-bis(trifluoromethyl)-2,3,4,5-tetrahydro-1,1'-biphenyl
19F NMR collected at 753.00 MHz in CDCl3

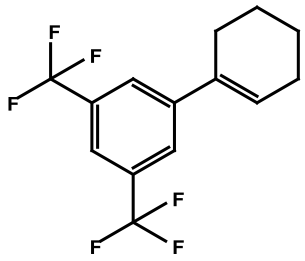

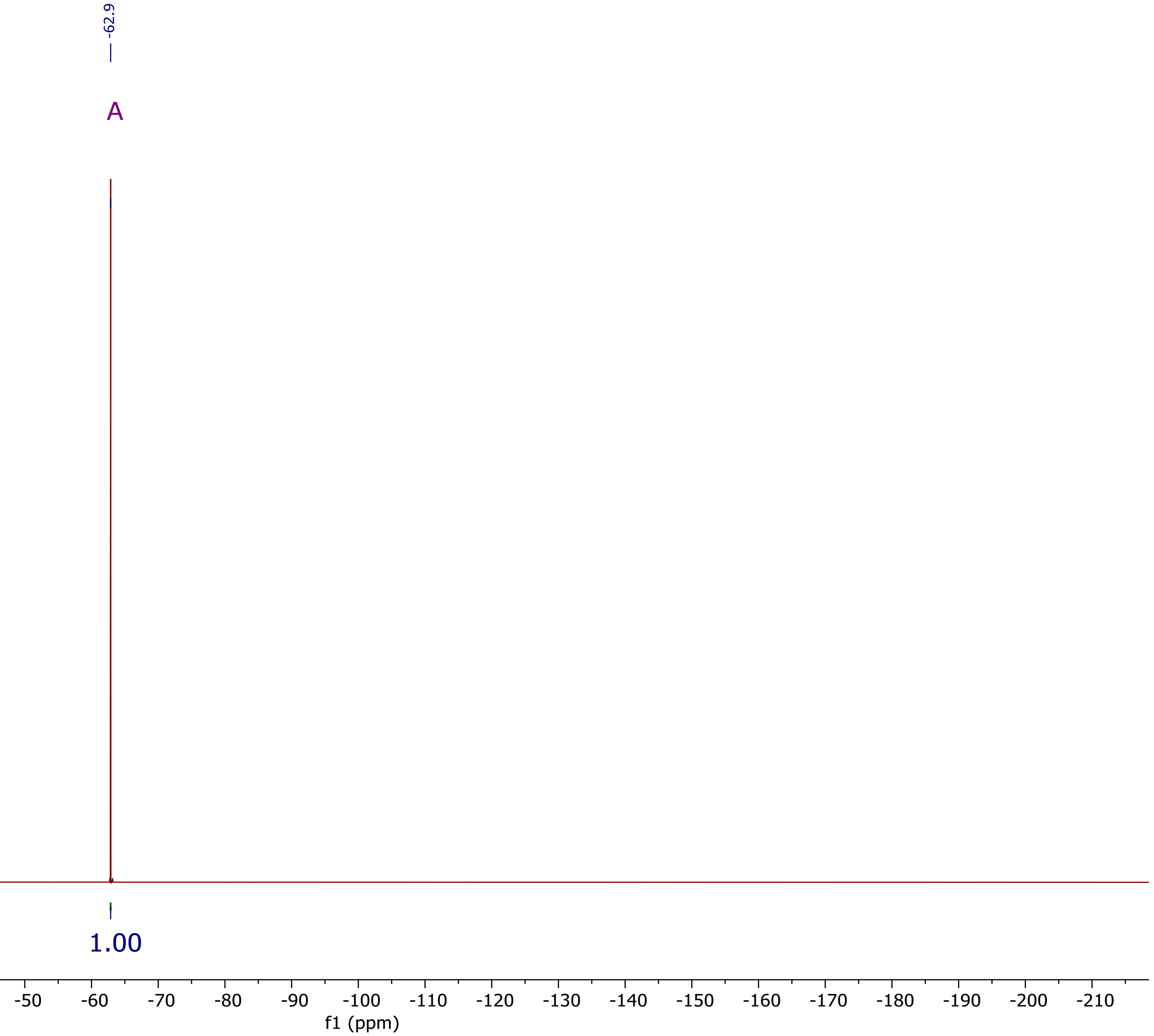

[1i] 4-methyl-2,3,4,5-tetrahydro-1,1'-biphenyl
1H NMR collected at 800.34 MHz in CDCl3

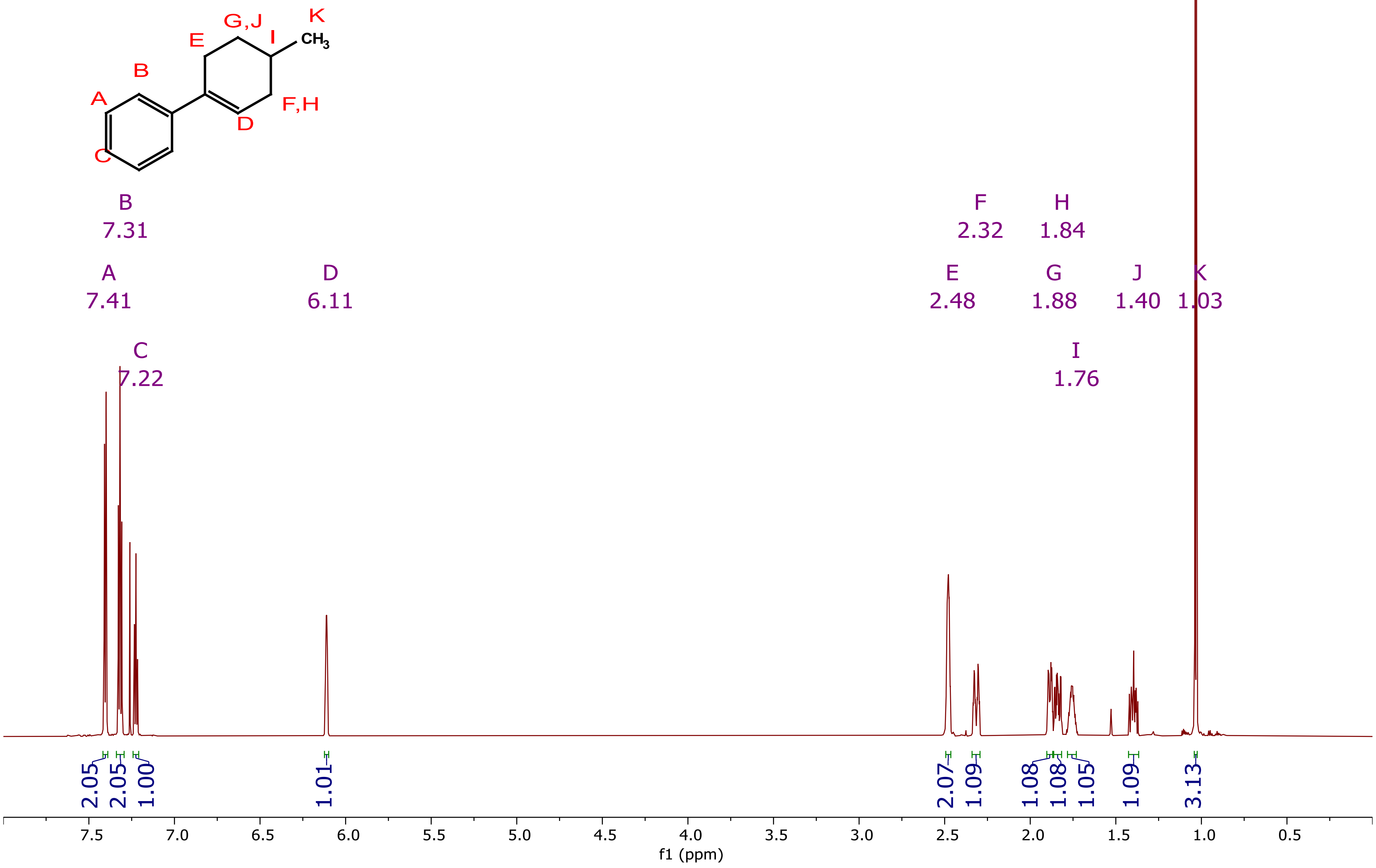

[1i] 4-methyl-2,3,4,5-tetrahydro-1,1'-biphenyl
13C NMR collected at 201.27 MHz in CDCl3

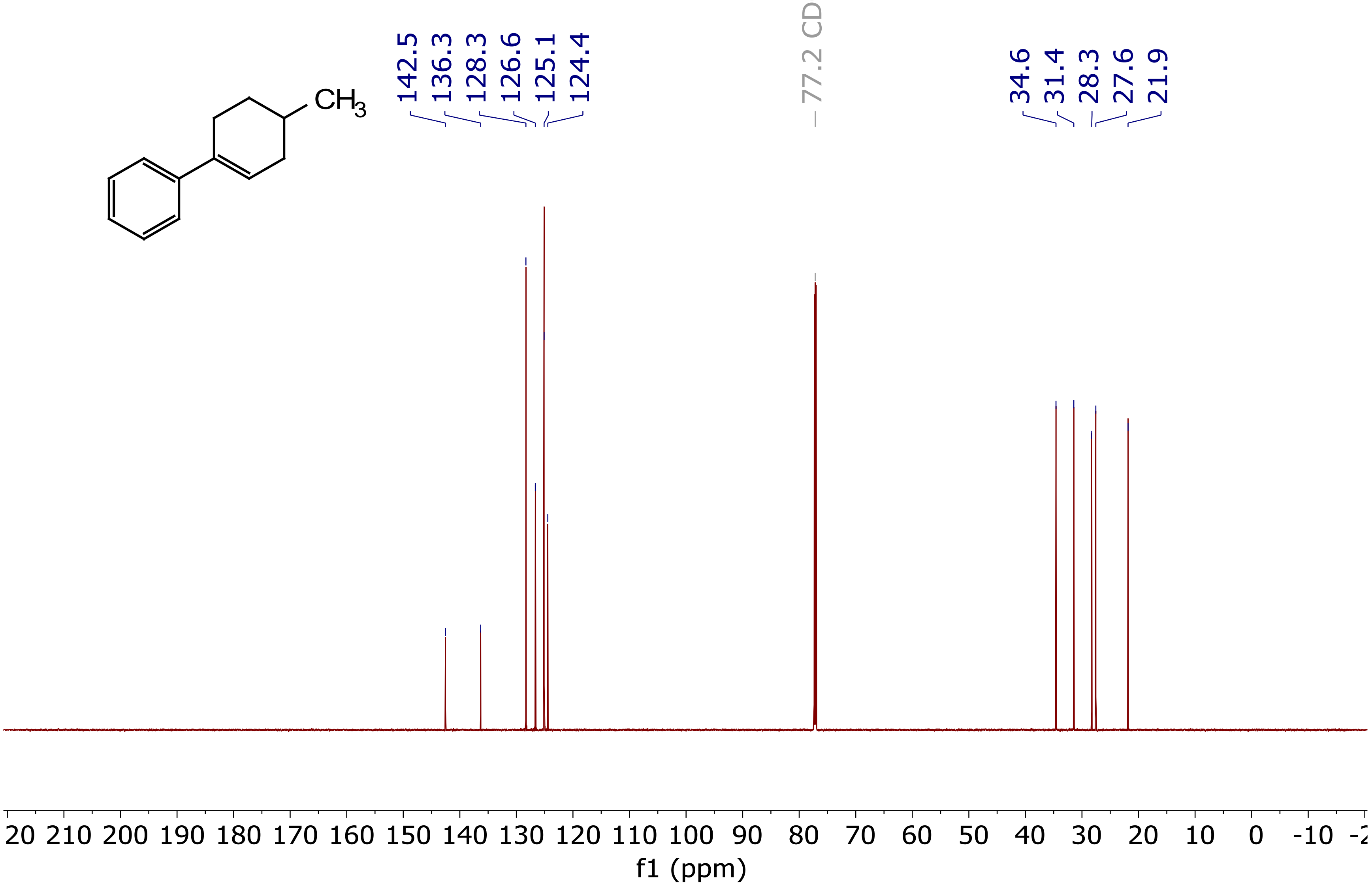

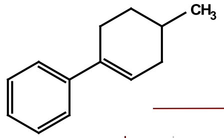

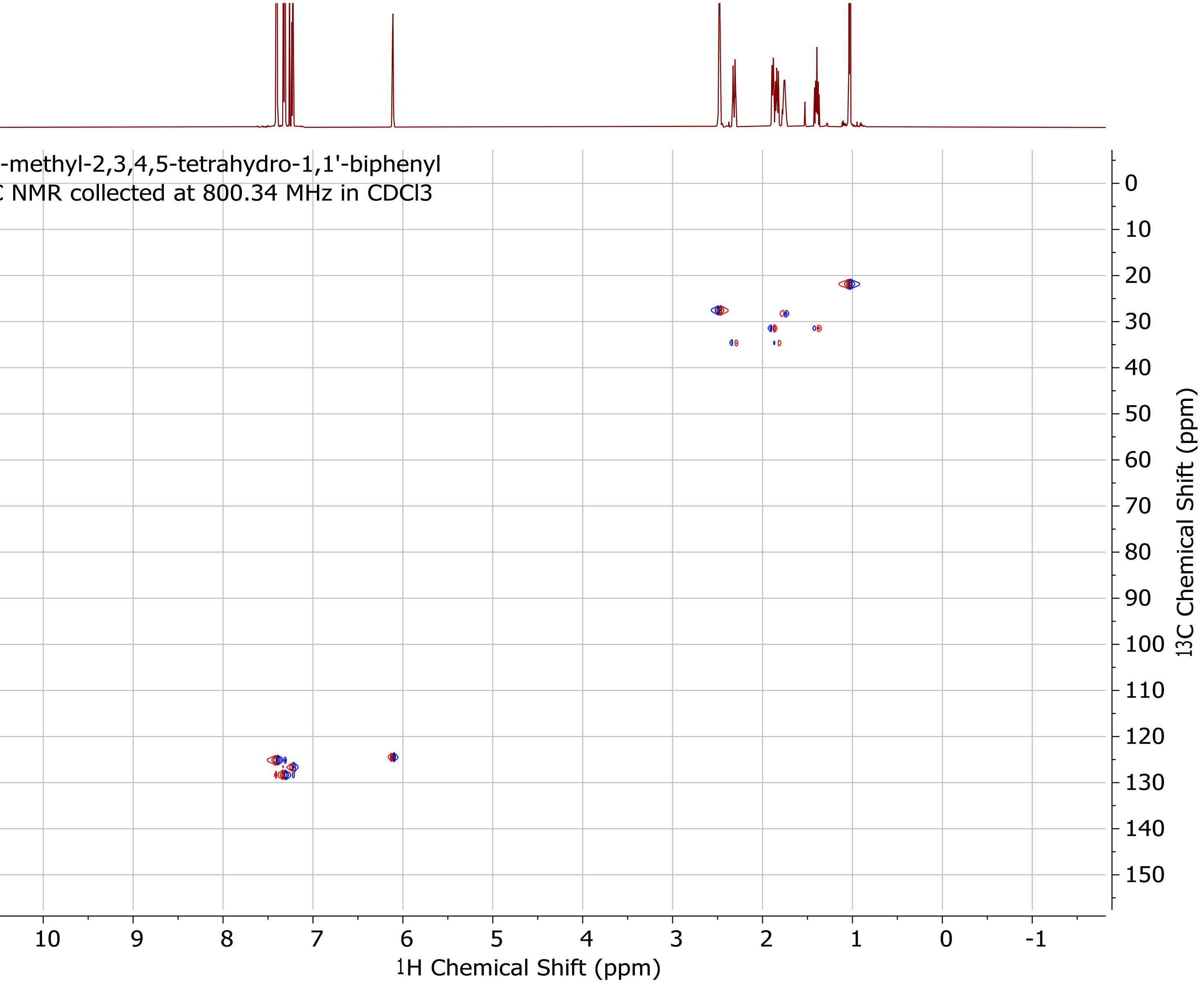

[1i] 4-methyl-2,3,4,5-tetrahydro-1,1'-biphenyl
HSQC NMR collected at 800.34 MHz in CDCl3
1H Chemical Shift (ppm)
13C Chemical Shift (ppm)

[1j] - 4-(tert-butyl)-2,3,4,5-tetrahydro-1,1'-biphenyl
1H NMR collected at 800.34 MHz in CDCl3

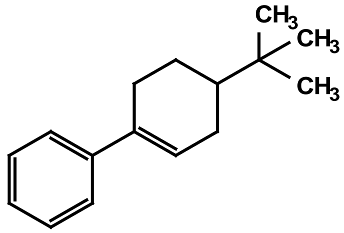

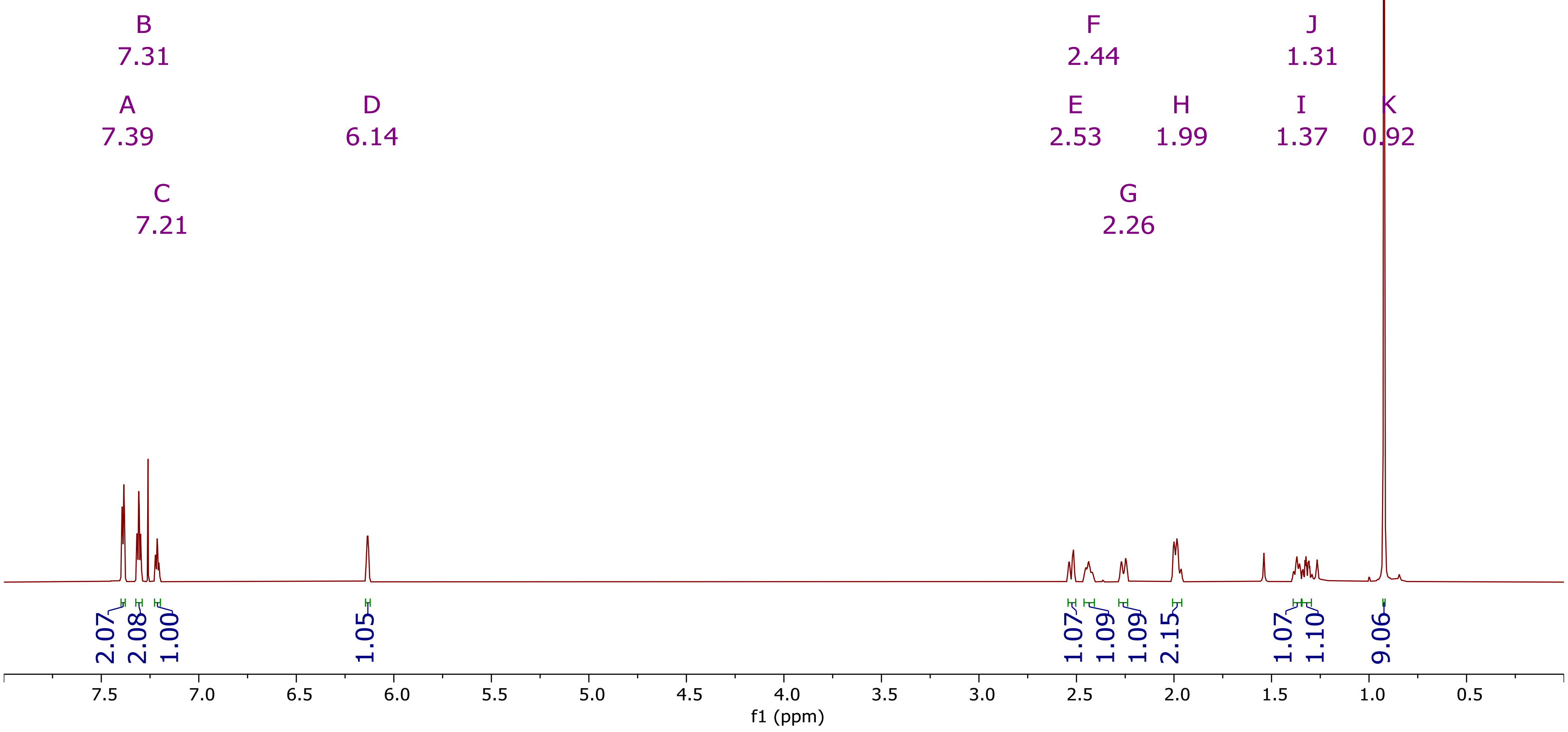

[1j] - 4-(tert-butyl)-2,3,4,5-tetrahydro-1,1'-biphenyl
13C NMR collected at 201.27 MHz in CDCl3

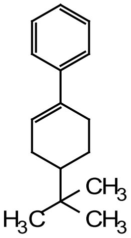

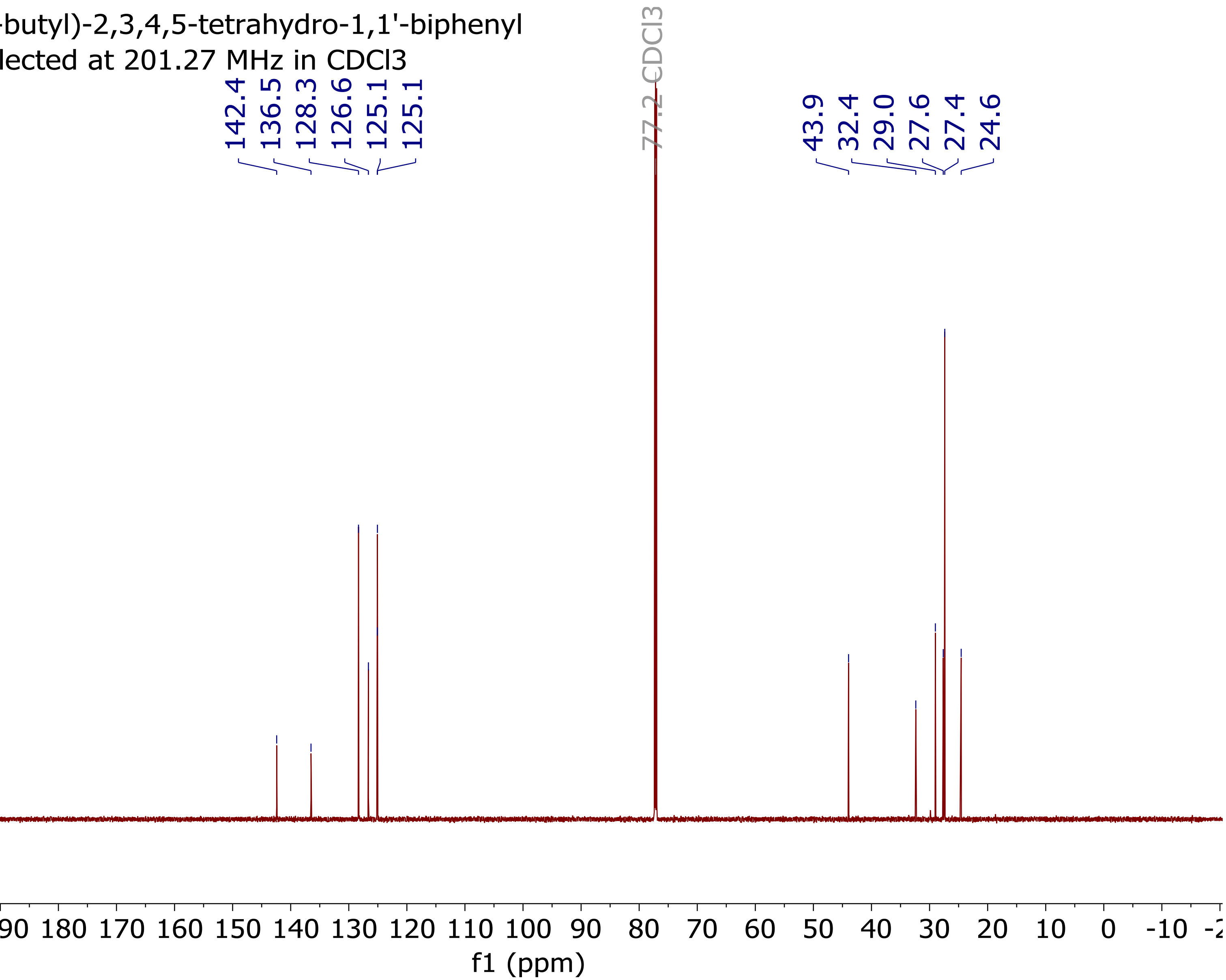

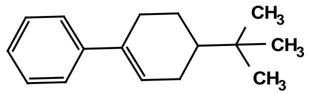
CH3
CH3
CH3

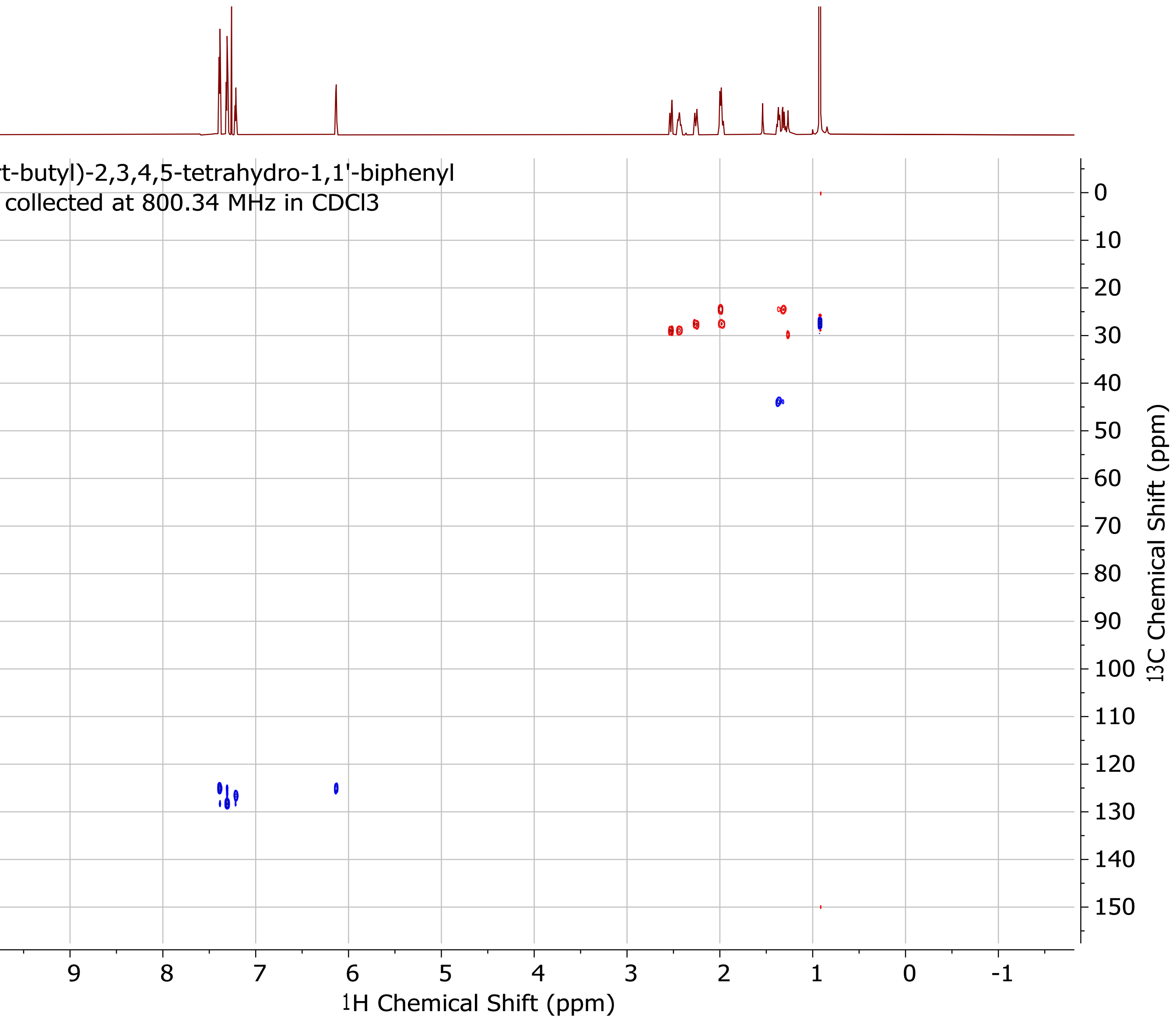
[1j] - 4-(tert-butyl)-2,3,4,5-tetrahydro-1,1'-biphenyl
HSQC NMR collected at 800.34 MHz in CDCl3
1H Chemical Shift (ppm)
13C Chemical Shift (ppm)

[1k] cis–3,5-dimethyl-2,3,4,5-tetrahydro-1,1'-biphenyl
1H NMR collected at 400.15 MHz in CDCl3

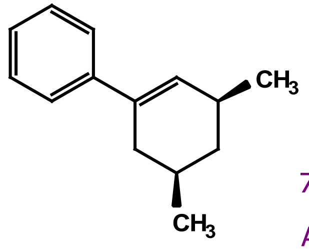

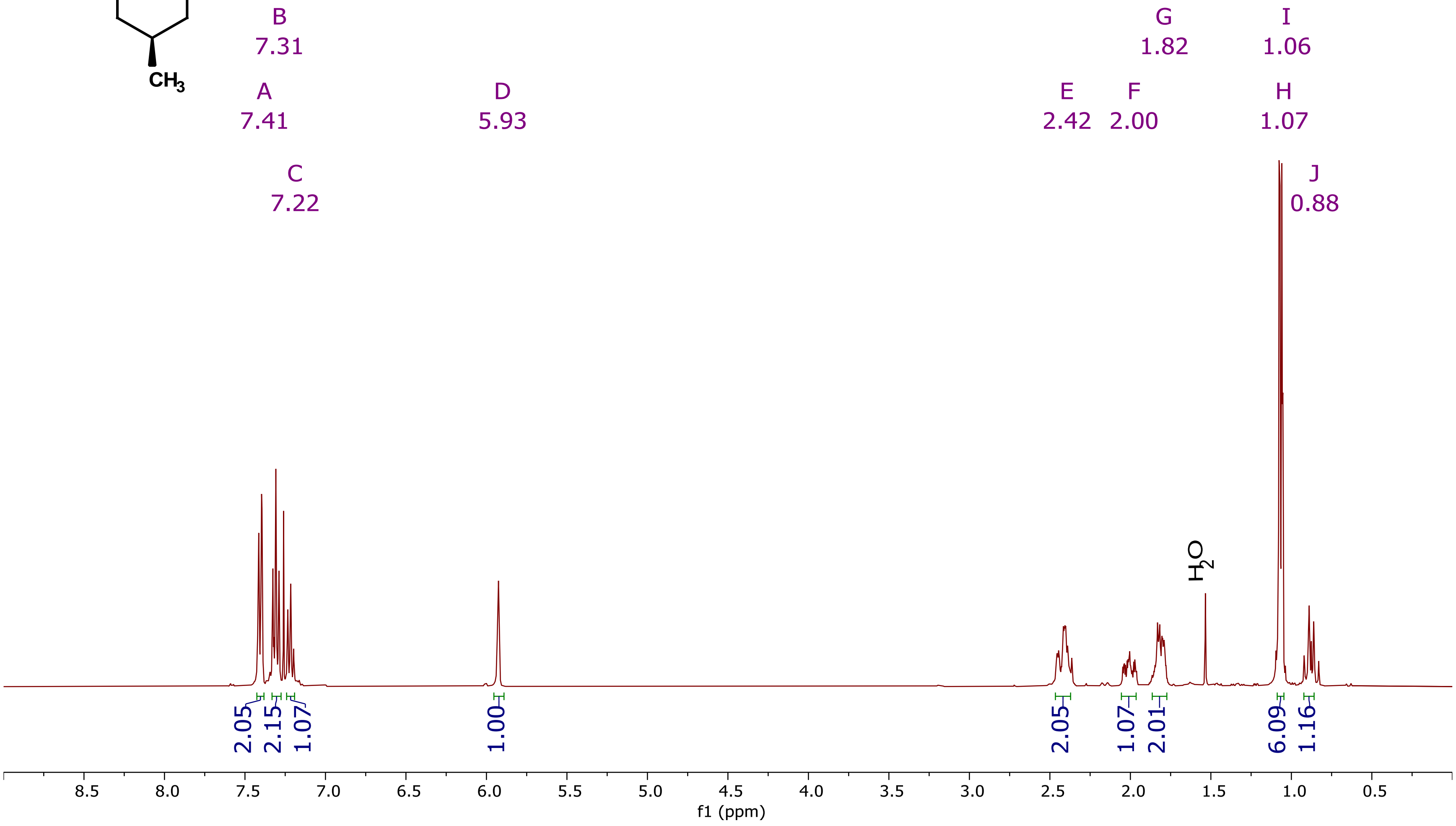

[1k] cis–3,5-dimethyl-2,3,4,5-tetrahydro-1,1'-biphenyl

13C NMR collected at 100.63 MHz in CDCl3

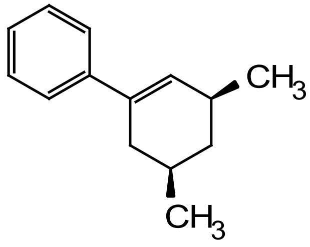

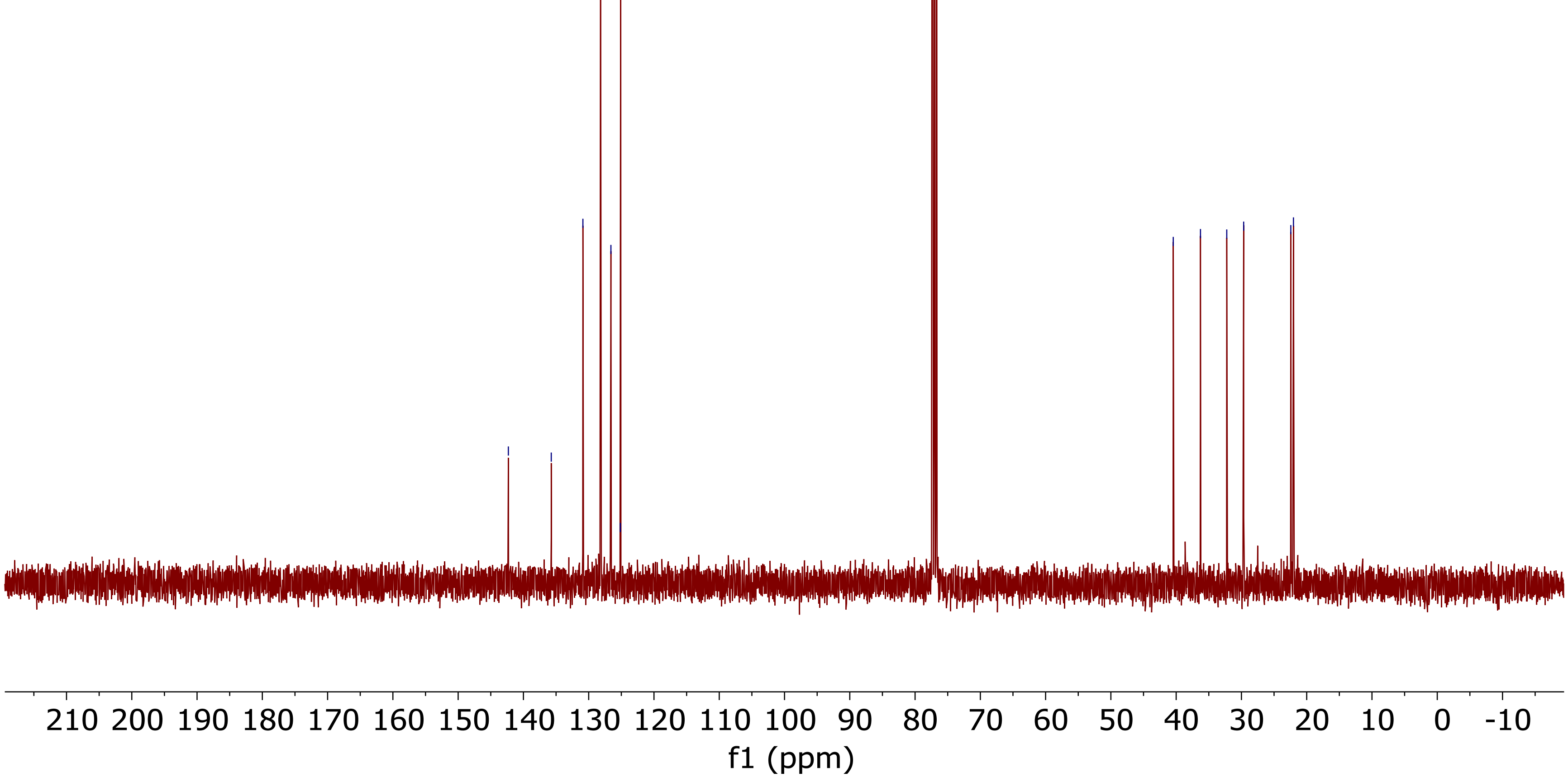

[1l] 4'-methoxy-2,5-dihydro-[1,1'-biphenyl]-4(3H)-one
1H NMR collected at 400.15 MHz in CDCl3

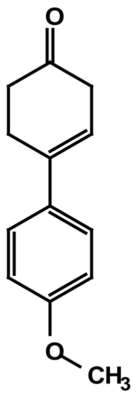

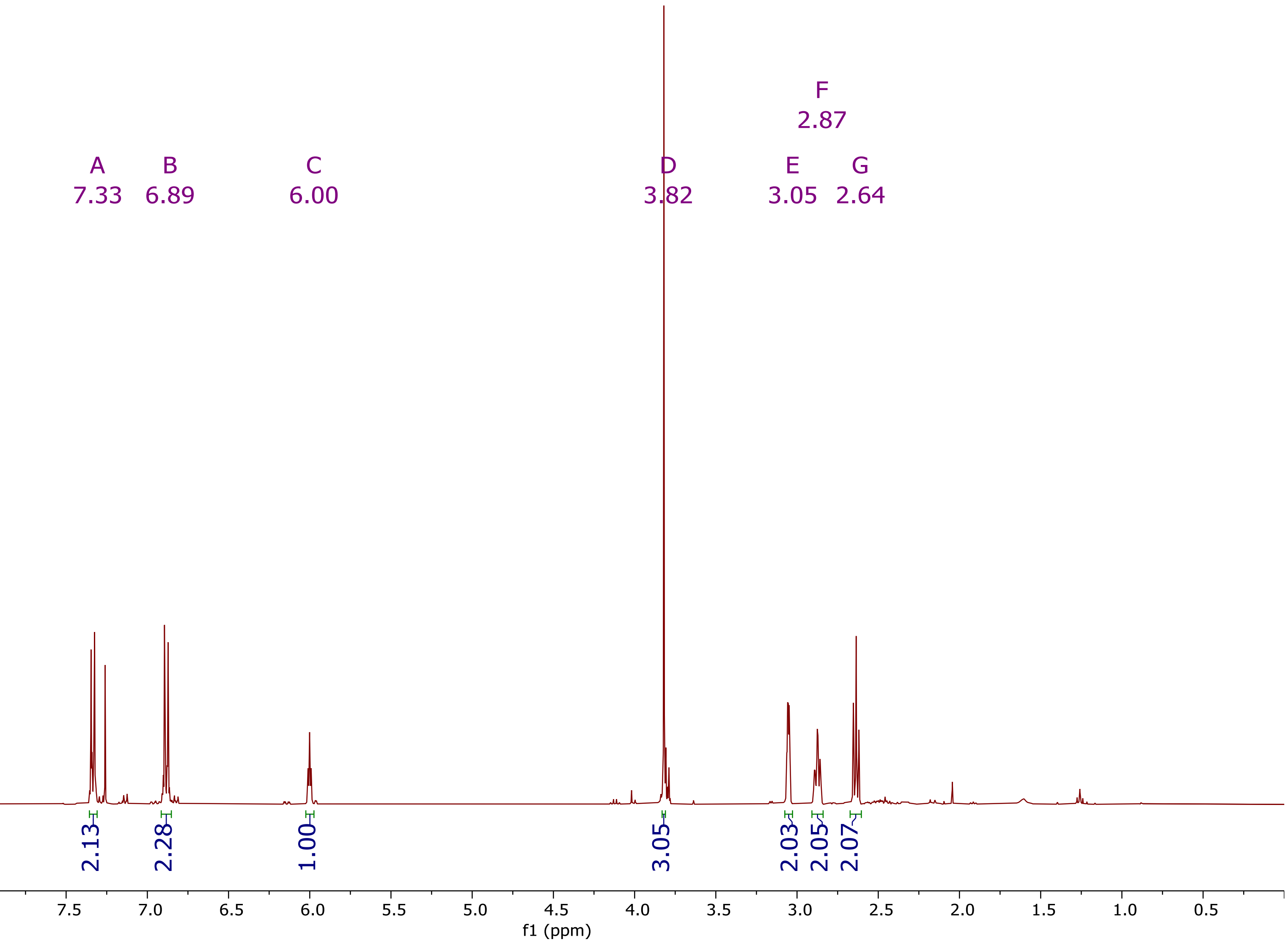

[1l] 4'-methoxy-2,5-dihydro-[1,1'-biphenyl]-4(3H)-one
13C NMR collected at 100.63 MHz in CDCl3

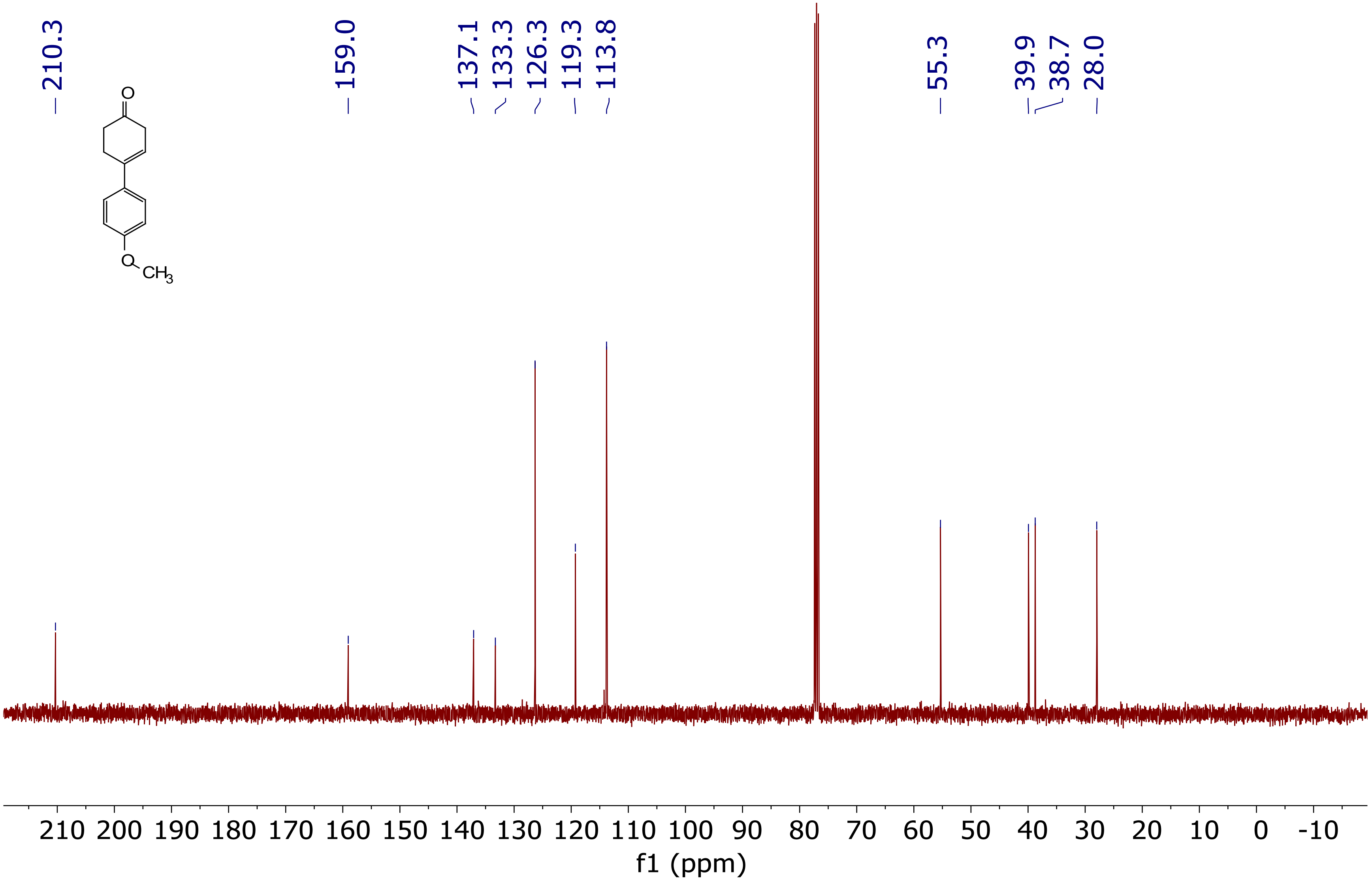

[1n] 4-phenyl-1-tosyl-1,2,3,6-tetrahydropyridine
1H NMR collected at 800.34 MHz in CDCl3

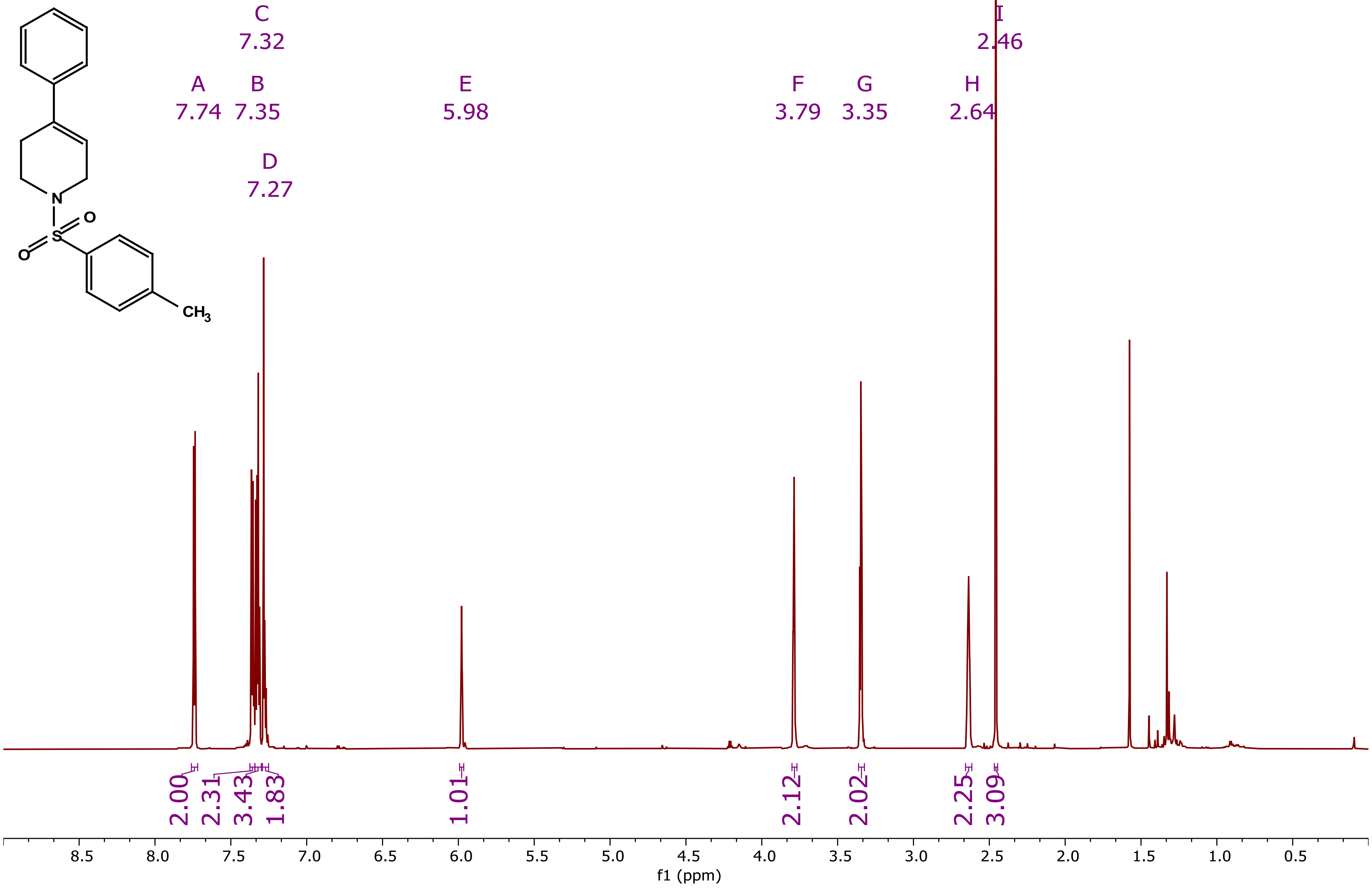

[1n] 4-phenyl-1-tosyl-1,2,3,6-tetrahydropyridine
13C NMR collected at 201.27 MHz in CDCl3

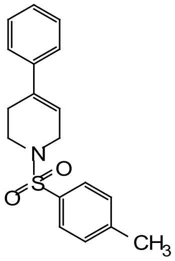

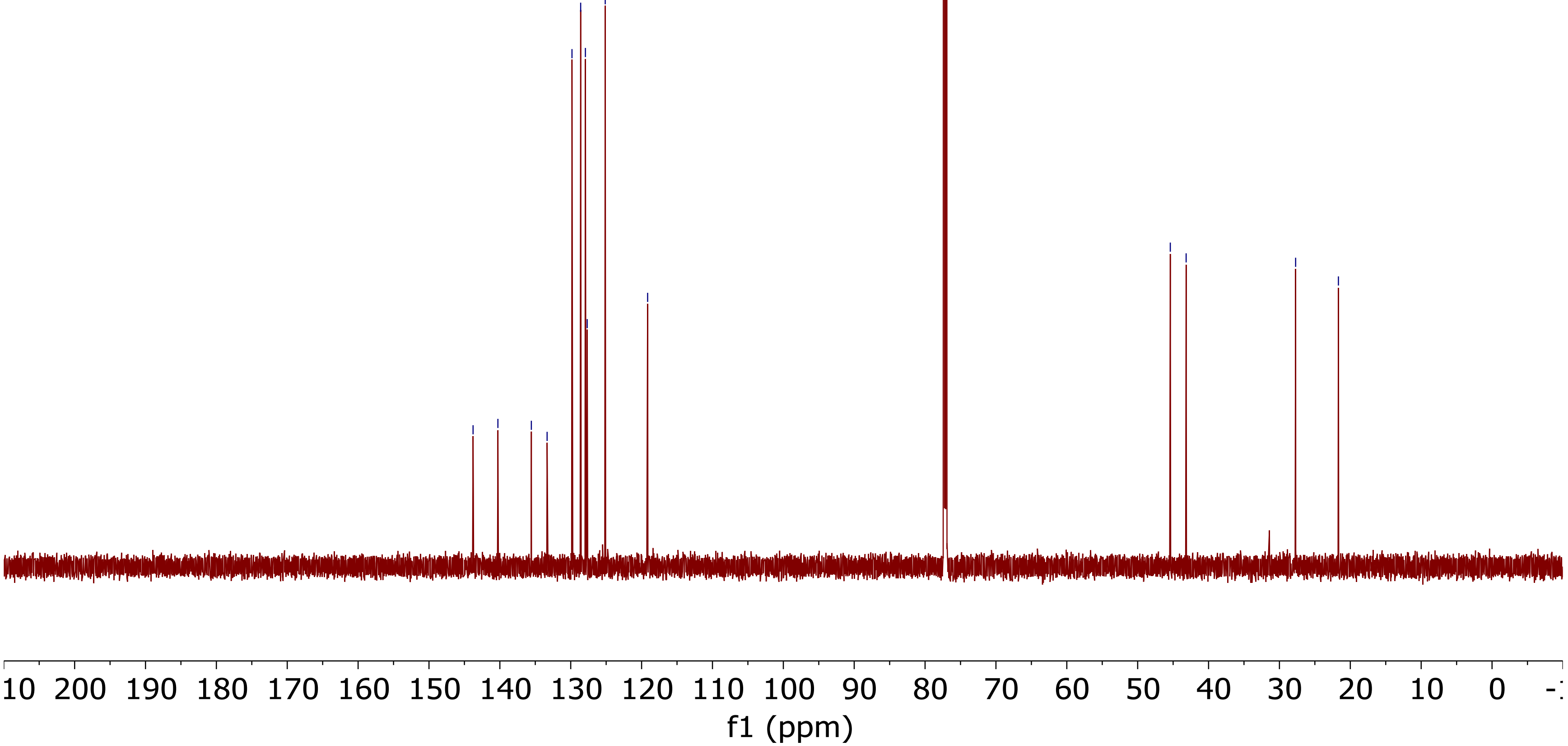

[1o] 1-(4-phenyl-3,6-dihydropyridin-1(2H)-yl)ethan-1-one
1H NMR collected at 800.34 MHz in CDCl3

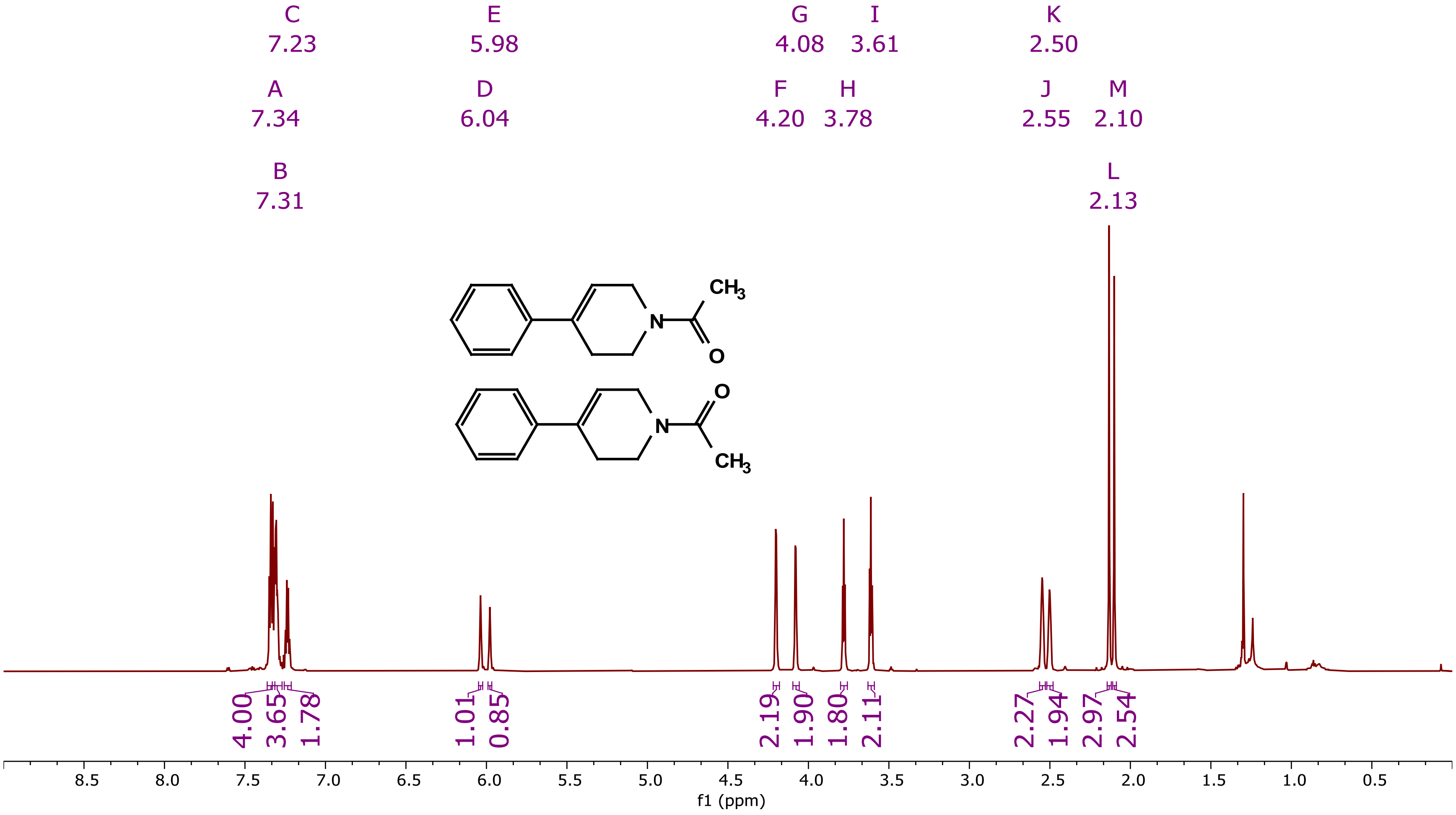

[1o] 1-(4-phenyl-3,6-dihydropyridin-1(2H)-yl)ethan-1-one
13C NMR collected at 201.27 MHz in CDCl3

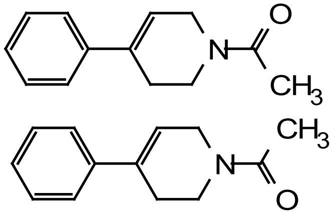

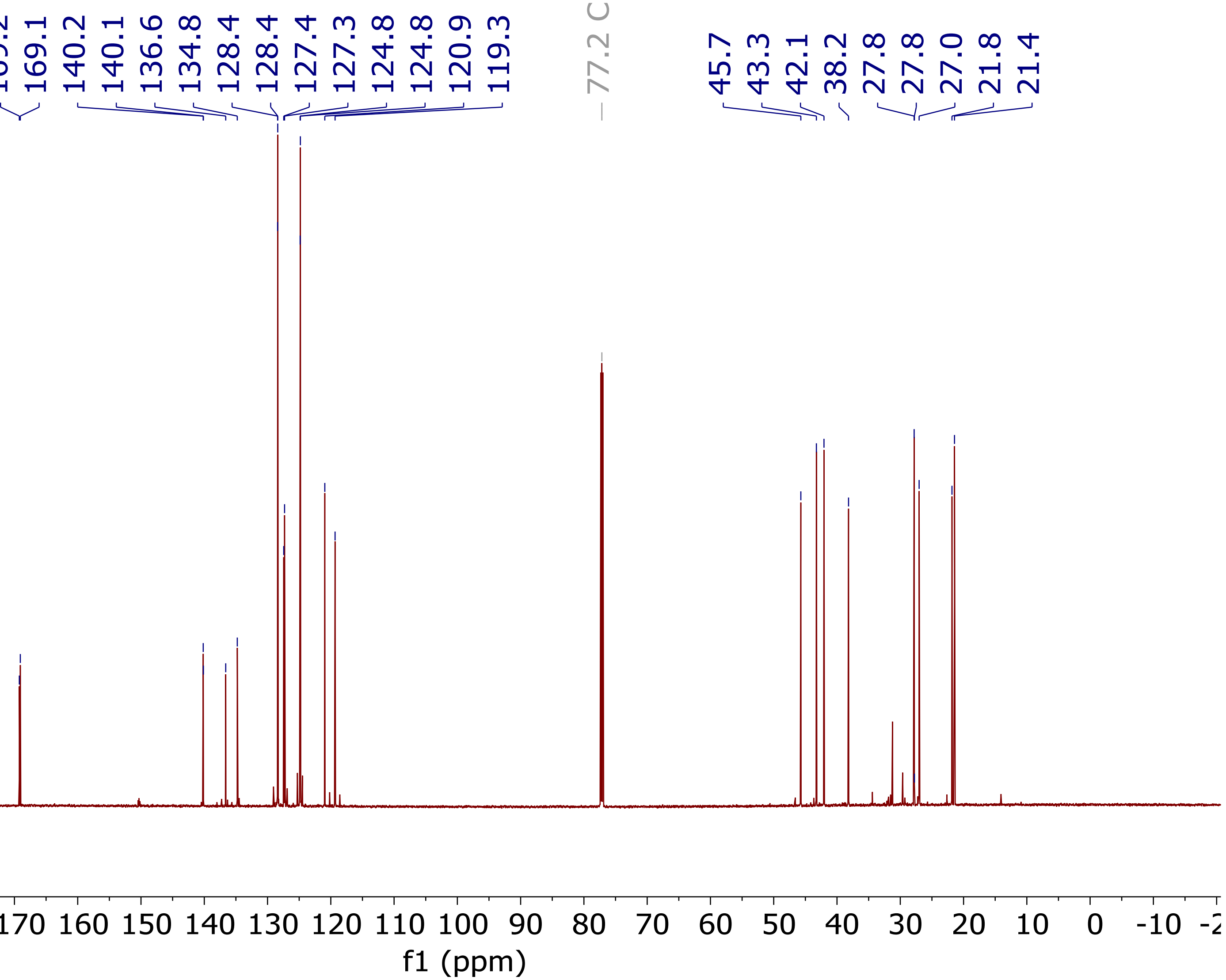

[1p] 4-phenyl-1,2,3,6-tetrahydropyridine
1H NMR collected at 800.34 MHz in CDCl3

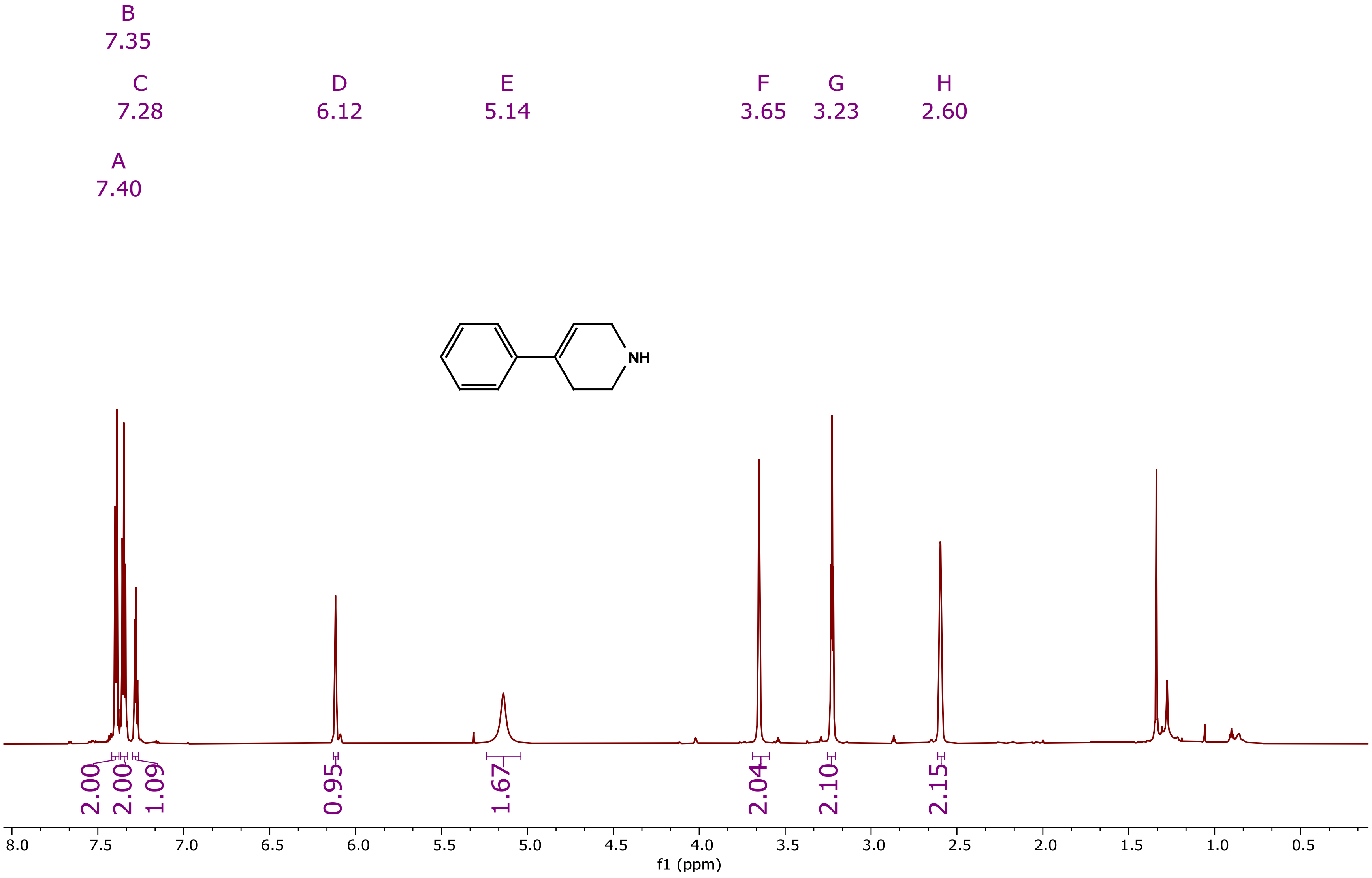

[1p] 4-phenyl-1,2,3,6-tetrahydropyridine
13C NMR collected at 201.27 MHz in CDCl3

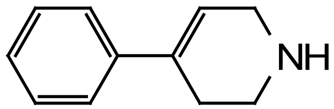

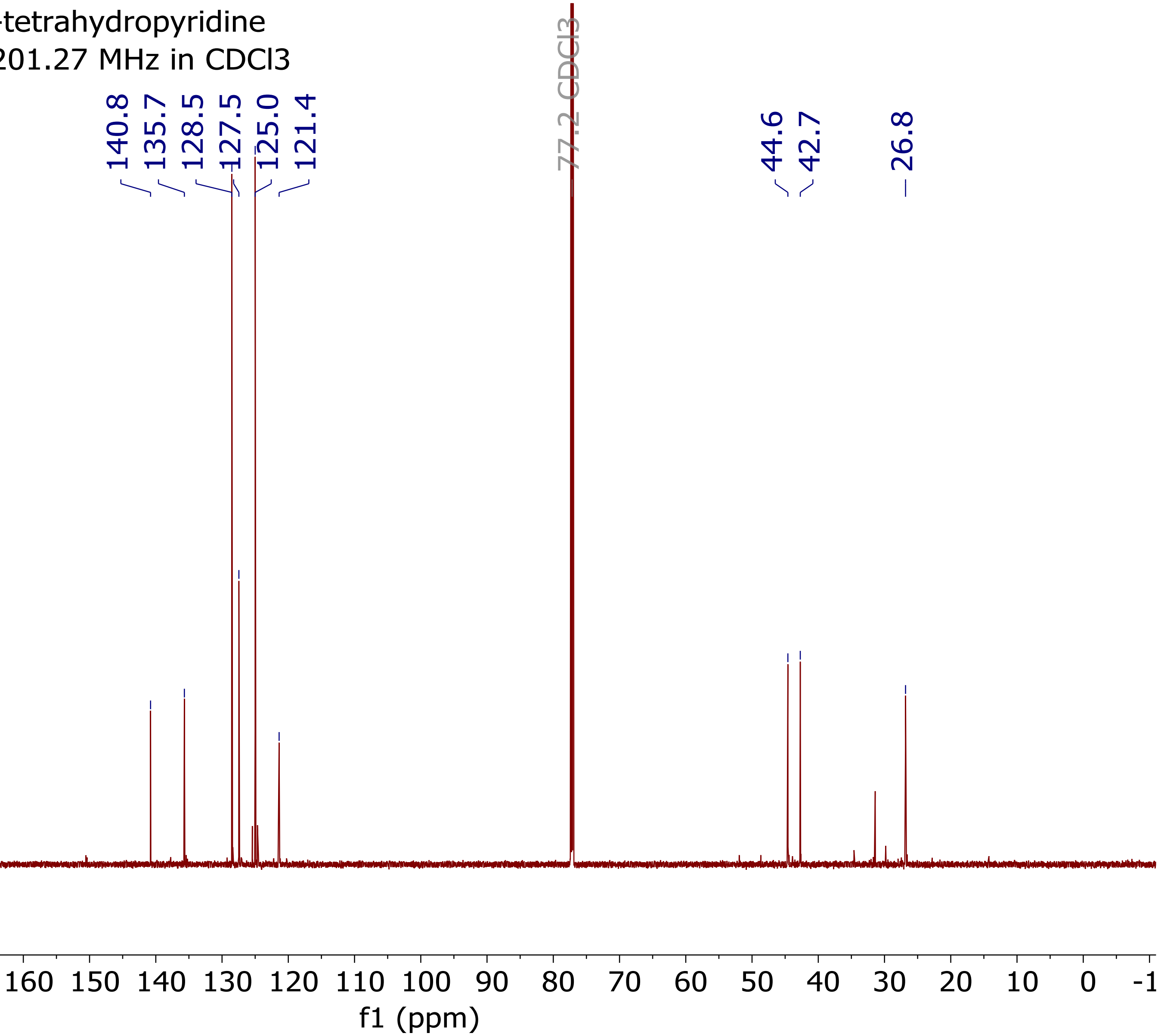

[1q] 1-(4-methoxybenzyl)-4-phenyl-1,2,3,6-tetrahydropyridine
1H NMR collected at 800.34 MHz in CDCl3

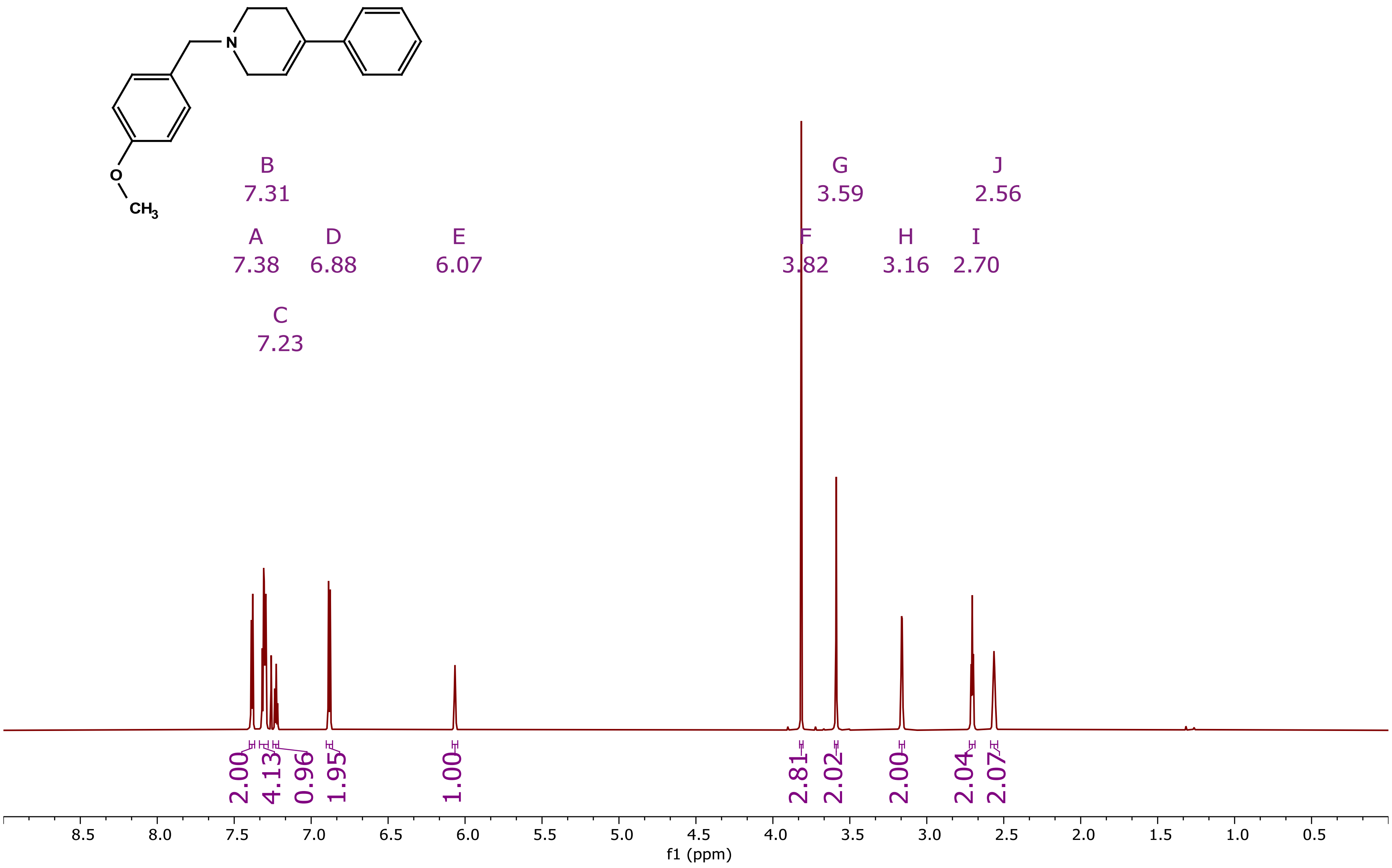

[1q] 1-(4-methoxybenzyl)-4-phenyl-1,2,3,6-tetrahydropyridine
13C NMR collected at 201.27 MHz in CDCl3

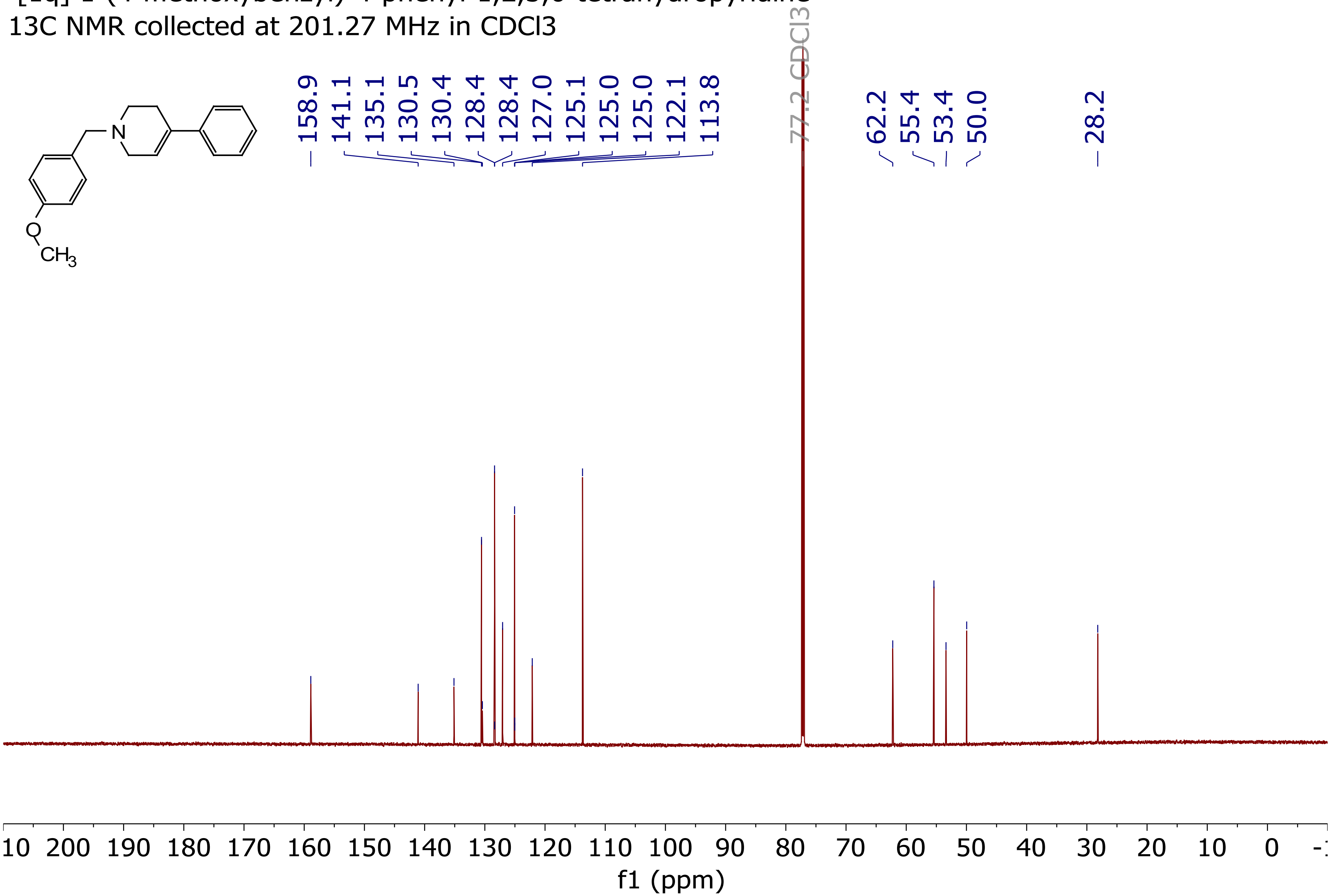

[1r] tert-butyl 4-(4-(trifluoromethyl)phenyl)-3,6-dihydropyridine-1(2H)-carboxylate
1H NMR collected at 800.34 MHz in CDCl3

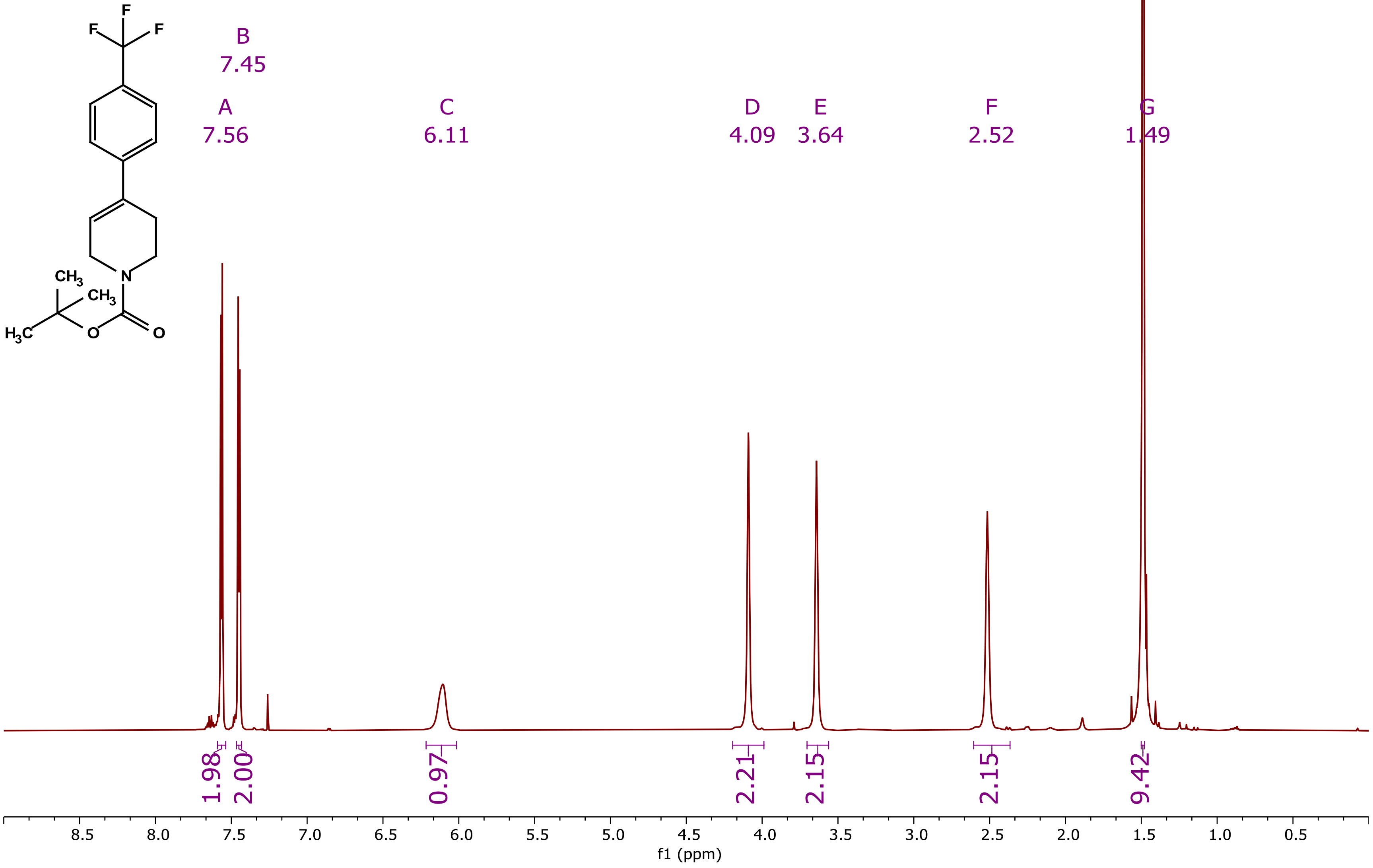

[1r] tert-butyl 4-(4-(trifluoromethyl)phenyl)-3,6-dihydropyridine-1(2H)-carboxylate
13C NMR collected at 201.27 MHz in CDCl3

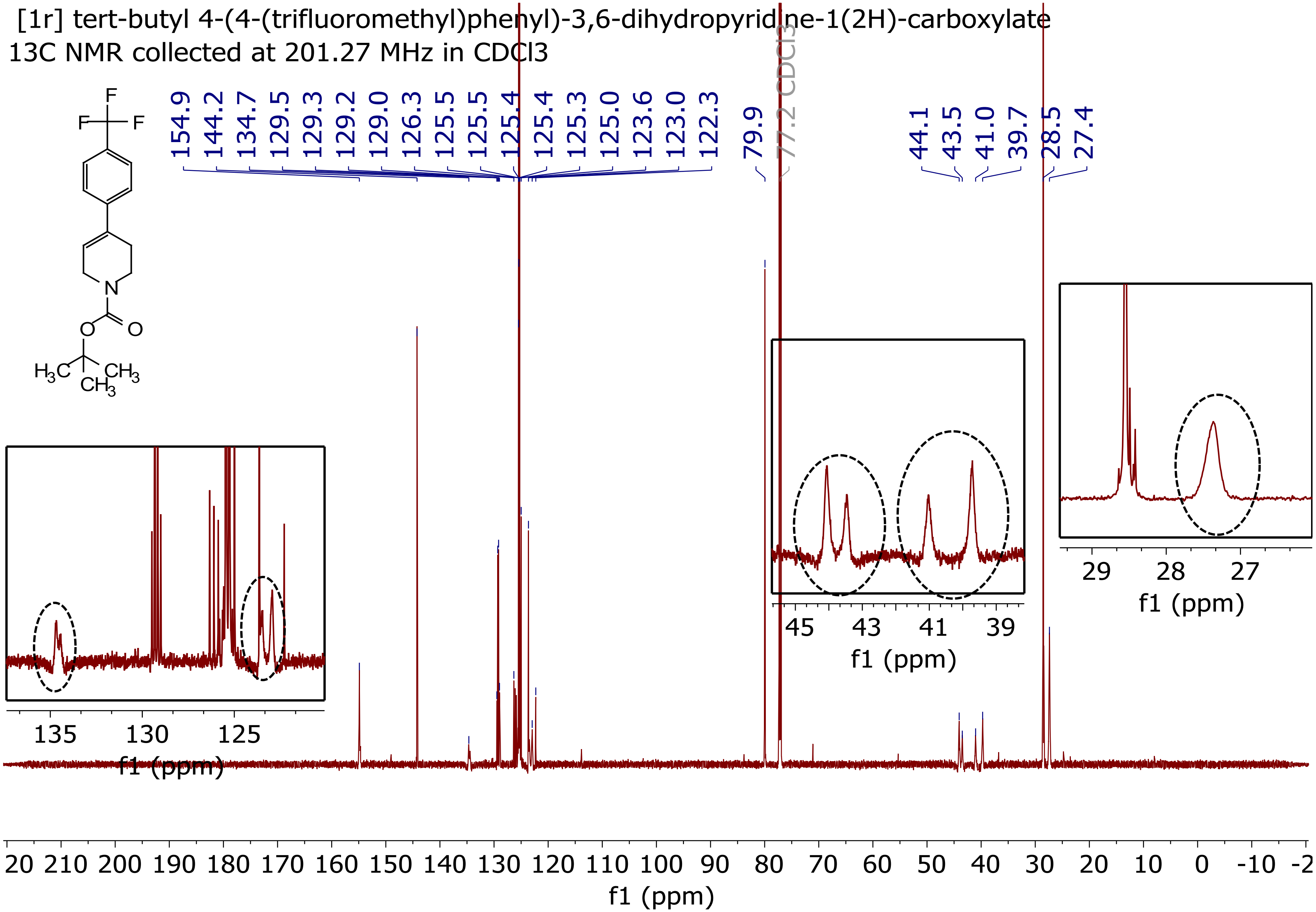

[1r] tert-butyl 4-(4-(trifluoromethyl)phenyl)-3,6-dihydropyridine-1(2H)-carboxylate
19F NMR collected at 753.00 MHz in CDCl3

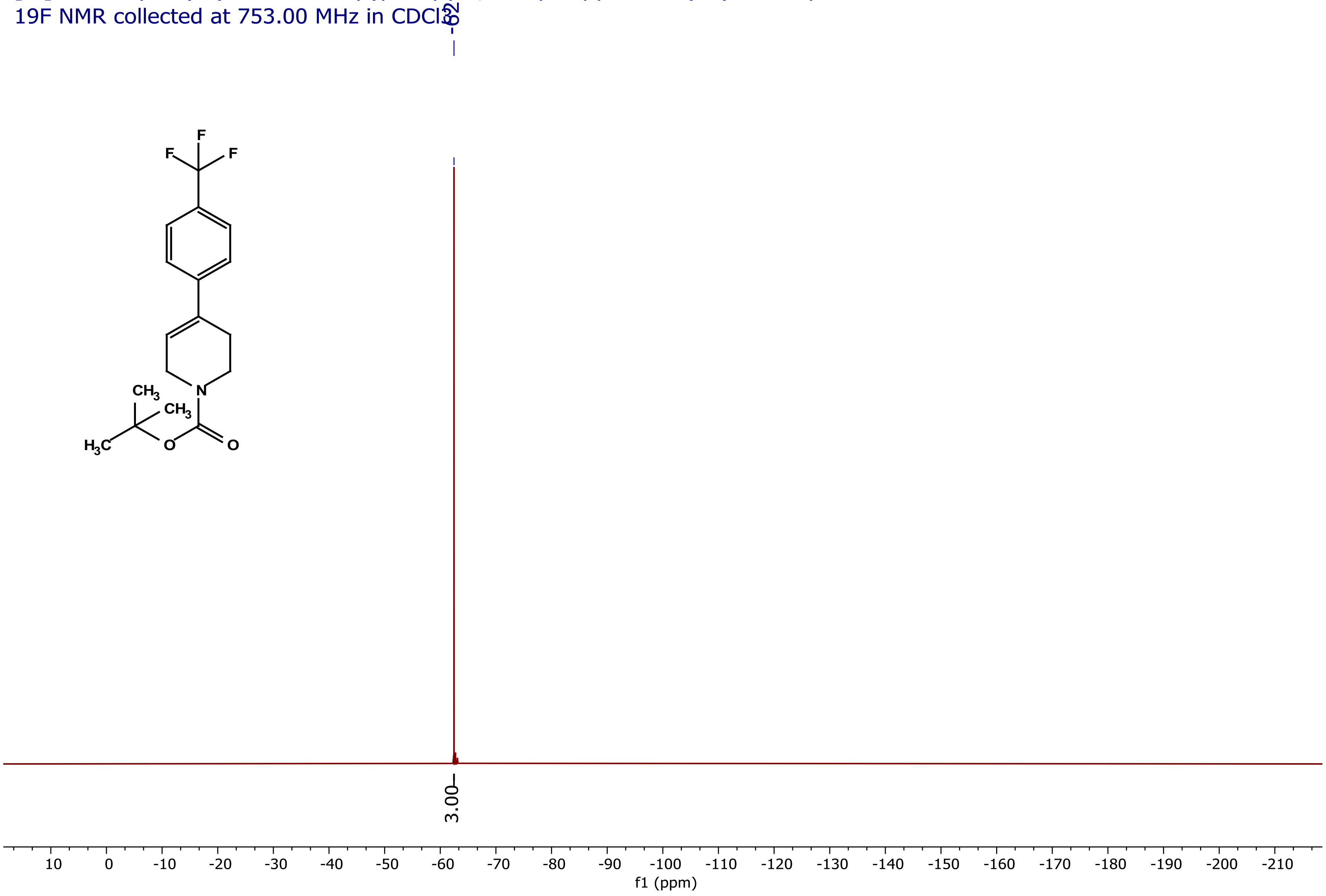

[1s]- tert-butyl 4-phenyl-3,6-dihydropyridine-1(2H)-carboxylate
1H NMR collected at 800.34 MHz in CDCl3

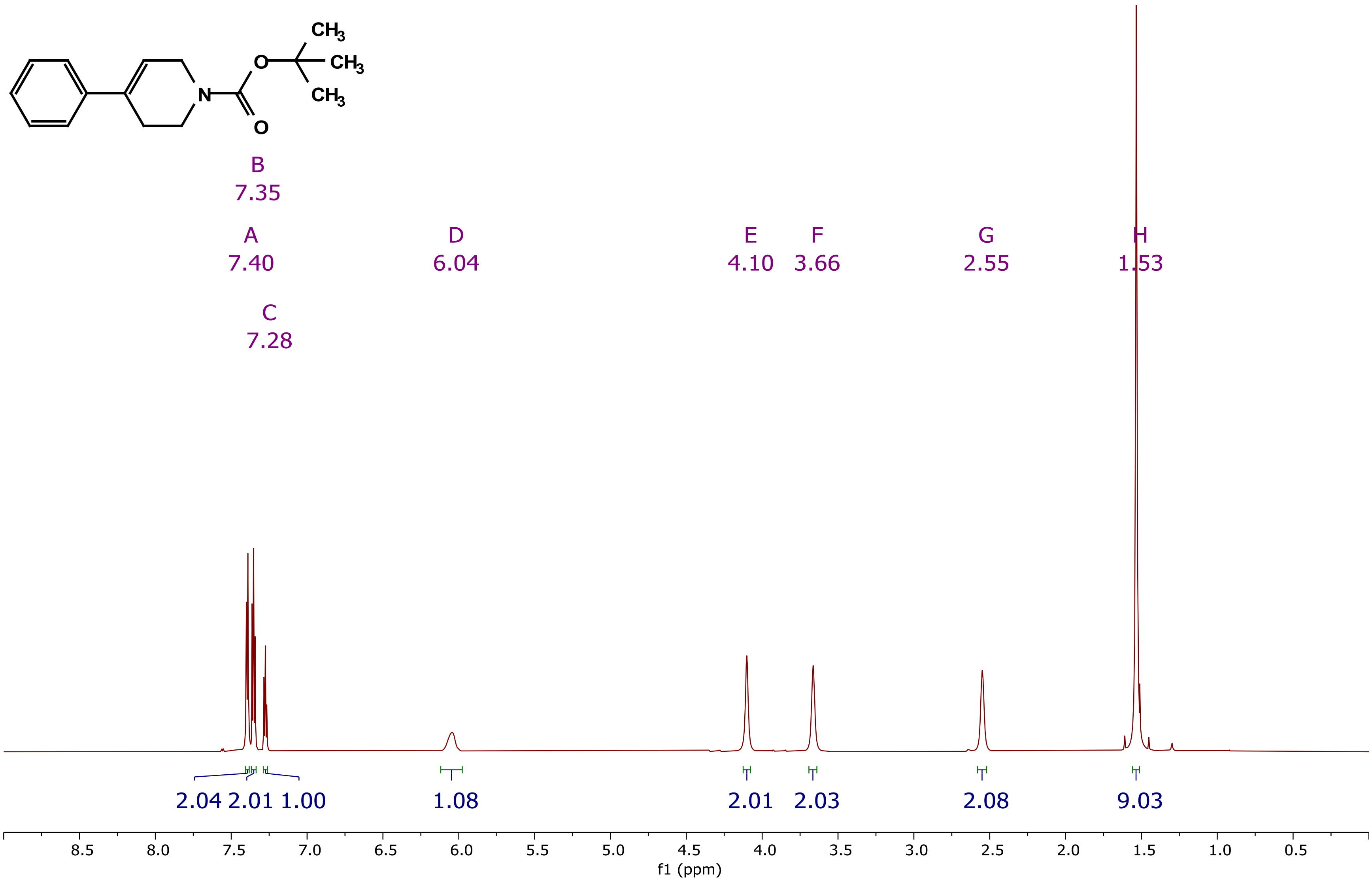

[1s]- tert-butyl 4-phenyl-3,6-dihydropyridine-1(2H)-carboxylate
13C NMR collected at 201.27 MHz in CDCl3

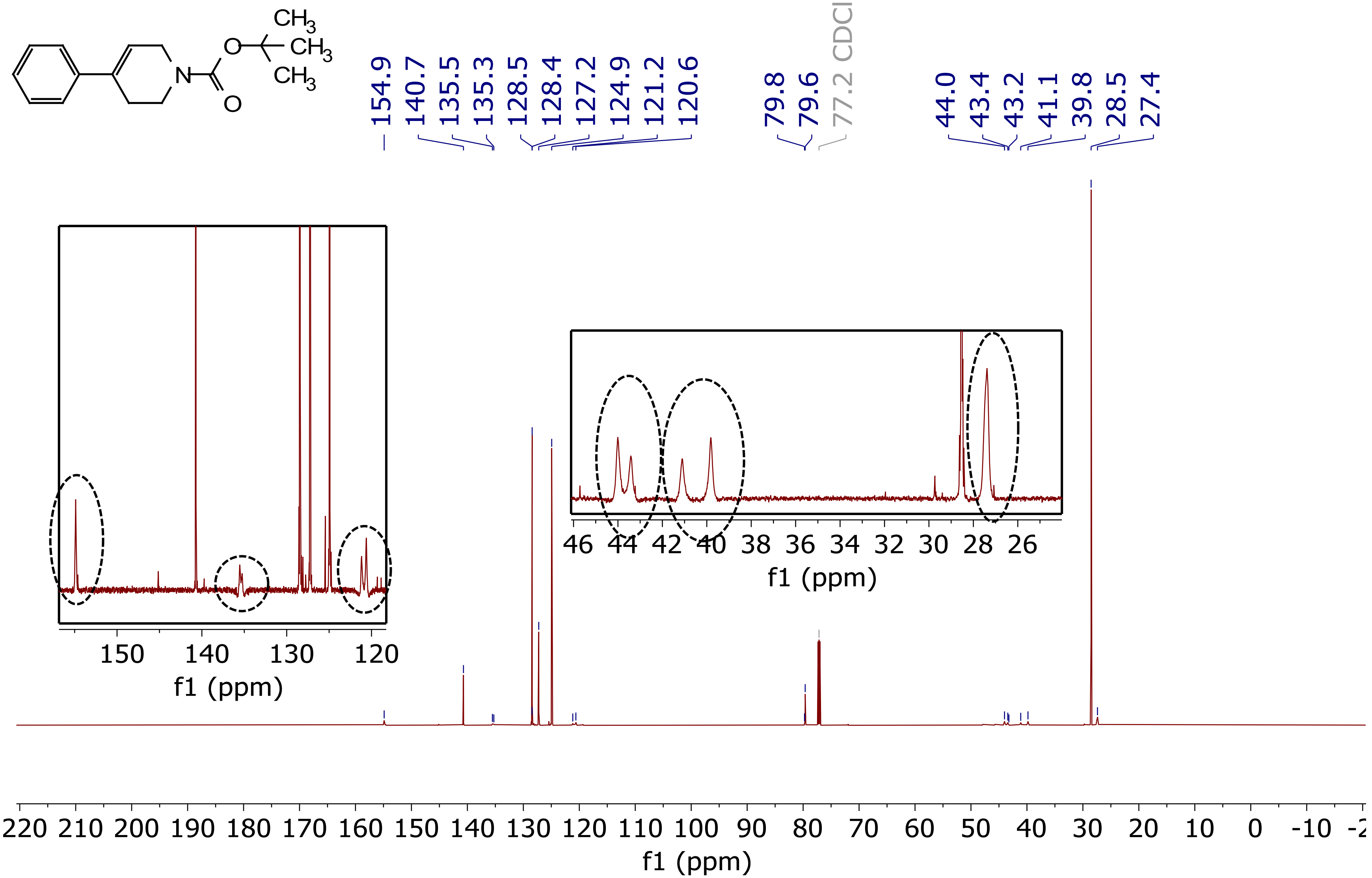

[1t] tert-butyl 4-(4-methoxyphenyl)-3,6-dihydropyridine-1(2H)-carboxylate
1H NMR collected at 800.34 MHz in CDCl3

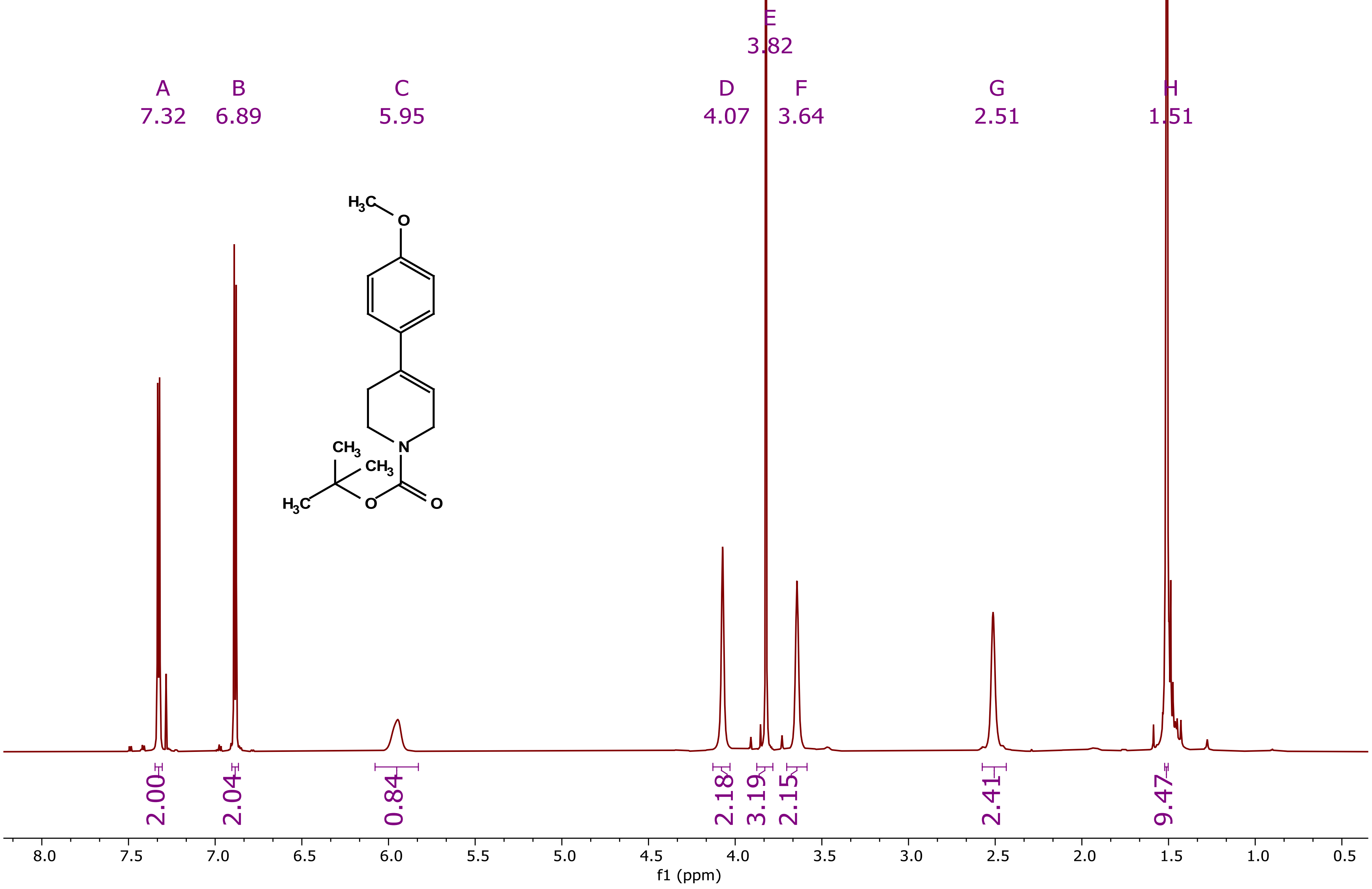

[1t] tert-butyl 4-(4-methoxyphenyl)-3,6-dihydropyridine-1(2H)-carboxylate
13C NMR collected at 201.27 MHz in CDCl3

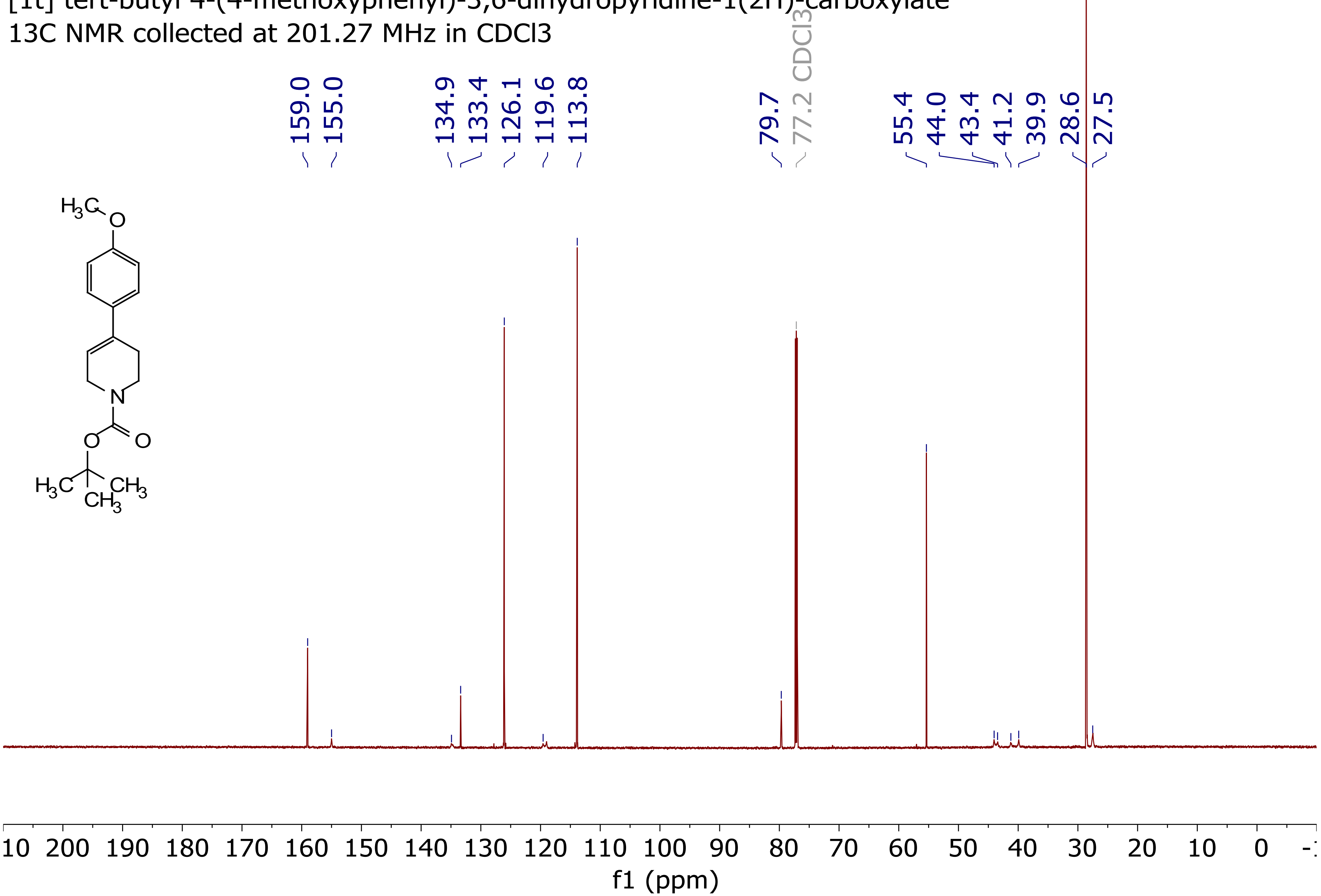

[1u] N-(2,3,4,5-tetrahydro-[1,1'-biphenyl]-4-yl)acetamide
1H NMR collected at 800.34 MHz in CDCl3

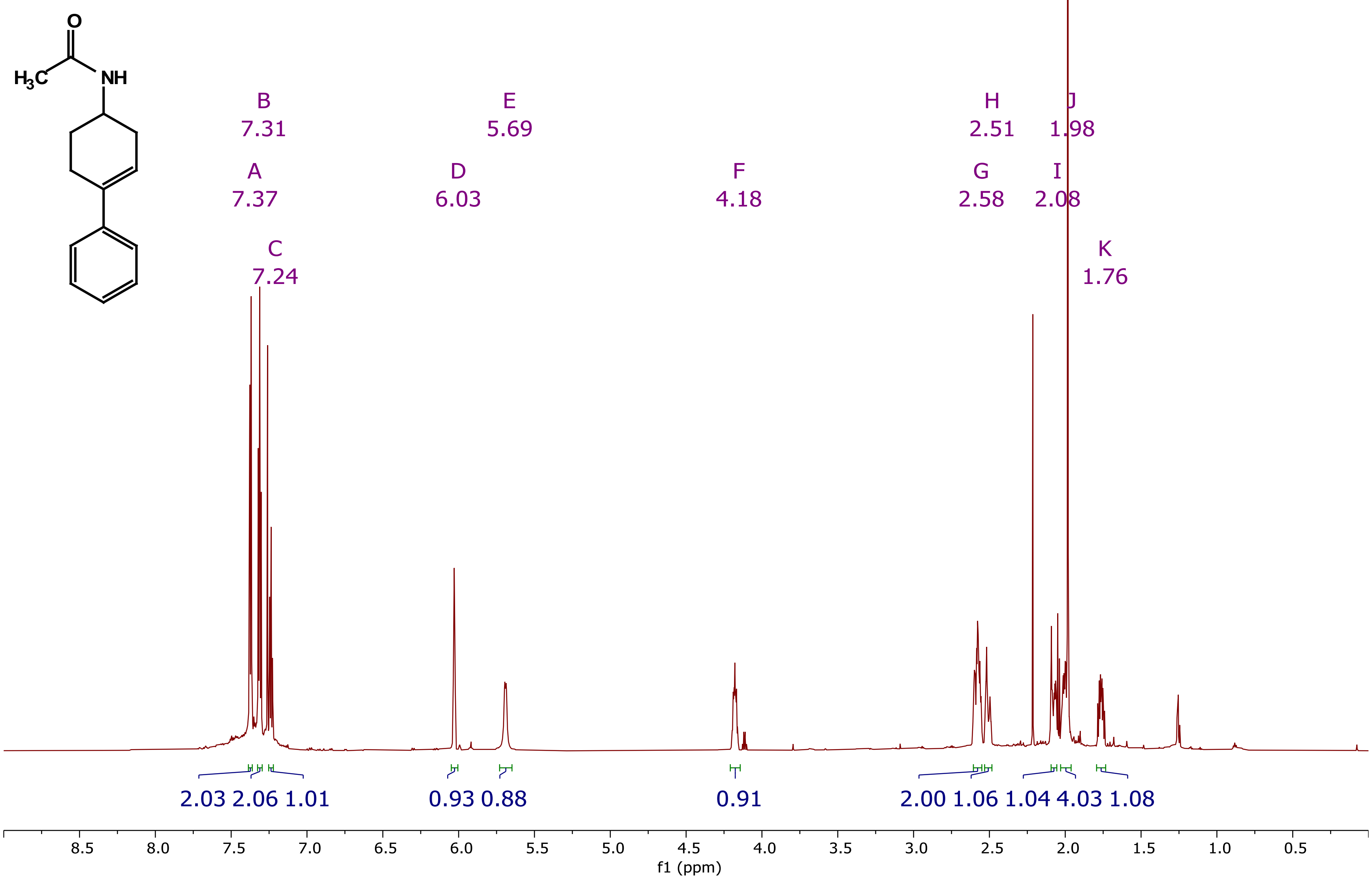

[1u] N-(2,3,4,5-tetrahydro-[1,1'-biphenyl]-4-yl)acetamide
13C NMR collected at 201.27 MHz in CDCl3

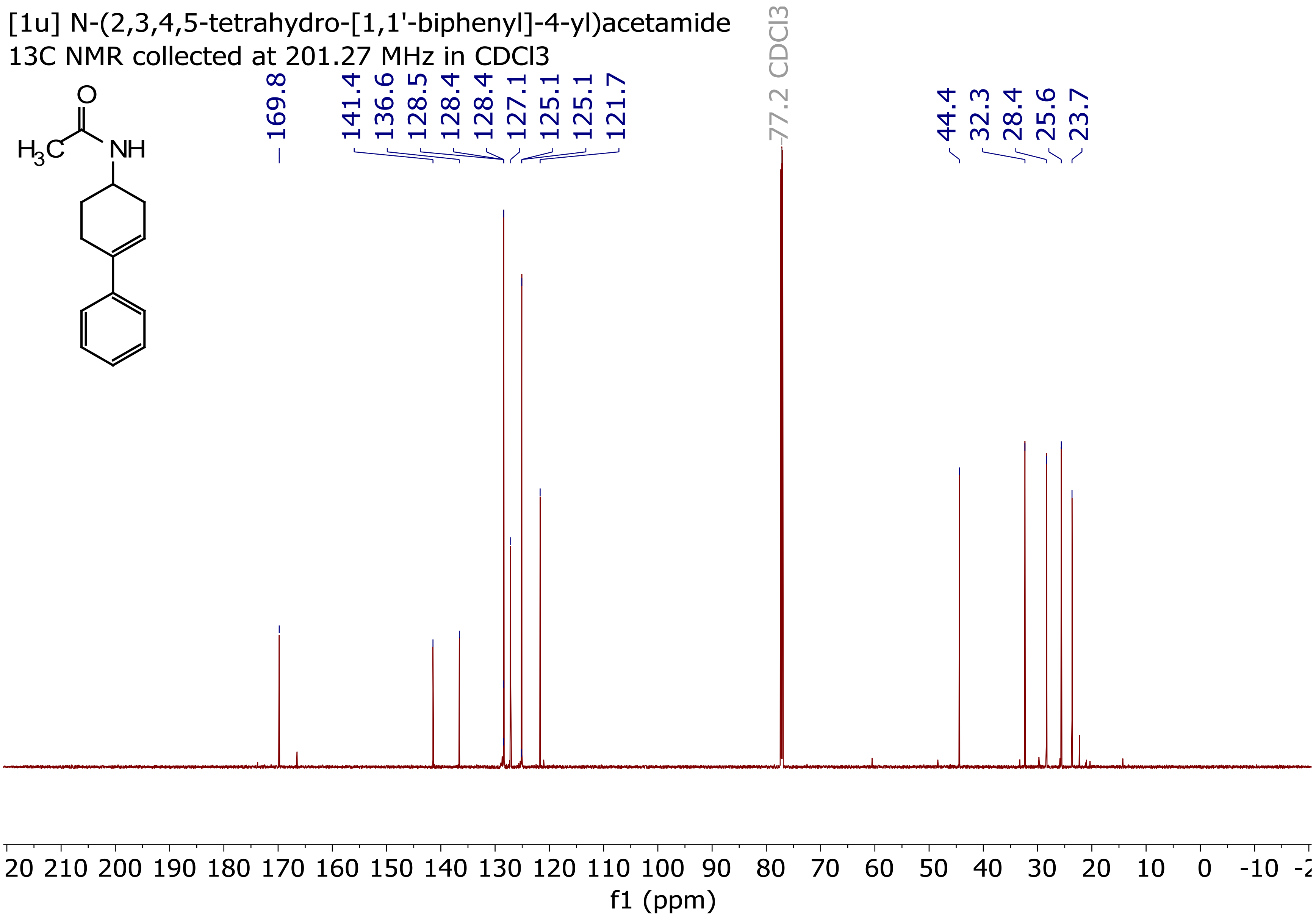

[1v] 1-phenylcyclohept-1-ene

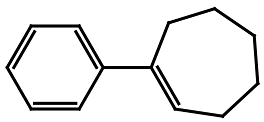

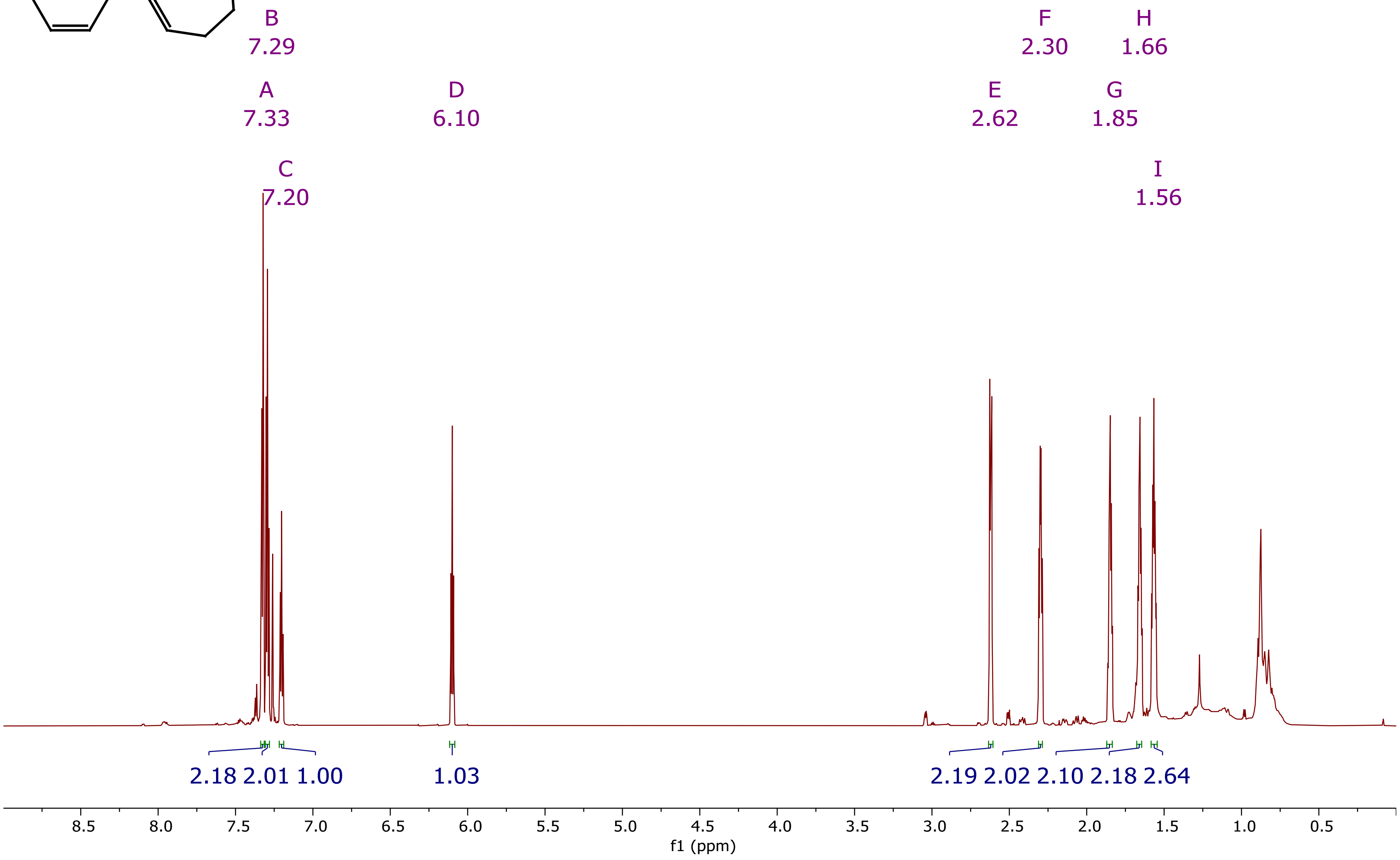

[1v] 1-phenylcyclohept-1-ene
13C NMR collected at 201.27 MHz in CDCl3

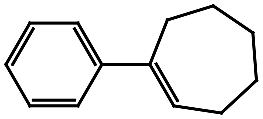

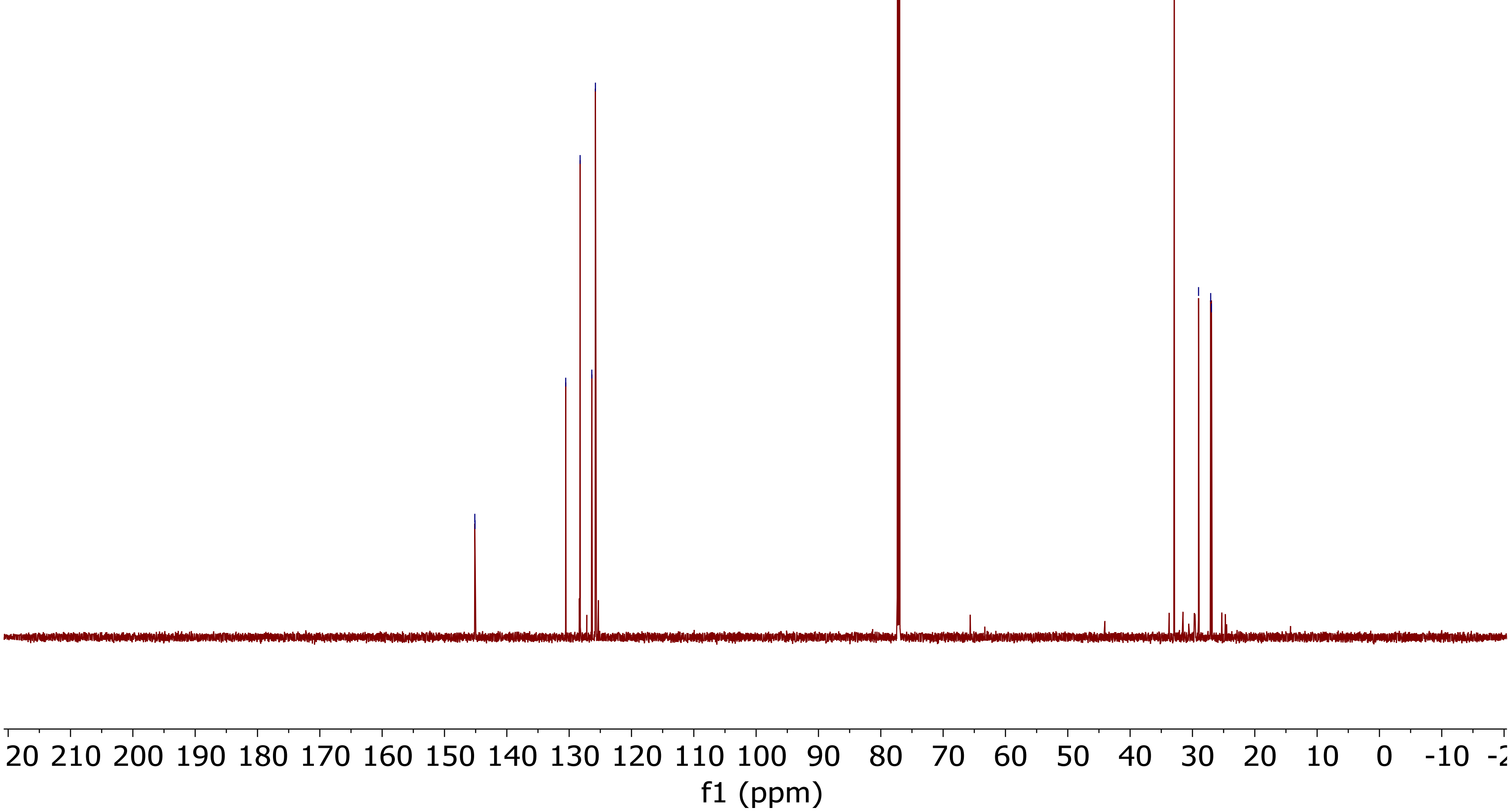

2,4,6-trimethylpyridinium hexafluorophosphate

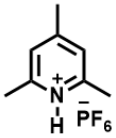

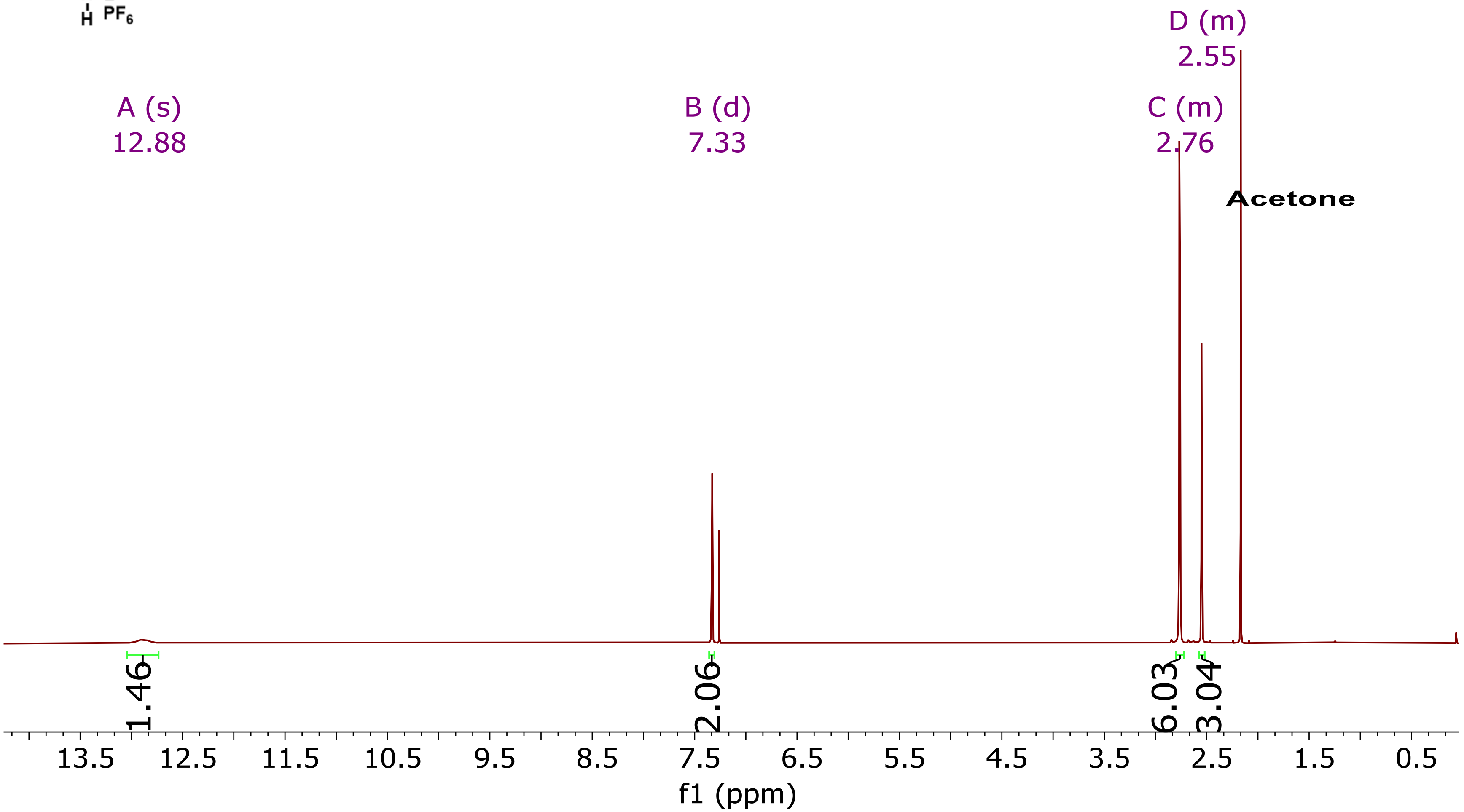

2,4,6-trimethylpyridinium hexafluorophosphate

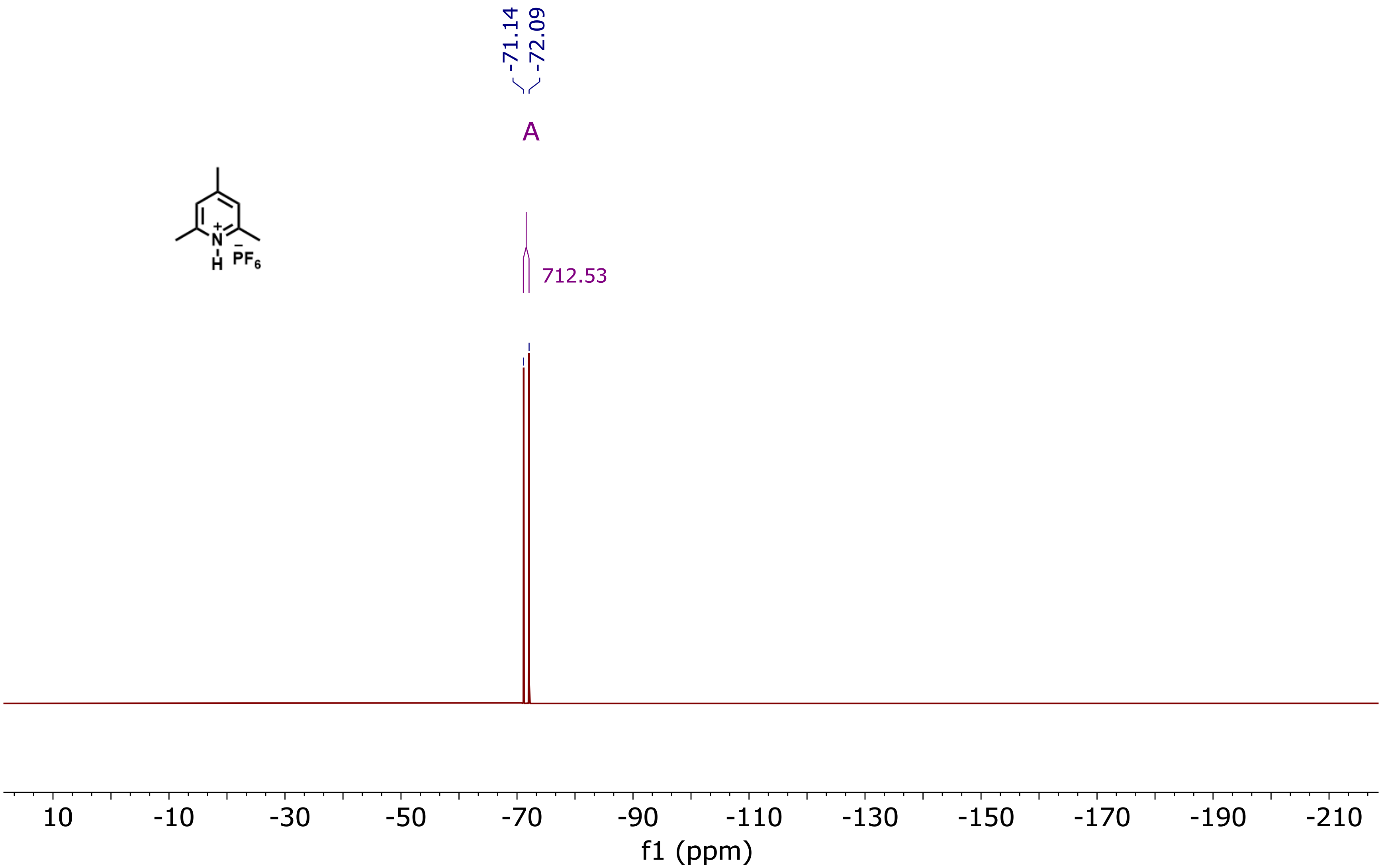

2,4,6-trimethylpyridinium triflate

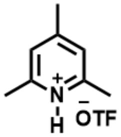

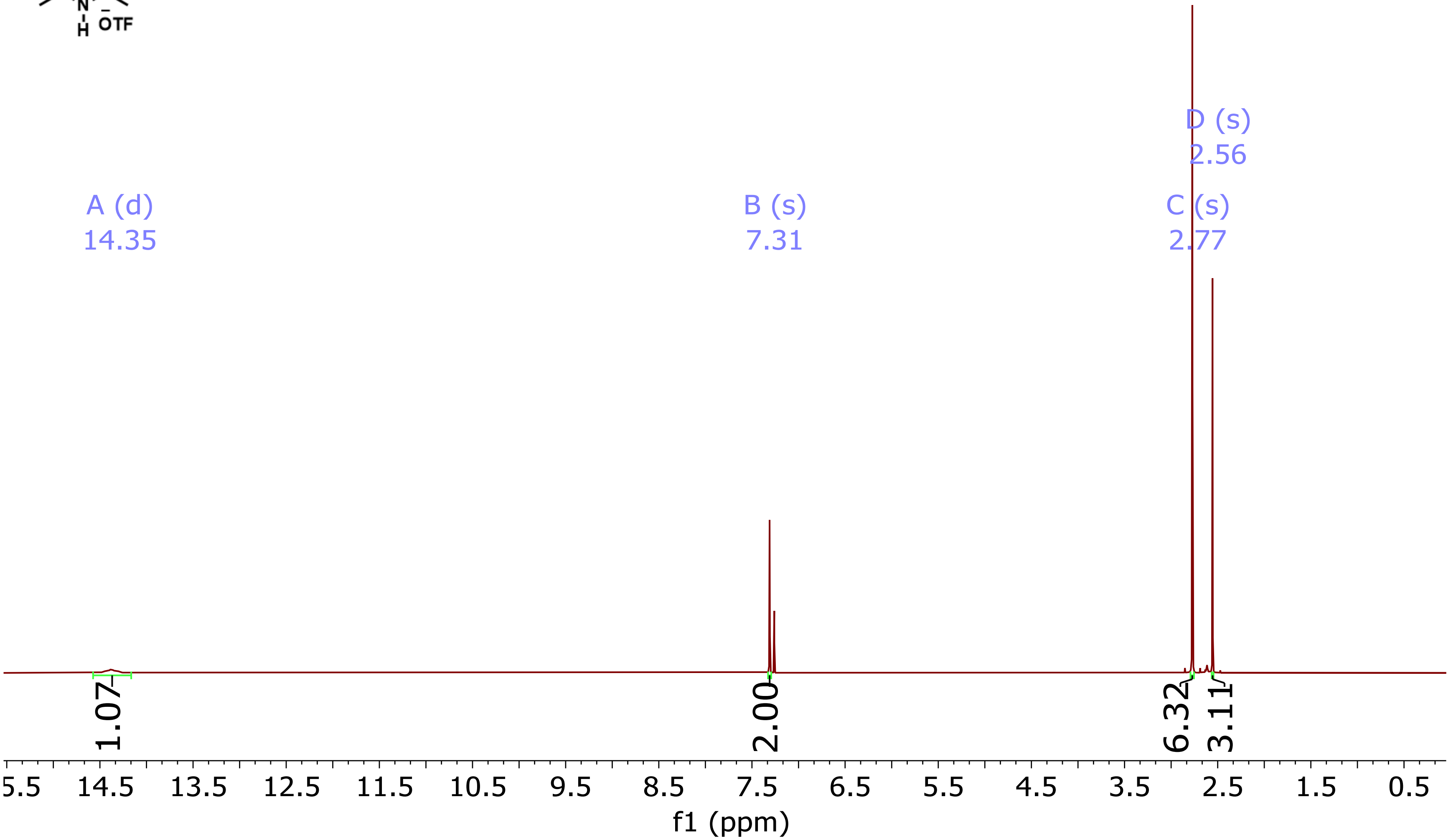

2,4,6-trimethylpyridinium triflate

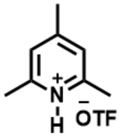

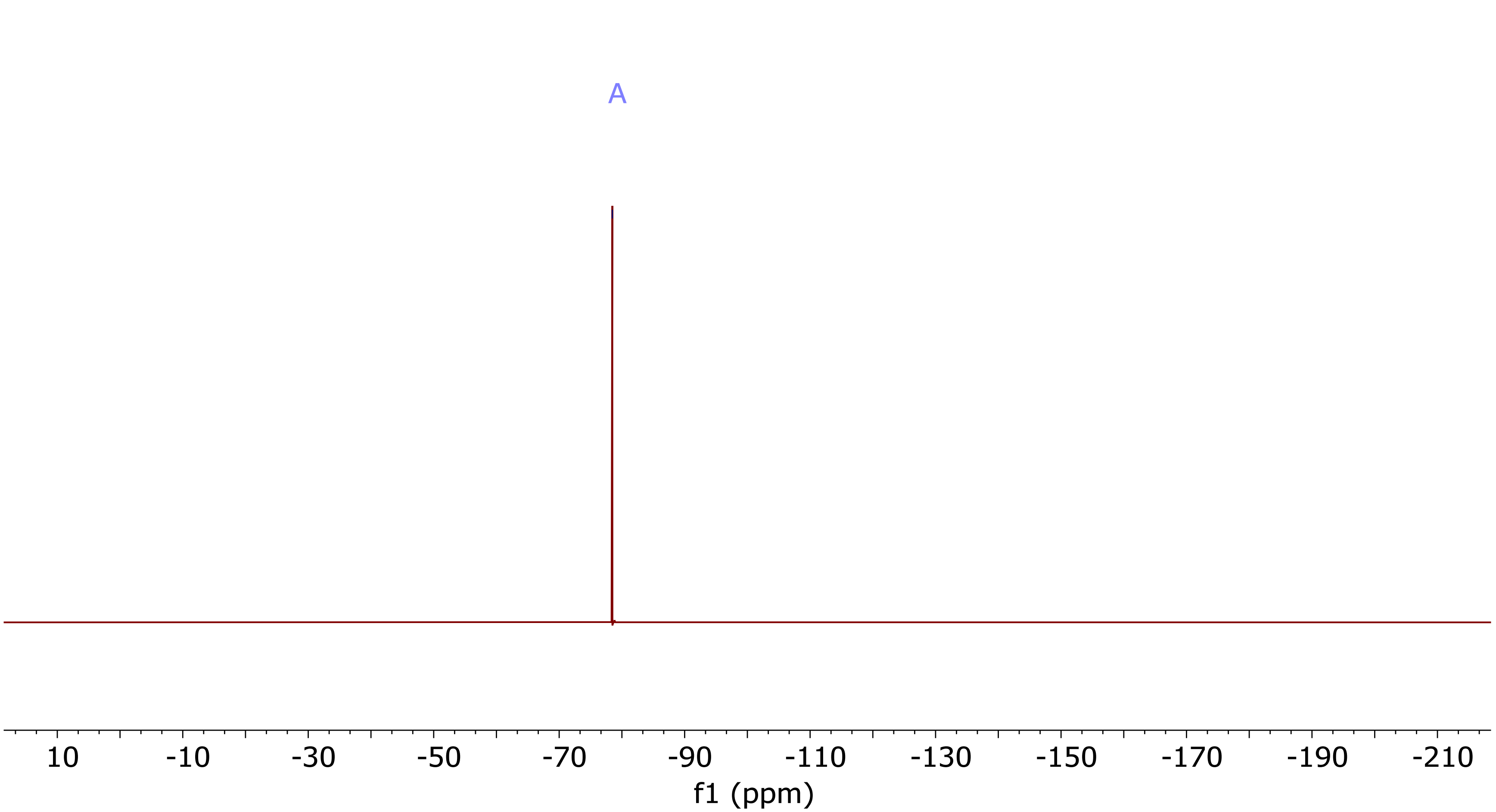

2,4,6-trimethylpyridinium tetrafluoroborate

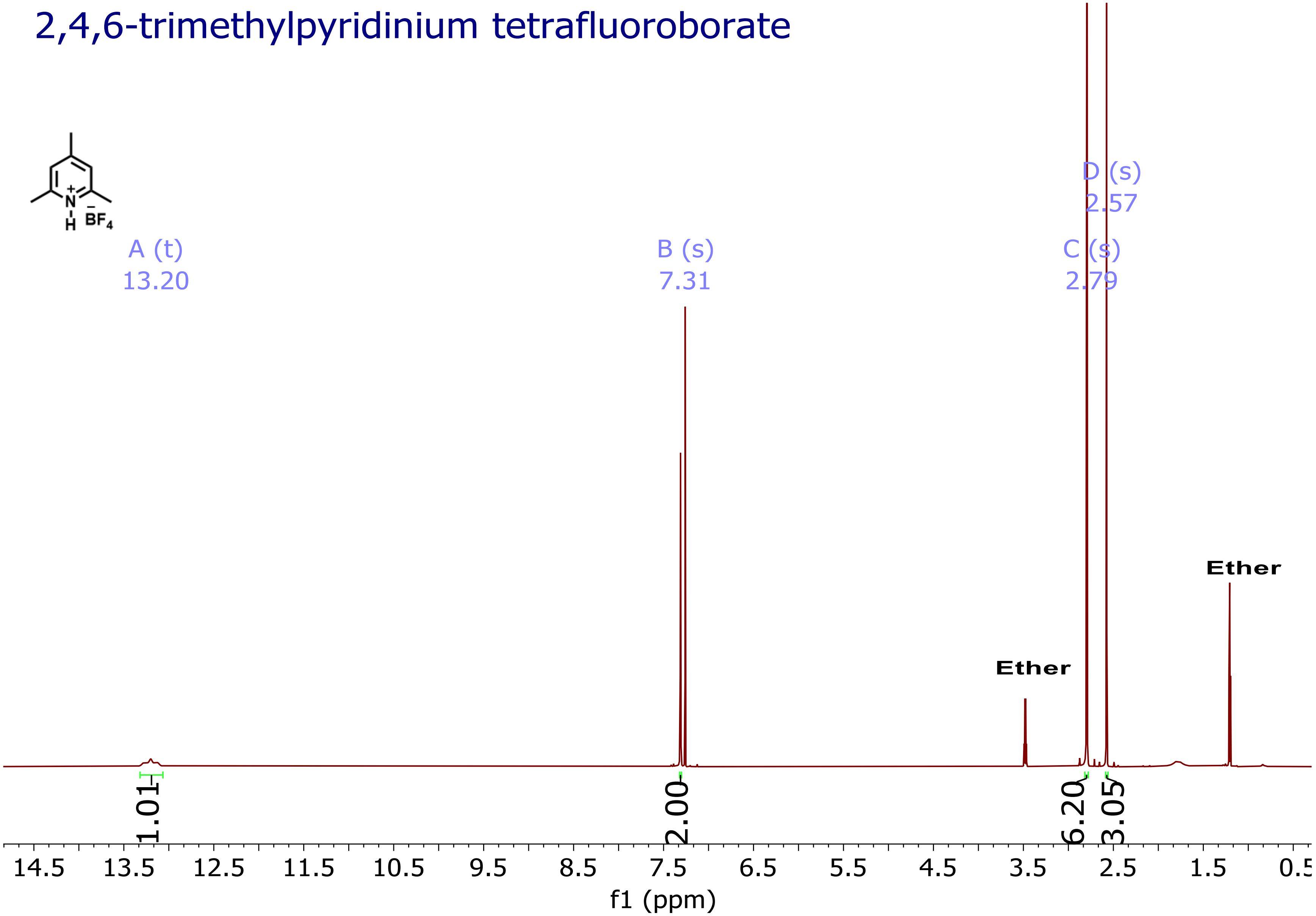

2,4,6-trimethylpyridinium tetrafluoroborate

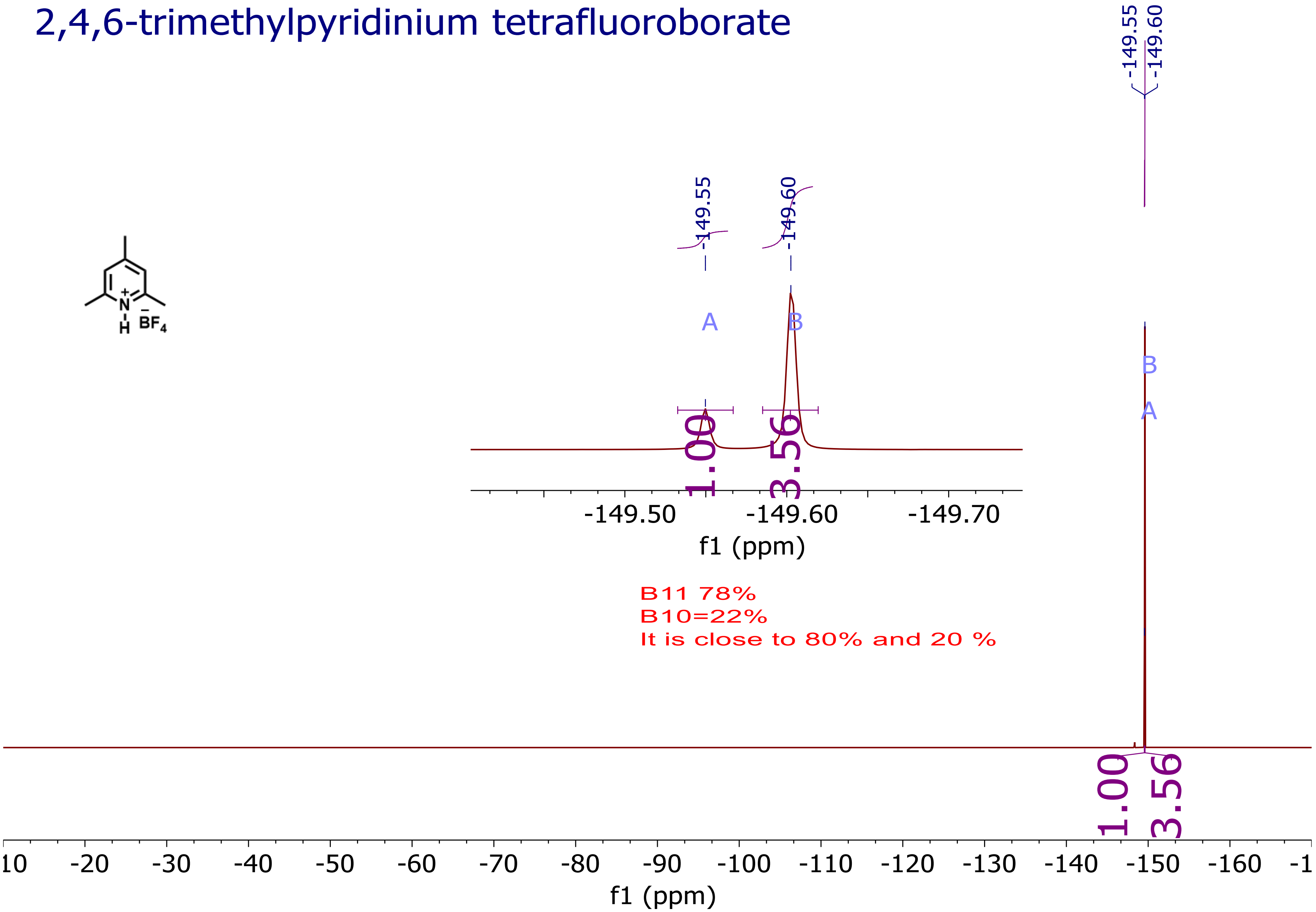

[2a] 2-(1-(4-fluorophenyl)cyclohexyl)-5-methylfuran
1H NMR at 800.34 MHz in CDCl3

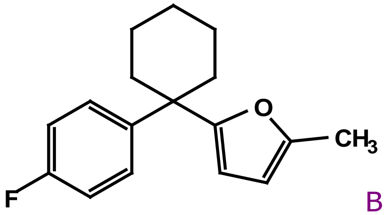

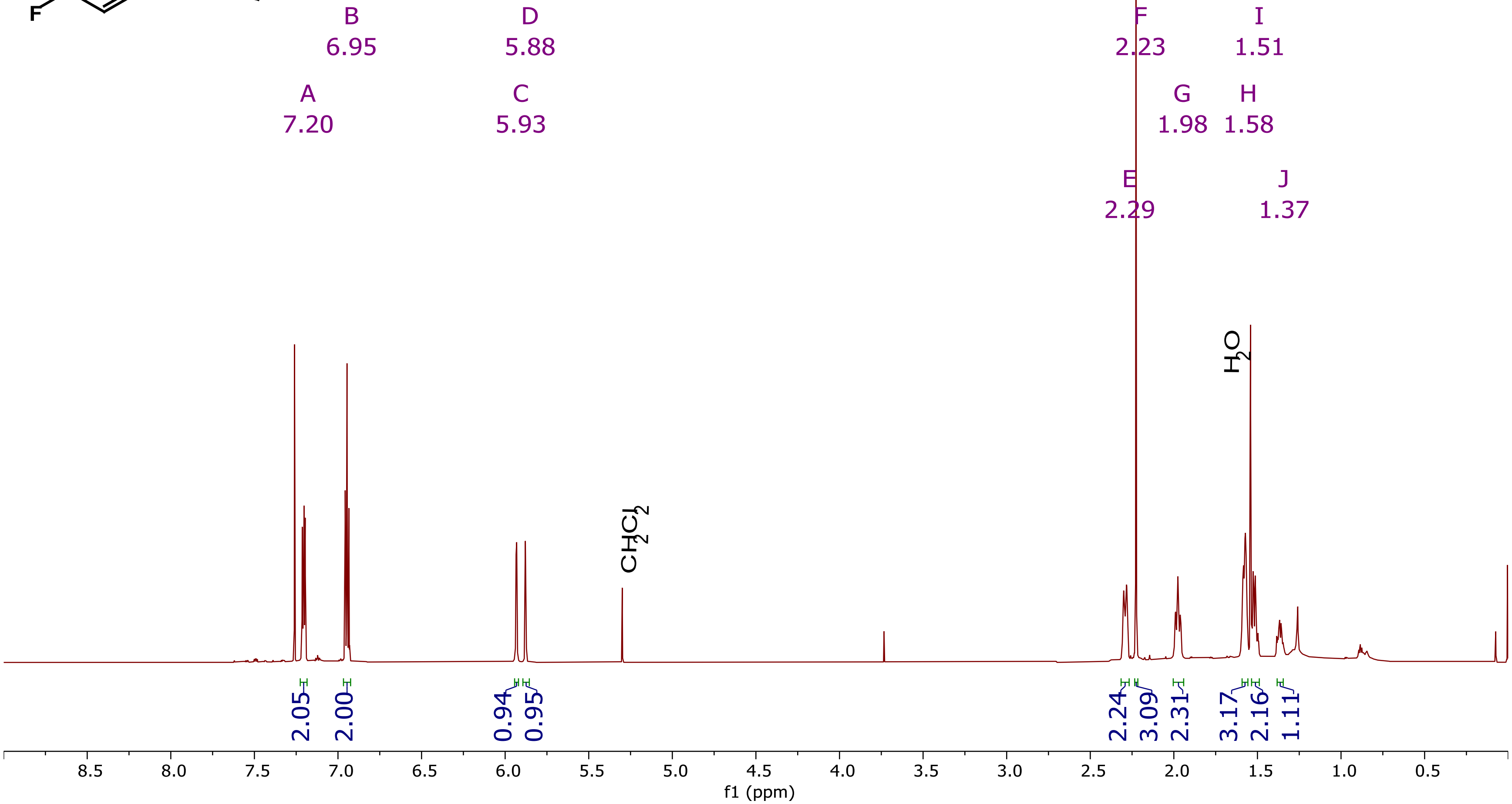

[2a] 2-(1-(4-fluorophenyl)cyclohexyl)-5-methylfuran
13C NMR collected at 201.27 MHz in CDCl3

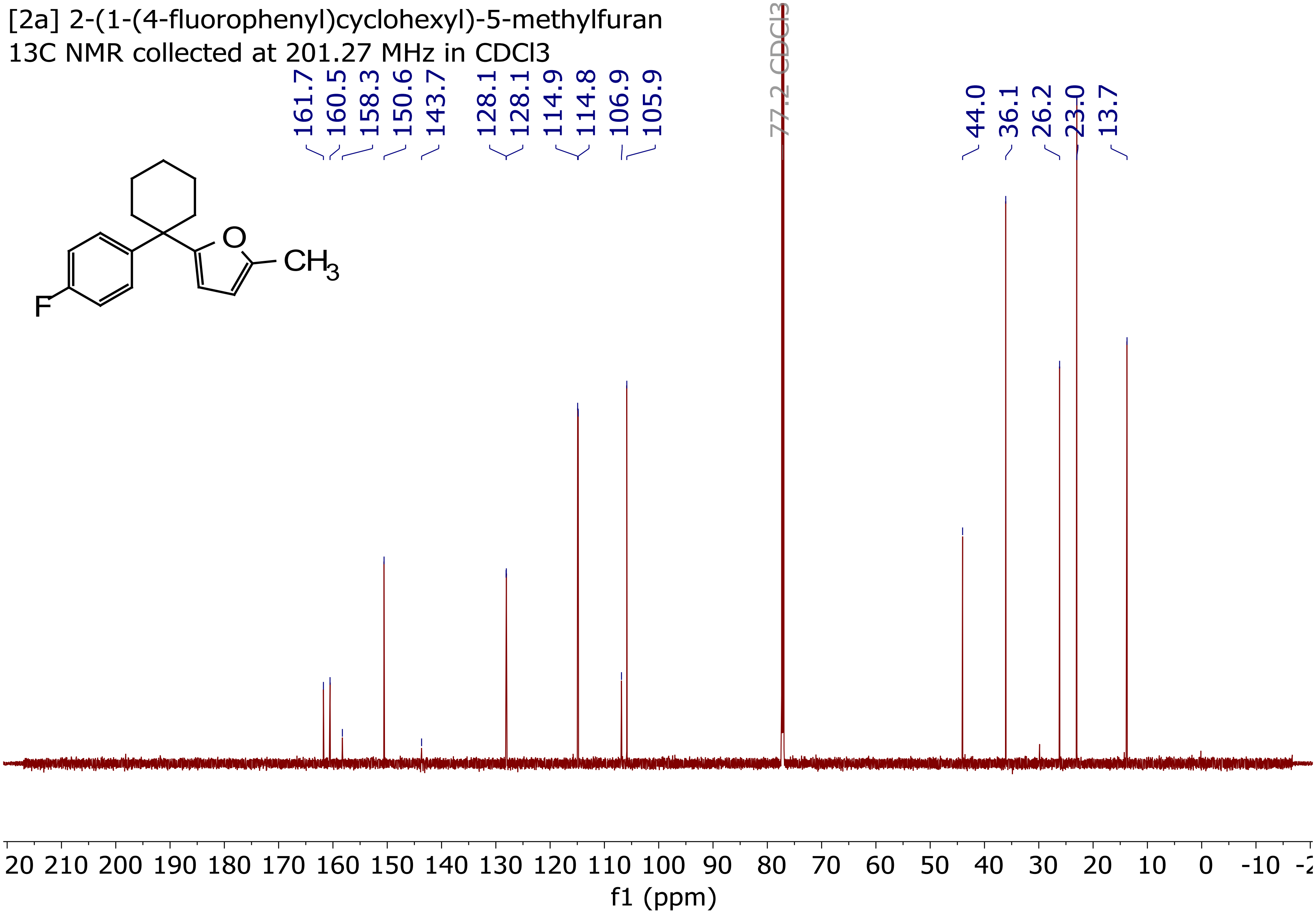

[2a] 2-(1-(4-fluorophenyl)cyclohexyl)-5-methylfuran

19F NMR at 753.00 MHz in CDCl3

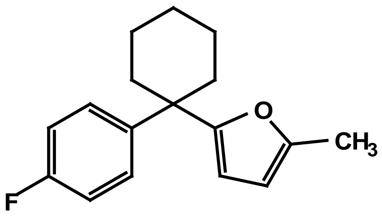

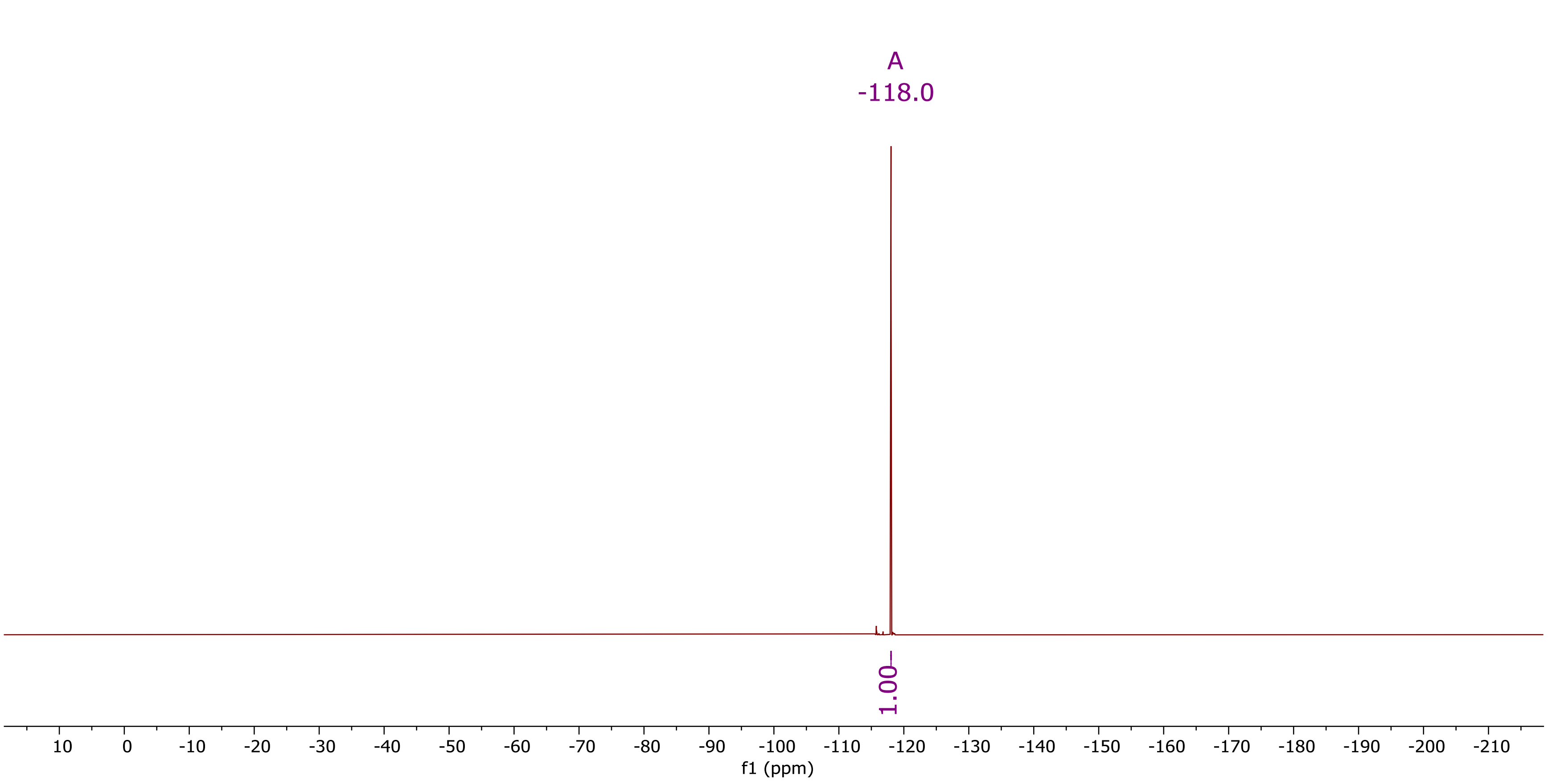

[2b] 2-methyl-5-(1-phenylcyclohexyl)furan
1H NMR collected at 800.34 MHz in CDCl3

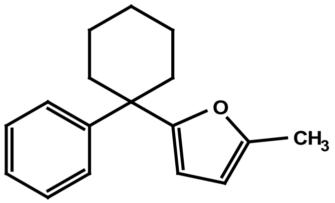

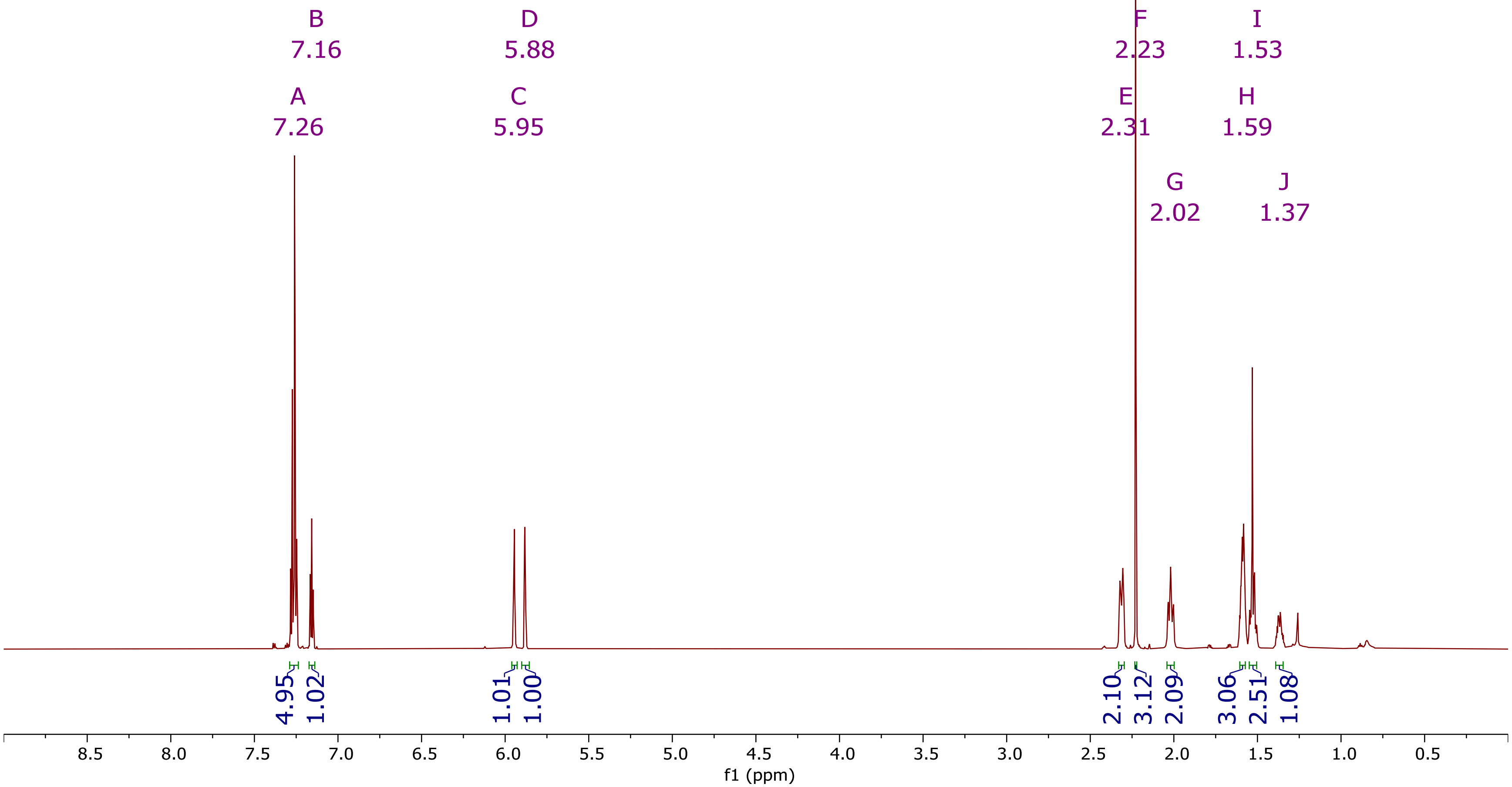

[2b] 2-methyl-5-(1-phenylcyclohexyl)furan
13C NMR collected at 201.27 MHz in CDCl3

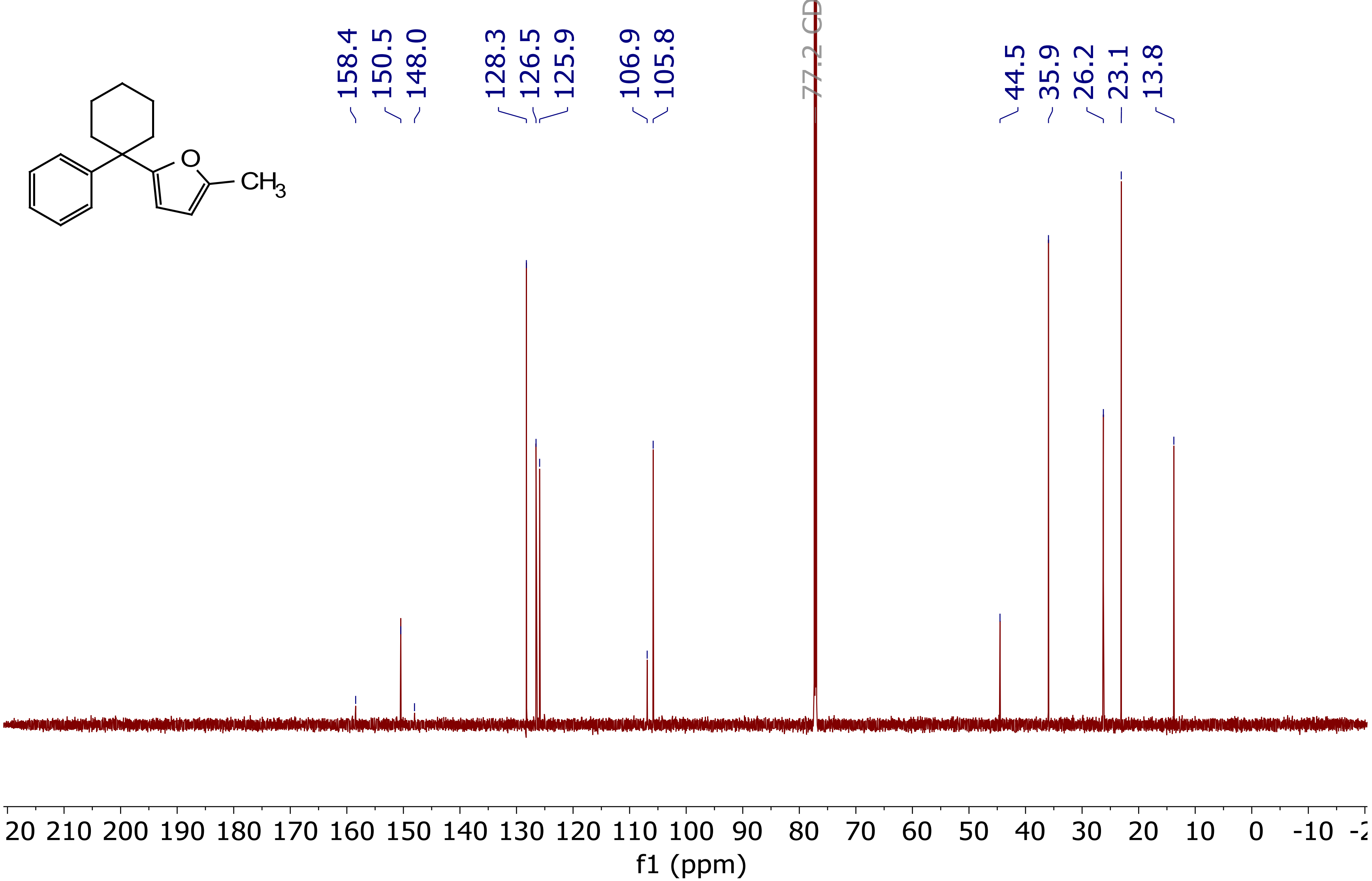

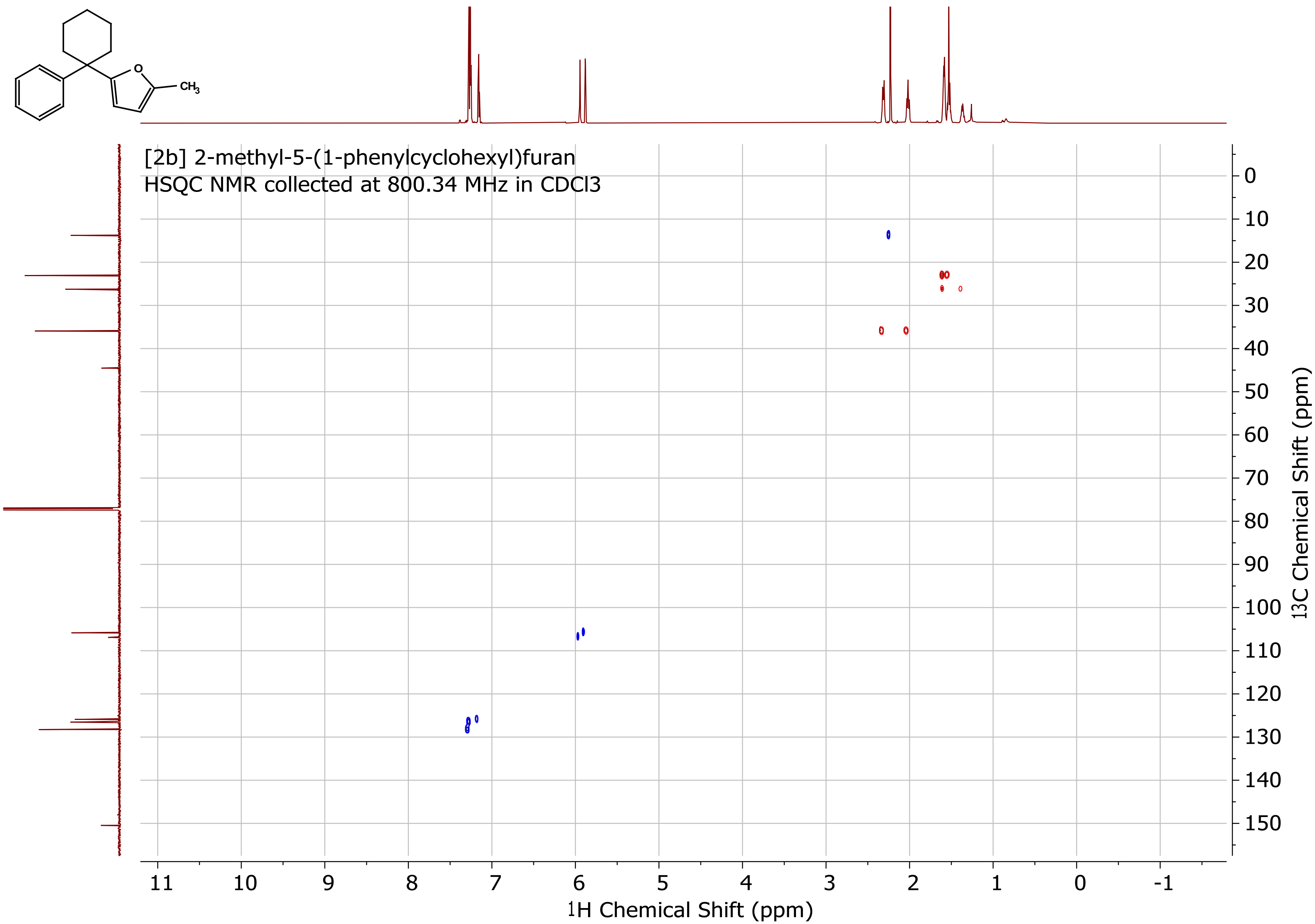
CH3
O
[2b] 2-methyl-5-(1-phenylcyclohexyl)furan
HSQC NMR collected at 800.34 MHz in CDCl3
1H Chemical Shift (ppm)
13C Chemical Shift (ppm)
11
10
9
8
7
6
5
4
3
2
1
0
-1
0
10
20
30
40
50
60
70
80
90
100
110
120
130
140
150

[2c] 2-methyl-5-(1-(p-tolyl)cyclohexyl)furan
1H NMR collected at 800.34 MHz in CDCl3

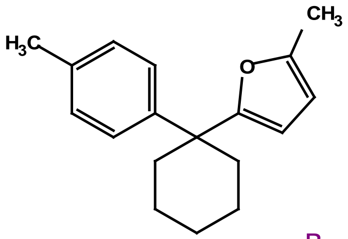

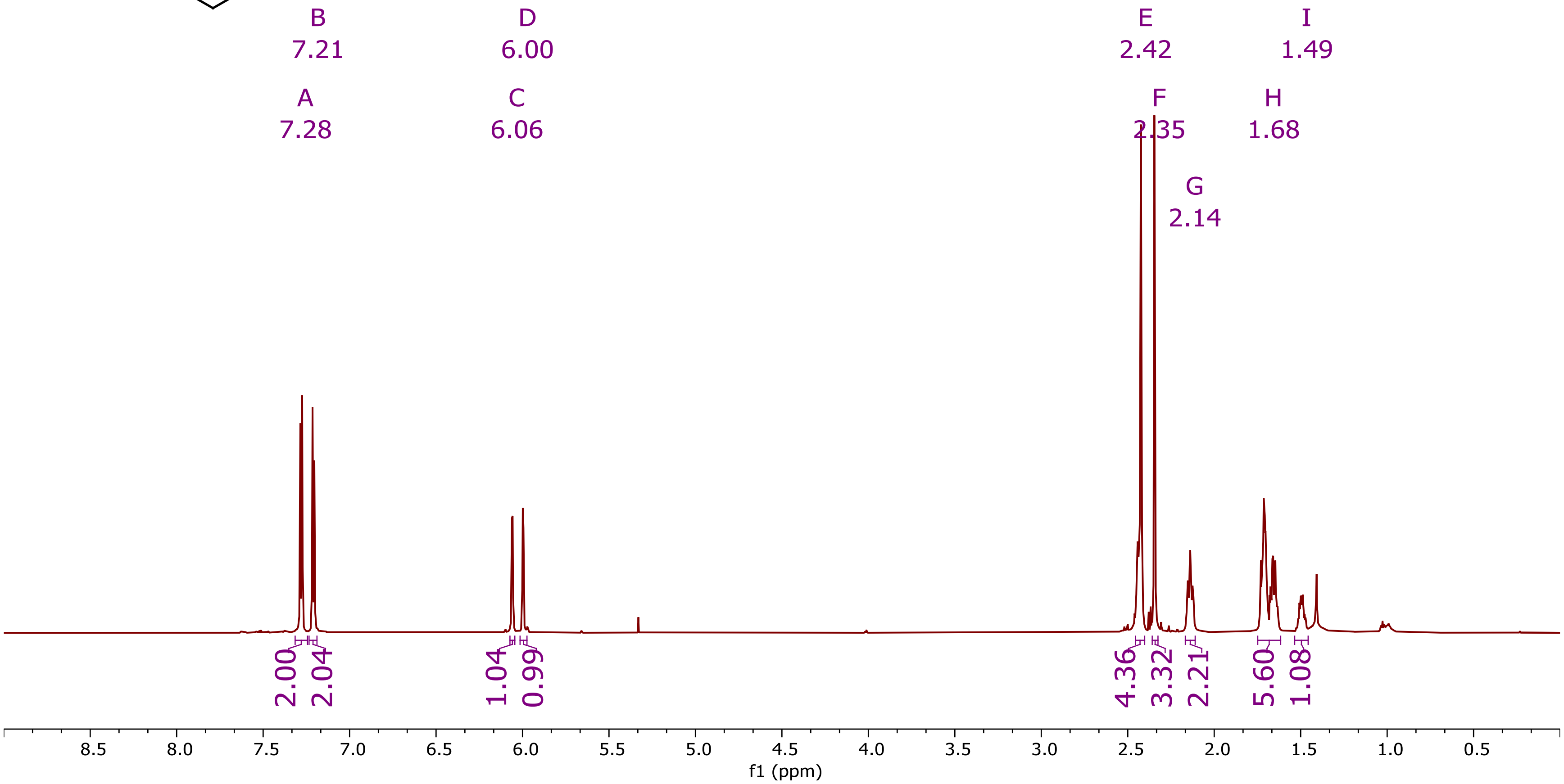

[2c] 2-methyl-5-(1-(p-tolyl)cyclohexyl)furan
13C NMR collected at 201.27 MHz in CDCl3

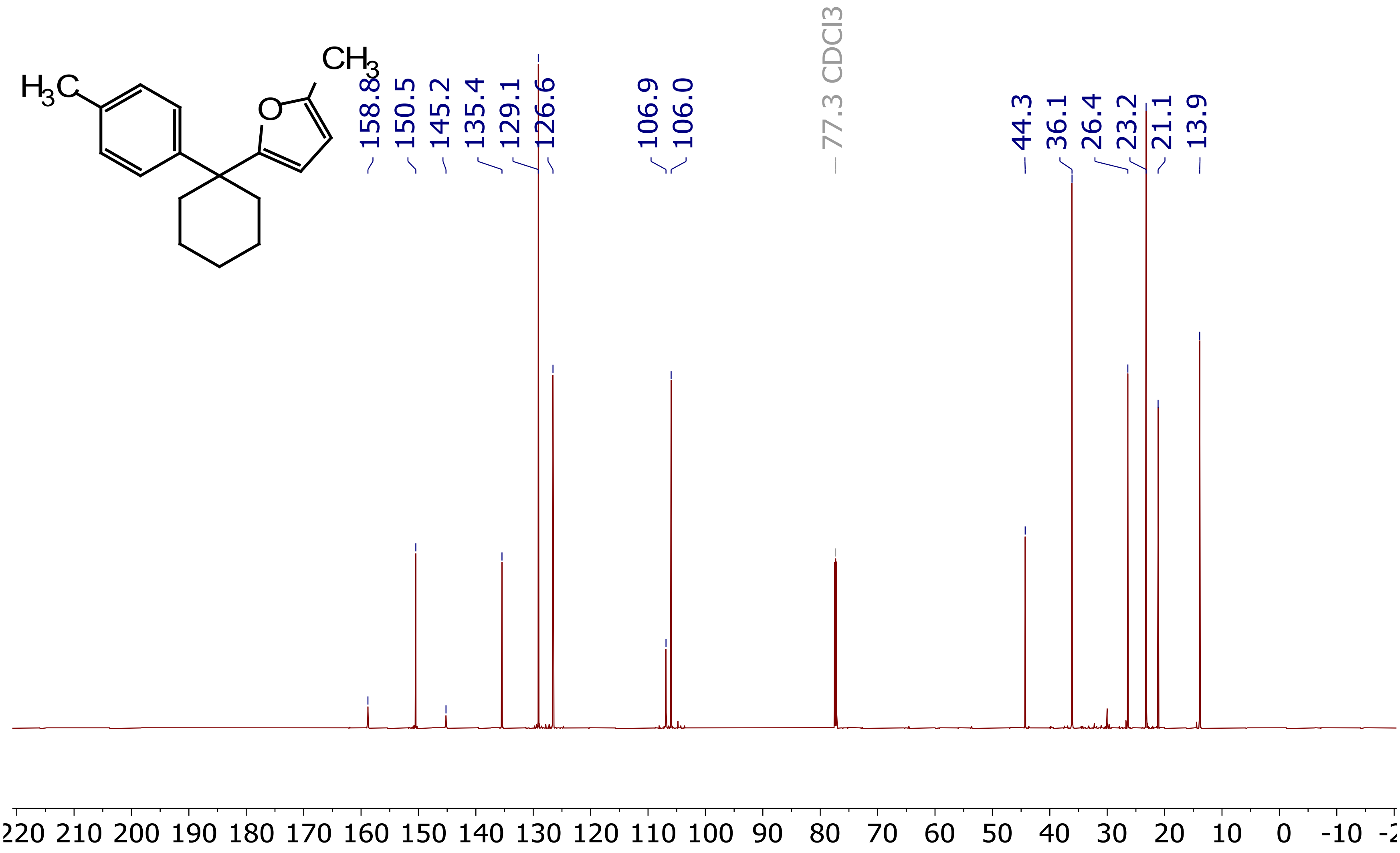

[2d] 2-(1-(3-fluorophenyl)cyclohexyl)-5-methylfuran
1H NMR at 800.34 MHz in CDCl3

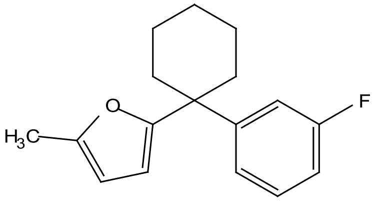

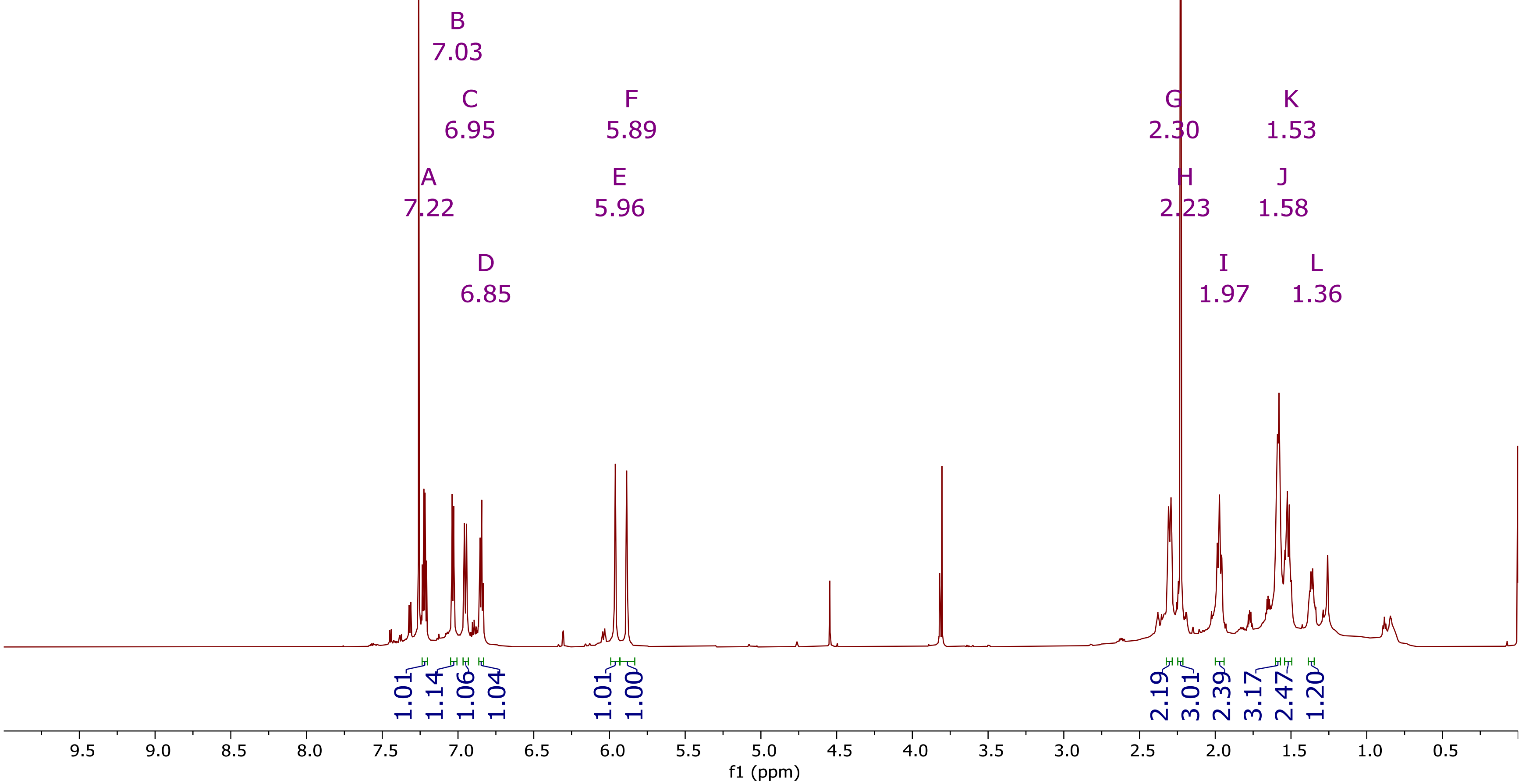

[2d] 2-(1-(3-fluorophenyl)cyclohexyl)-5-methylfuran
13C NMR collected at 201.27 MHz in CDCl3

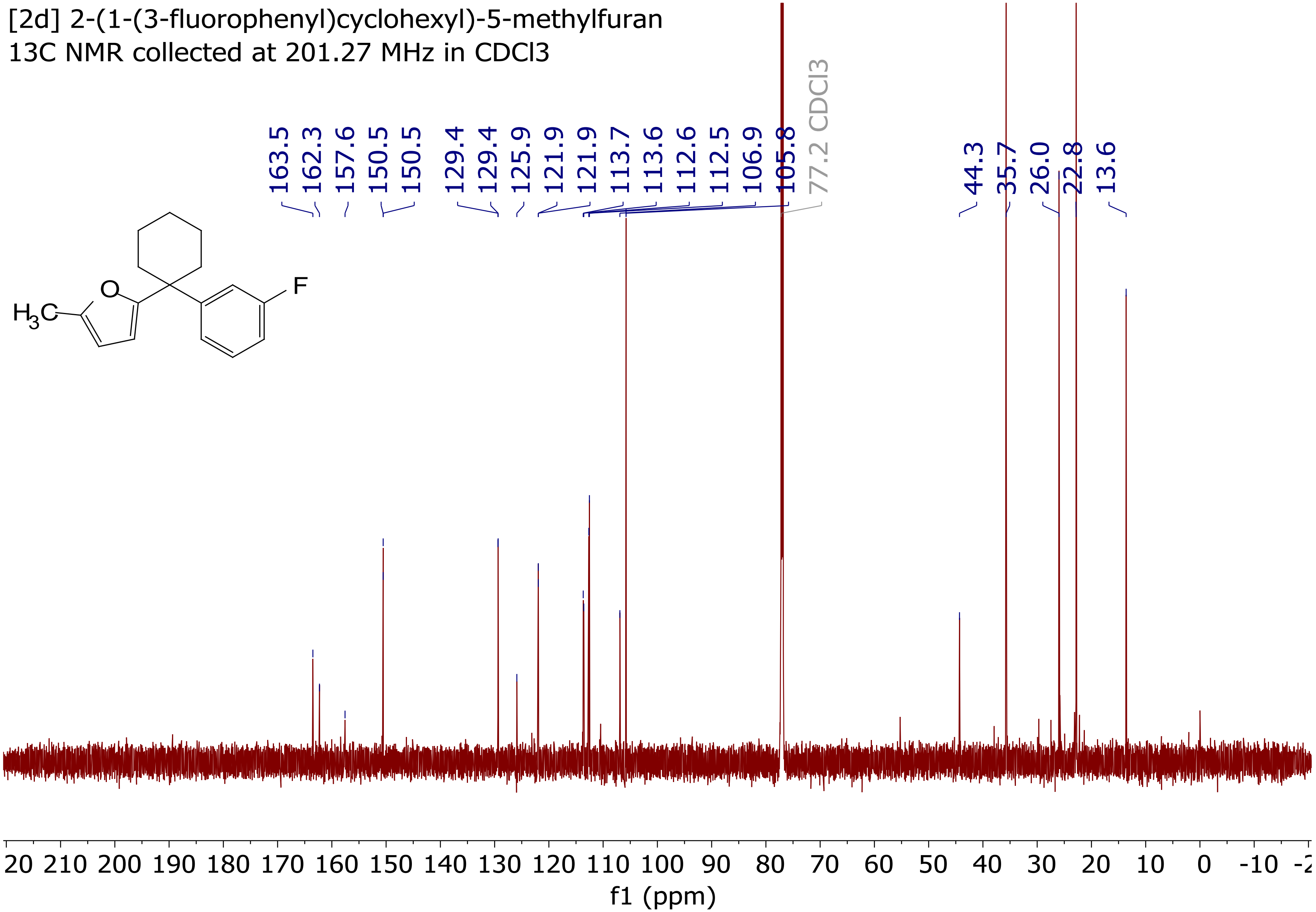

[2d] 2-(1-(3-fluorophenyl)cyclohexyl)-5-methylfuran
19F NMR at 753.00 MHz in CDCl3

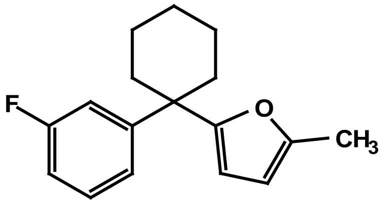

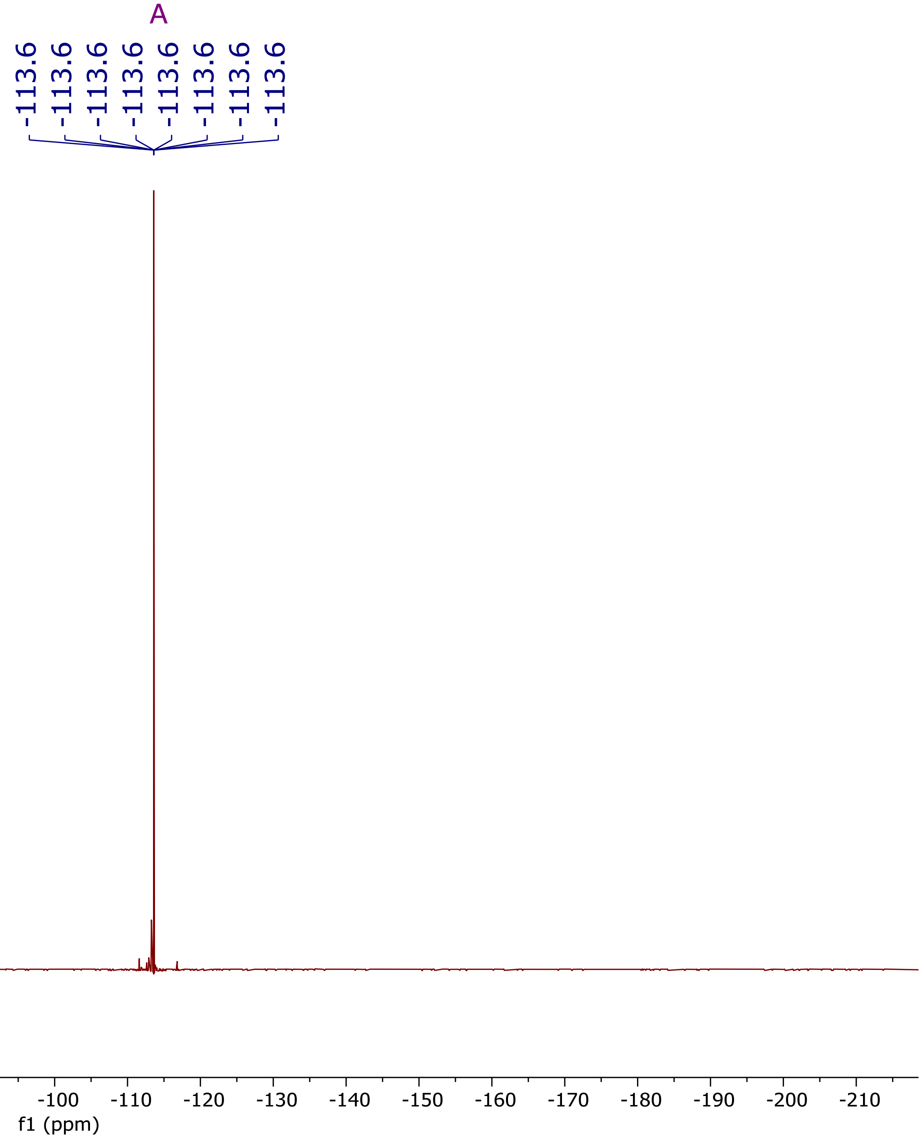

[2e] 2-(1-(4-methoxyphenyl)cyclohexyl)-5-methylfuran
1H NMR collected at 800.34 MHz in CDCl3

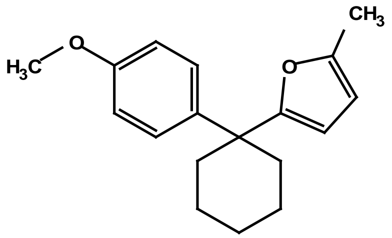

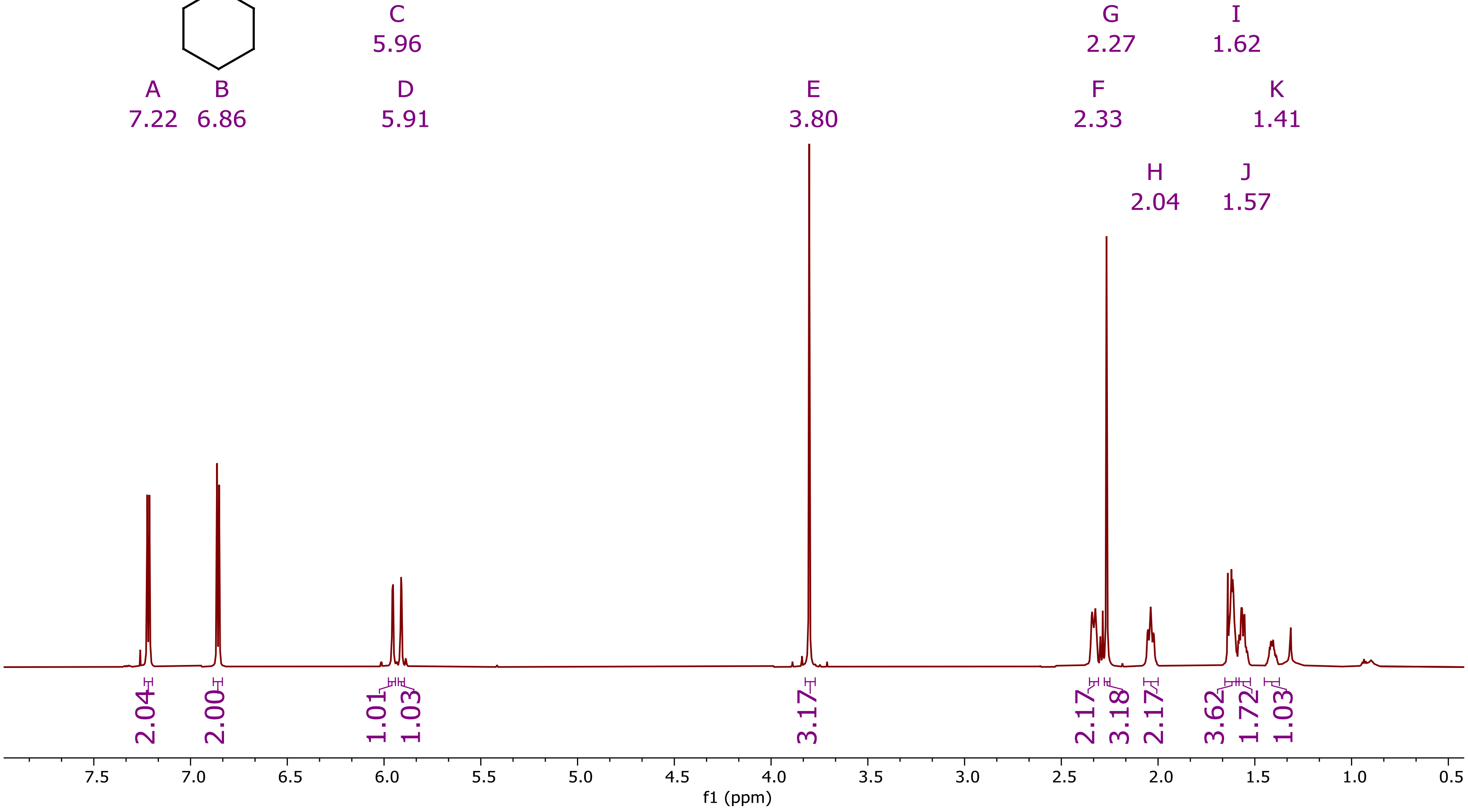

[2e] 2-(1-(4-methoxyphenyl)cyclohexyl)-5-methylfuran
13C NMR collected at 201.27 MHz in CDCl3

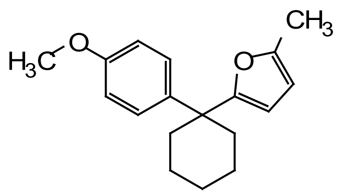

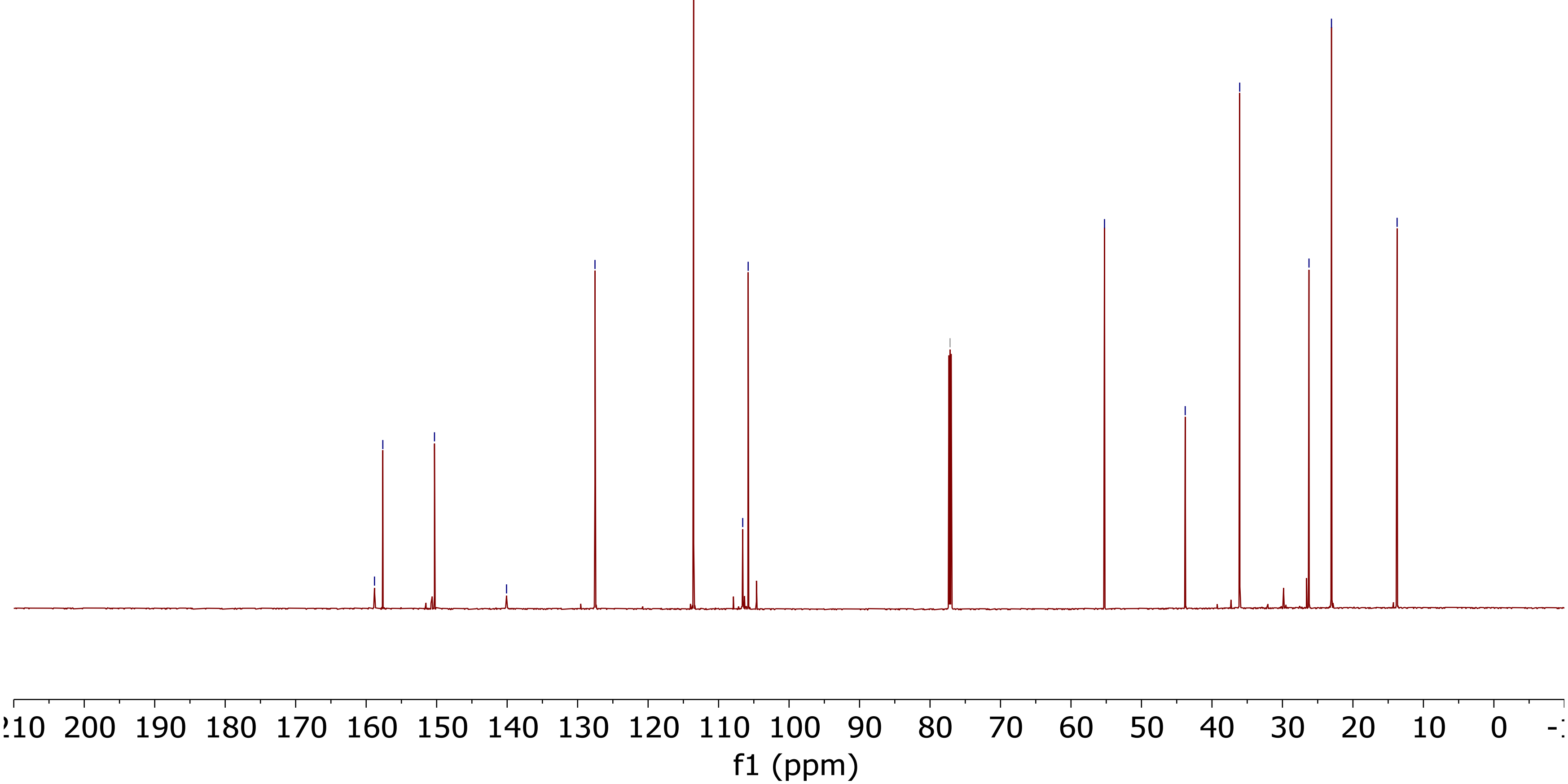

[2f] - 5-(1-(5-methylfuran-2-yl)cyclohexyl)benzo[d][1,3]dioxole
1H NMR collected at 800.34 MHz in CDCl3

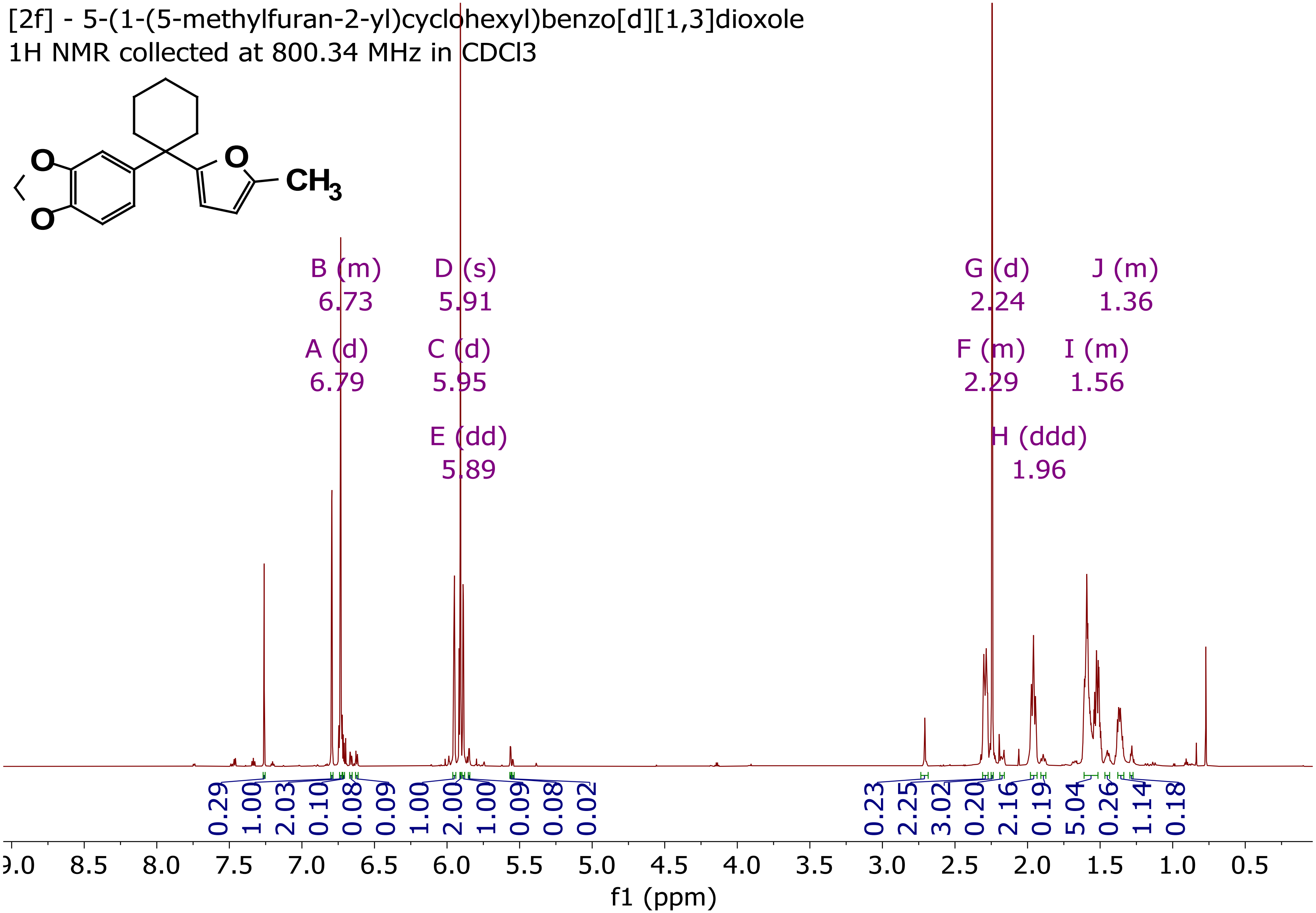

[2f] - 5-(1-(5-methylfuran-2-yl)cyclohexyl)benzo[d][1,3]dioxole
13C NMR collected at 201.27 MHz in CDCl3

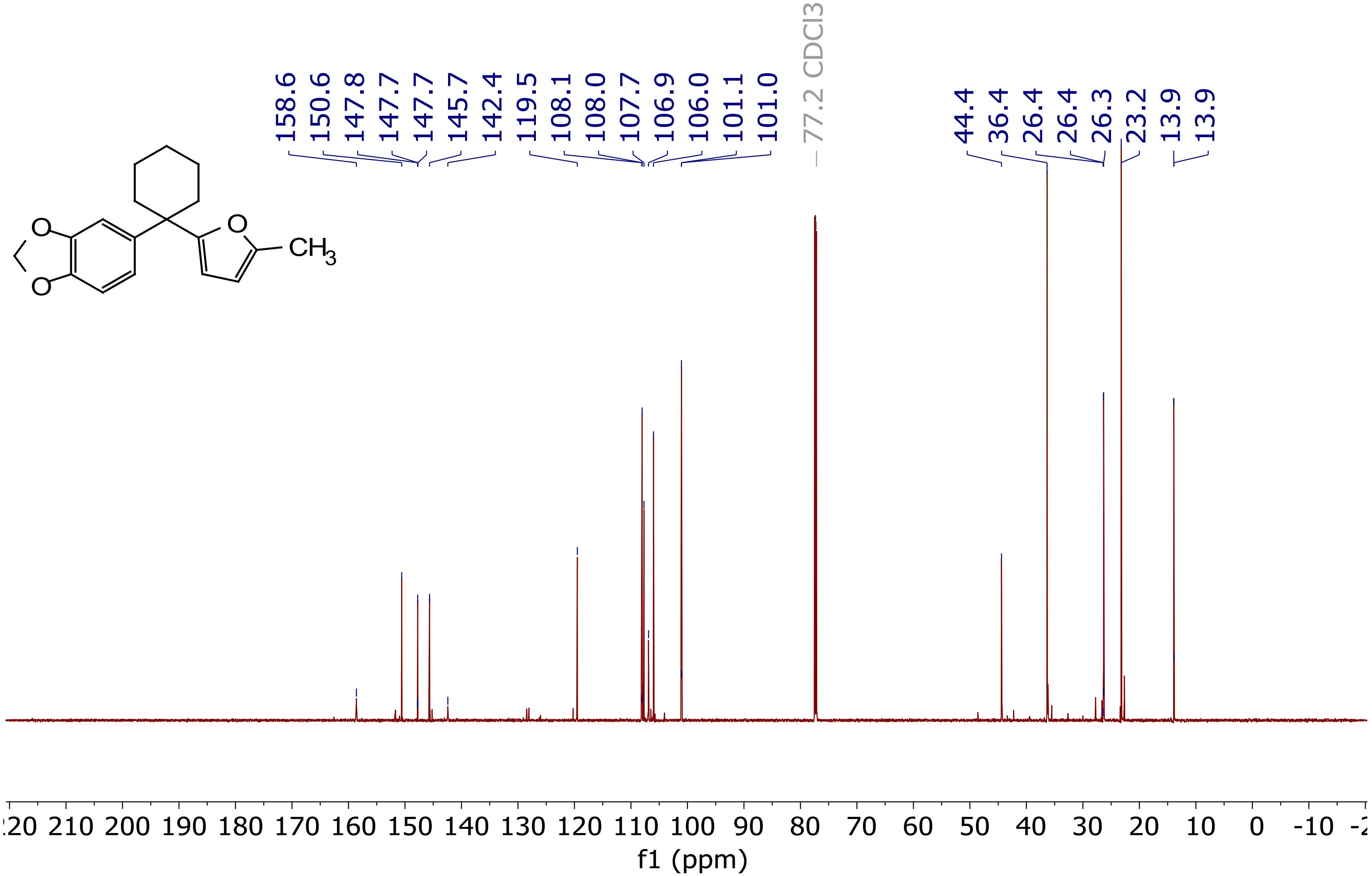

[2g] 2-methyl-5-(1-(4-(trifluoromethyl)phenyl)cyclohexyl)furan
1H NMR collected at 800.34 MHz in CDCl3

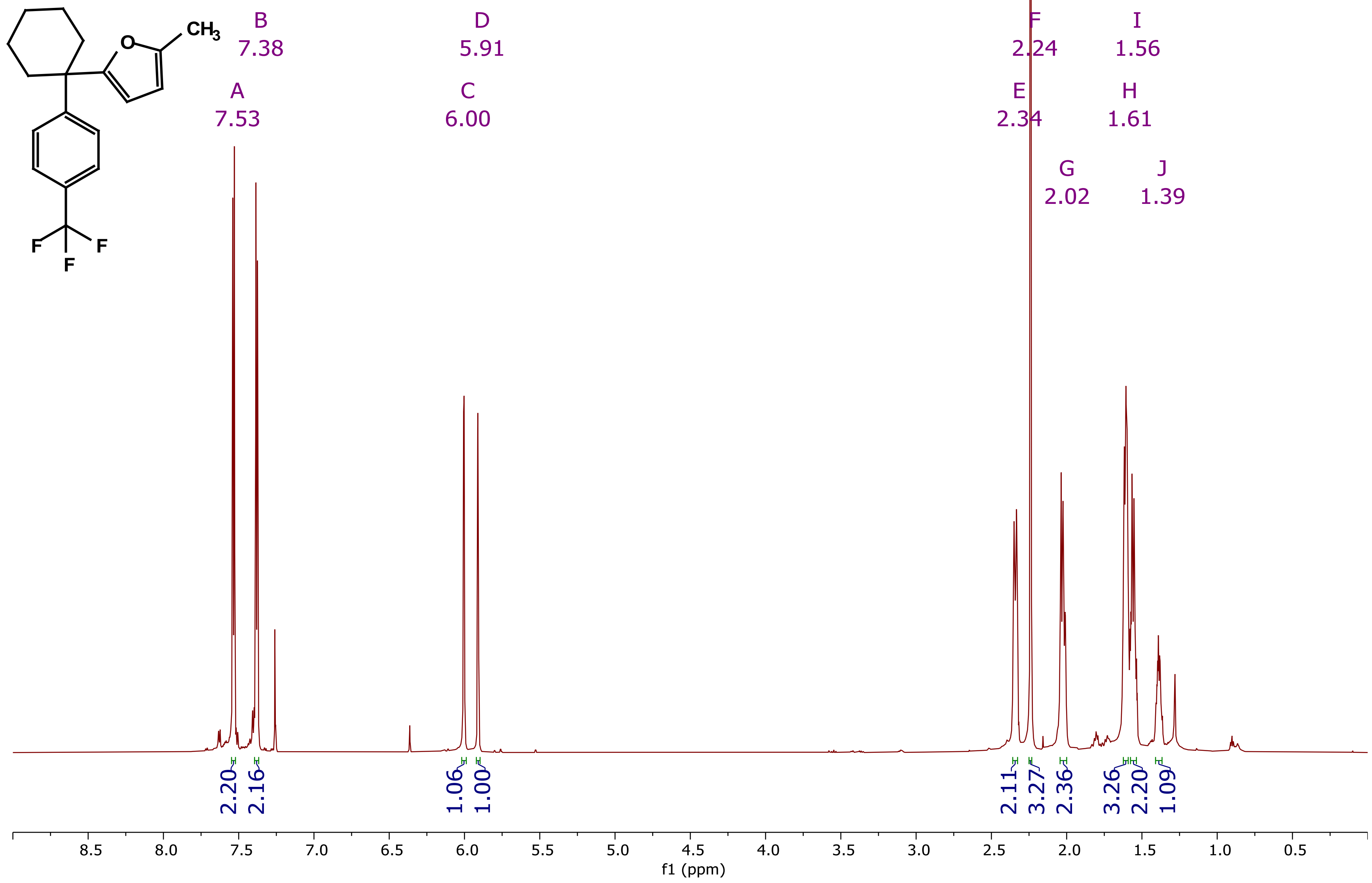

[2g] 2-methyl-5-(1-(4-(trifluoromethyl)phenyl)cyclohexyl)furan
13C NMR collected at 201.27 MHz in CDCl3

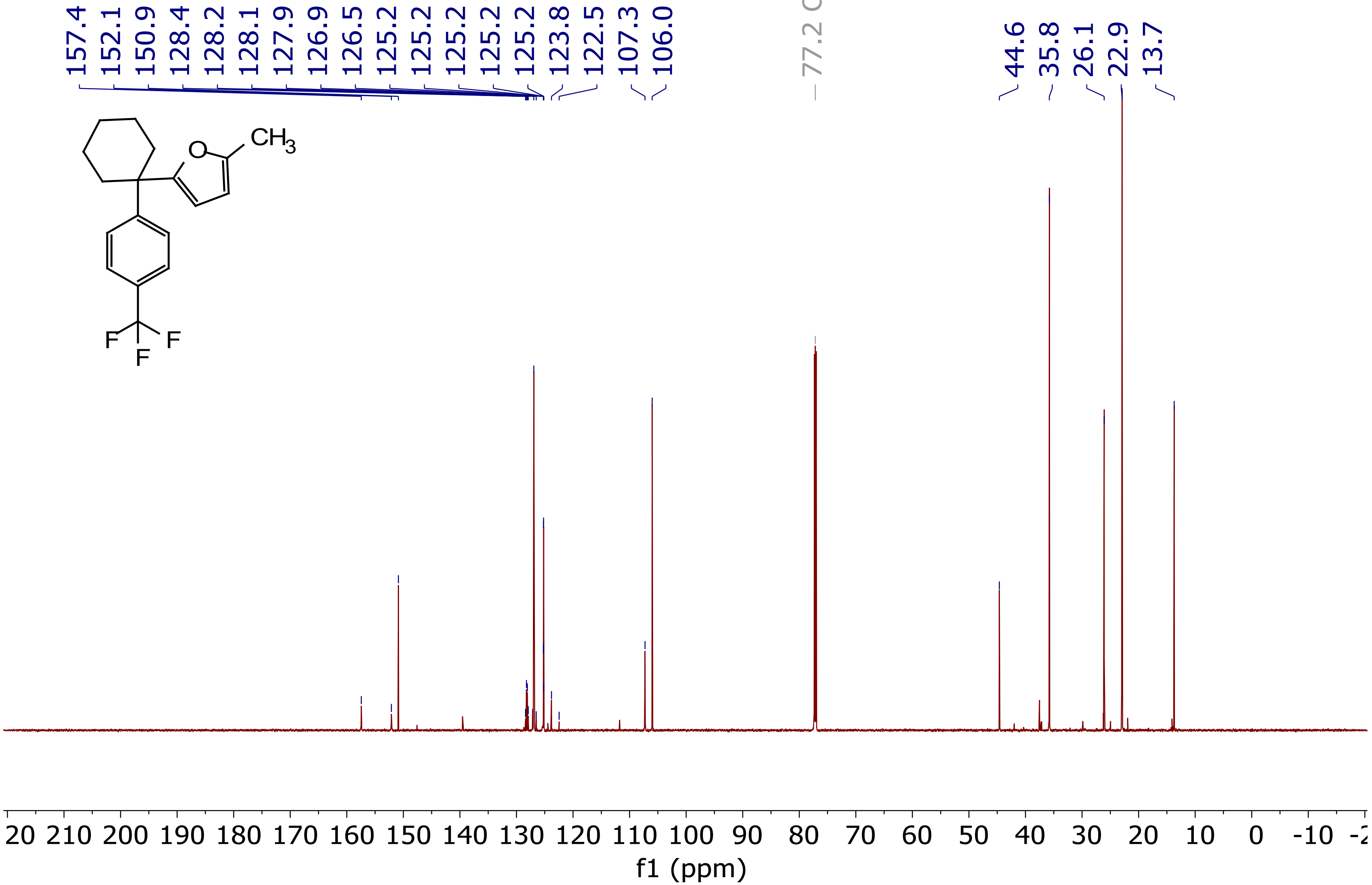

[2g] 2-methyl-5-(1-(4-(trifluoromethyl)phenyl)cyclohexyl)furan
19F NMR collected at 753.00 MHz in CDCl3

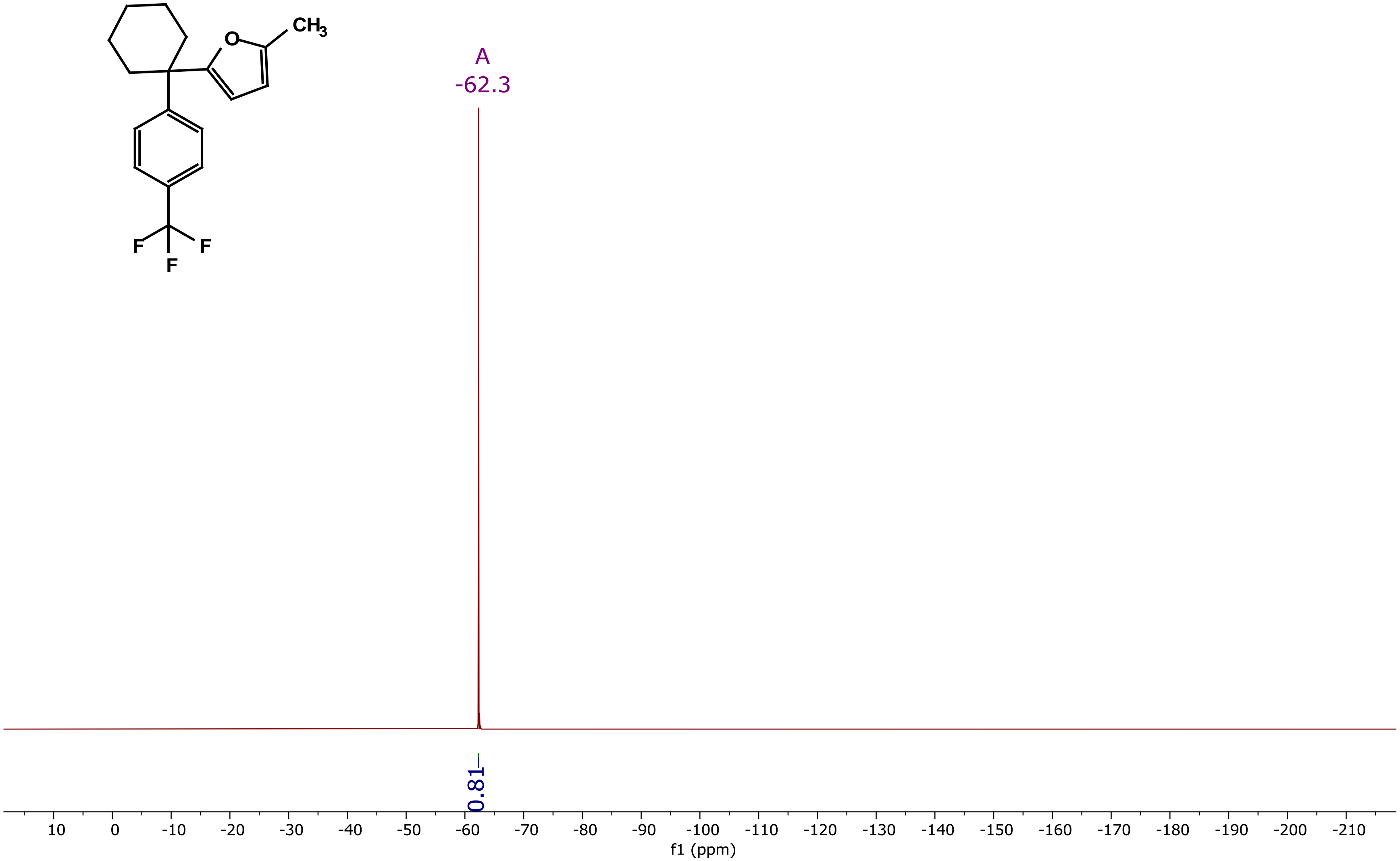

[2gg] 4a,4b-bis(4-(trifluoromethyl)phenyl)dodecahydrobiphenylene
1H NMR collected at 800.34 MHz in CDCl3

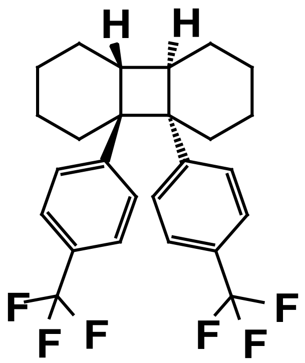

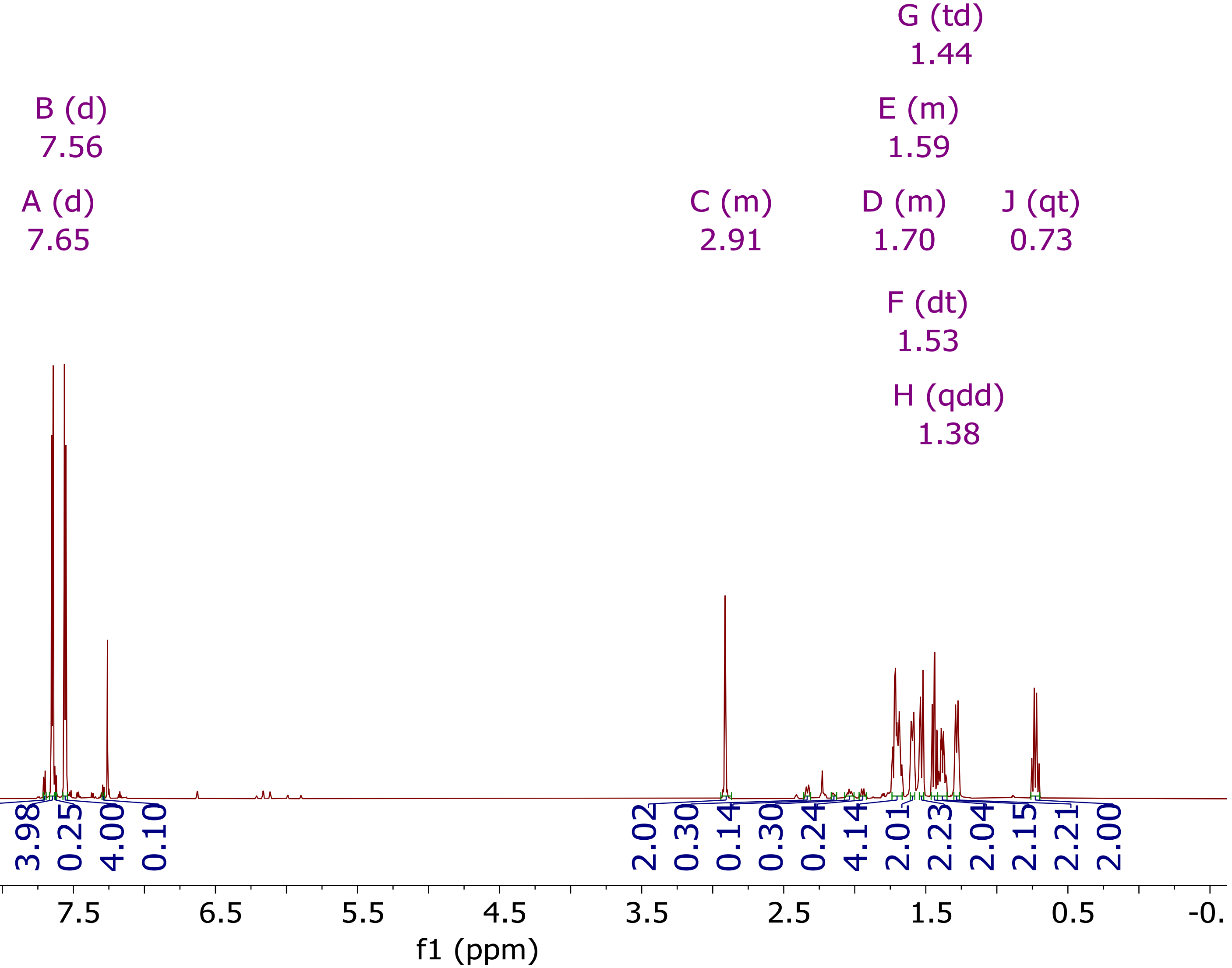

[2gg] 4a,4b-bis(4-(trifluoromethyl)phenyl)dodecahydrobiphenylene
13C NMR collected at 201.27 MHz in CDCl3

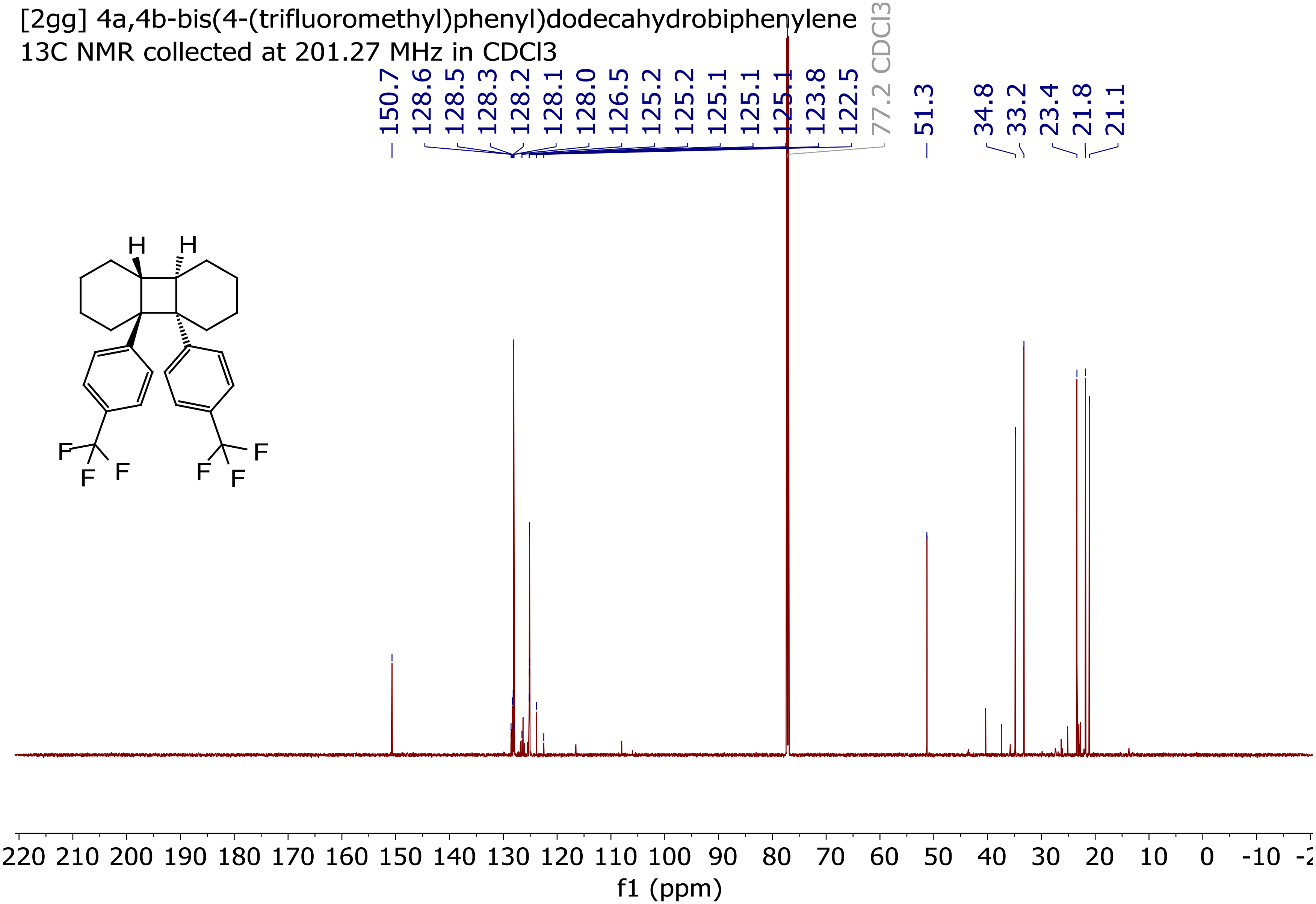

[2gg] 4a,4b-bis(4-(trifluoromethyl)phenyl)dodecahydrobiphenylene
19F NMR collected at 753.00 MHz in CDCl3

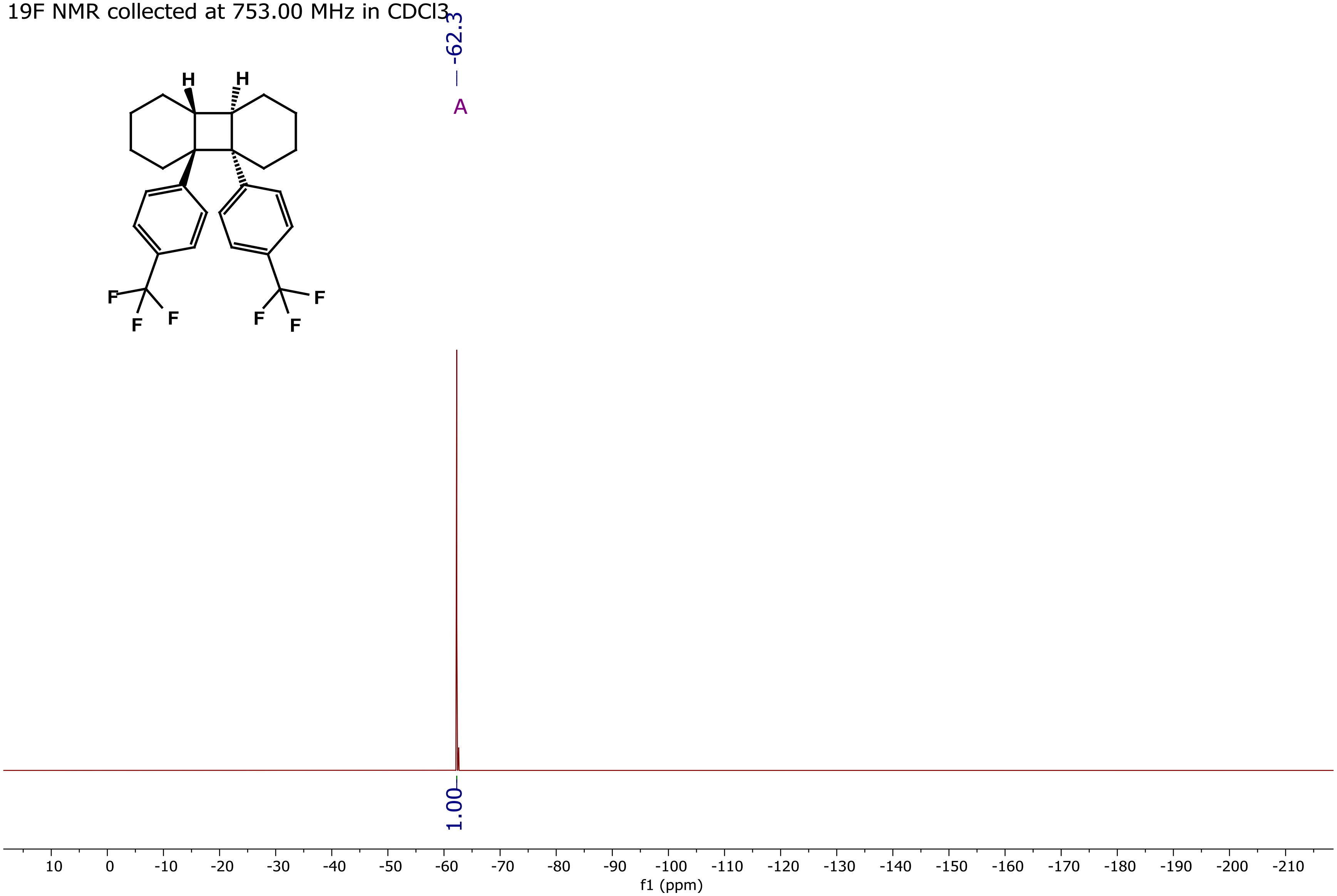

[3a] 2-(1-(4-fluorophenyl)cyclohexyl)-5-methylthiophene
1H NMR at 800.34 MHz in CDCl3

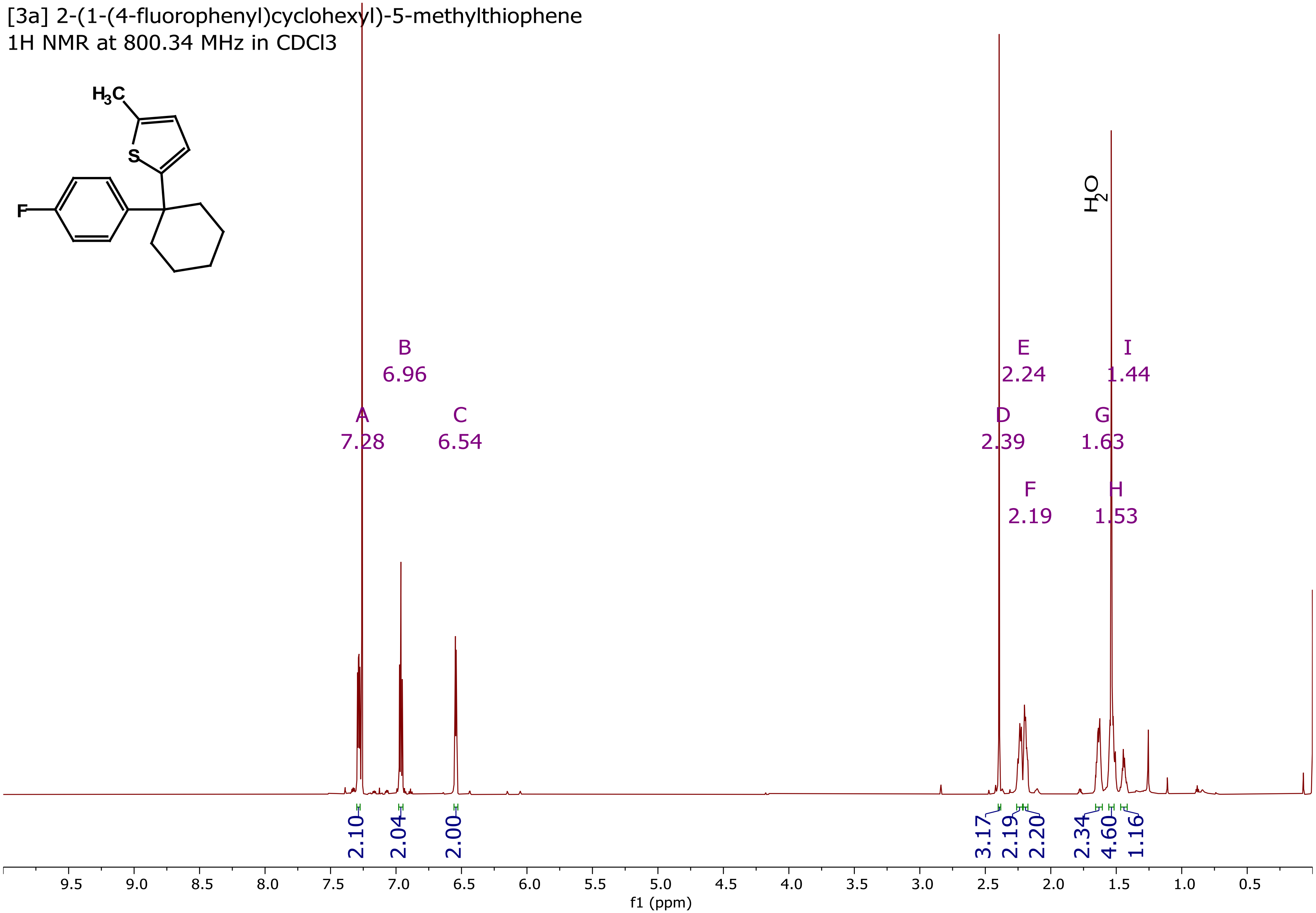

[3a] 2-(1-(4-fluorophenyl)cyclohexyl)-5-methylthiophene
13C NMR collected at 201.27 MHz in CDCl3

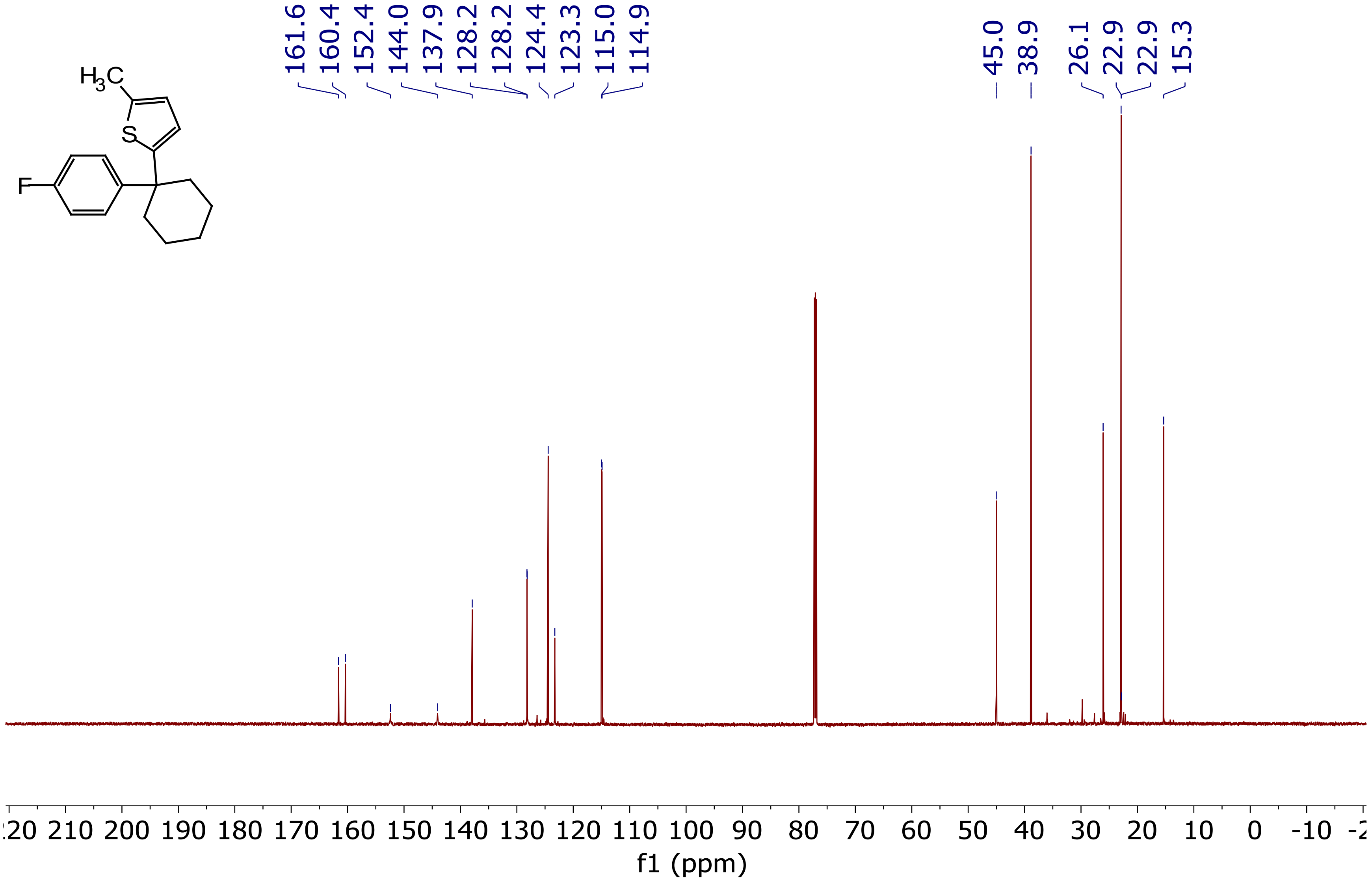

[3a] 2-(1-(4-fluorophenyl)cyclohexyl)-5-methylthiophene
19F NMR at 753.00 MHz in CDCl3

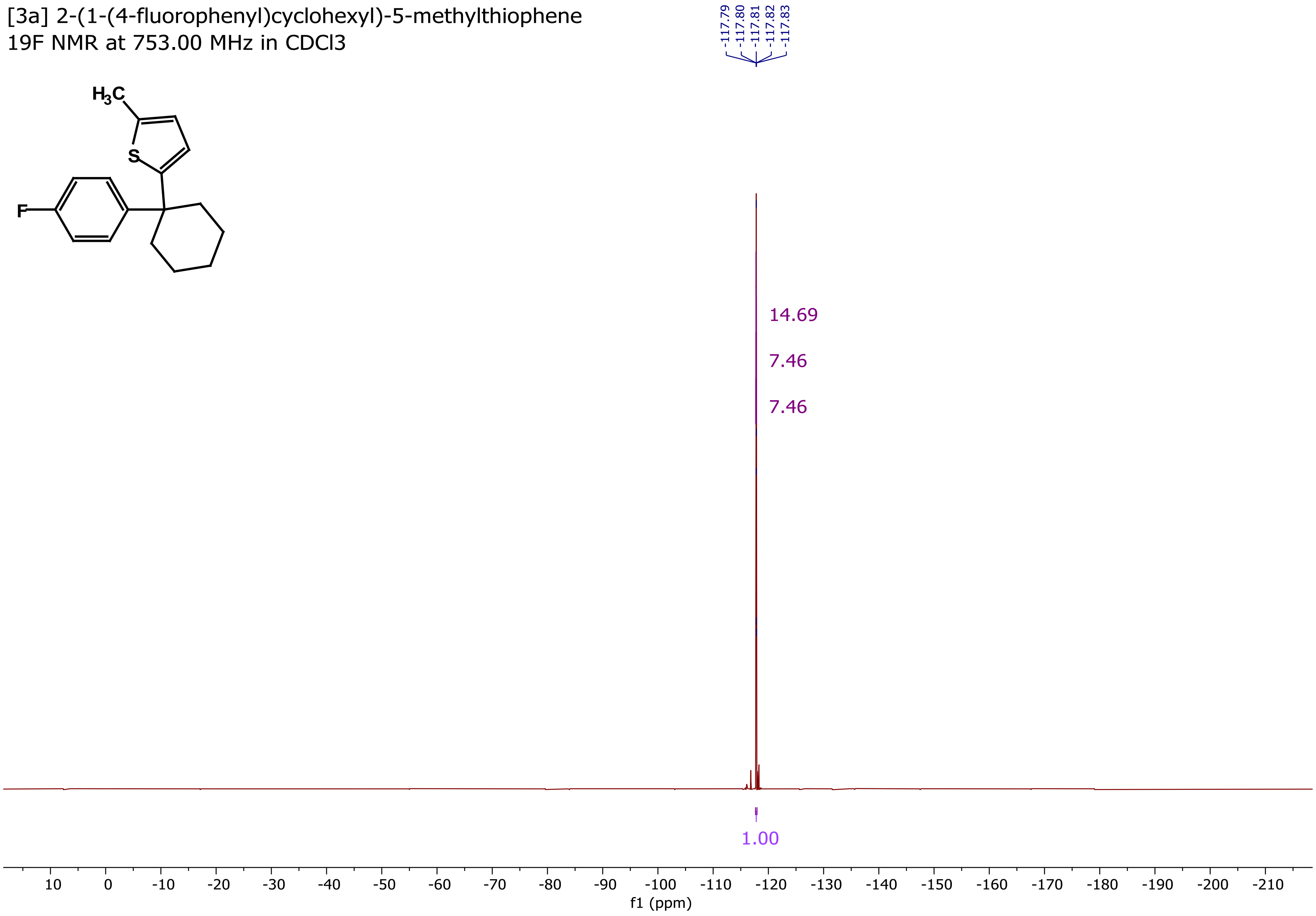

[4b] - 2,5-bis(1-phenylcyclohexyl)thiophene
1H NMR collected at 800.34 MHz in CDCl3

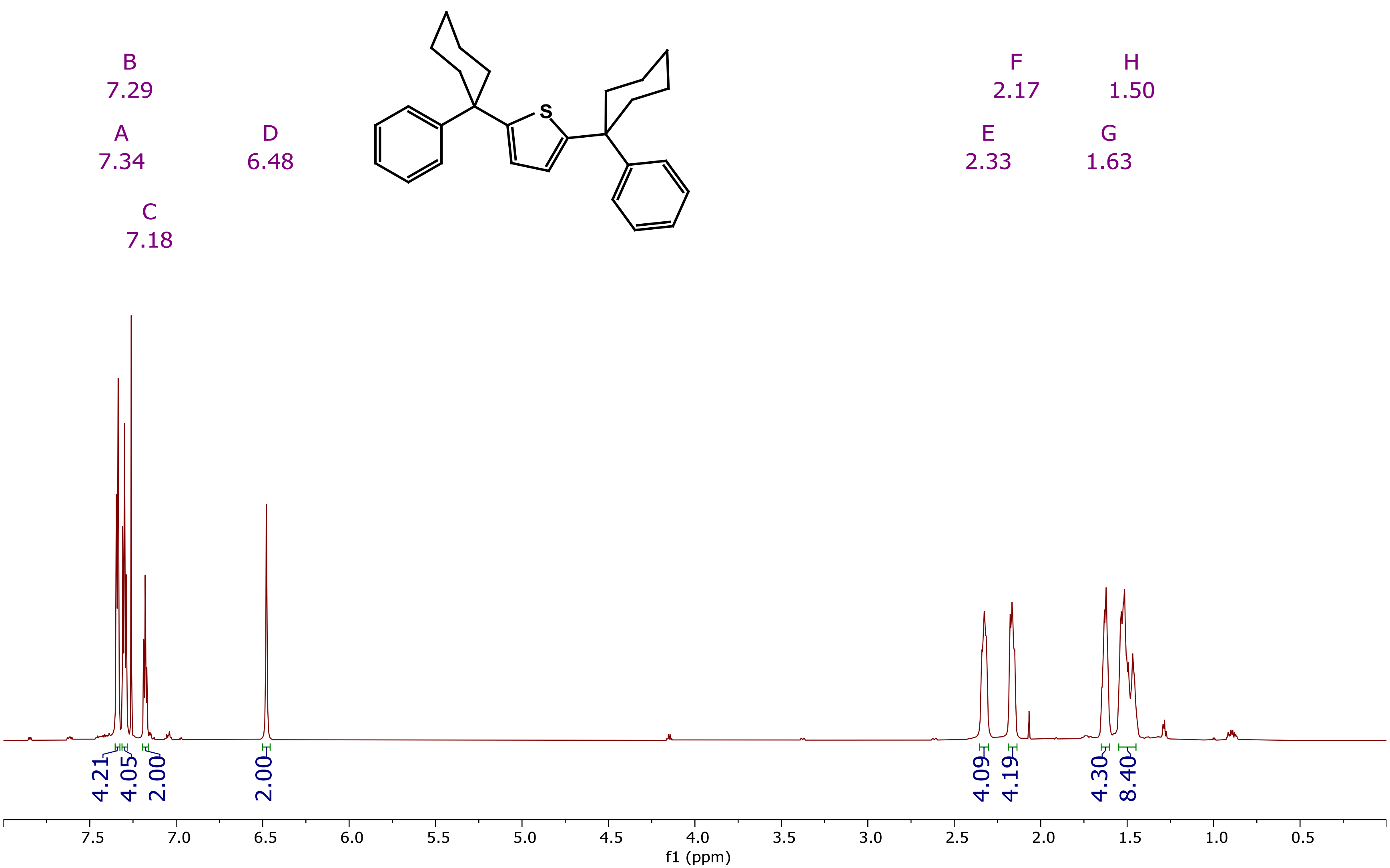

[4b] - 2,5-bis(1-phenylcyclohexyl)thiophene
13C NMR collected at 201.27 MHz in CDCl3

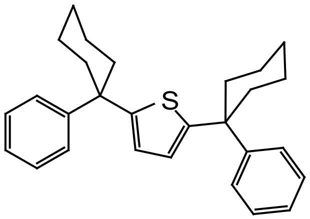

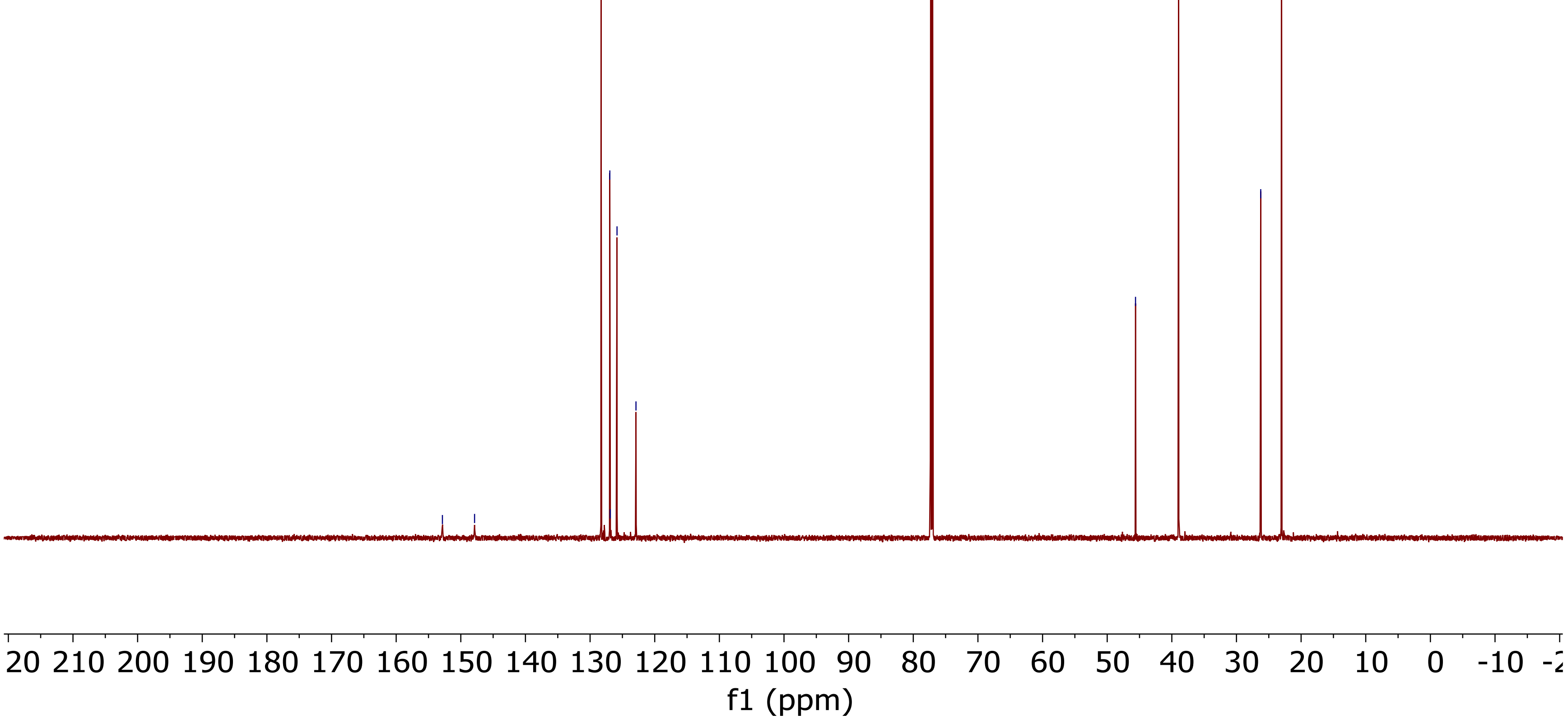

[5b] 2-(1-phenylcyclohexyl)-1H-pyrrole
1H NMR at 800.34 MHz in CDCl3

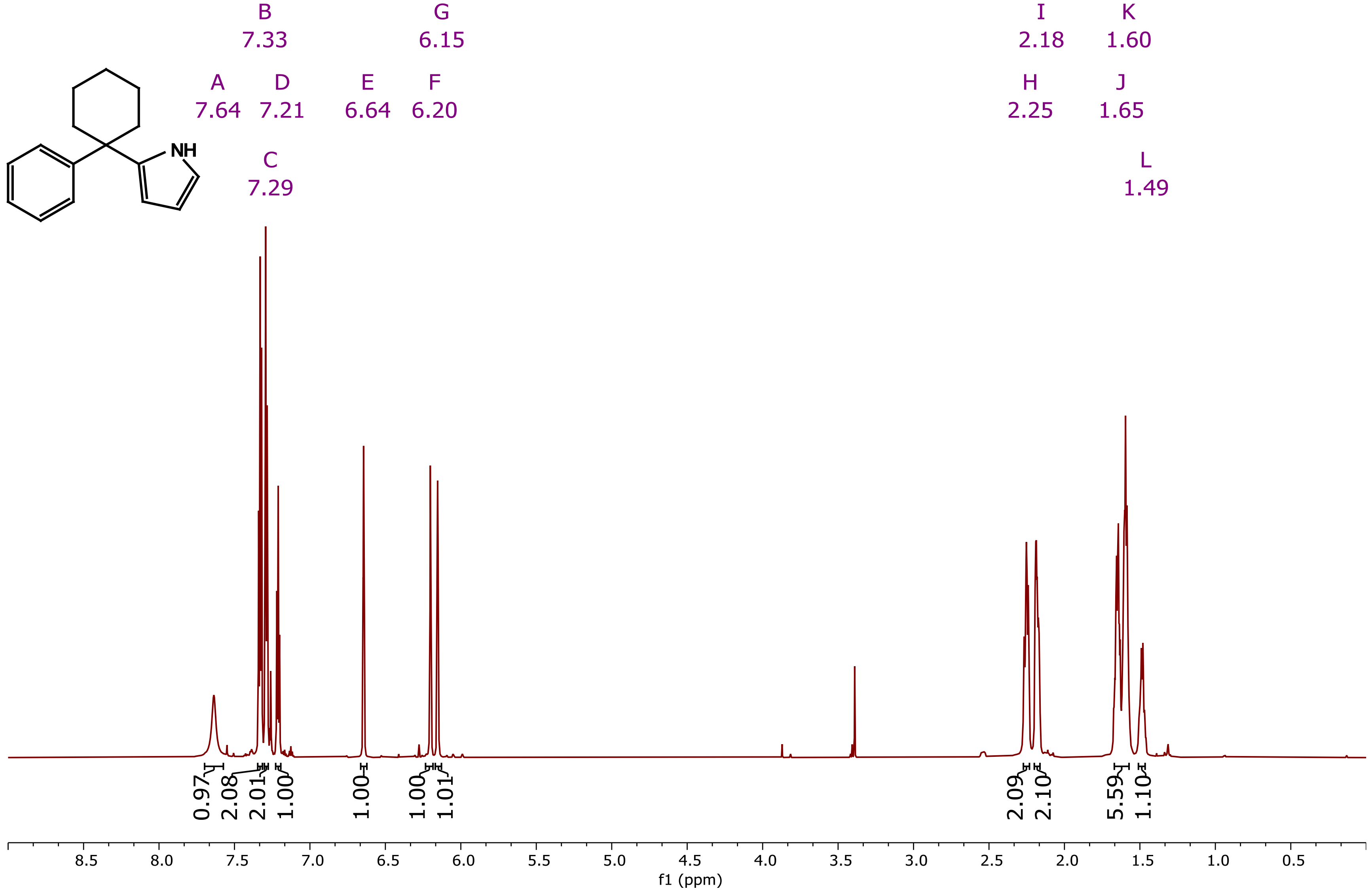

[5b] 2-(1-phenylcyclohexyl)-1H-pyrrole
13C NMR collected at 201.27 MHz in CDCl3

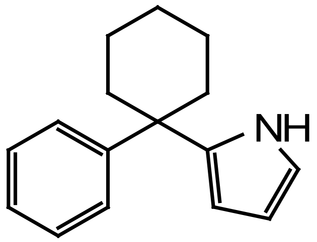

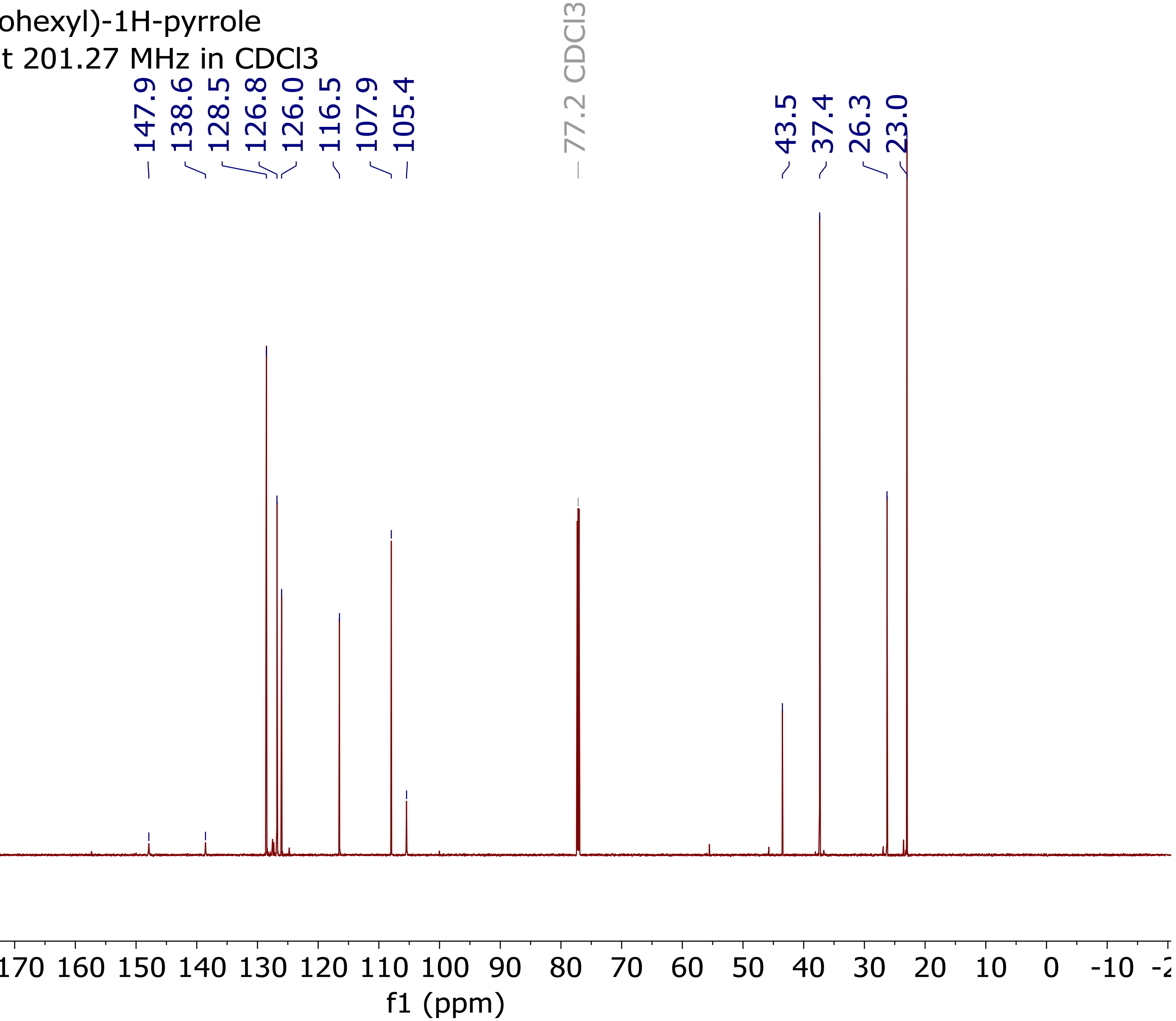

[5b'] 3-(1-phenylcyclohexyl)-1H-pyrrole
1H NMR at 800.34 MHz in CDCl3

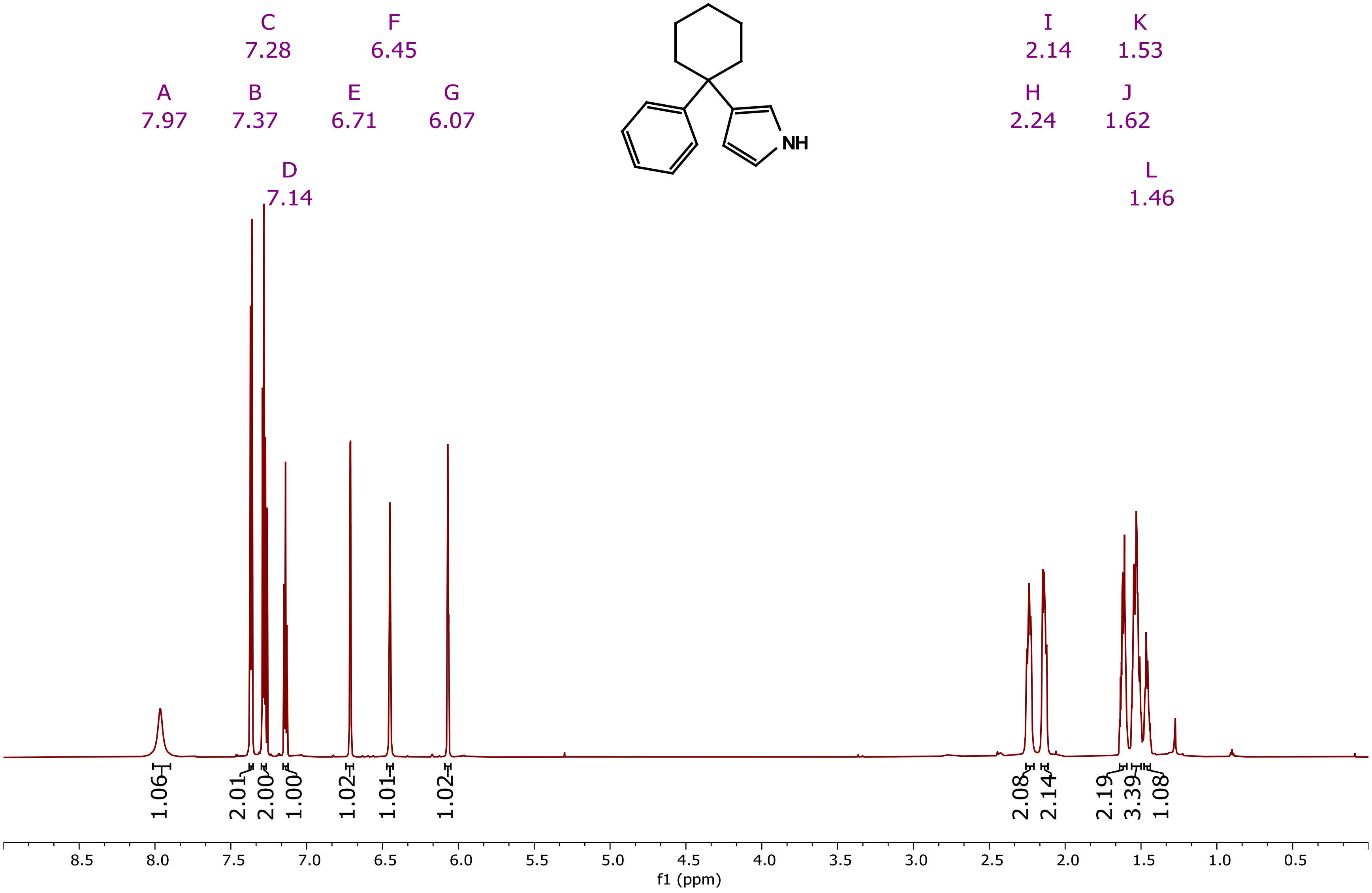

[5b'] - 3-(1-phenylcyclohexyl)-1H-pyrrole
13C NMR collected at 201.27 MHz in CDCl3

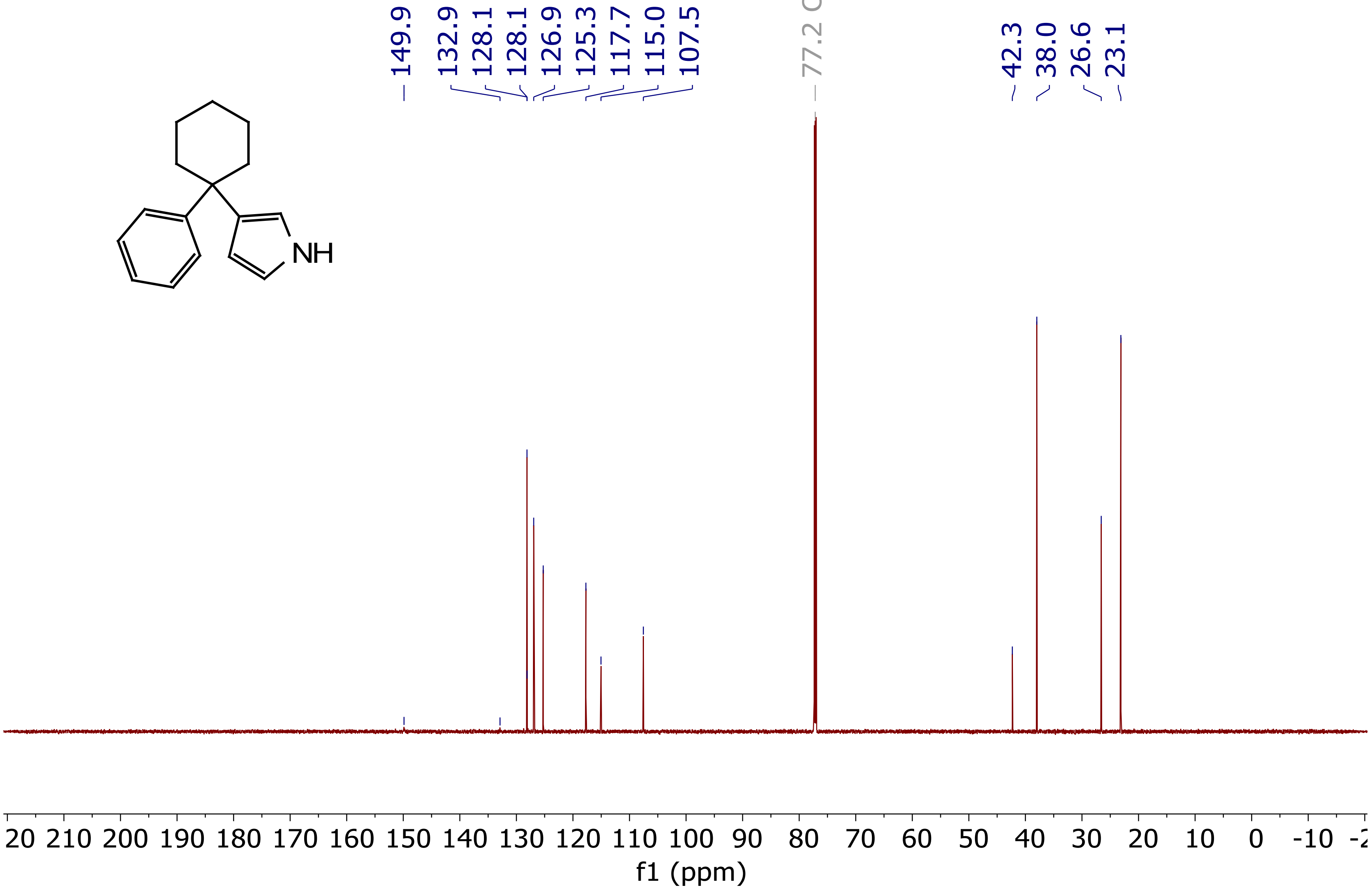

[5bII] 5-chloro-2-(1-phenylcyclohexyl)pyridine
1H NMR collected at 800.34 MHz in CDCl3

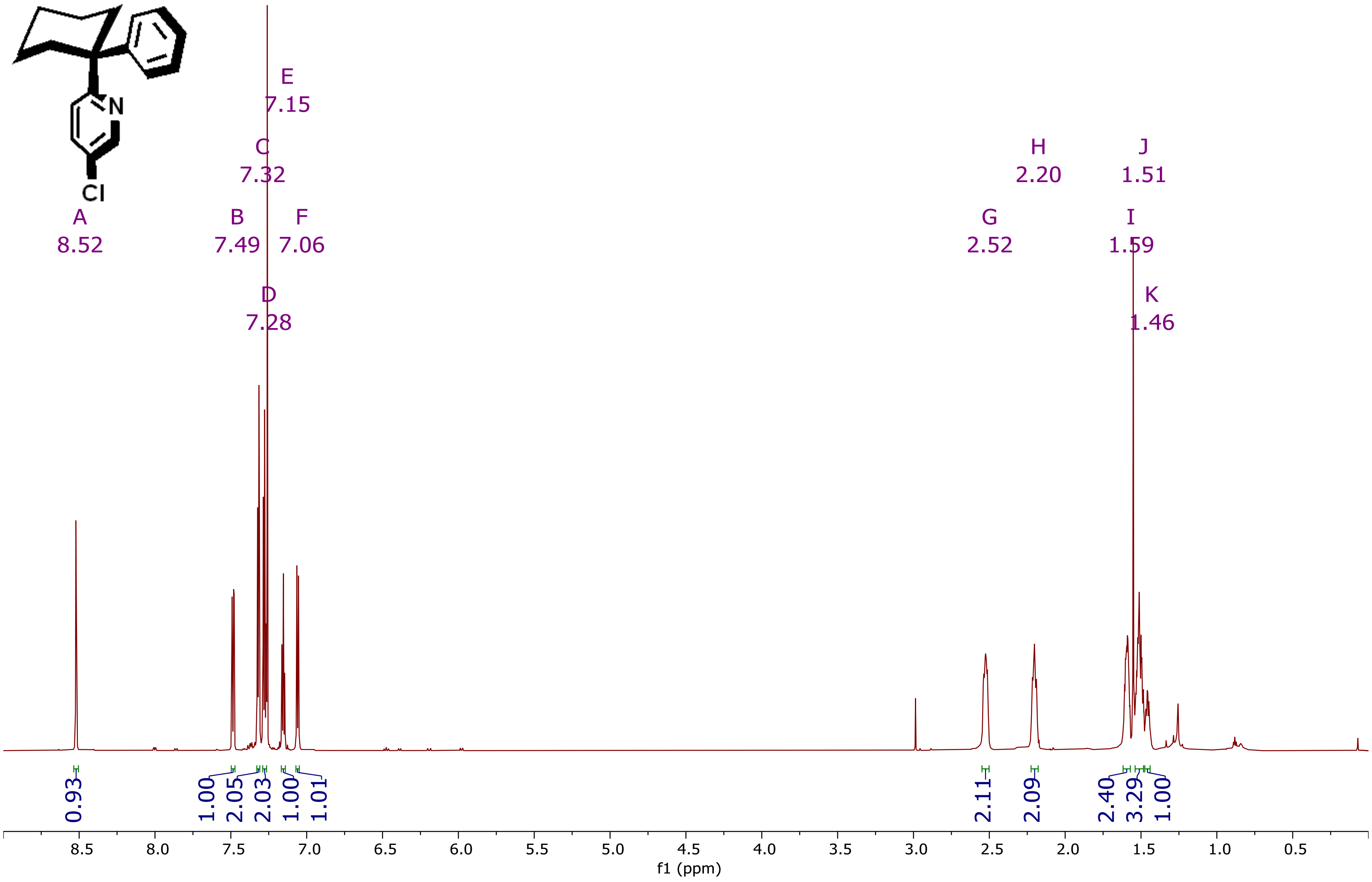

[5bII] 5-chloro-2-(1-phenylcyclohexyl)pyridine
13C NMR collected at 201.27 MHz in CDCl3

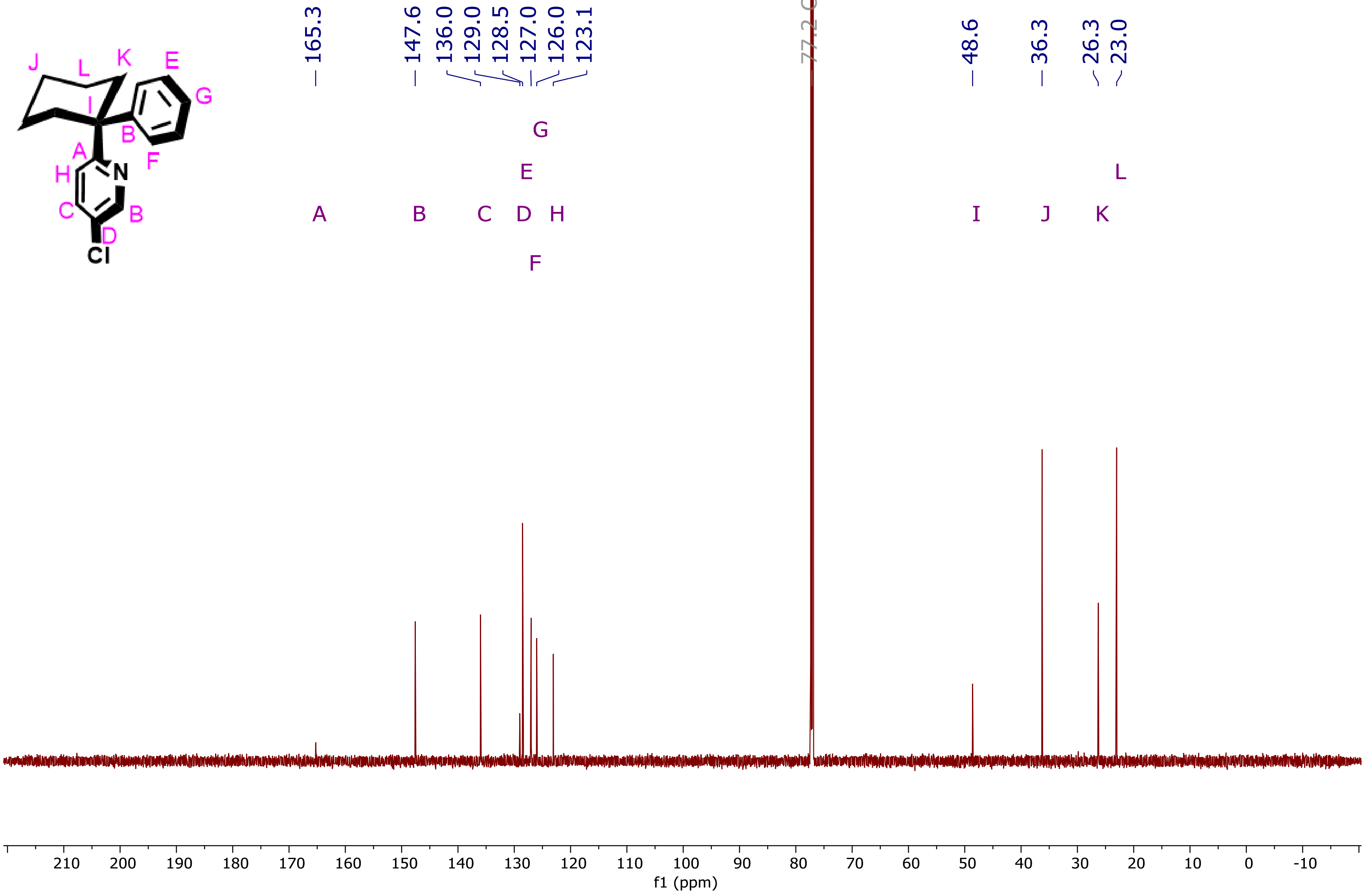

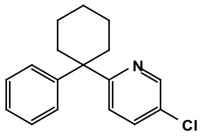

N
Cl

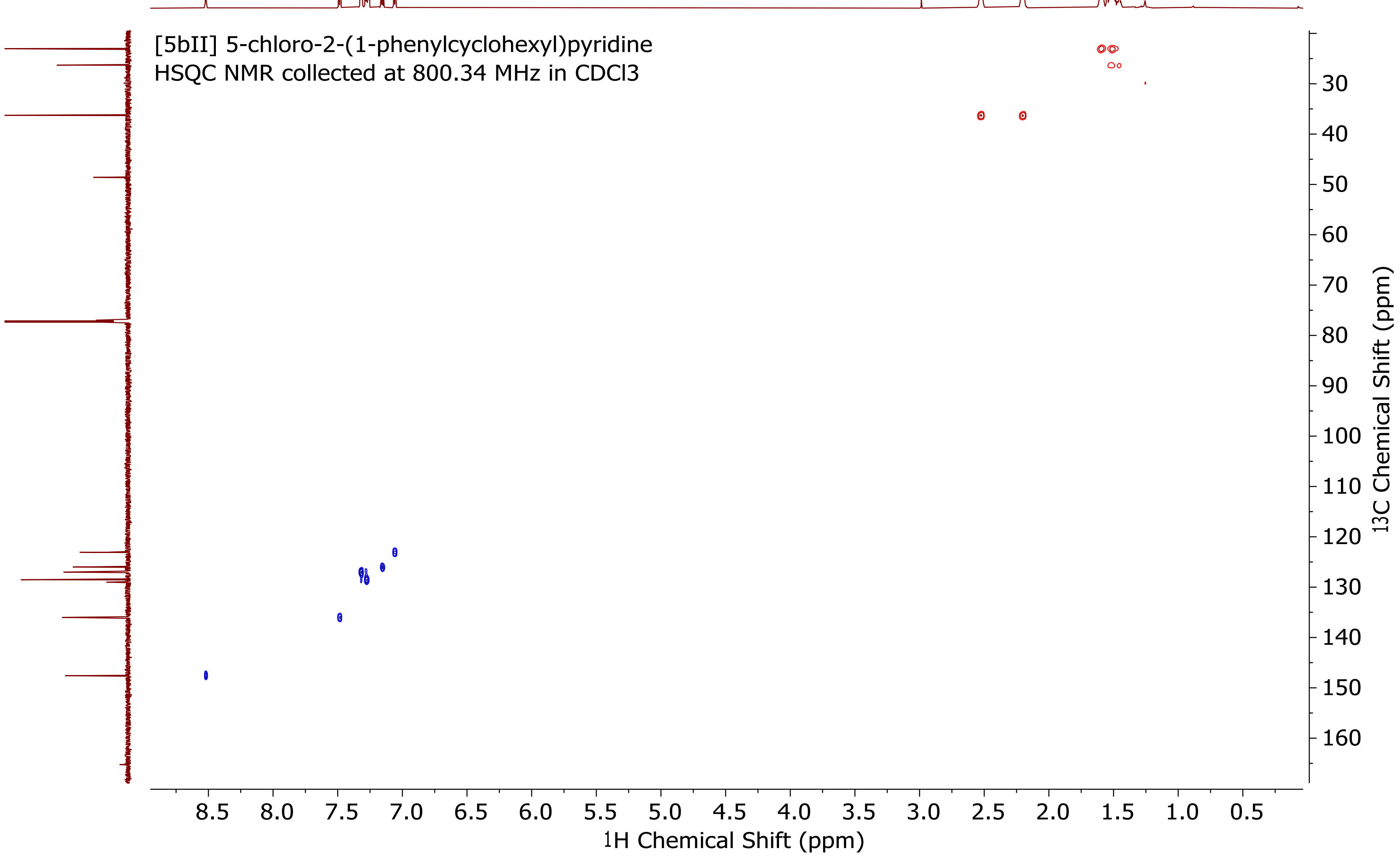

[5bII] 5-chloro-2-(1-phenylcyclohexyl)pyridine
HSQC NMR collected at 800.34 MHz in CDCl3
1H Chemical Shift (ppm)
13C Chemical Shift (ppm)
8.5 8.0 7.5 7.0 6.5 6.0 5.5 5.0 4.5 4.0 3.5 3.0 2.5 2.0 1.5 1.0 0.5
30 40 50 60 70 80 90 100 110 120 130 140 150 160

[6b] 6-fluoro-3-(1-phenylcyclohexyl)-1H-indole

1H NMR collected at 800.34 MHz in CDCl3

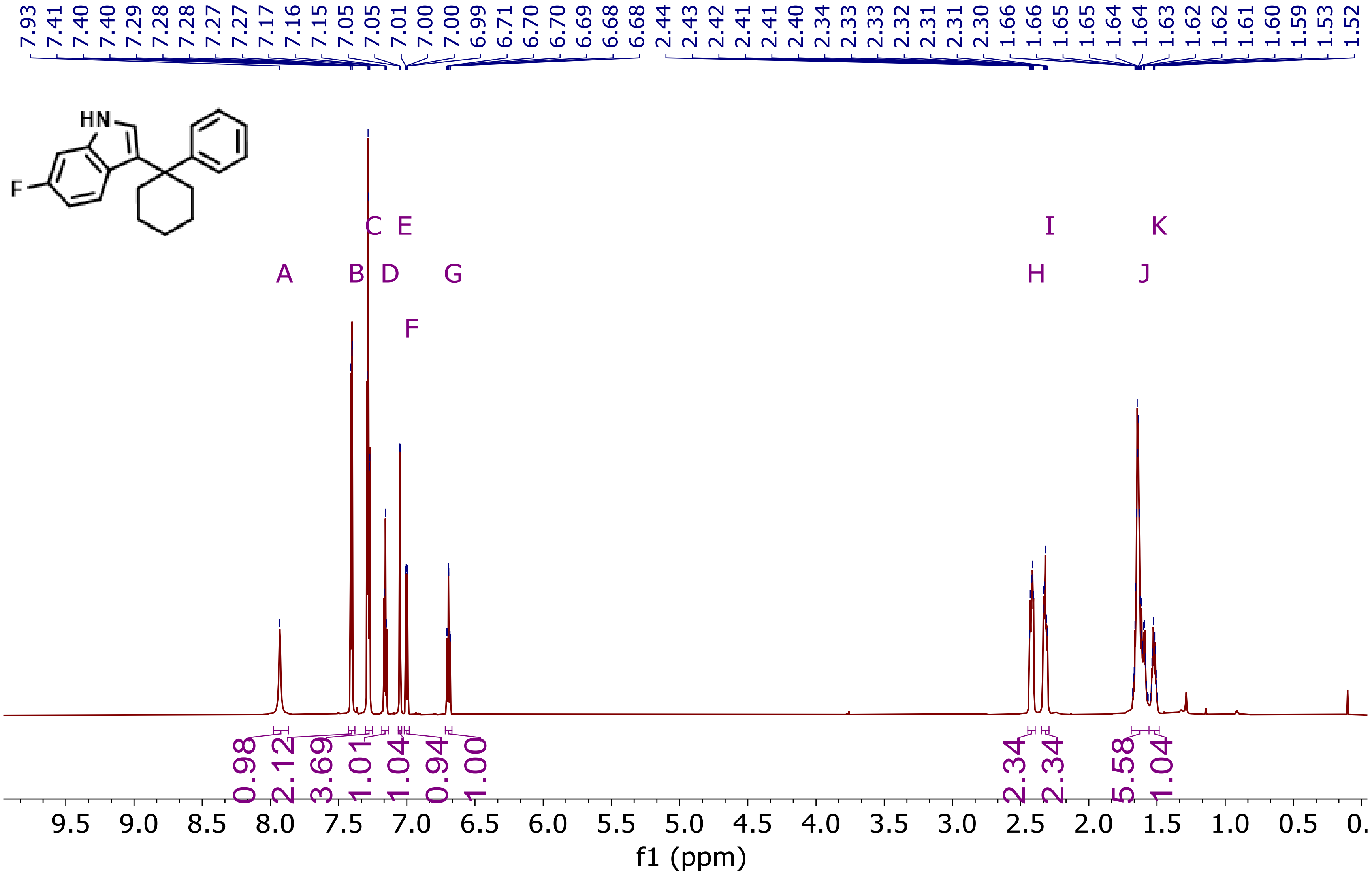

[6b] 6-fluoro-3-(1-phenylcyclohexyl)-1H-indole
13C NMR collected at 201.27 MHz in CDCl3

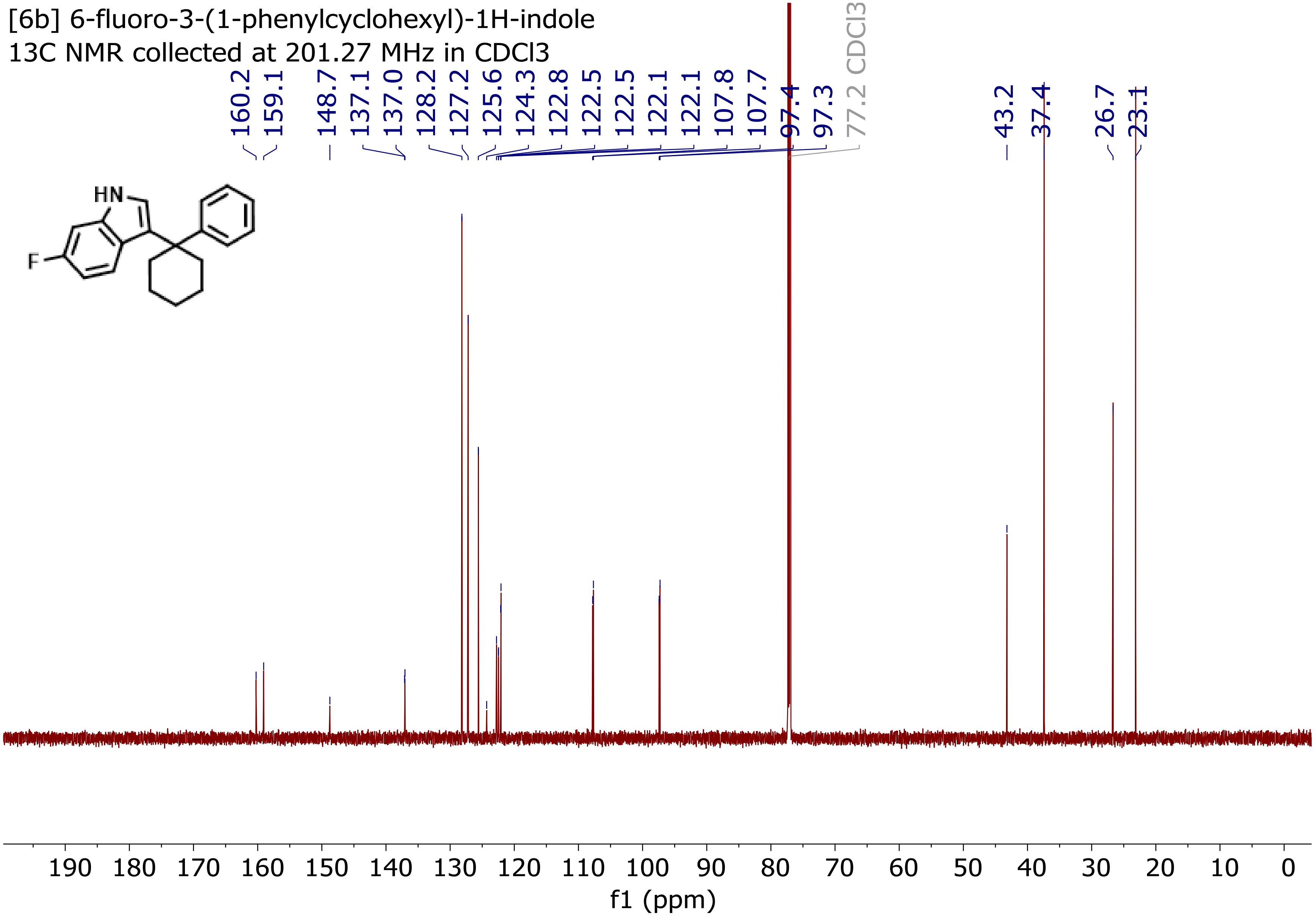

[6b] 6-fluoro-3-(1-phenylcyclohexyl)-1H-indole
19F NMR collected at 753.00 MHz in CDCl3

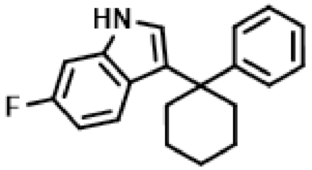

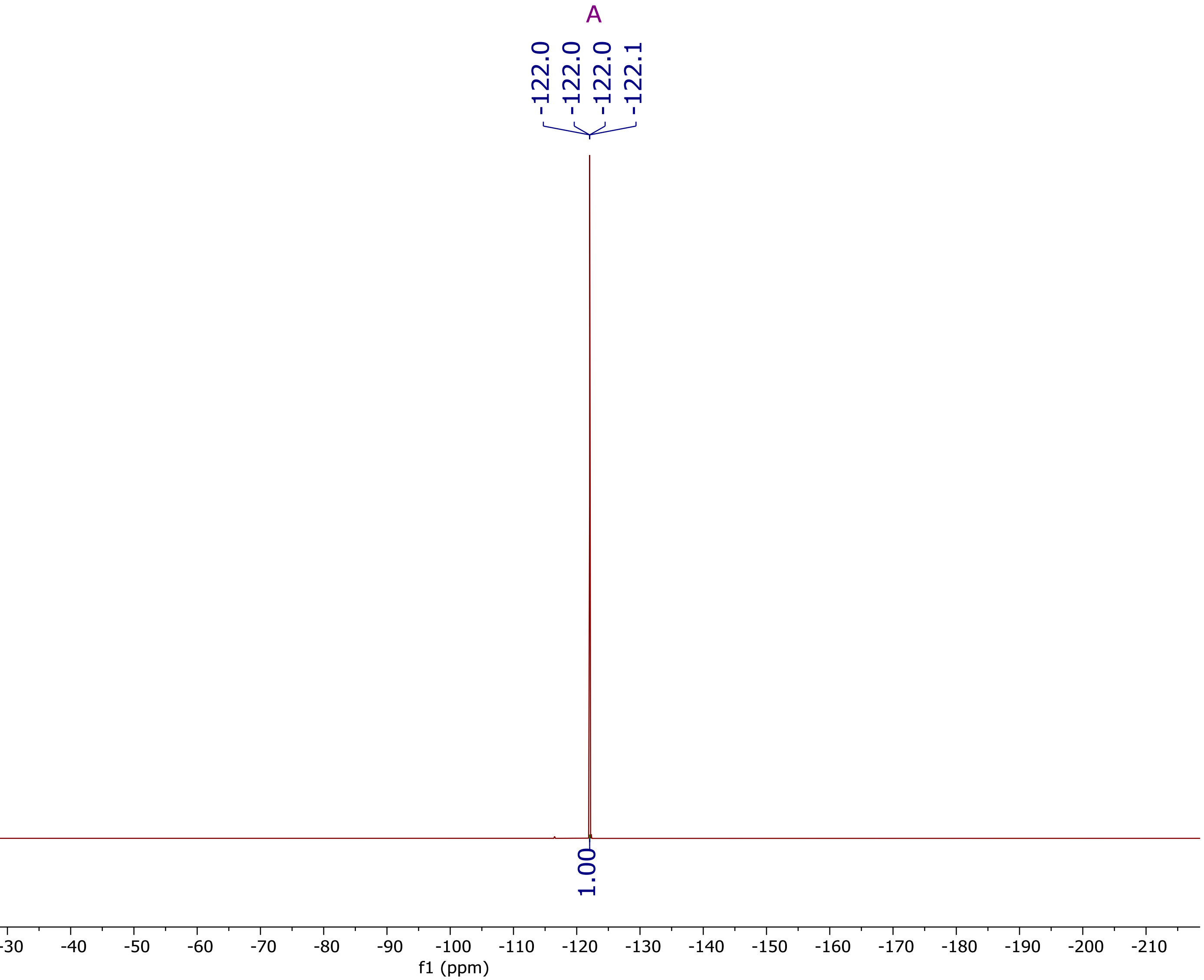

[7b] 3-(1-phenylcyclohexyl)benzofuran
1H NMR collected at 800.34 MHz in CDCl3

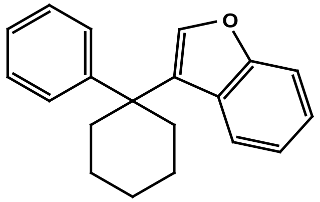

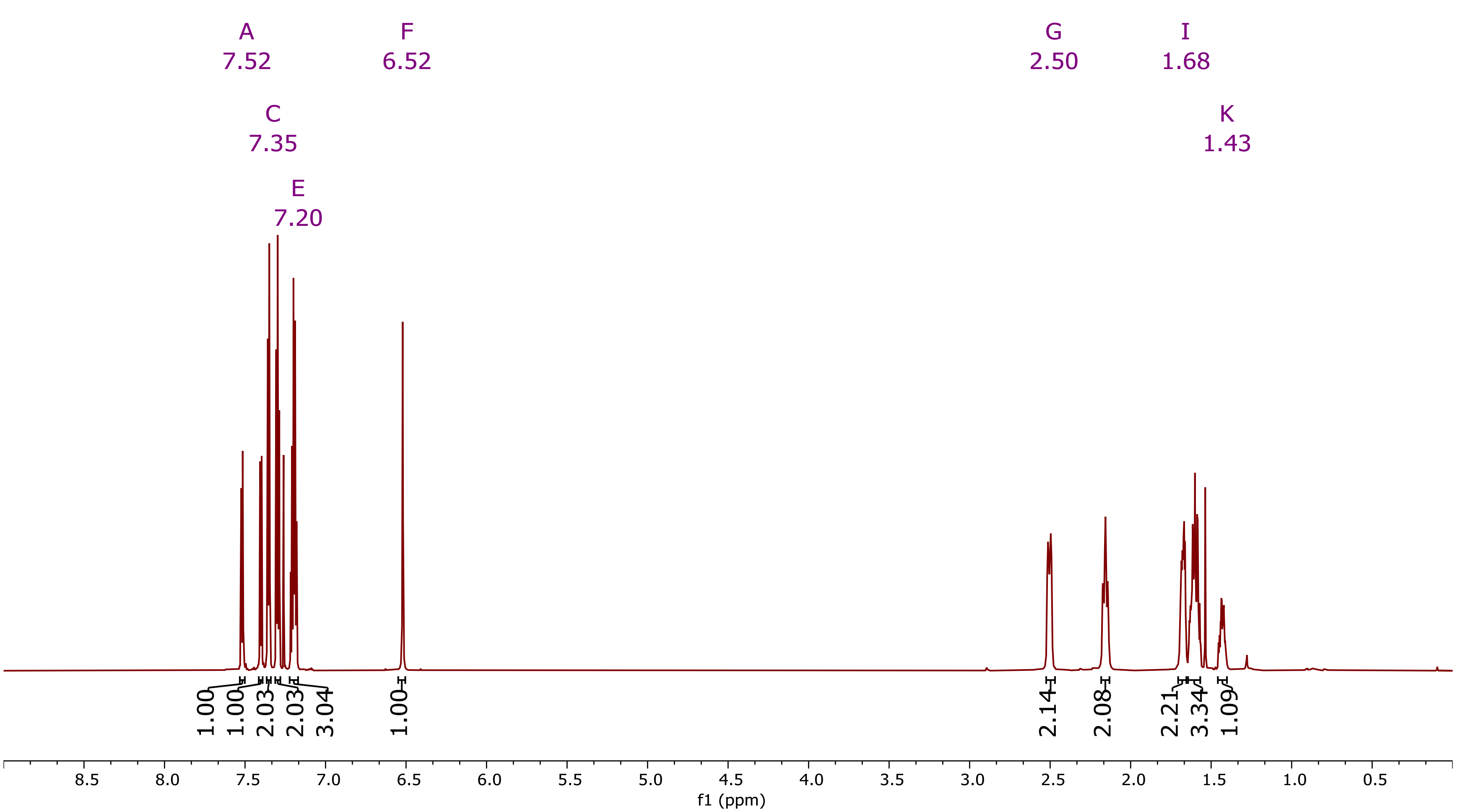

[7b] 3-(1-phenylcyclohexyl)benzofuran
13C NMR collected at 201.27 MHz in CDCl3

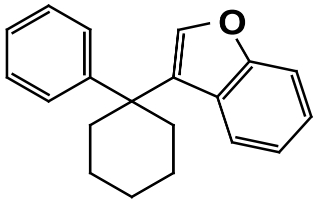

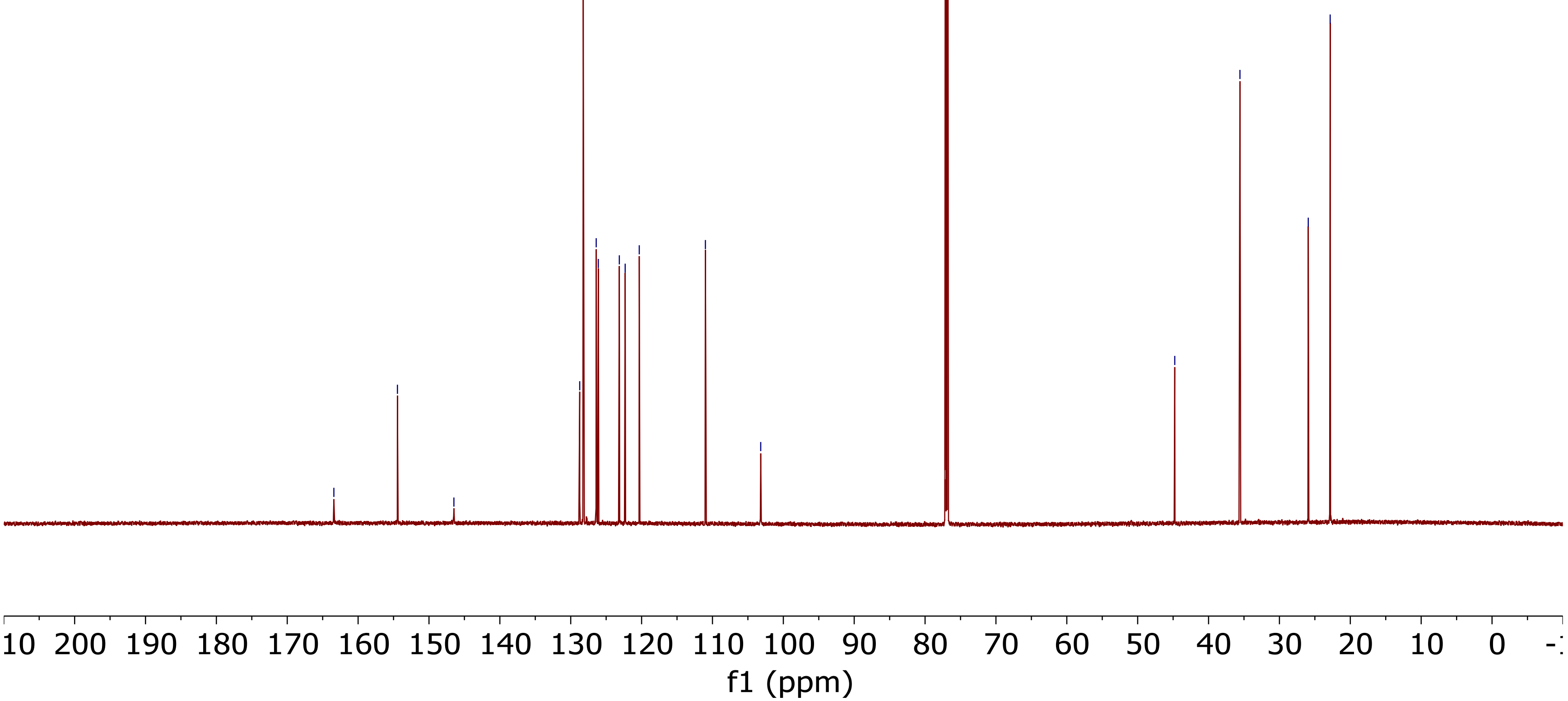

[8b] ((4,6-dimethoxy-1,3-phenylene)bis(cyclohexane-1,1-diyl))dibenzene
1H NMR collected at 800.34 MHz in CDCl3

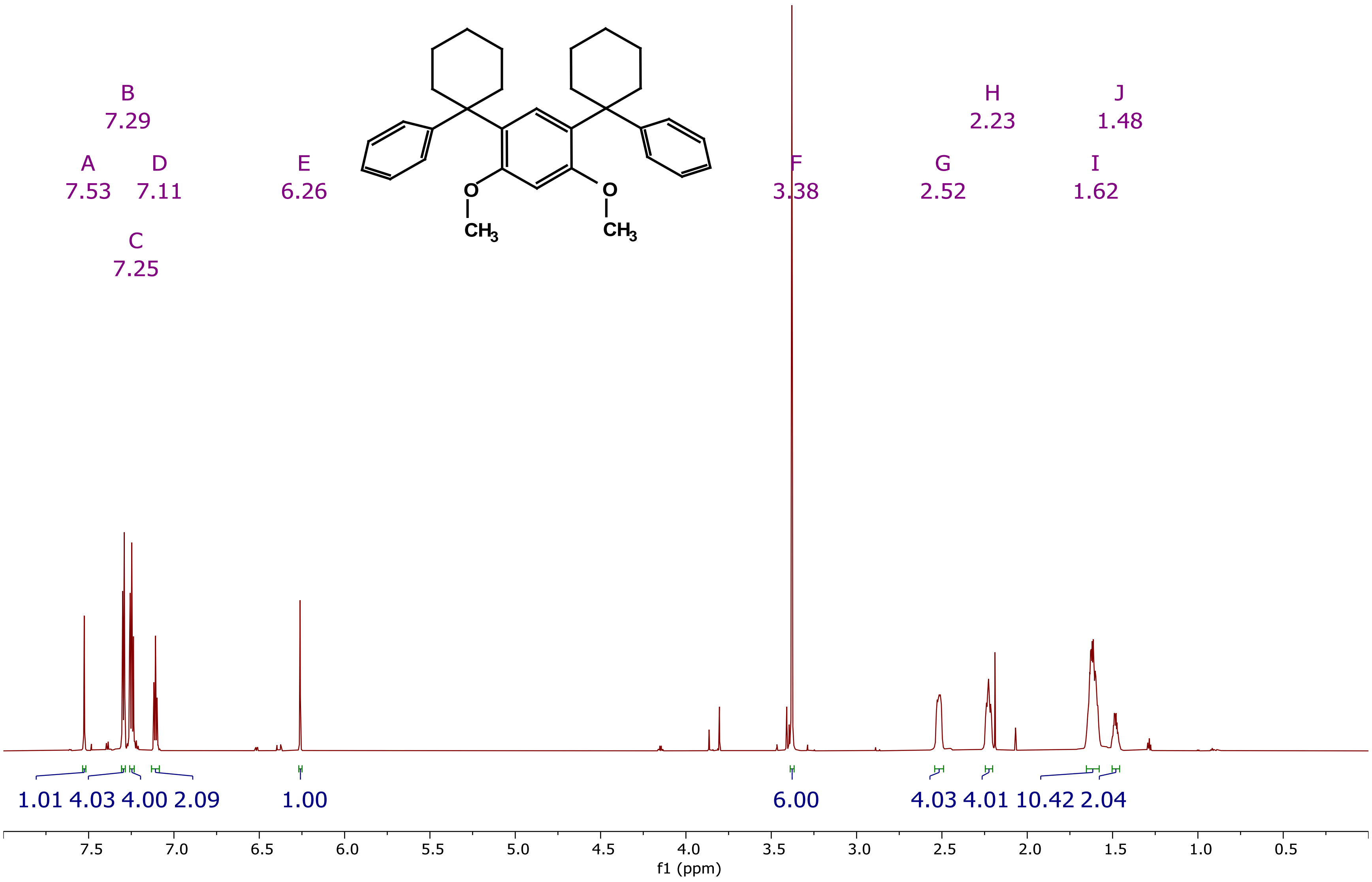

[8b] ((4,6-dimethoxy-1,3-phenylene)bis(cyclohexane-1,1-diyl))dibenzene
13C NMR collected at 201.27 MHz in CDCl3

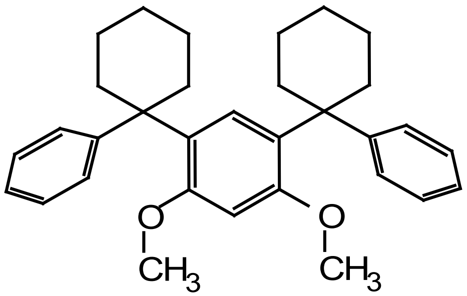

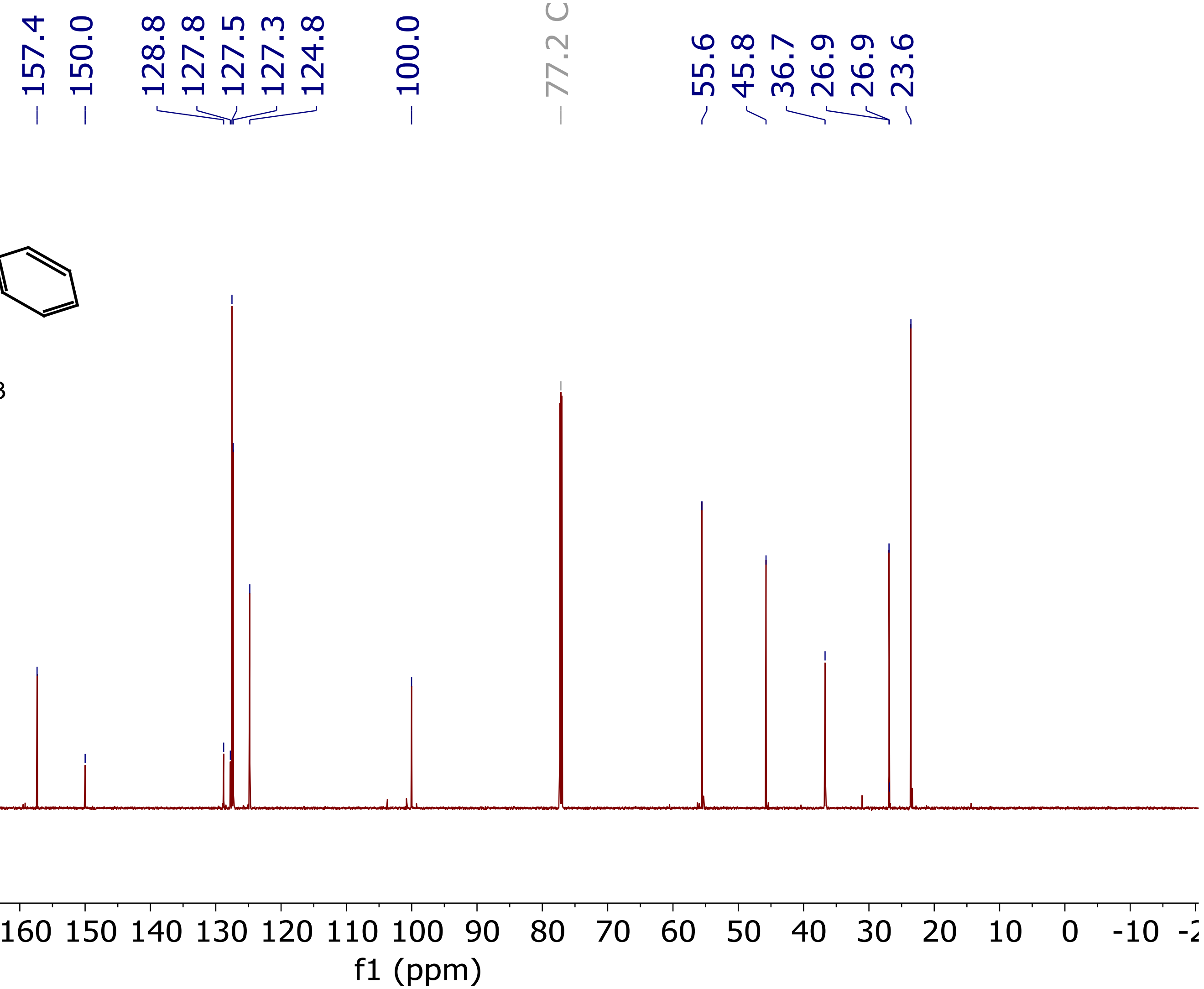

[9b] N,N-dimethyl-4-(1-phenylcyclohexyl)aniline
1H NMR collected at 800.34 MHz in CDCl3

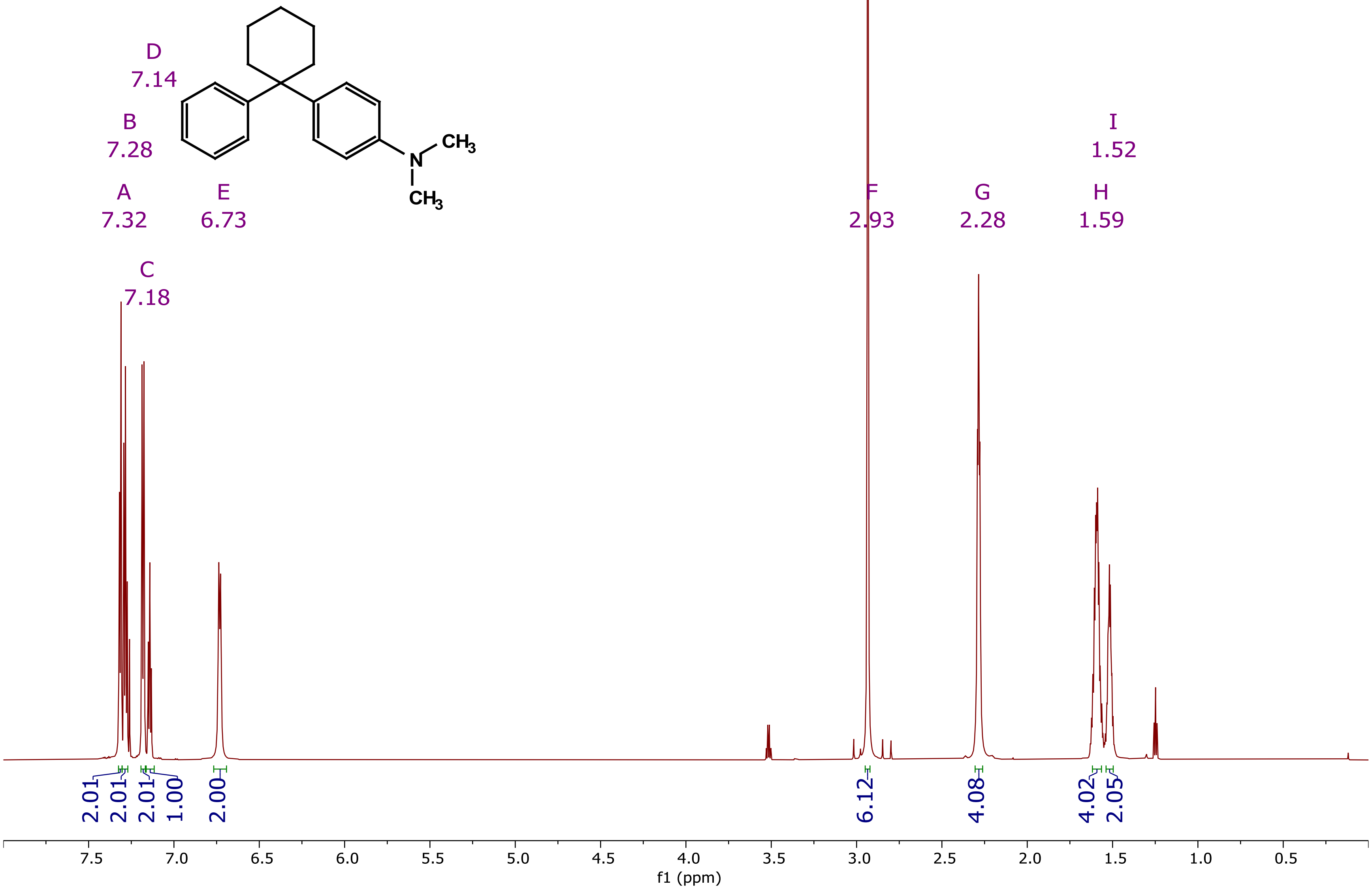

[9b] N,N-dimethyl-4-(1-phenylcyclohexyl)aniline
13C NMR collected at 201.27 MHz in CDCl3

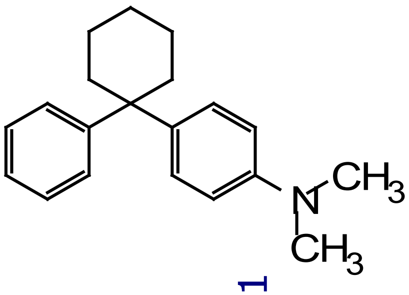

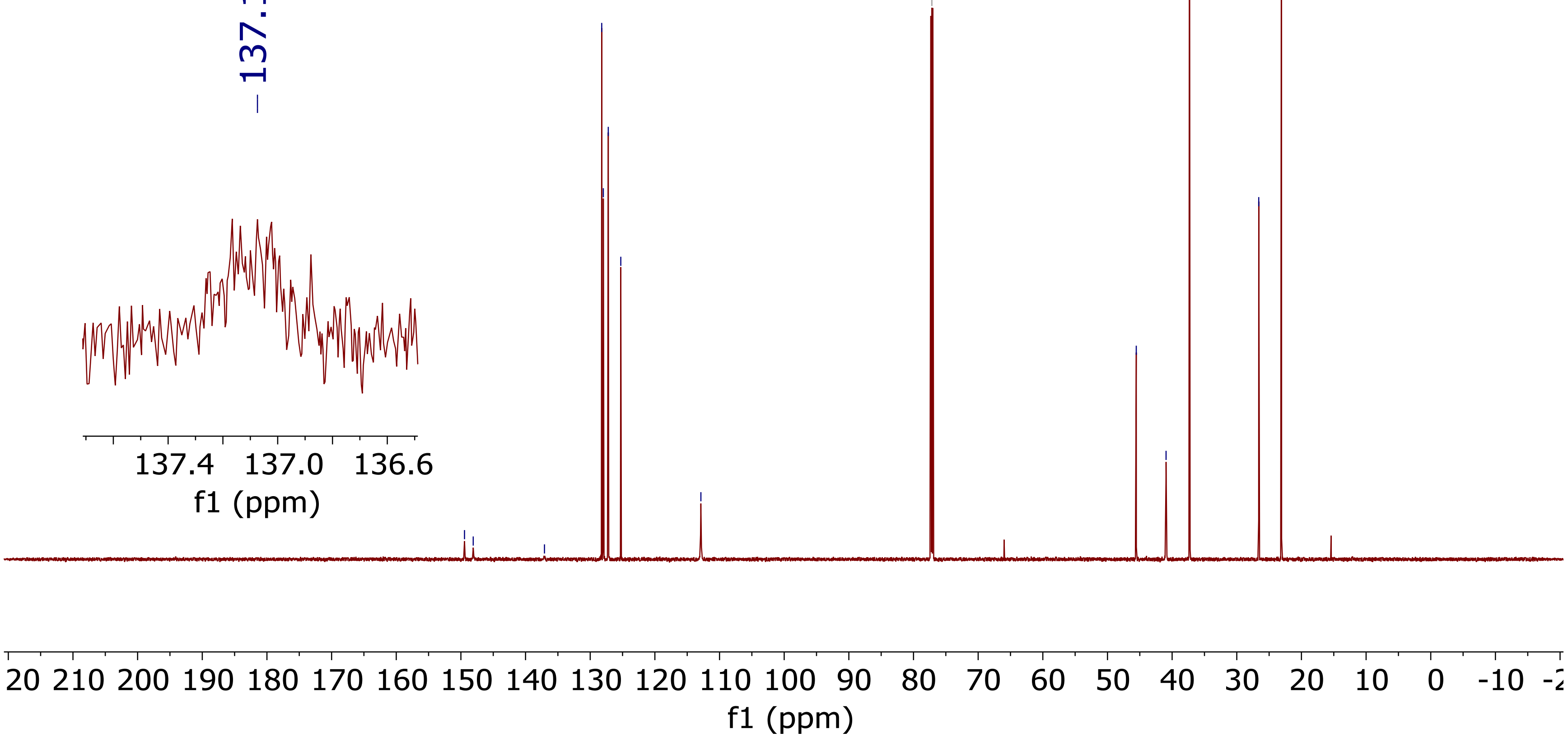

[10b’] N-(1-phenylcyclohexyl)aniline
1H NMR collected at 800.34 MHz in CDCl3

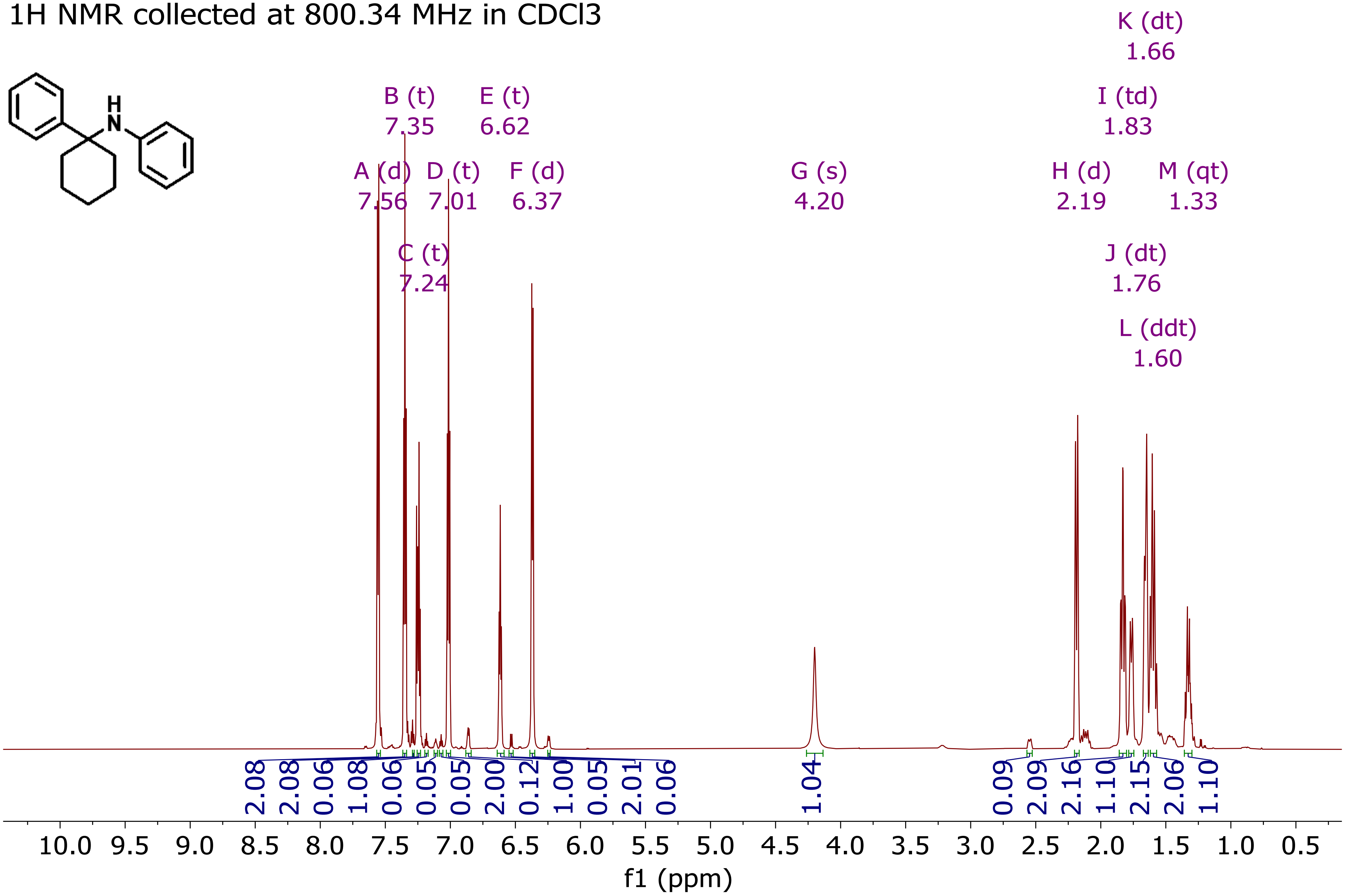

sss

[10b′] N-(1-phenylcyclohexyl)aniline
13C NMR collected at 201.27 MHz in CDCl3

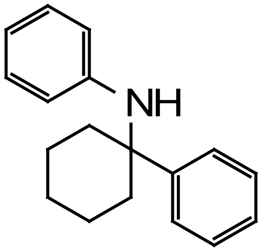

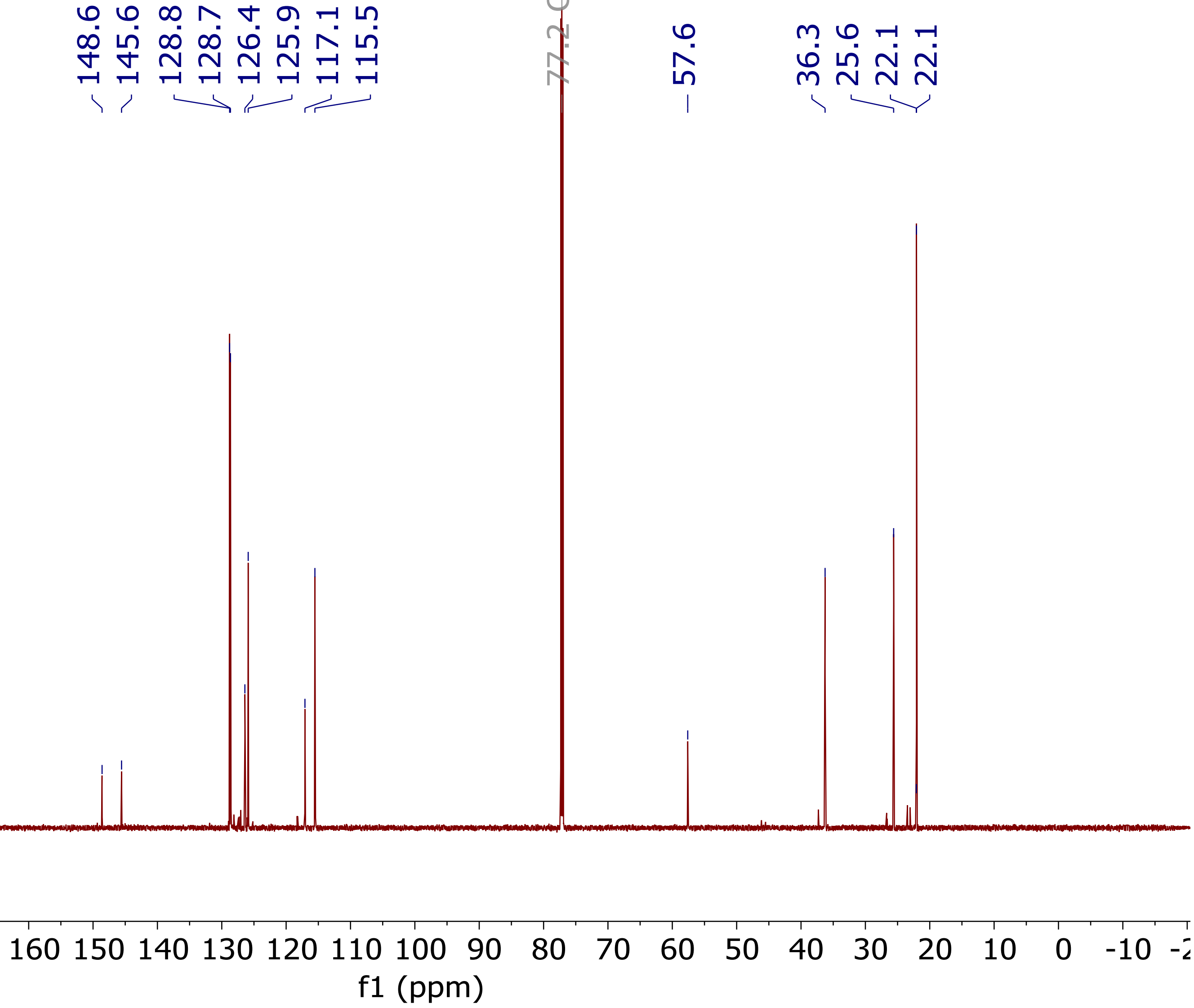

[10b] 4-(1-phenylcyclohexyl)aniline
1H NMR collected at 800.34 MHz in CDCl3

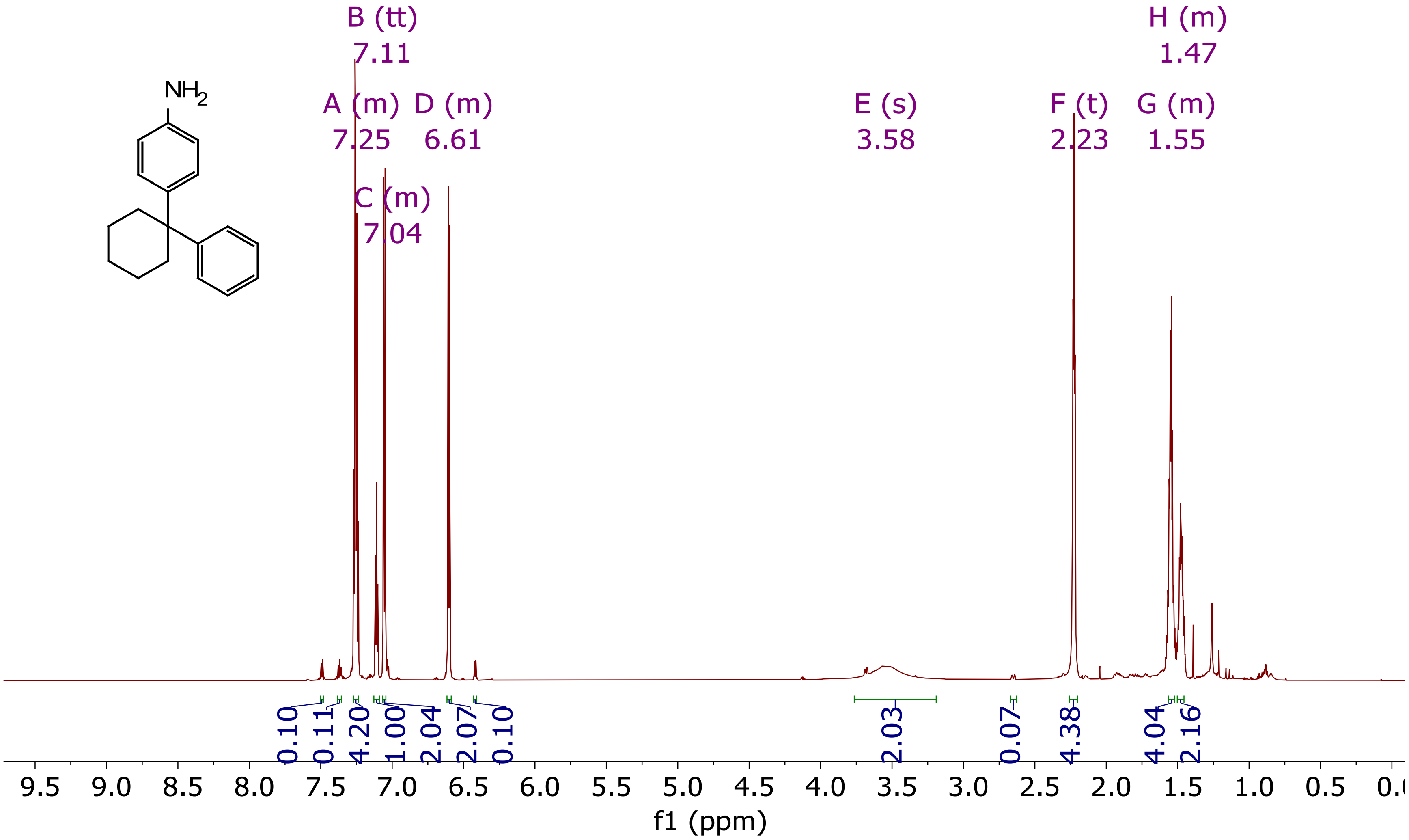

[10b] 4-(1-phenylcyclohexyl)aniline
13C NMR collected at 201.27 MHz in CDCl3

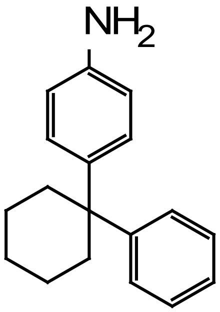

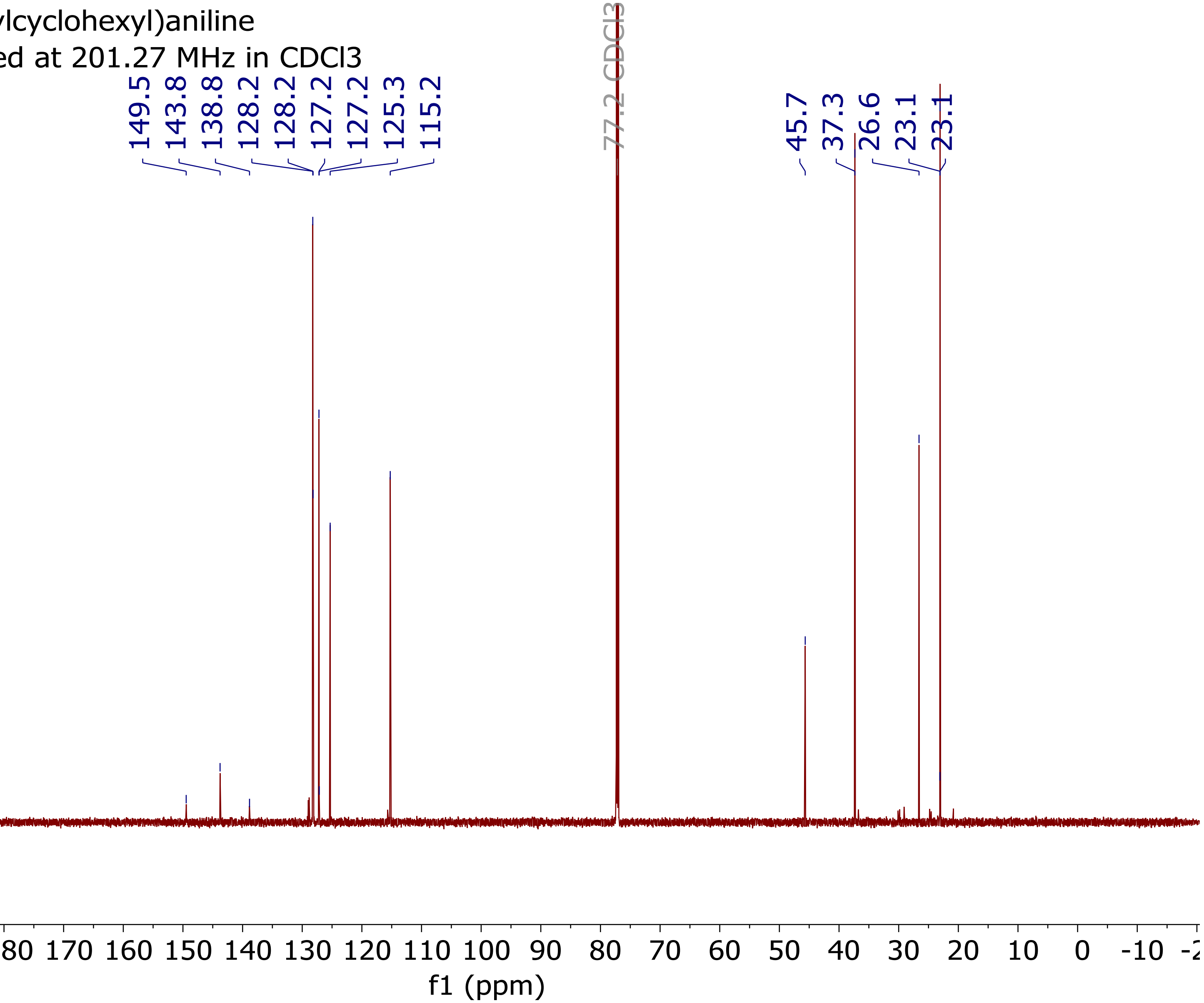

[11b] (1-(2-phenylallyl)cyclohexyl)benzene
1H NMR collected at 800.34 MHz in CDCl3

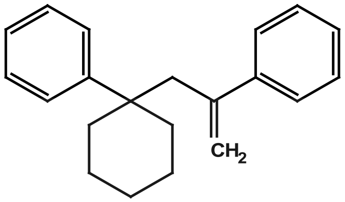

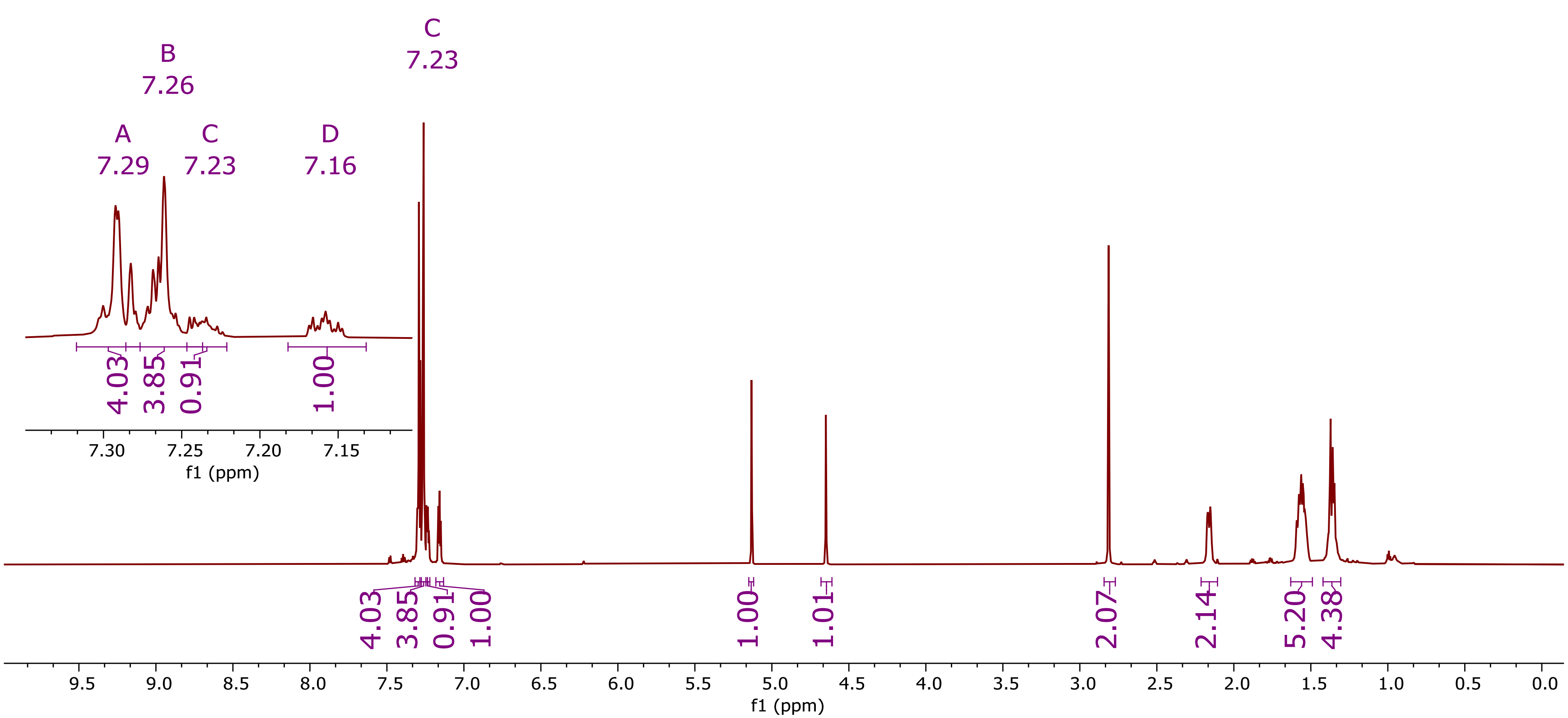

[11b] (1-(2-phenylallyl)cyclohexyl)benzene

13C NMR collected at 201.27 MHz in CDCl3

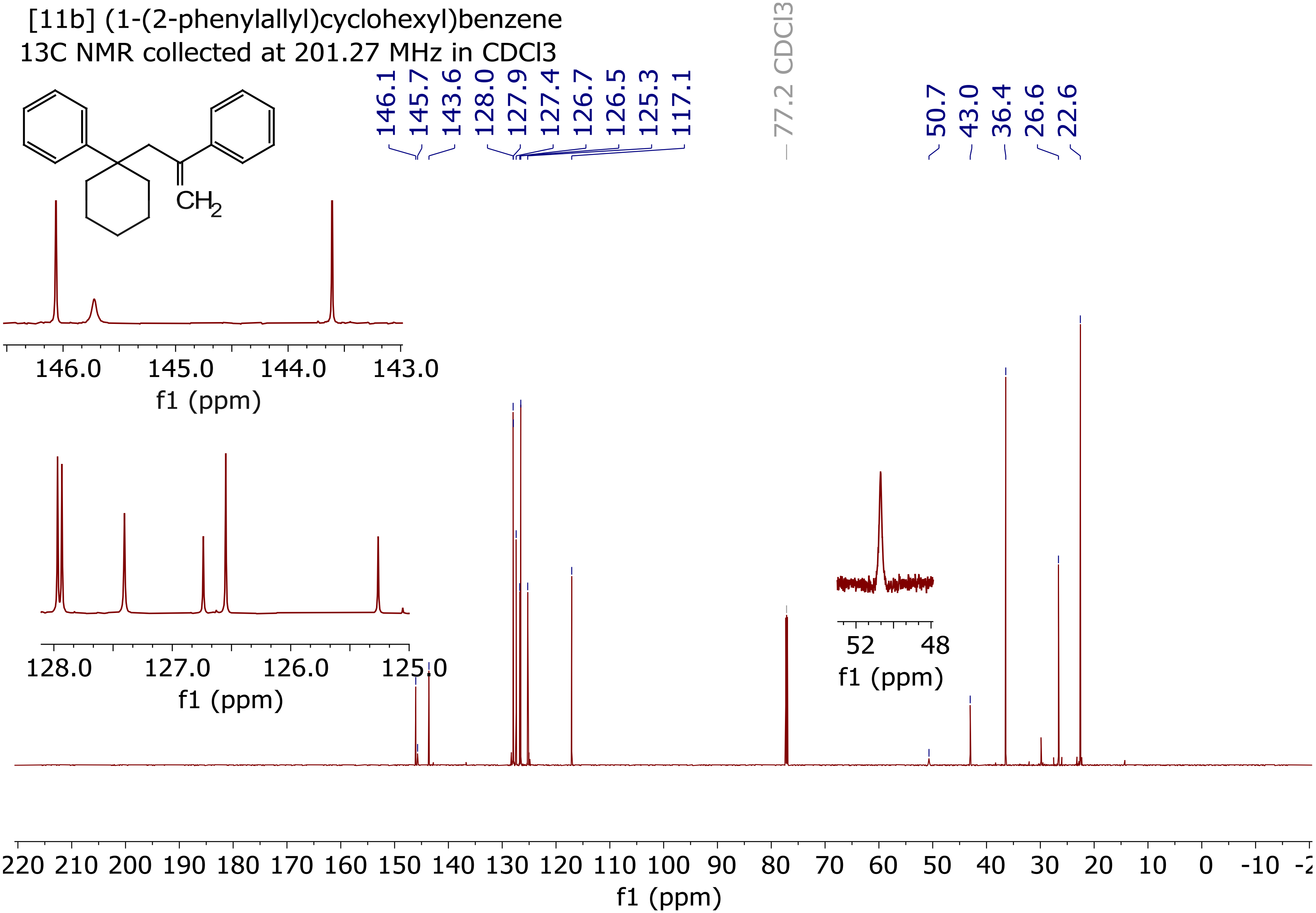

[12b] 4-(1-phenylcyclohexyl)phenol
1H NMR collected at 800.34 MHz in CDCl3

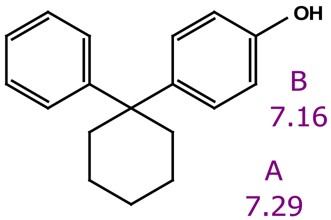

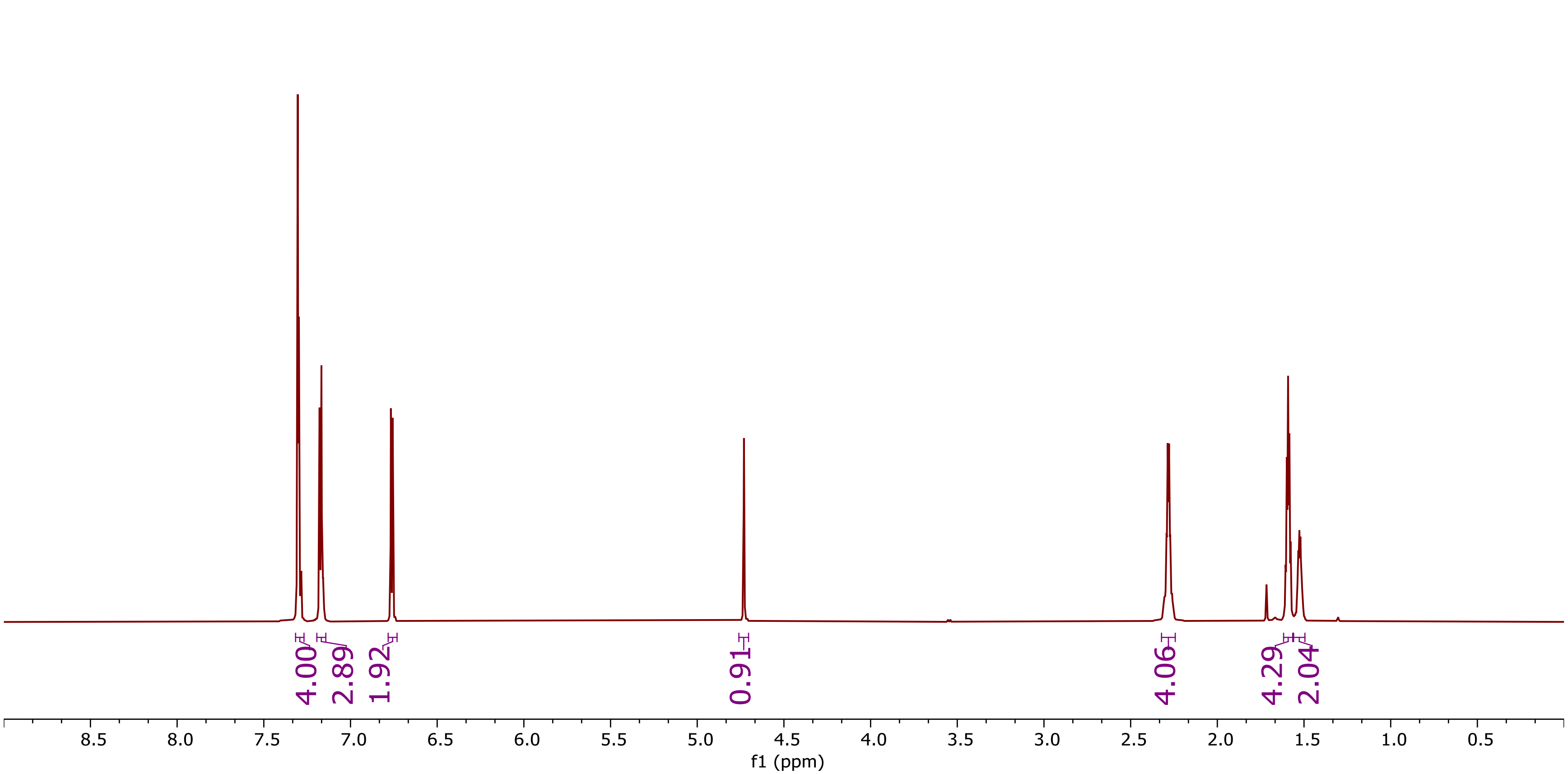

[12b] 4-(1-phenylcyclohexyl)phenol
13C NMR collected at 201.27 MHz in CDCl3

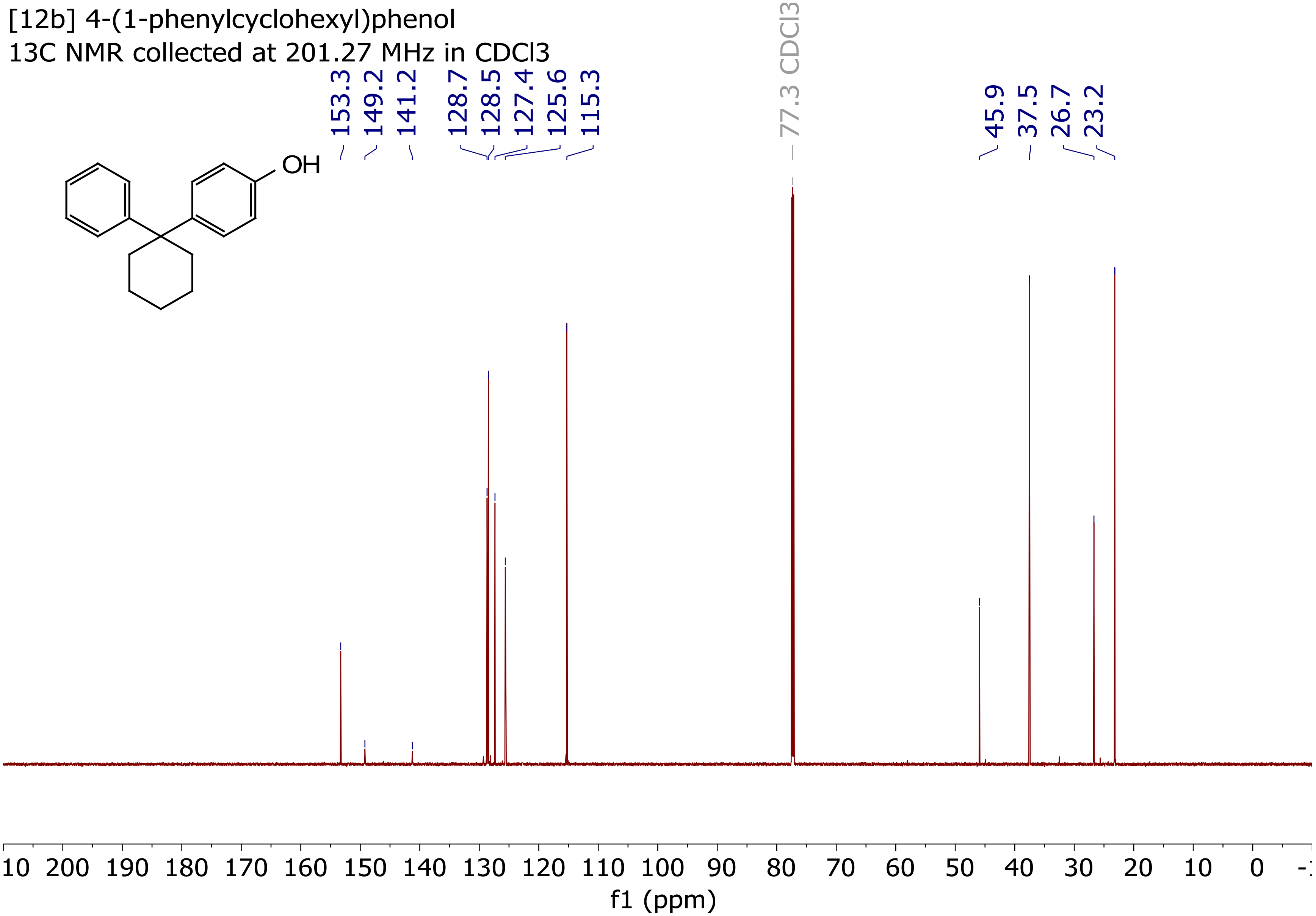

[13b] 4-(1-phenylcyclohexyl)benzene-1,3-diol
1H NMR collected at 800.34 MHz in CDCl3

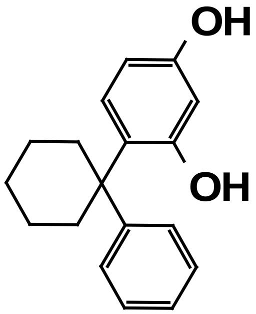

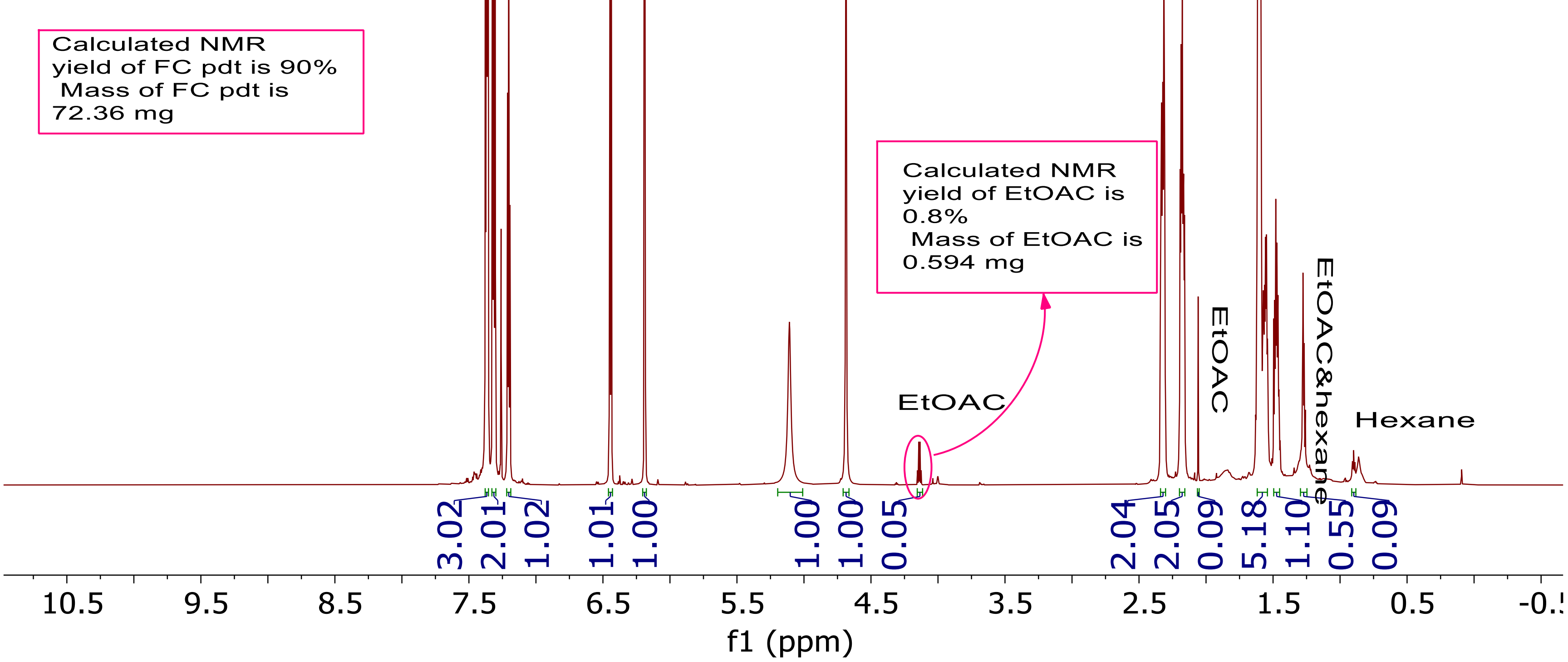

[13b] 4-(1-phenylcyclohexyl)benzene-1,3-diol
13C NMR collected at 201.27 MHz in CDCl3

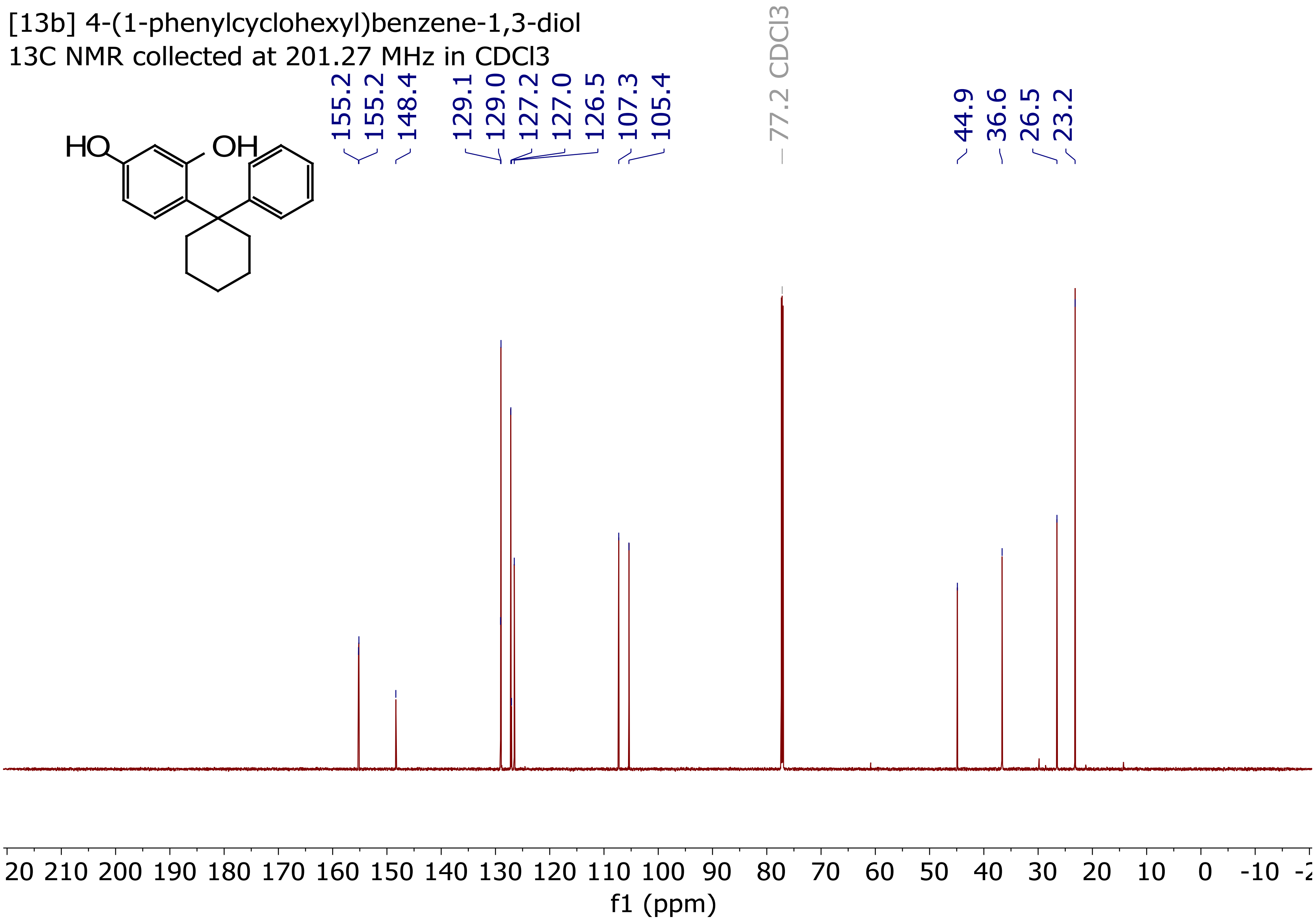

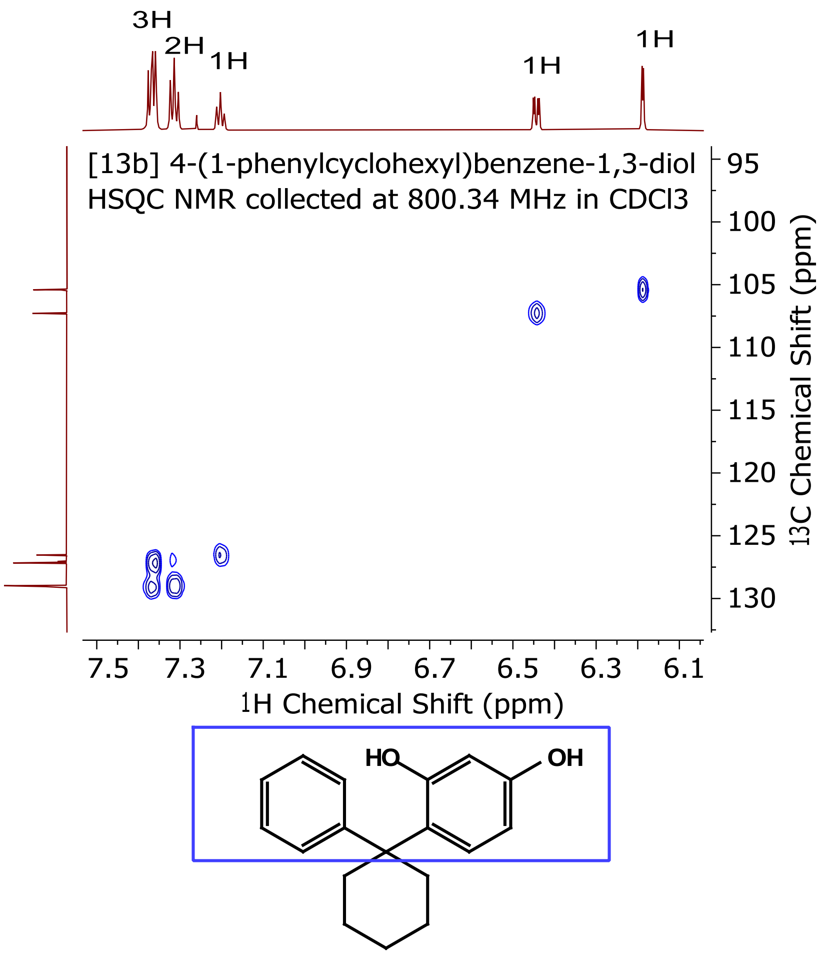
3H
2H
1H
1H
1H
[13b] 4-(1-phenylcyclohexyl)benzene-1,3-diol
HSQC NMR collected at 800.34 MHz in CDCl3
95
100
105
110
115
120
125
130
13C Chemical Shift (ppm)
7.5
7.3
7.1
6.9
6.7
6.5
6.3
6.1
1H Chemical Shift (ppm)
HO
OH

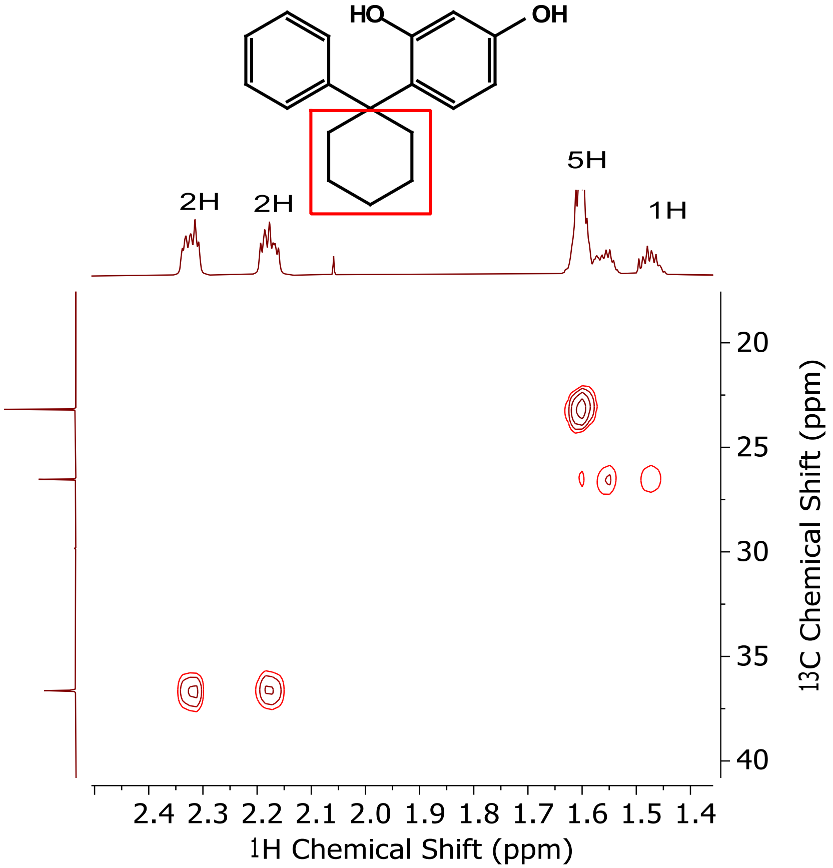
HO
OH
2H
2H
5H
1H
20
25
30
35
40
13C Chemical Shift (ppm)
2.4
2.3
2.2
2.1
2.0
1.9
1.8
1.7
1.6
1.5
1.4
1H Chemical Shift (ppm)

[E15b] 4-((1-phenylcyclohexyl)oxy)-2H-chromen-2-one
1H NMR collected at 800.34 MHz in CDCl3

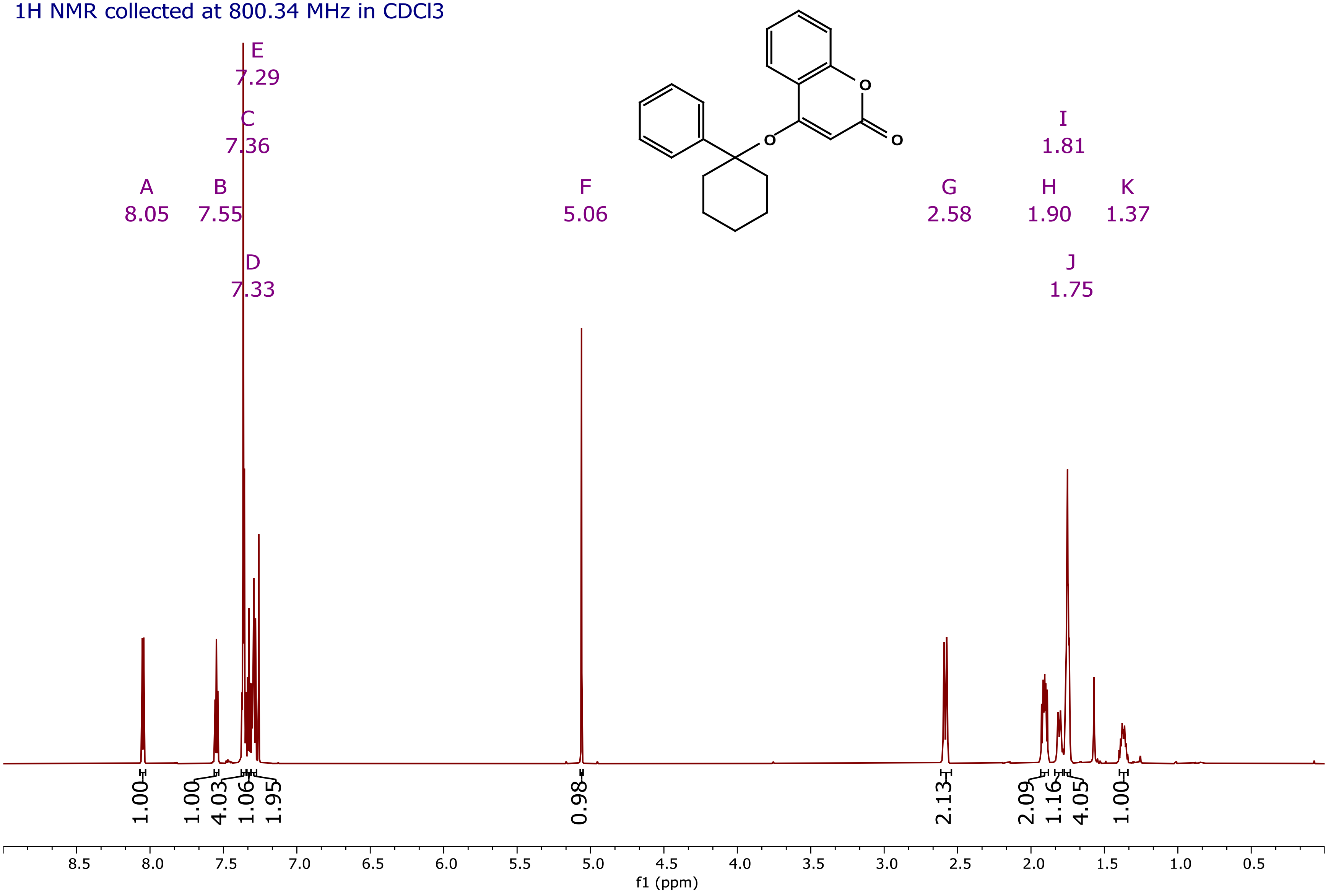

[E15b] 4-((1-phenylcyclohexyl)oxy)-2H-chromen-2-one
13C NMR collected at 201.27 MHz in CDCl3

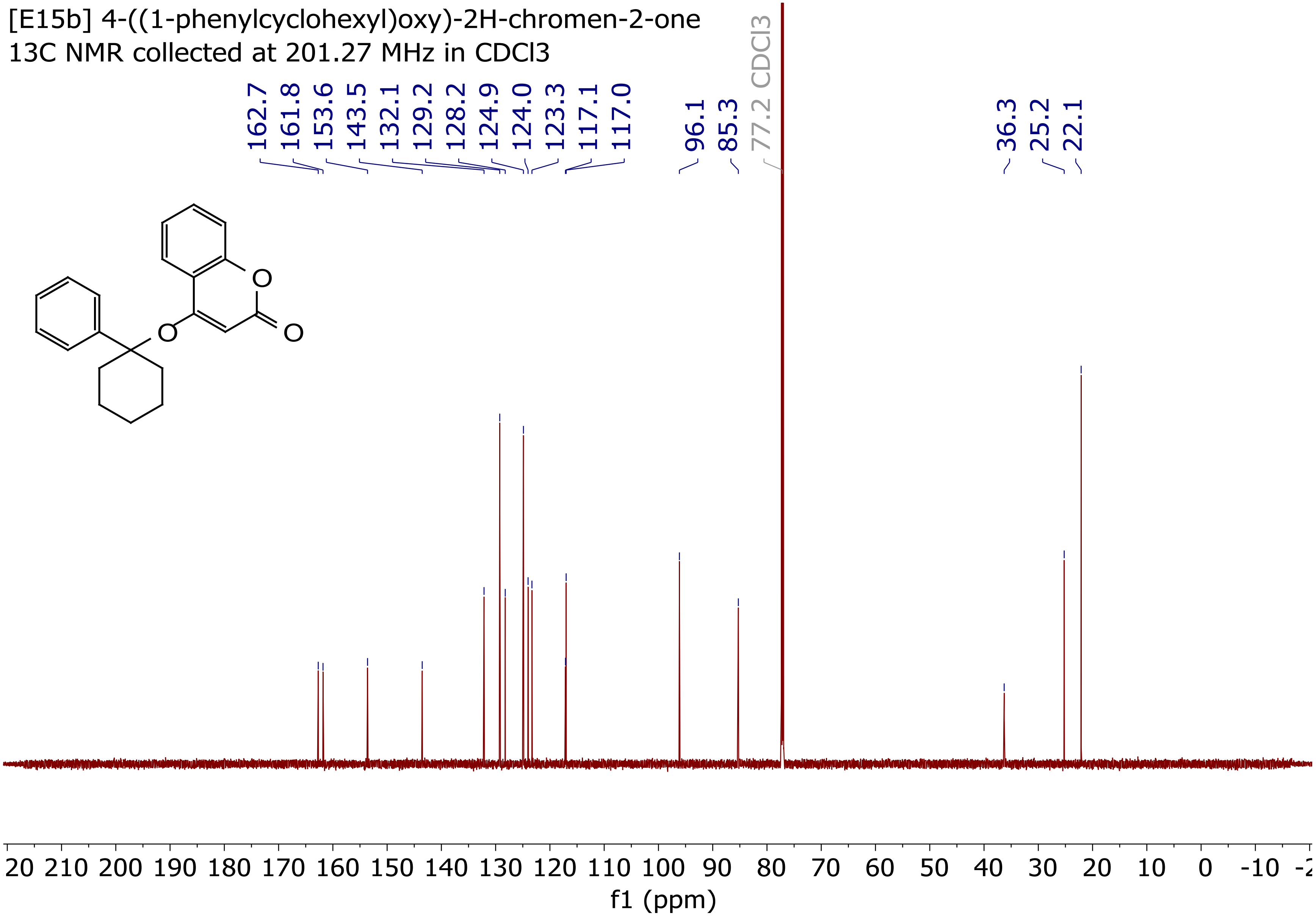

[E15s] tert-butyl 4-((2-oxo-2H-chromen-4-yl)oxy)-4-phenylpiperidine-1-carboxylate
1H NMR collected at 800.34 MHz in CDCl3

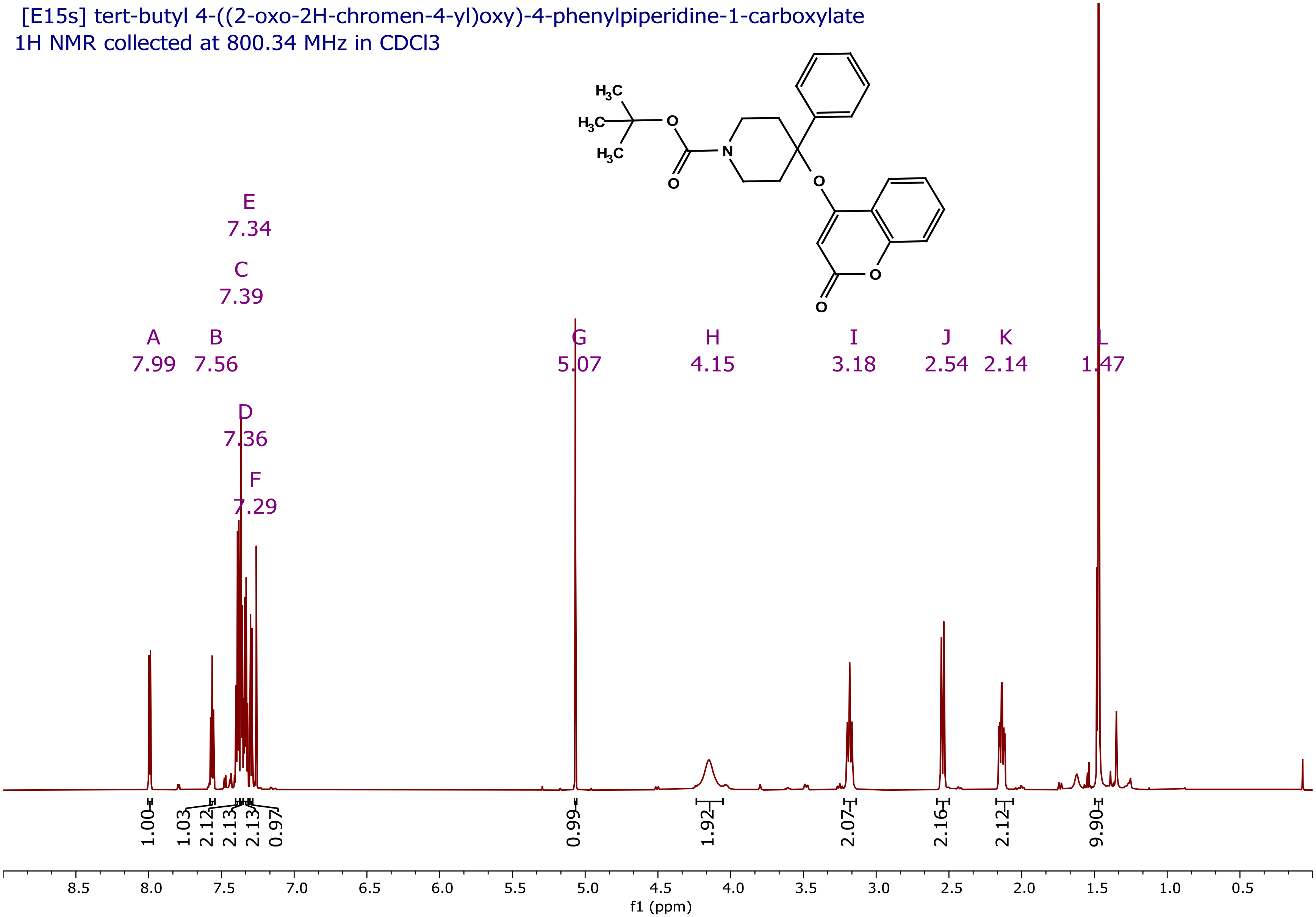

[E15s] tert-butyl 4-((2-oxo-2H-chromen-4-yl)oxy)-4-phenylpiperidine-1-carboxylate
13C NMR collected at 201.27 MHz in CDCl3

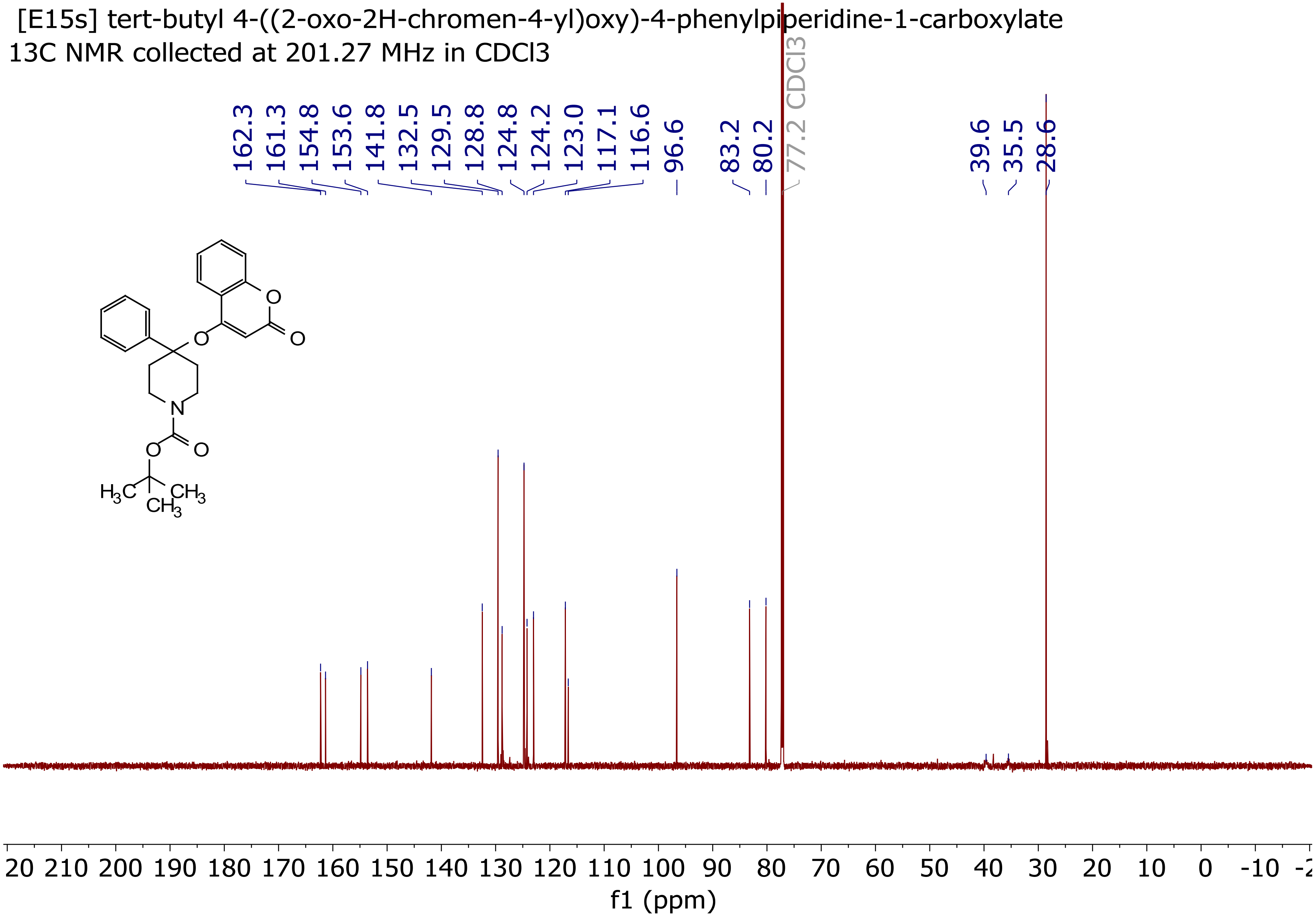

[E12s] tert-butyl 4-phenoxy-4-phenylcyclohexane-1-carboxylate
1H NMR collected at 800.34 MHz in CDCl3

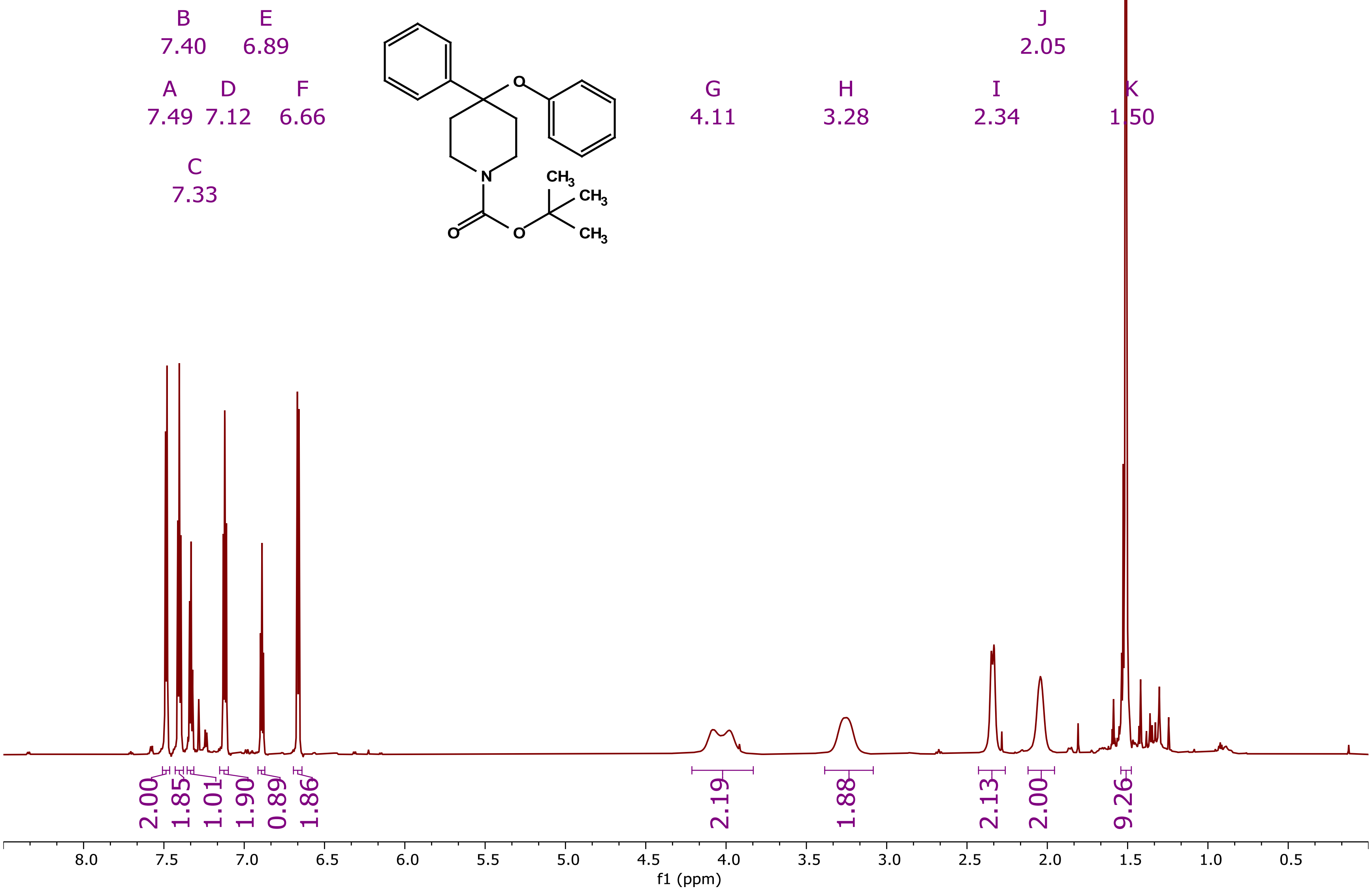

[E12s] tert-butyl 4-phenoxy-4-phenylcyclohexane-1-carboxylate
13C NMR collected at 201.27 MHz in CDCl3

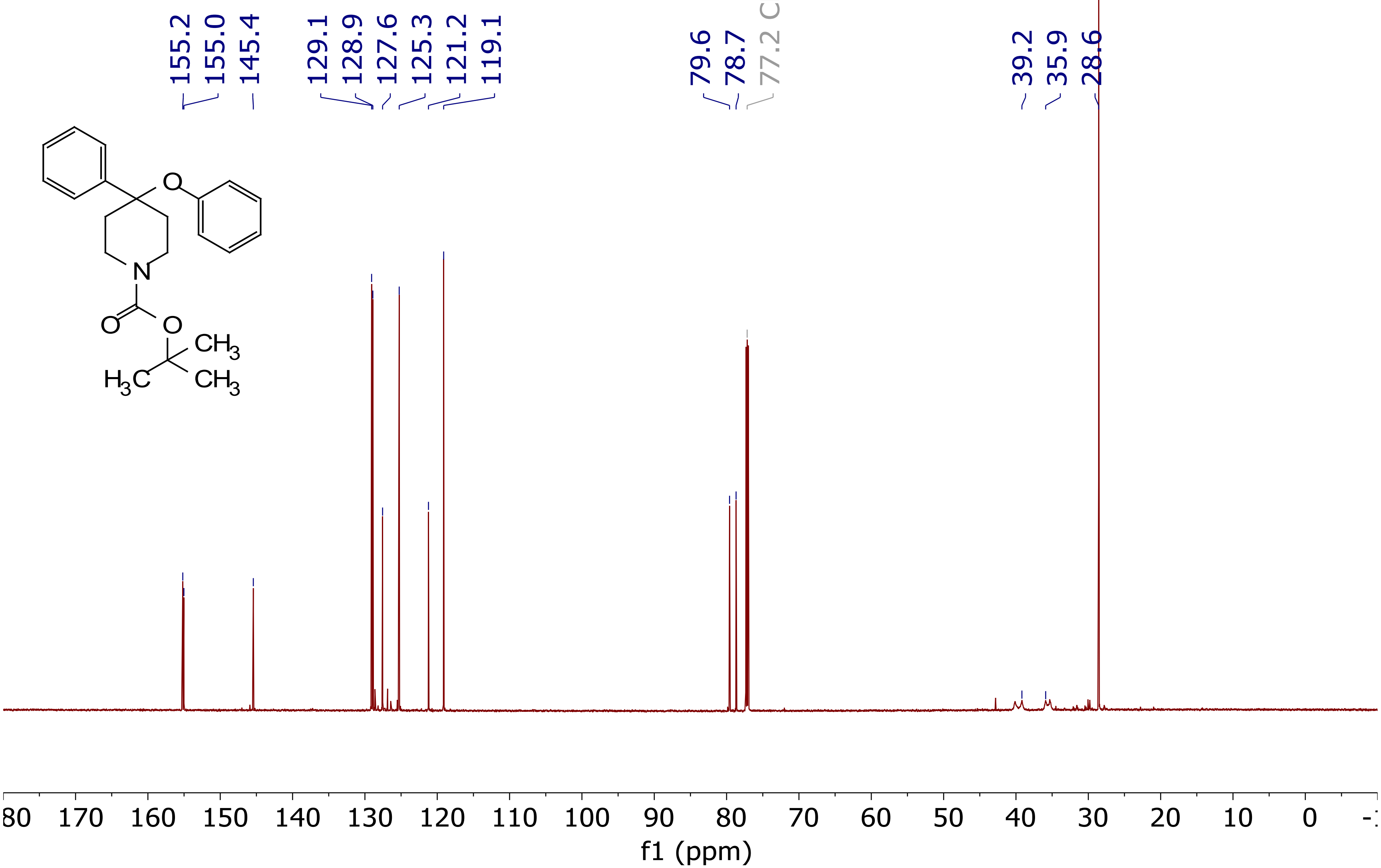

[E13s] tert-butyl 4-(3-hydroxyphenoxy)-4-phenylpiperidine-1-carboxylate
1H NMR collected at 800.34 MHz in CD3CN

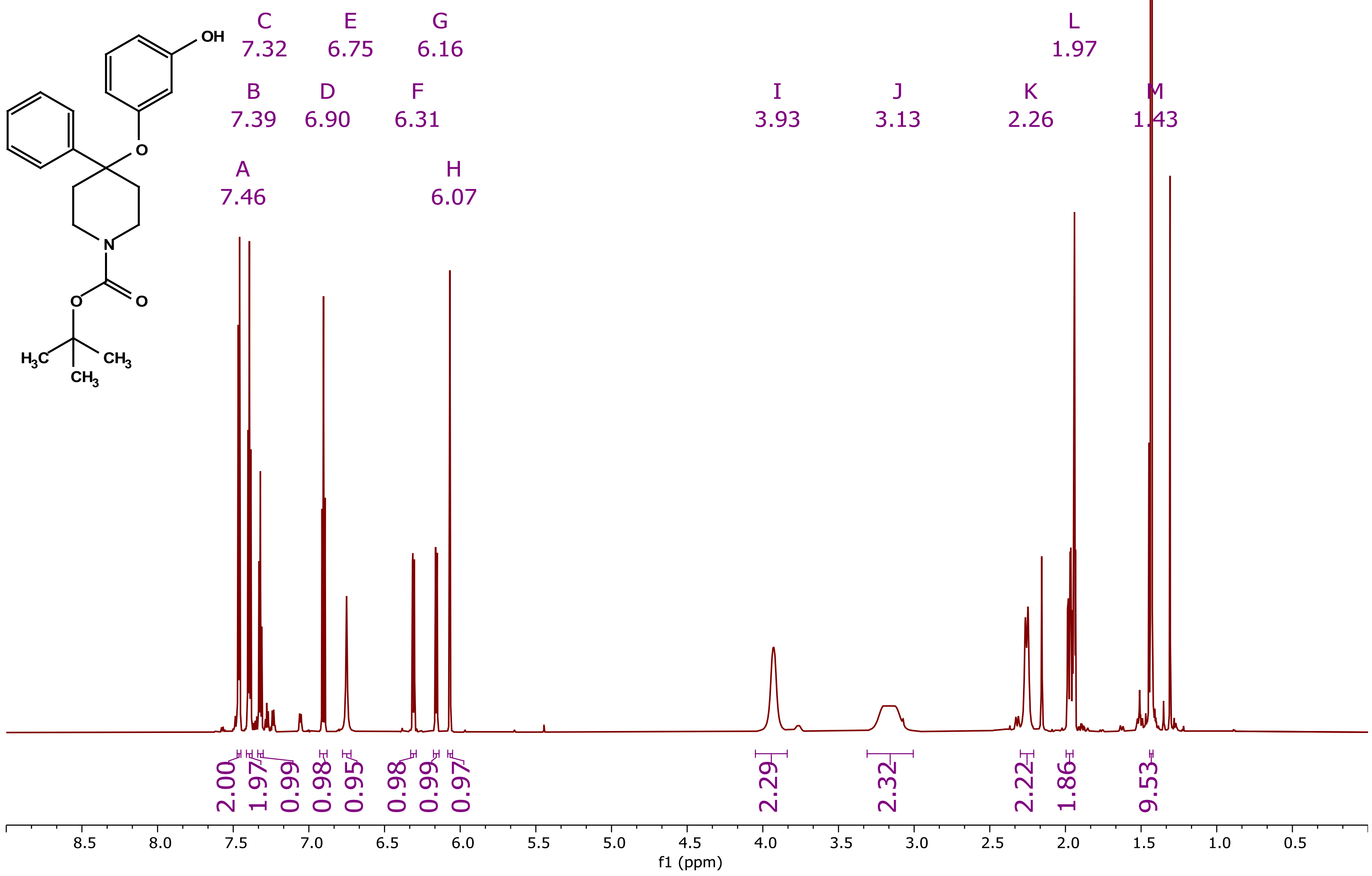

[E13s] tert-butyl 4-(3-hydroxyphenoxy)-4-phenylpiperidine-1-carboxylate
13C NMR collected at 201.27 MHz in CD3CN

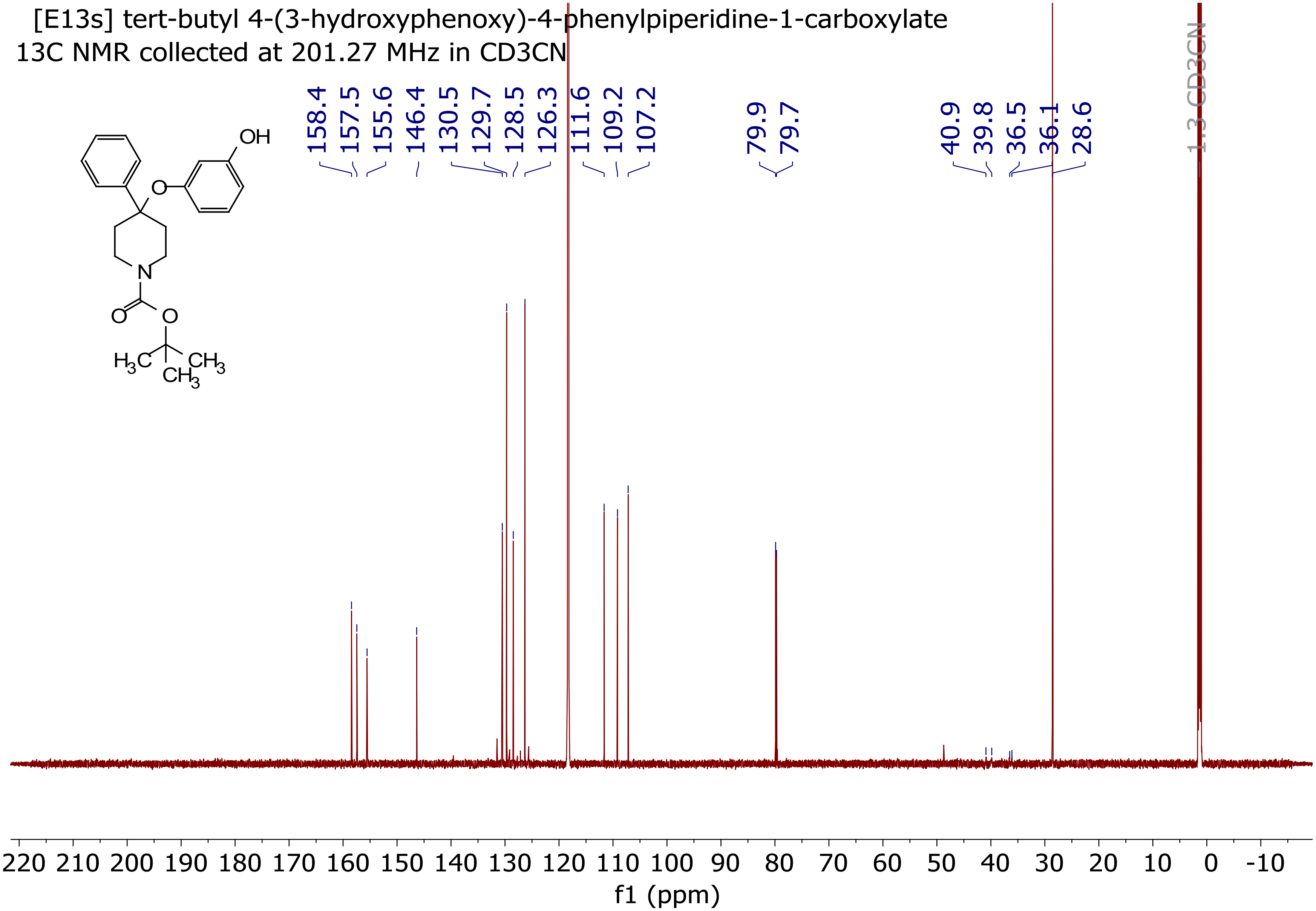

[2i/2i′] 2-methyl-5-(4-methyl-1-phenylcyclohexyl)furan
1H NMR collected at 800.34 MHz in CDCl3

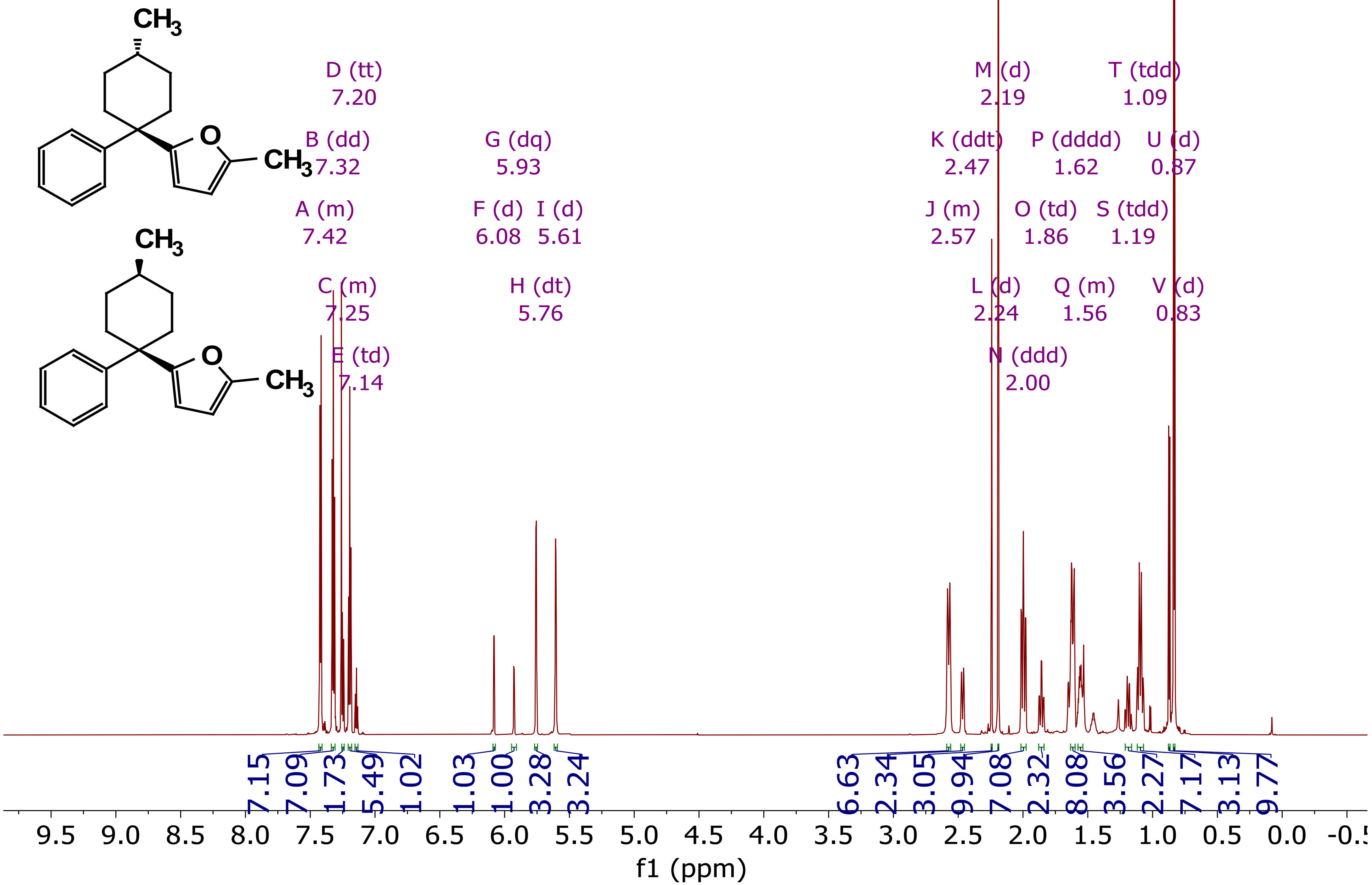

[2i/2i′] 2-methyl-5-(4-methyl-1-phenylcyclohexyl)furan

13C NMR collected at 201.27 MHz in CDCl3

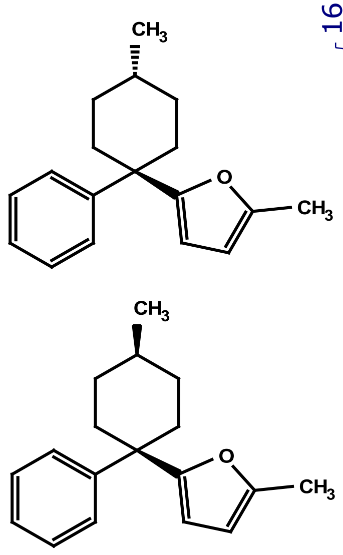

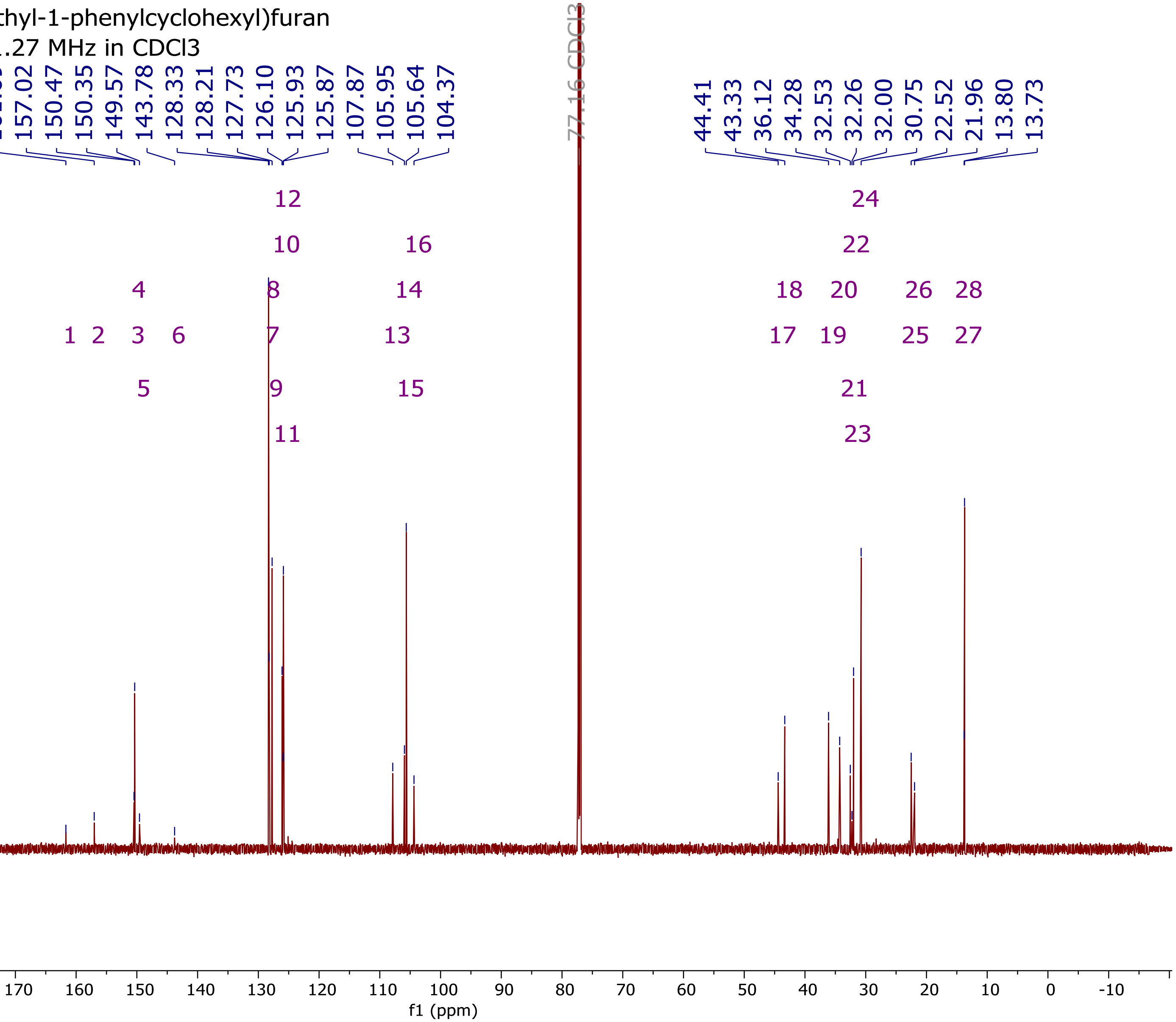

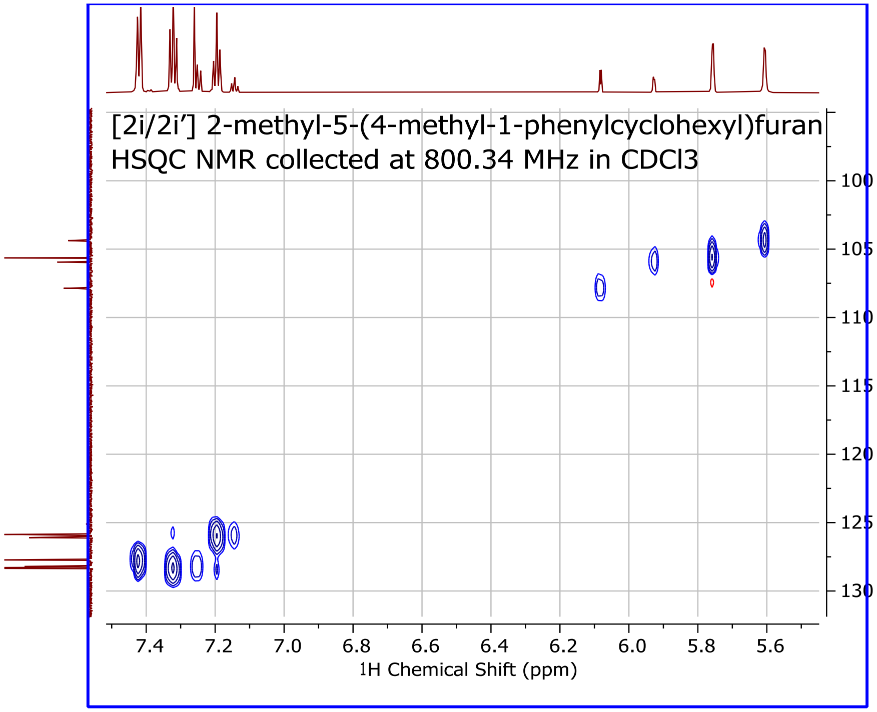
[2i/2i’] 2-methyl-5-(4-methyl-1-phenylcyclohexyl)furan
HSQC NMR collected at 800.34 MHz in CDCl3
1H Chemical Shift (ppm)
13C Chemical Shift (ppm)

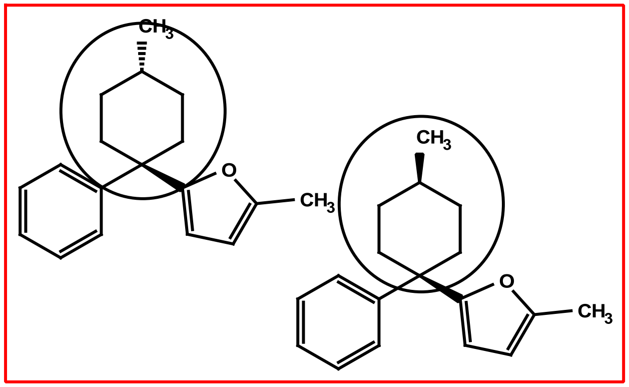
CH3
O
CH3

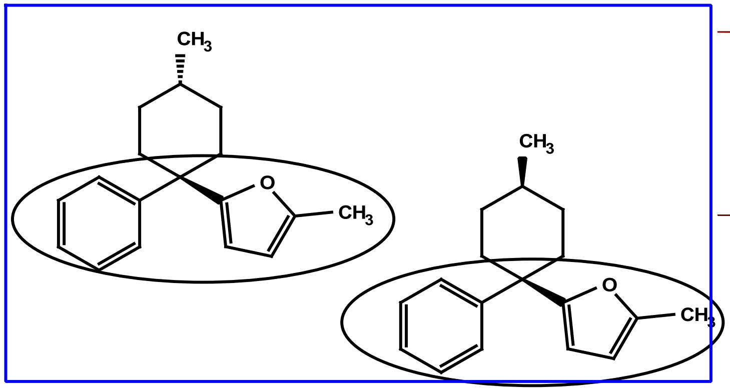
CH3
O
CH3

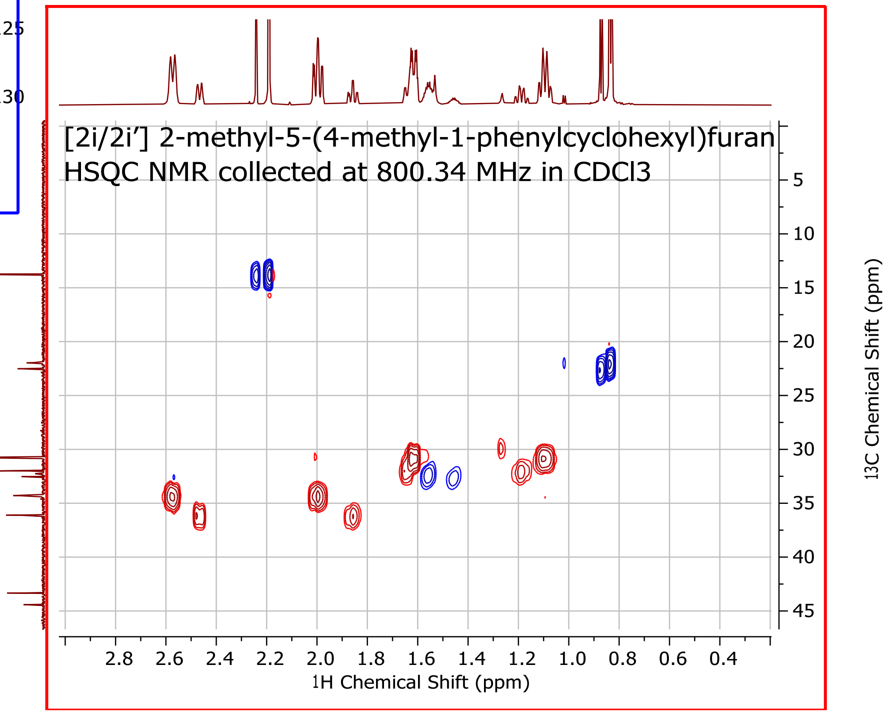
[2i/2i’] 2-methyl-5-(4-methyl-1-phenylcyclohexyl)furan
HSQC NMR collected at 800.34 MHz in CDCl3
1H Chemical Shift (ppm)
13C Chemical Shift (ppm)

[2j/j'] - 2-(4-(tert-butyl)-1-phenylcyclohexyl)-5-methylfuran
1H NMR collected at 800.34 MHz in CDCl3

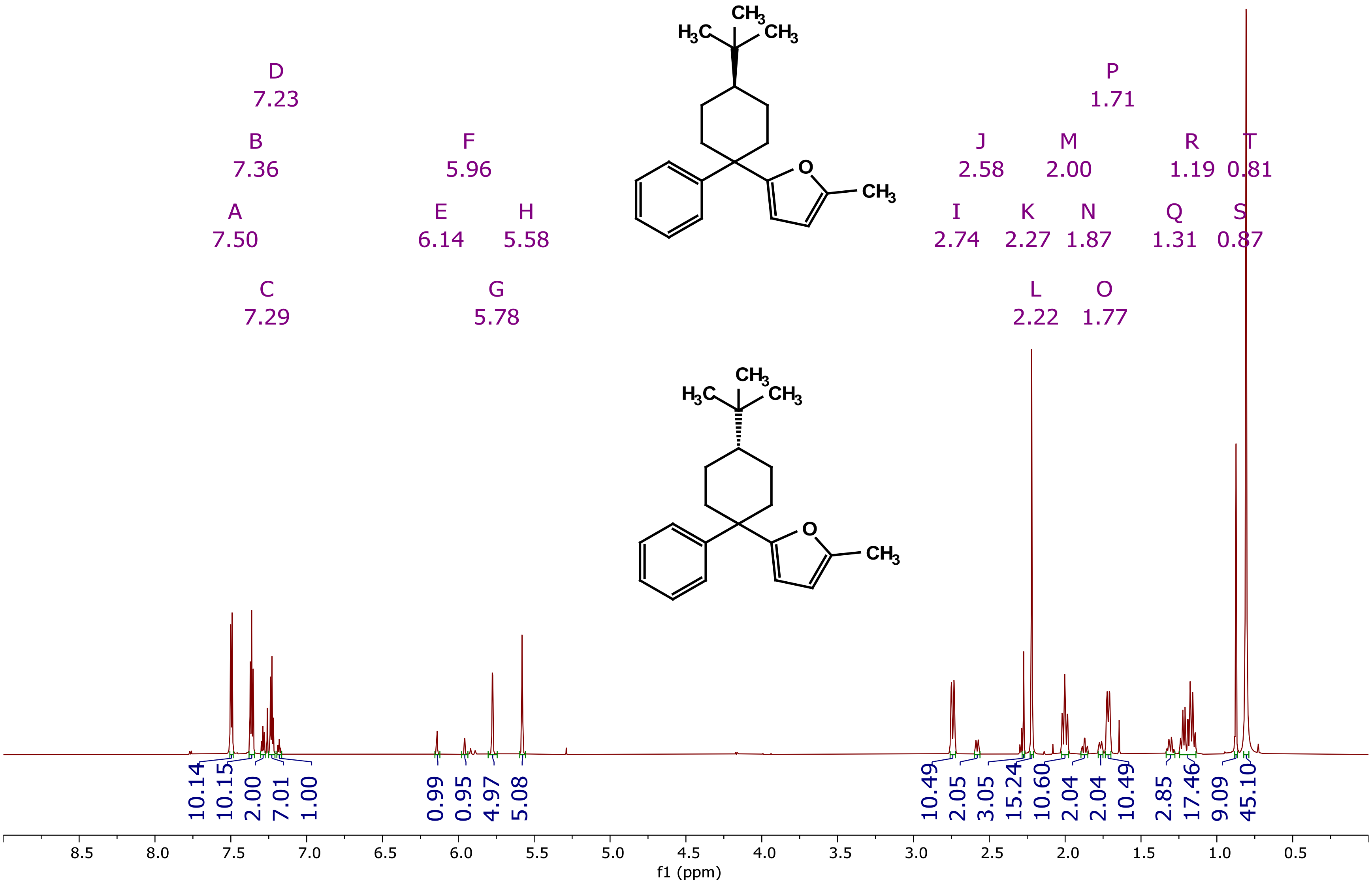

[2j/j'] - 2-(4-(tert-butyl)-1-phenylcyclohexyl)-5-methylfuran
13C NMR collected at 201.27 MHz in CDCl3

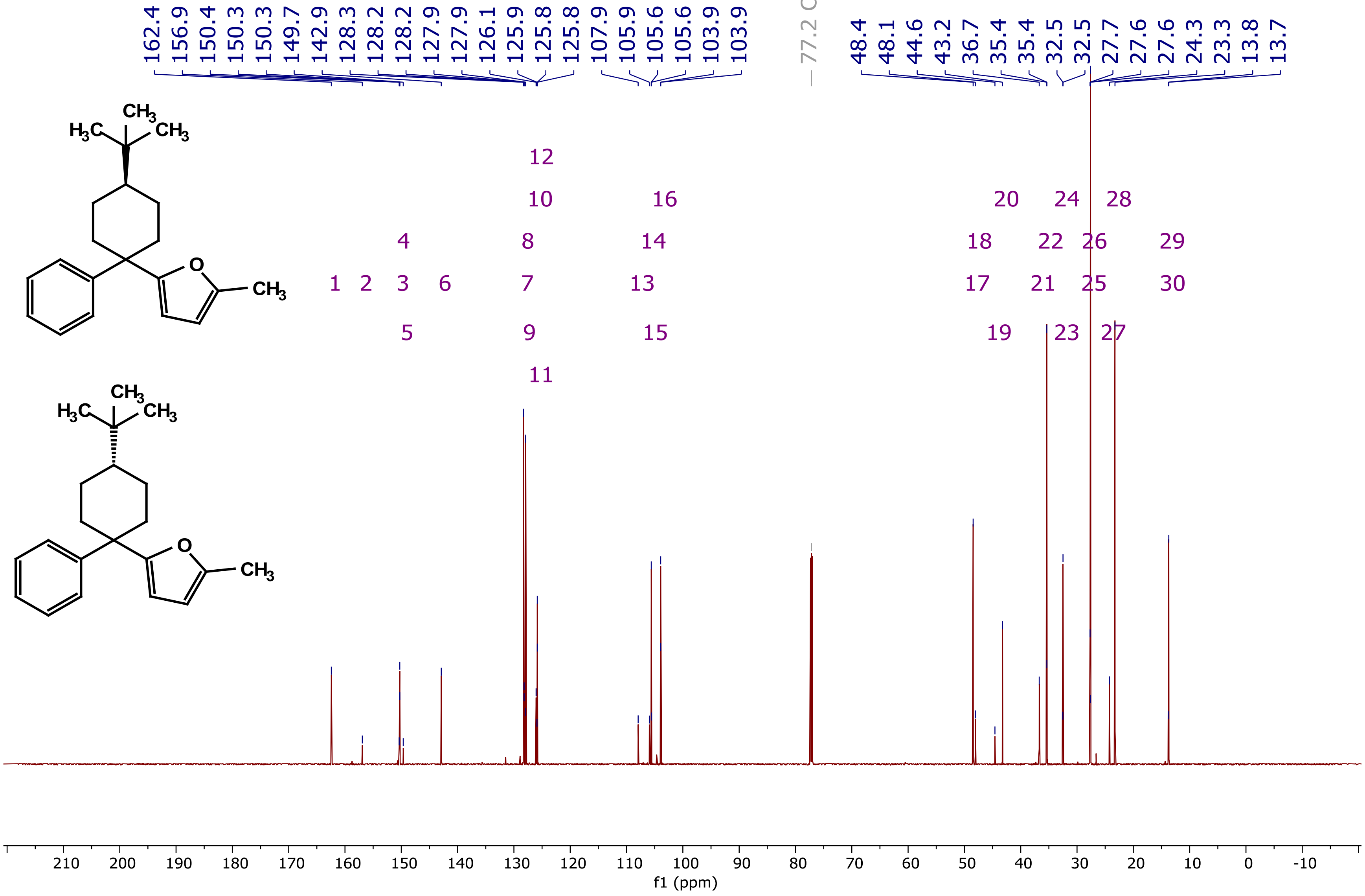

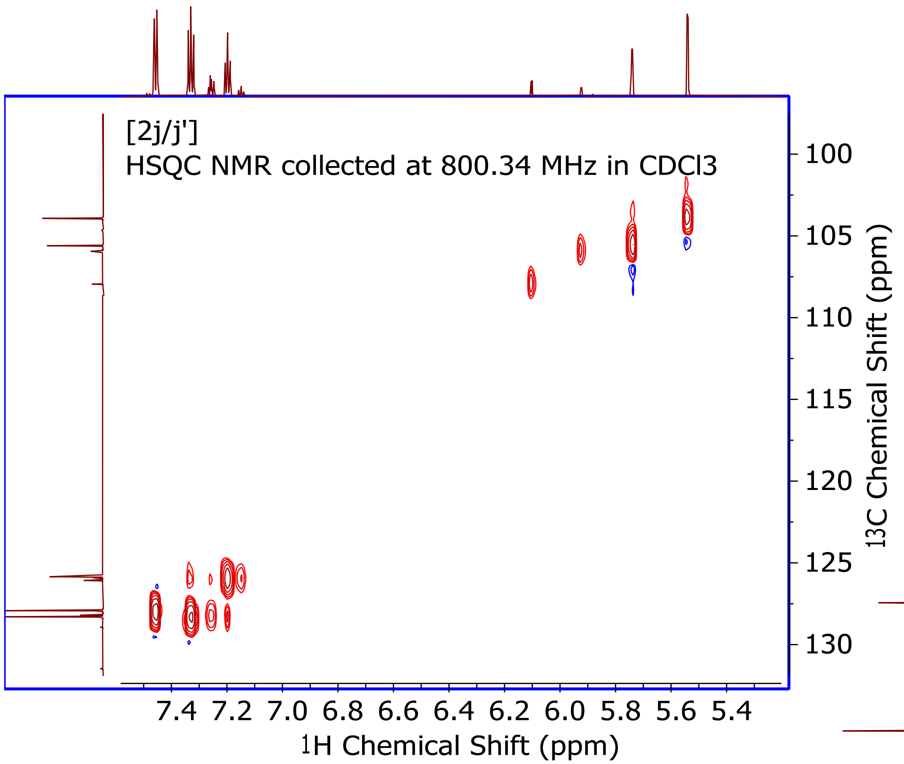
[2j/j']
HSQC NMR collected at 800.34 MHz in CDCl3
100
105
110
115
120
125
130
13C Chemical Shift (ppm)
7.4 7.2 7.0 6.8 6.6 6.4 6.2 6.0 5.8 5.6 5.4
1H Chemical Shift (ppm)

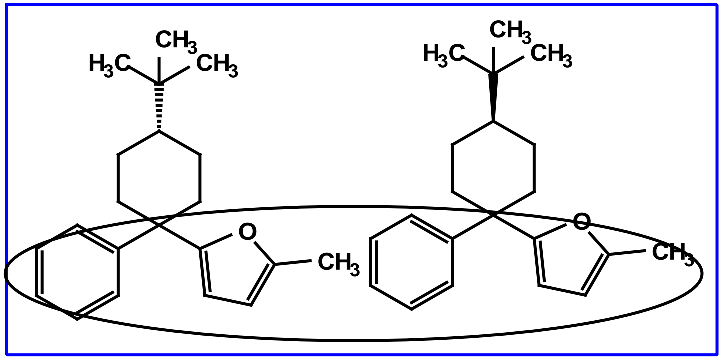

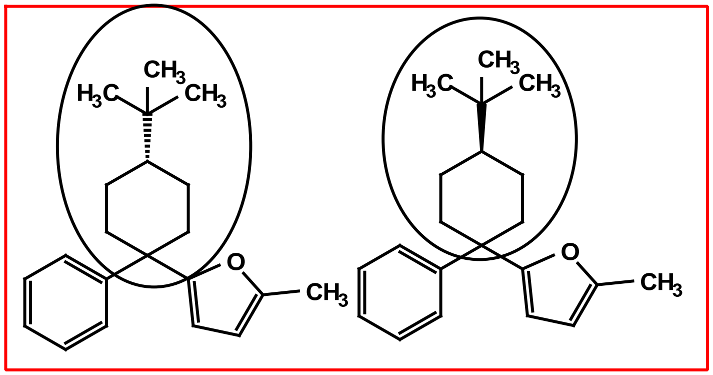

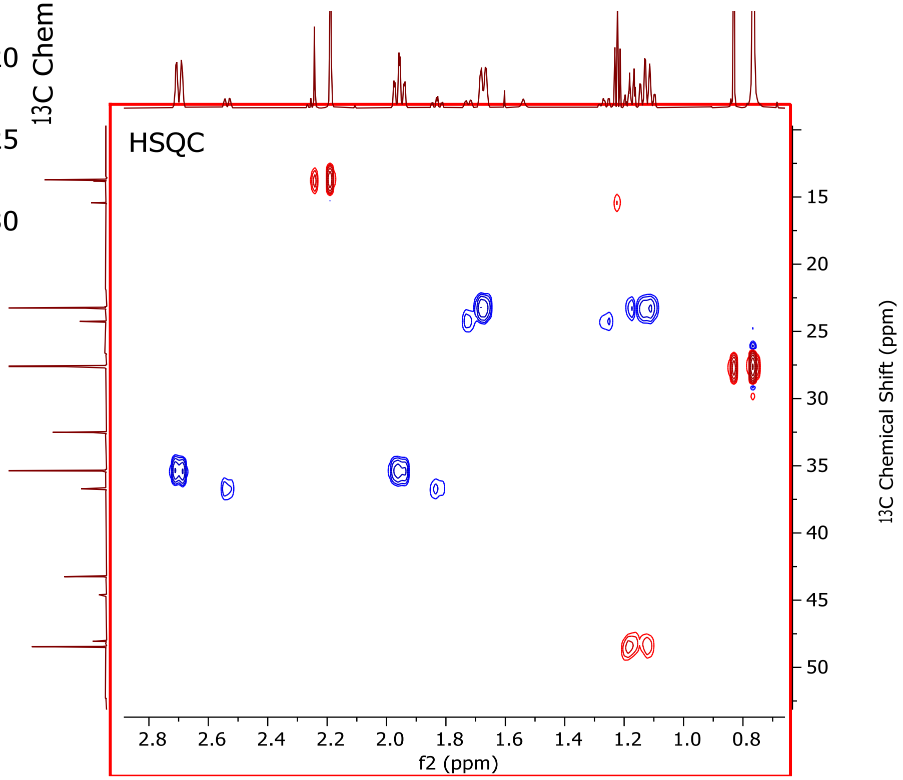
HSQC
15
20
25
30
35
40
45
50
13C Chemical Shift (ppm)
2.8 2.6 2.4 2.2 2.0 1.8 1.6 1.4 1.2 1.0 0.8
f2 (ppm)

[2k] 2-((1s,3R,5S)-3,5-dimethyl-1-phenylcyclohexyl)-5-methylfuran
1H NMR collected at 800.34 MHz in CDCl3

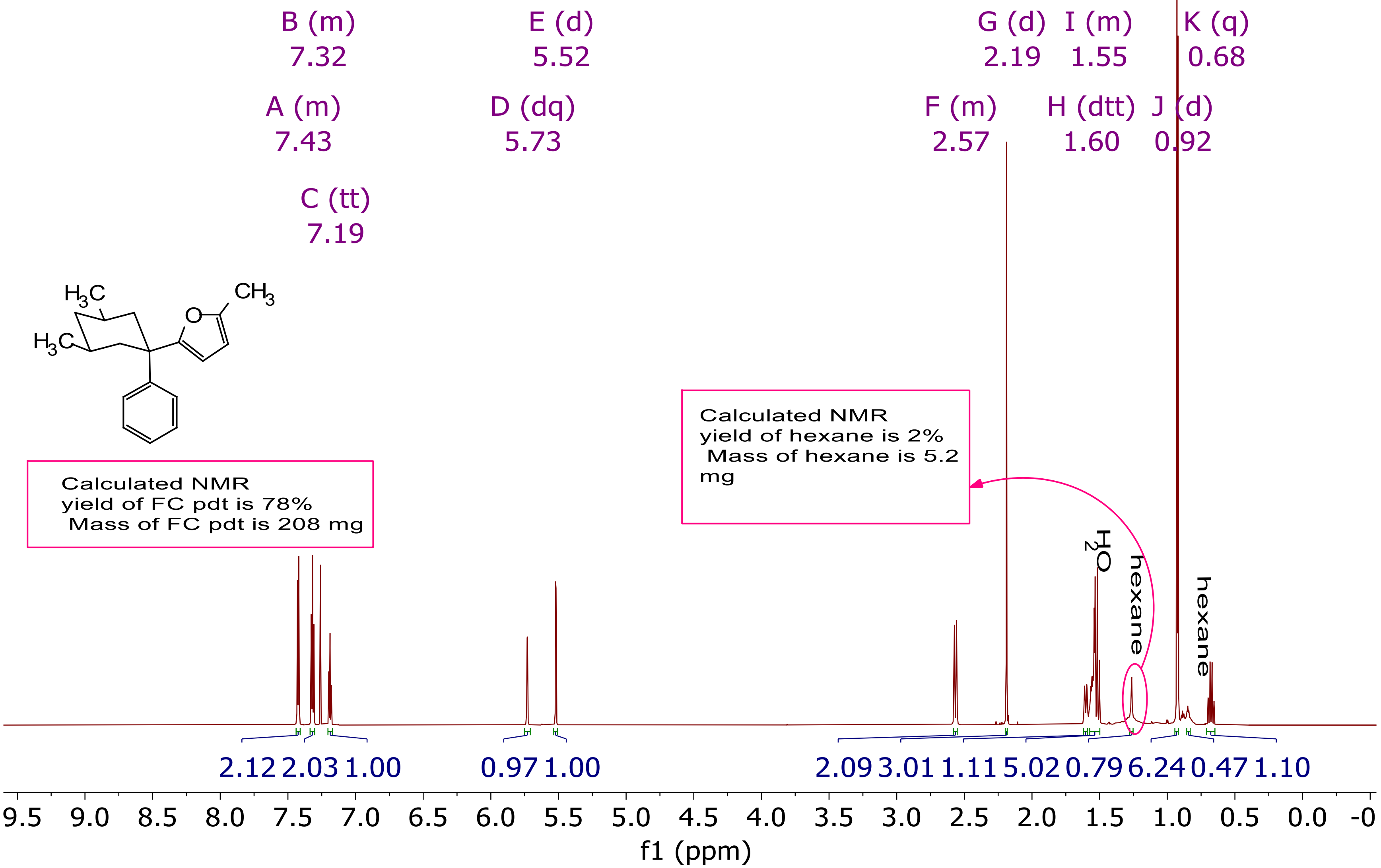

[2k] 2-((1s,3R,5S)-3,5-dimethyl-1-phenylcyclohexyl)-5-methylfuran
13C NMR collected at 201.27 MHz in CDCl3

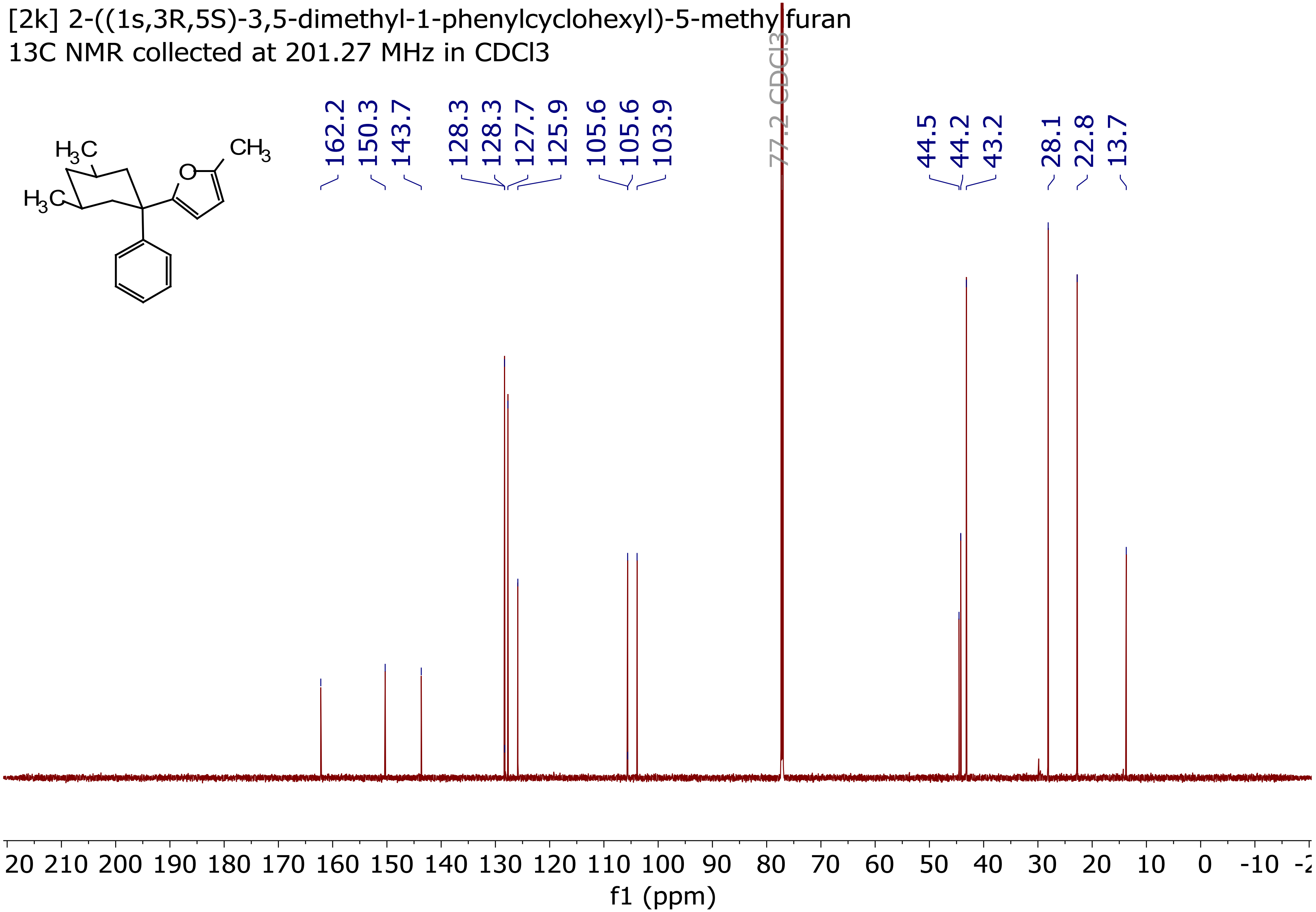

HSQC for 2k

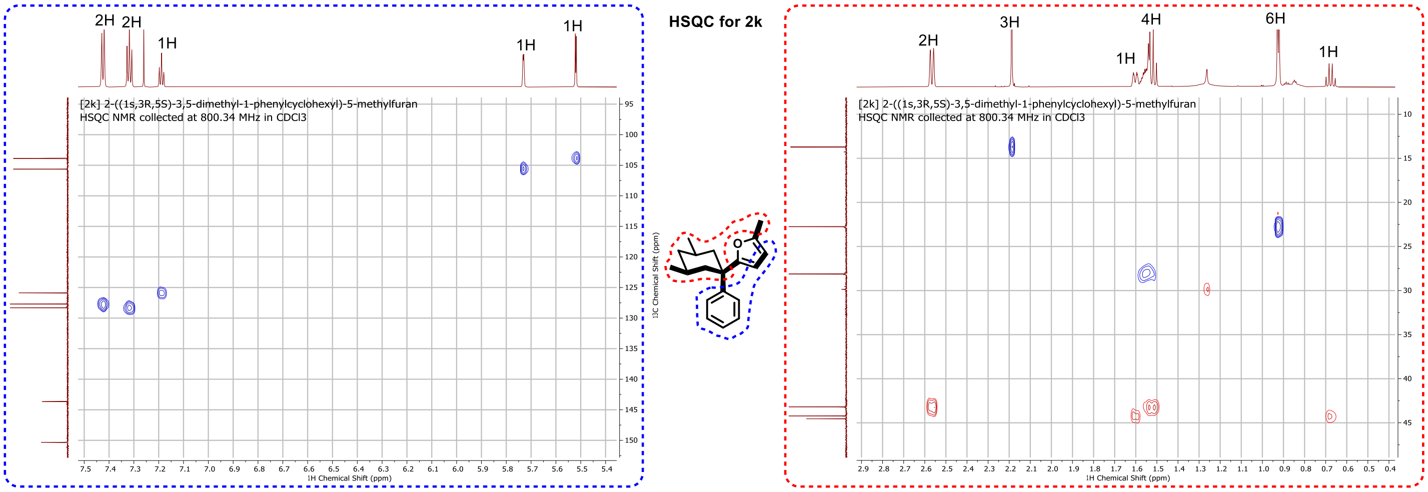

NOE Experiments for 2k

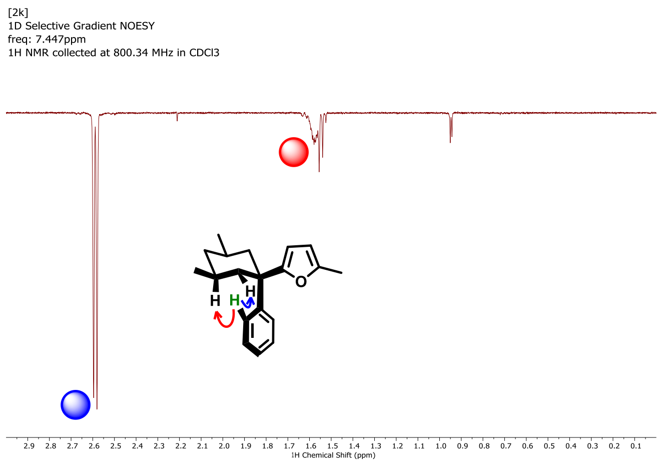

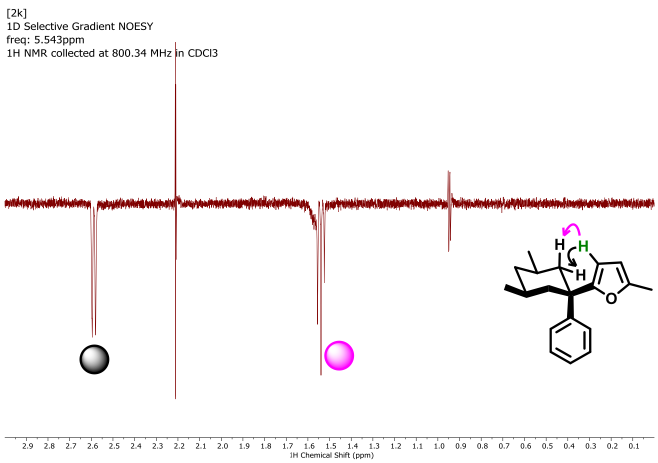

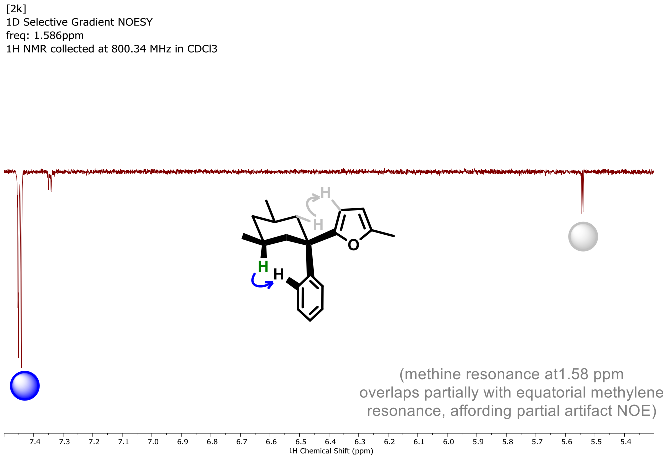

[2l] 4-(4-methoxyphenyl)-4-(5-methylfuran-2-yl)cyclohexan-1-one
1H NMR at 800.34 MHz in CDCl3

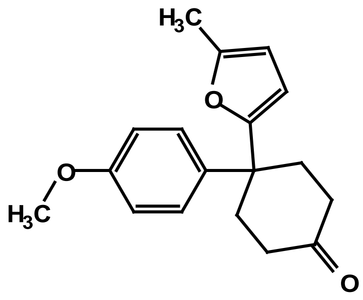

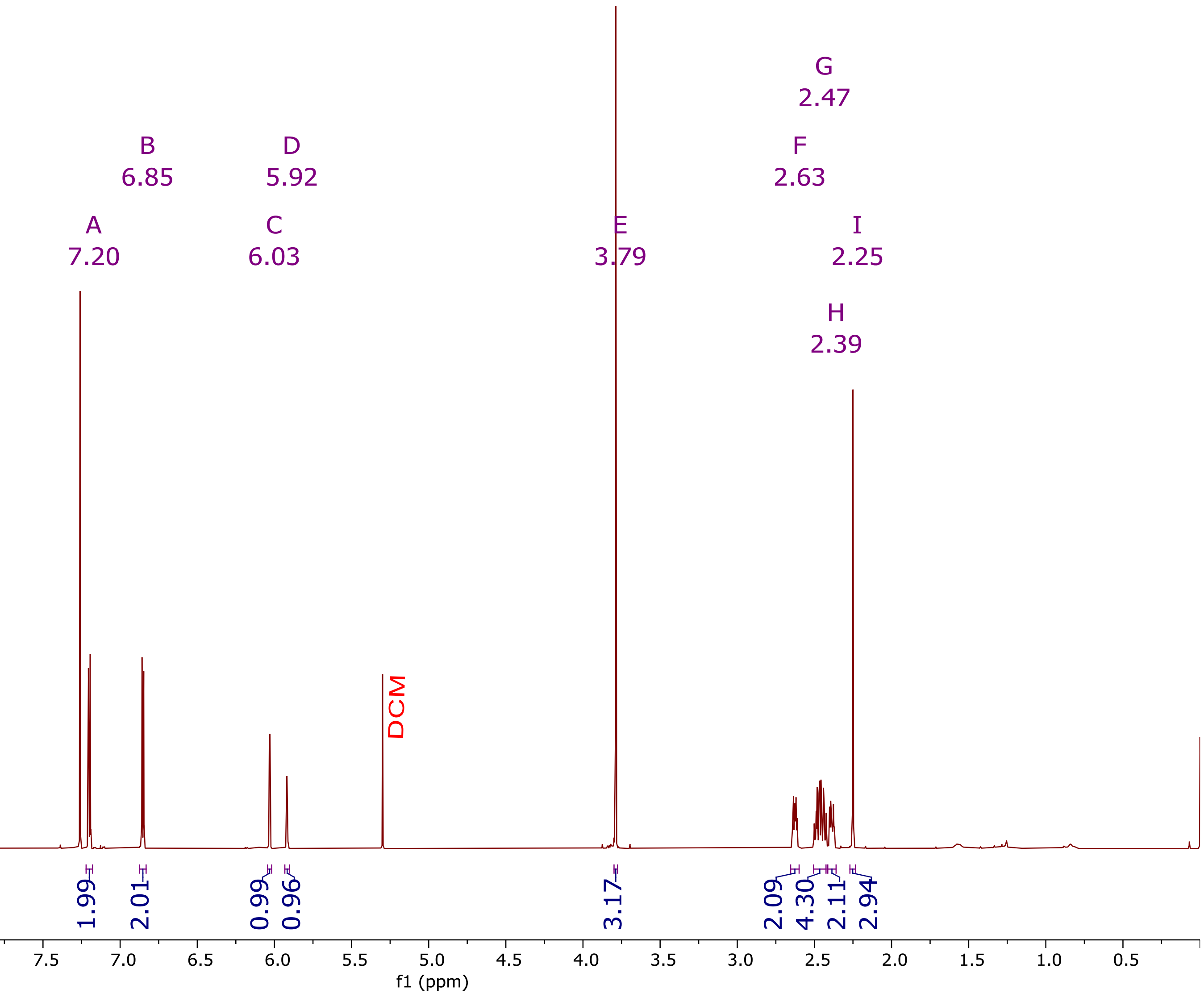

[2l] 4-(4-methoxyphenyl)-4-(5-methylfuran-2-yl)cyclohexan-1-one
13C NMR collected at 201.27 MHz in CDCl3

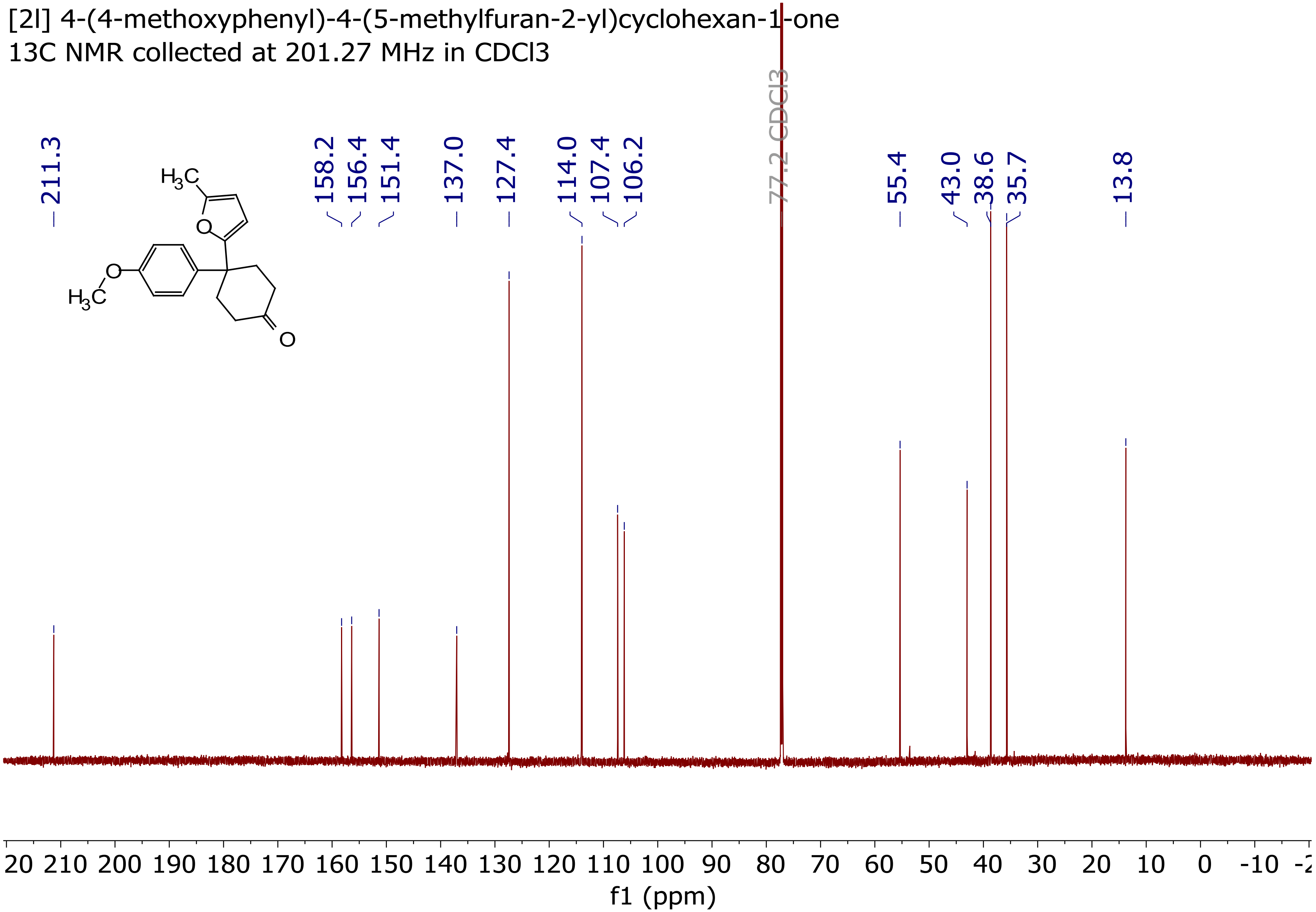

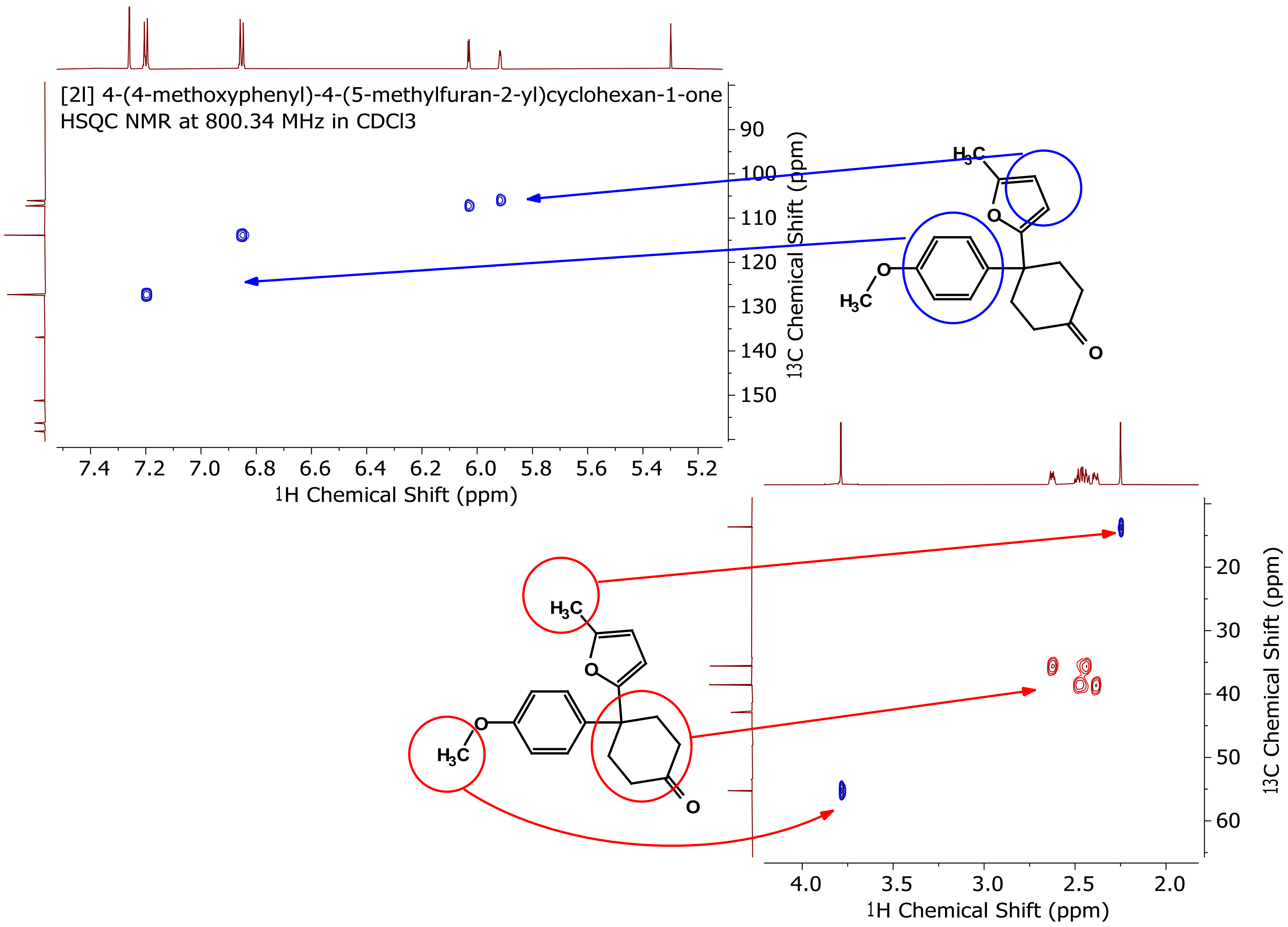

[2l] 4-(4-methoxyphenyl)-4-(5-methylfuran-2-yl)cyclohexan-1-one
HSQC NMR at 800.34 MHz in CDCl3
1H Chemical Shift (ppm)
13C Chemical Shift (ppm)
1H Chemical Shift (ppm)
13C Chemical Shift (ppm)

[2m] 3-(4-methoxyphenyl)-3-(5-methylfuran-2-yl)cyclohexan-1-one
1H NMR collected at 800.34 MHz in CDCl3

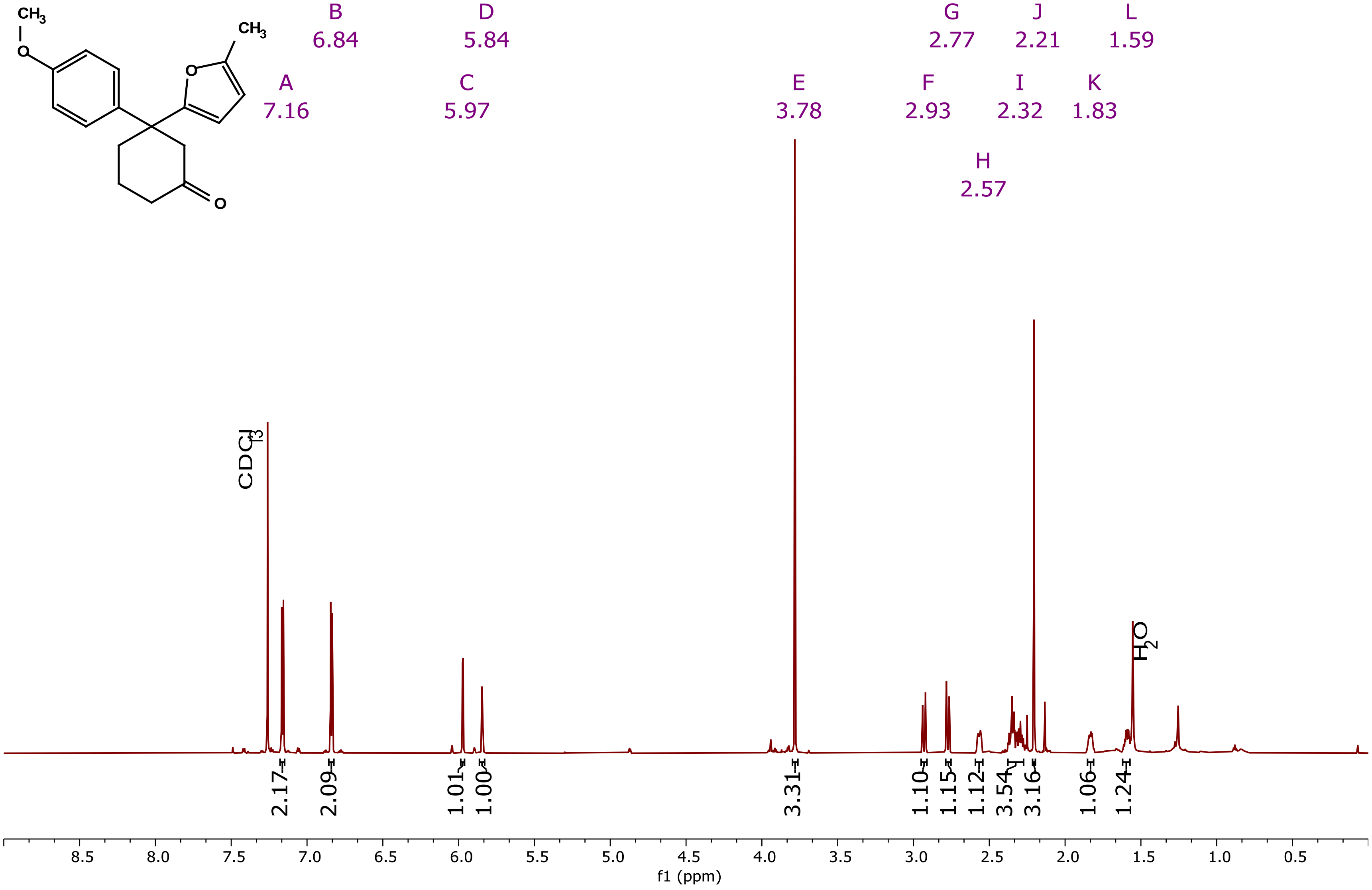

[2m] 3-(4-methoxyphenyl)-3-(5-methylfuran-2-yl)cyclohexan-1-one
13C NMR collected at 201.27 MHz in CDCl3

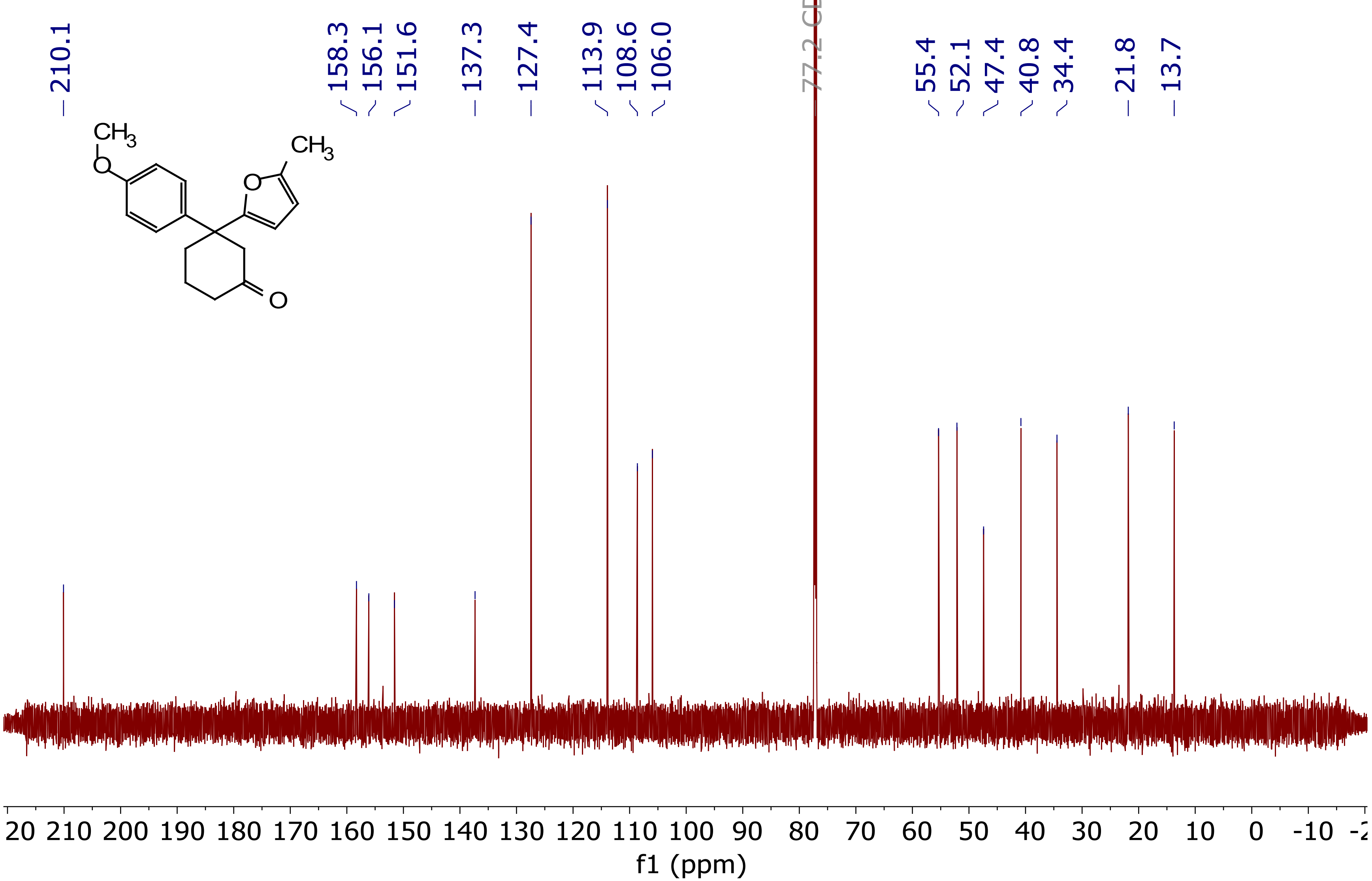

[2n] 4-(5-methylfuran-2-yl)-4-phenyl-1-tosylpiperidine
1H NMR collected at 800.34 MHz in CDCl3

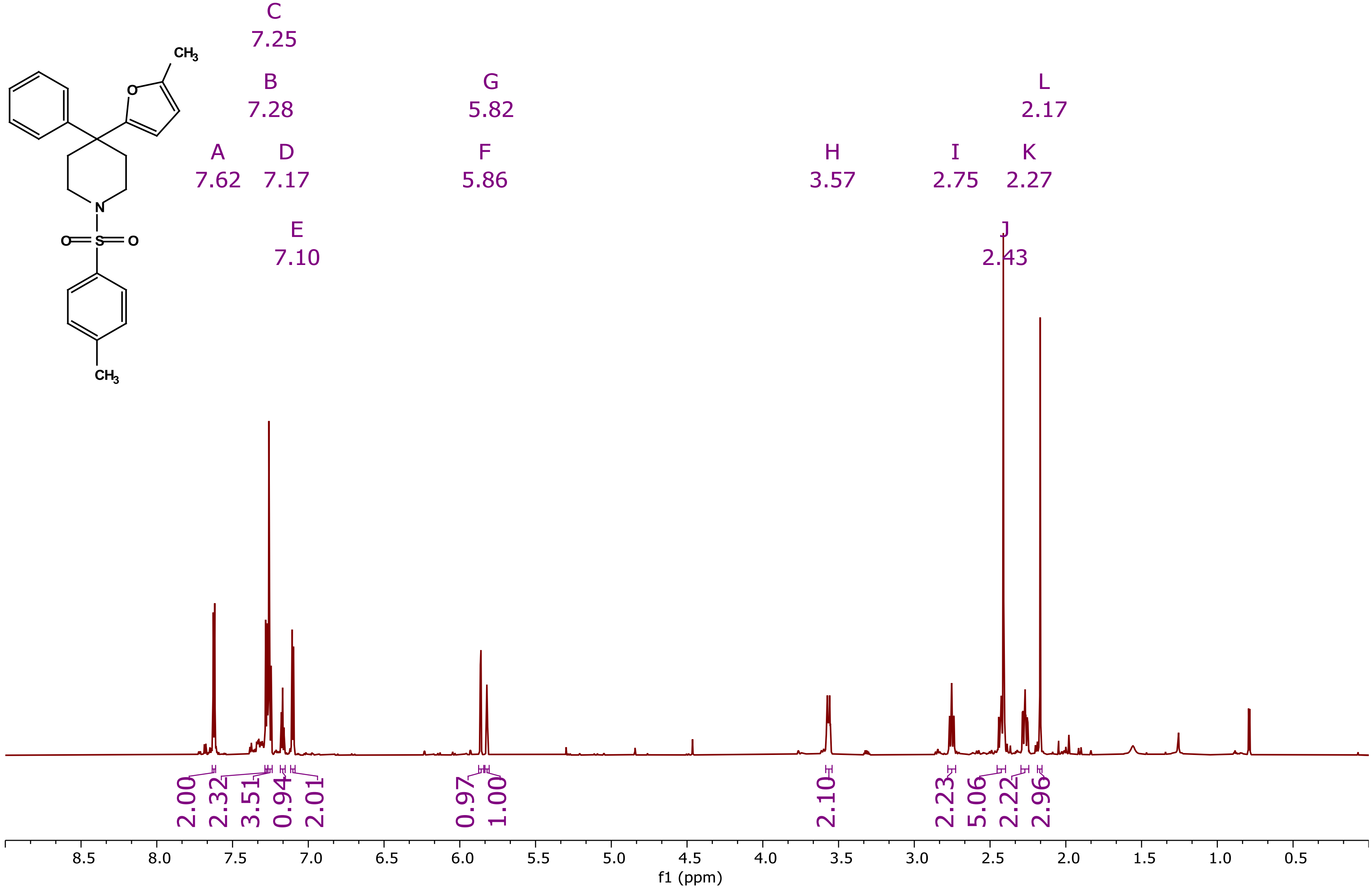

[2n] 4-(5-methylfuran-2-yl)-4-phenyl-1-tosylpiperidine
13C NMR collected at 201.27 MHz in CDCl3

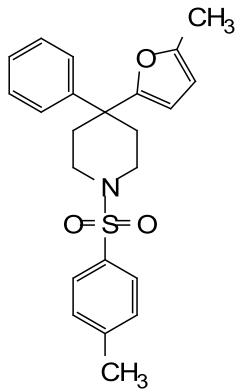

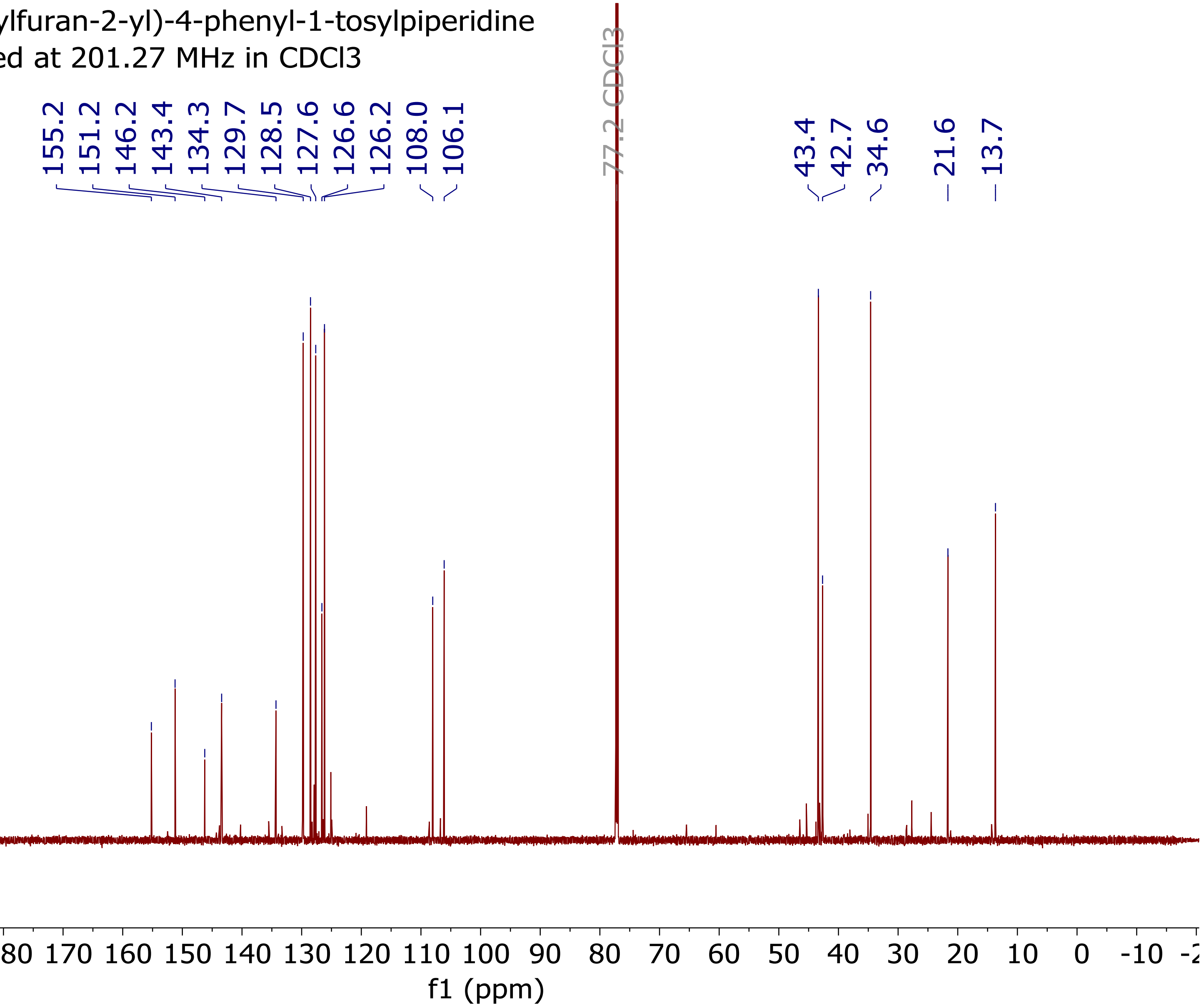

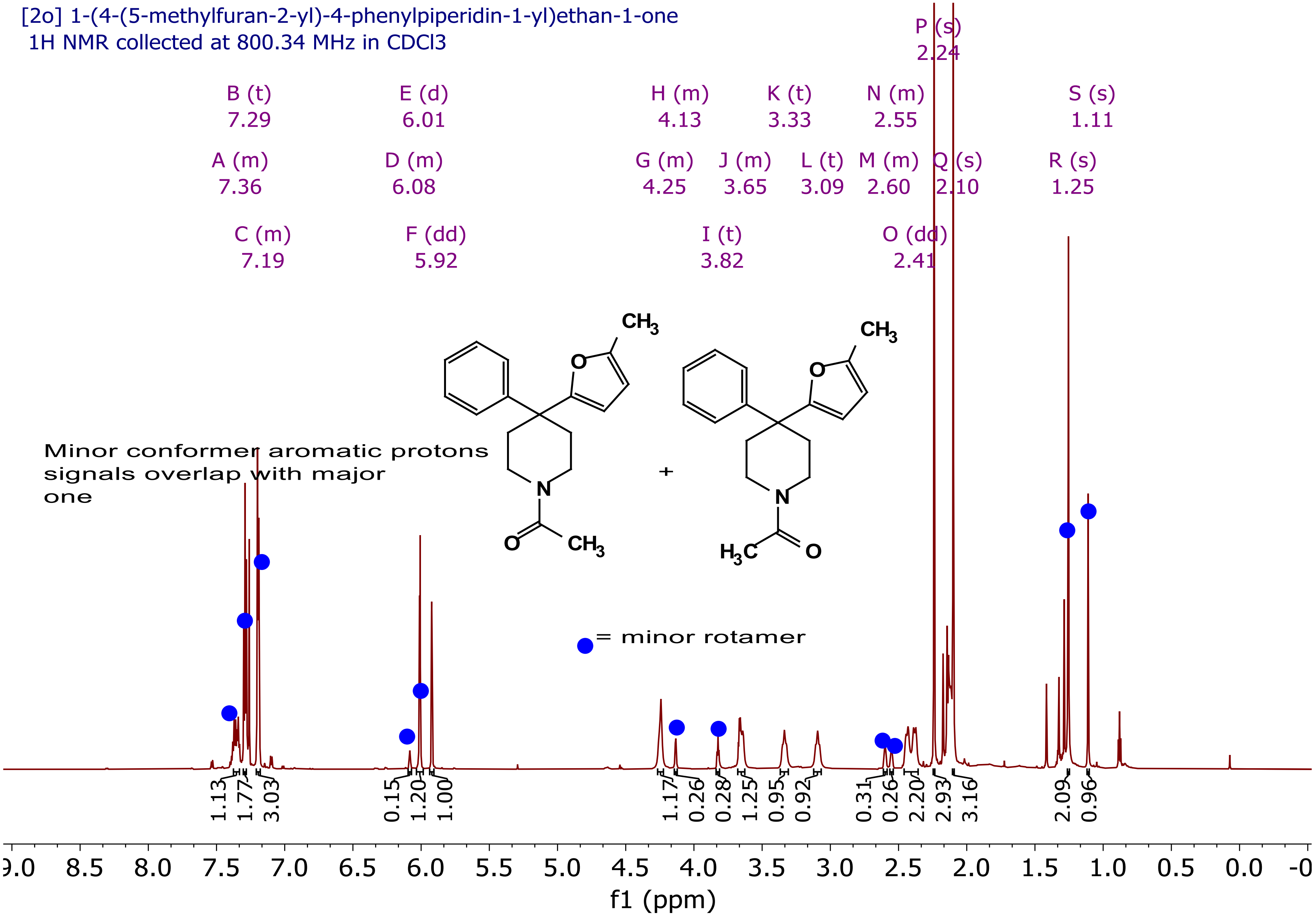
[2o] 1-(4-(5-methylfuran-2-yl)-4-phenylpiperidin-1-yl)ethan-1-one
1H NMR collected at 800.34 MHz in CDCl3
P (s) 2.24
B (t) 7.29
E (d) 6.01
H (m) 4.13
K (t) 3.33
N (m) 2.55
S (s) 1.11
A (m) 7.36
D (m) 6.08
G (m) 4.25
J (m) 3.65
L (t) 3.09
M (m) 2.60
Q (s) 2.10
R (s) 1.25
C (m) 7.19
F (dd) 5.92
I (t) 3.82
O (dd) 2.41
Minor conformer aromatic protons signals overlap with major one
+
= minor rotamer
1.13 1.77 3.03 0.15 1.20 1.00 1.17 0.26 0.28 1.25 0.95 0.92 0.31 0.26 2.20 2.93 3.16 2.09 0.96
f1 (ppm)

[2o] 1-(4-(5-methylfuran-2-yl)-4-phenylpiperidin-1-yl)ethan-1-one
13C NMR collected at 201.27 MHz in CDCl3

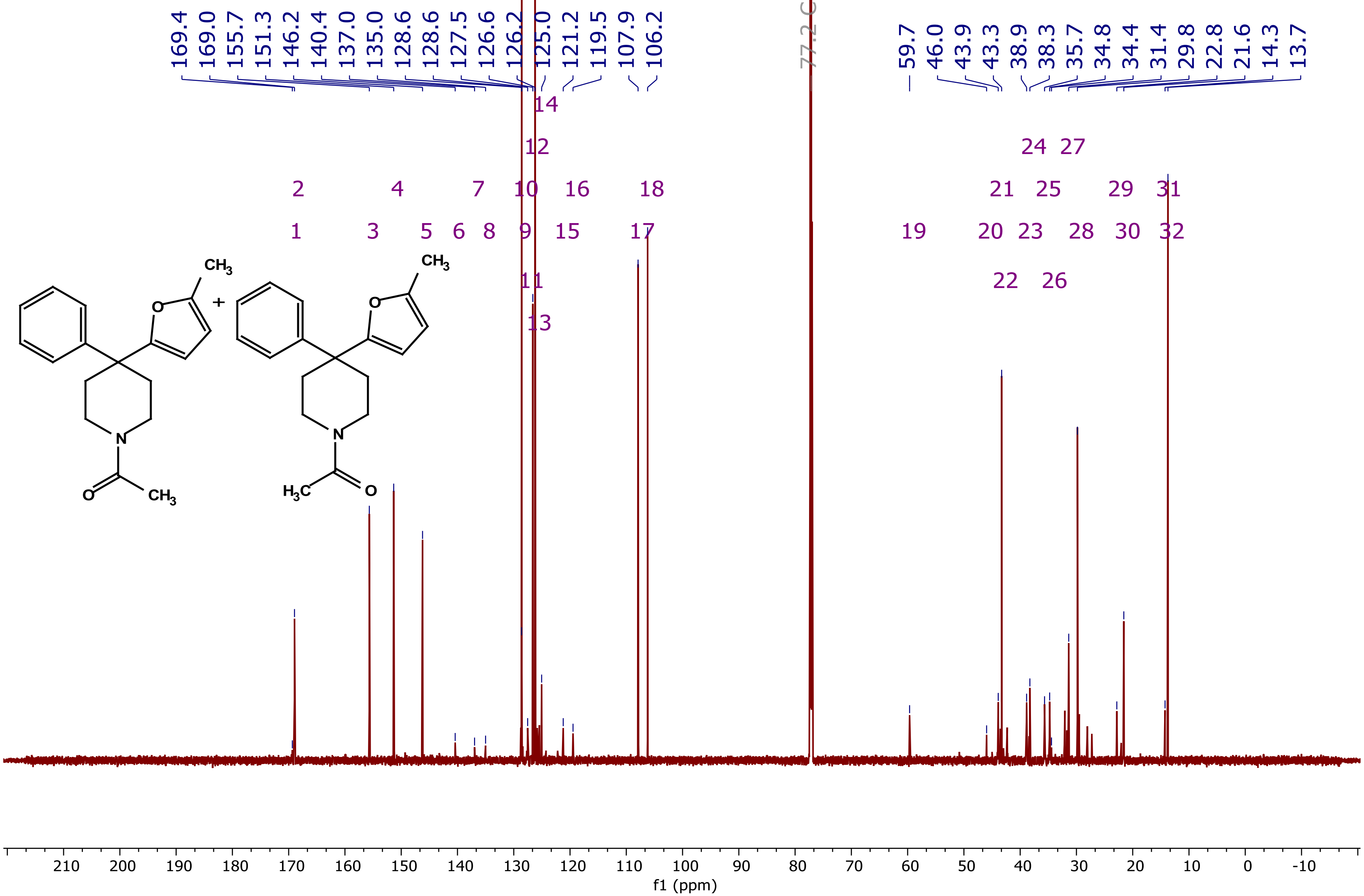

[2s] tert-butyl 4-(5-methylfuran-2-yl)-4-phenylpiperidine-1-carboxylate
1H NMR collected at 800.34 MHz in CDCl3

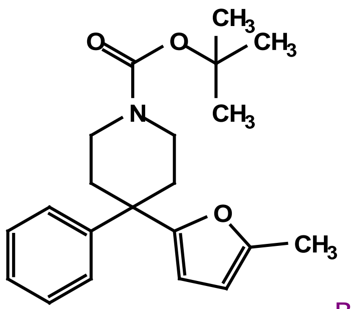

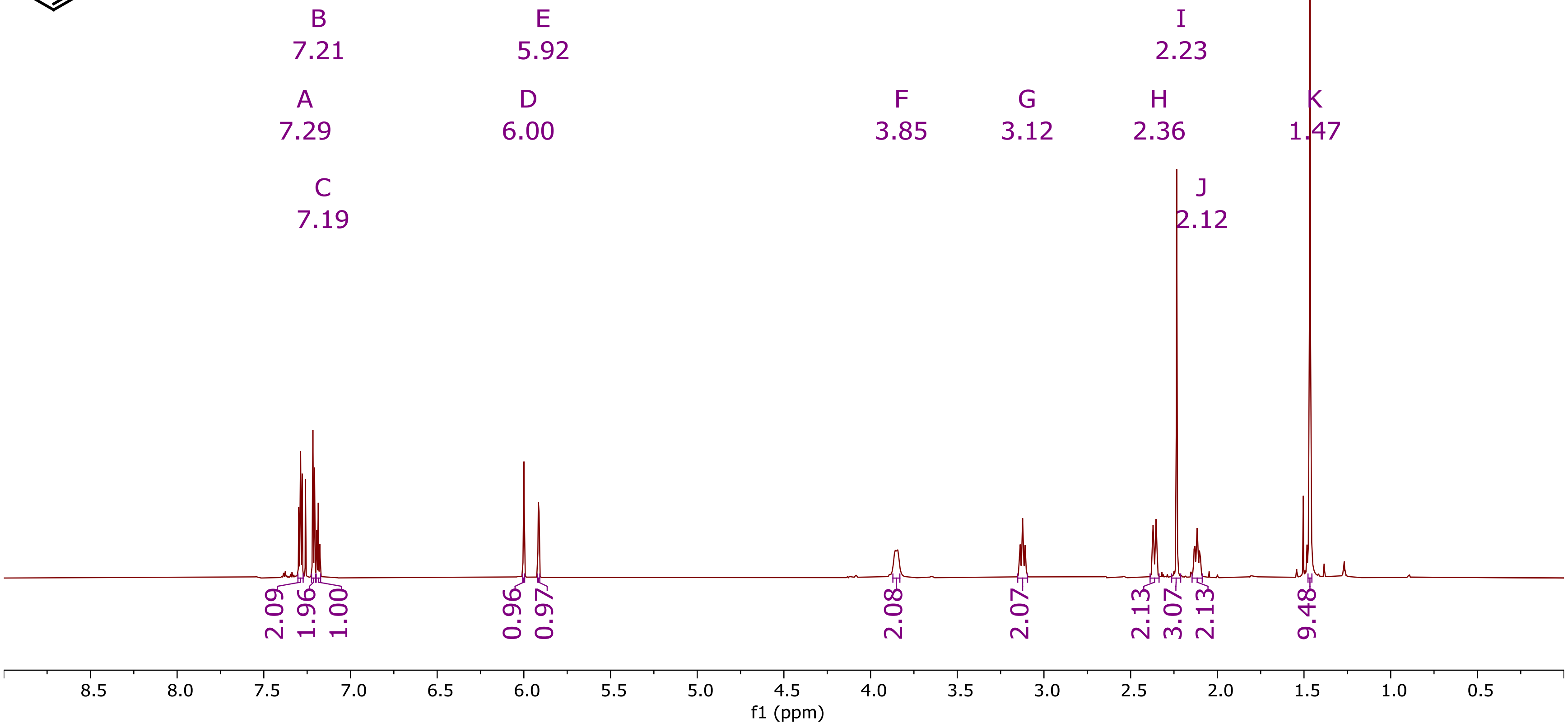

[2s] tert-butyl 4-(5-methylfuran-2-yl)-4-phenylpiperidine-1-carboxylate
13C NMR collected at 201.27 MHz in CDCl3

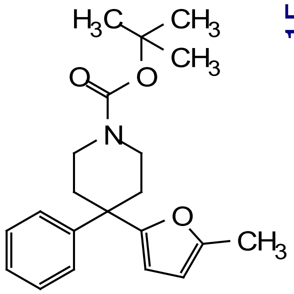

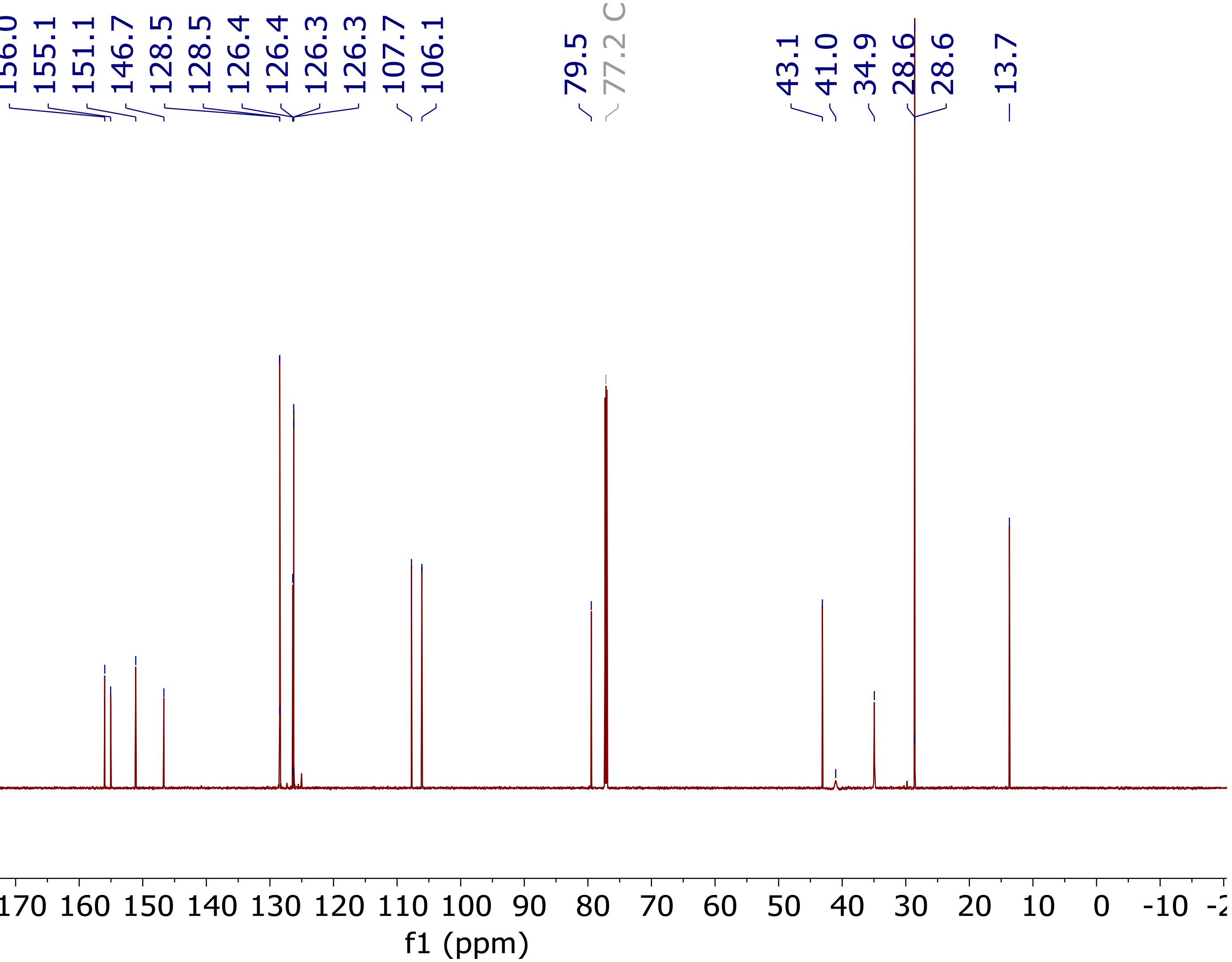

[8s] tert-butyl 4-(2,4-dimethoxyphenyl)-4-phenylpiperidine-1-carboxylate
1H NMR collected at 800.34 MHz in CDCl3

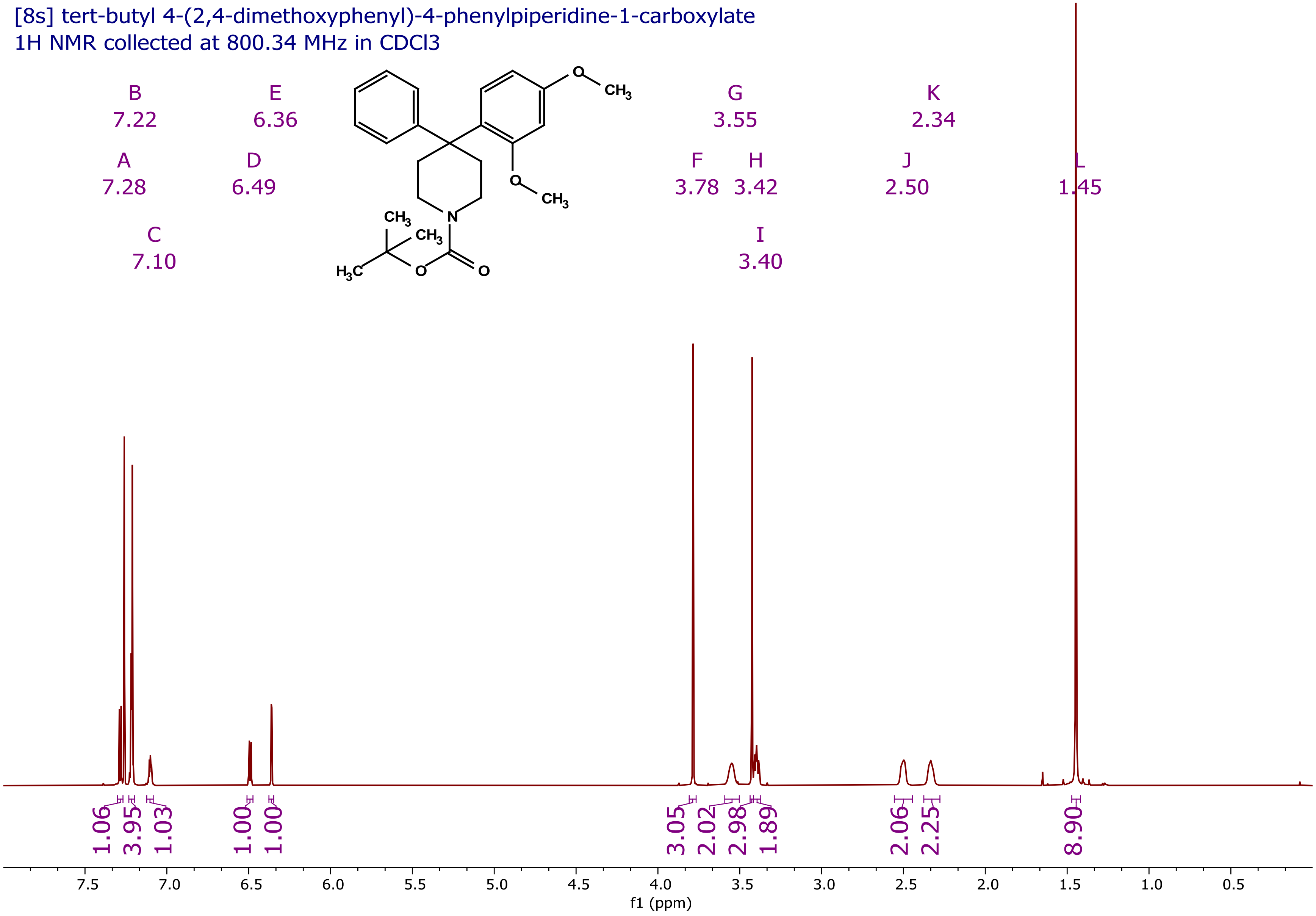

[8s] tert-butyl 4-(2,4-dimethoxyphenyl)-4-phenylpiperidine-1-carboxylate
13C NMR collected at 201.27 MHz in CDCl3

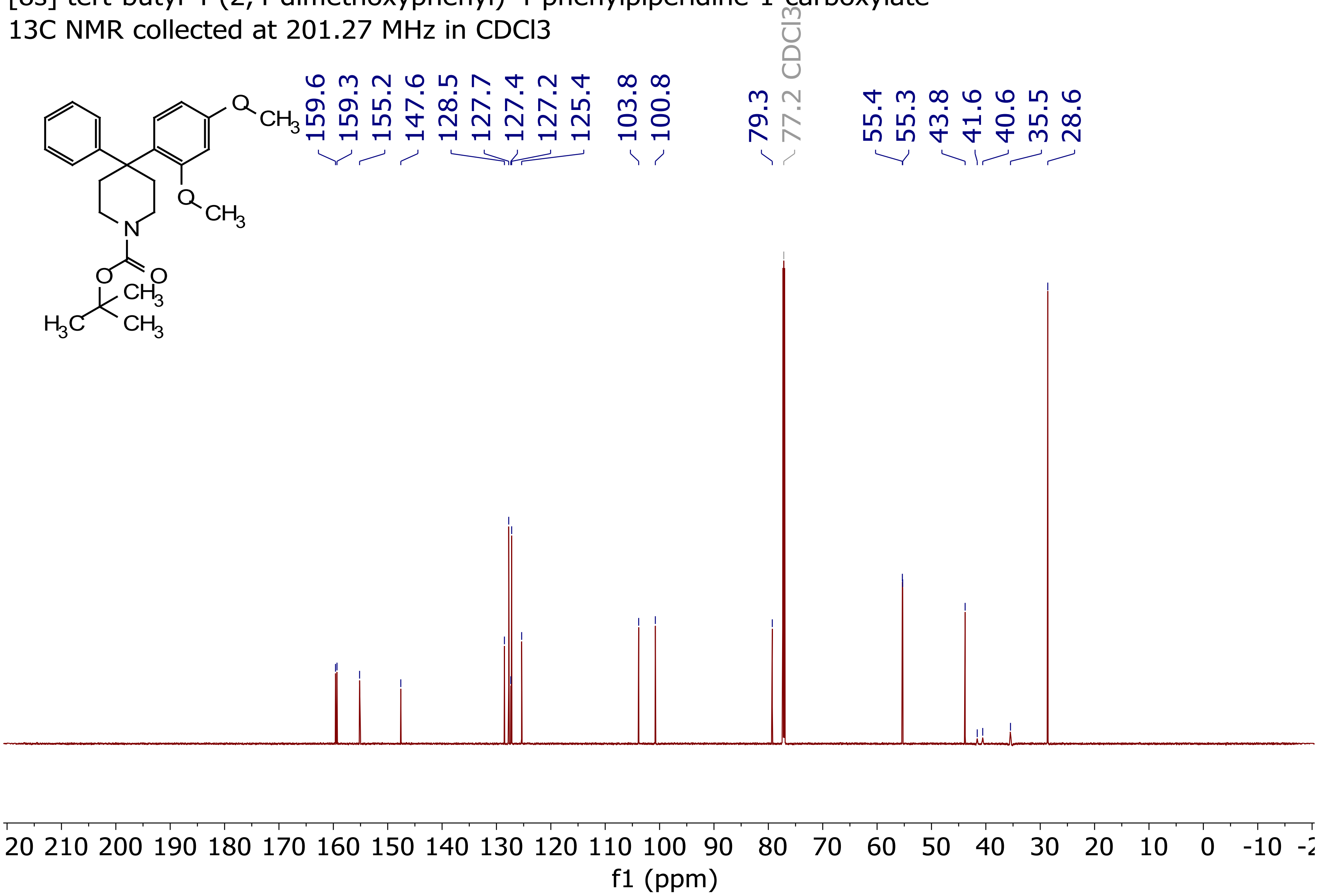

[16s] tert-butyl 4-(1H-indol-3-yl)-4-phenylpiperidine-1-carboxylate
1H NMR collected at 800.34 MHz in CDCl3

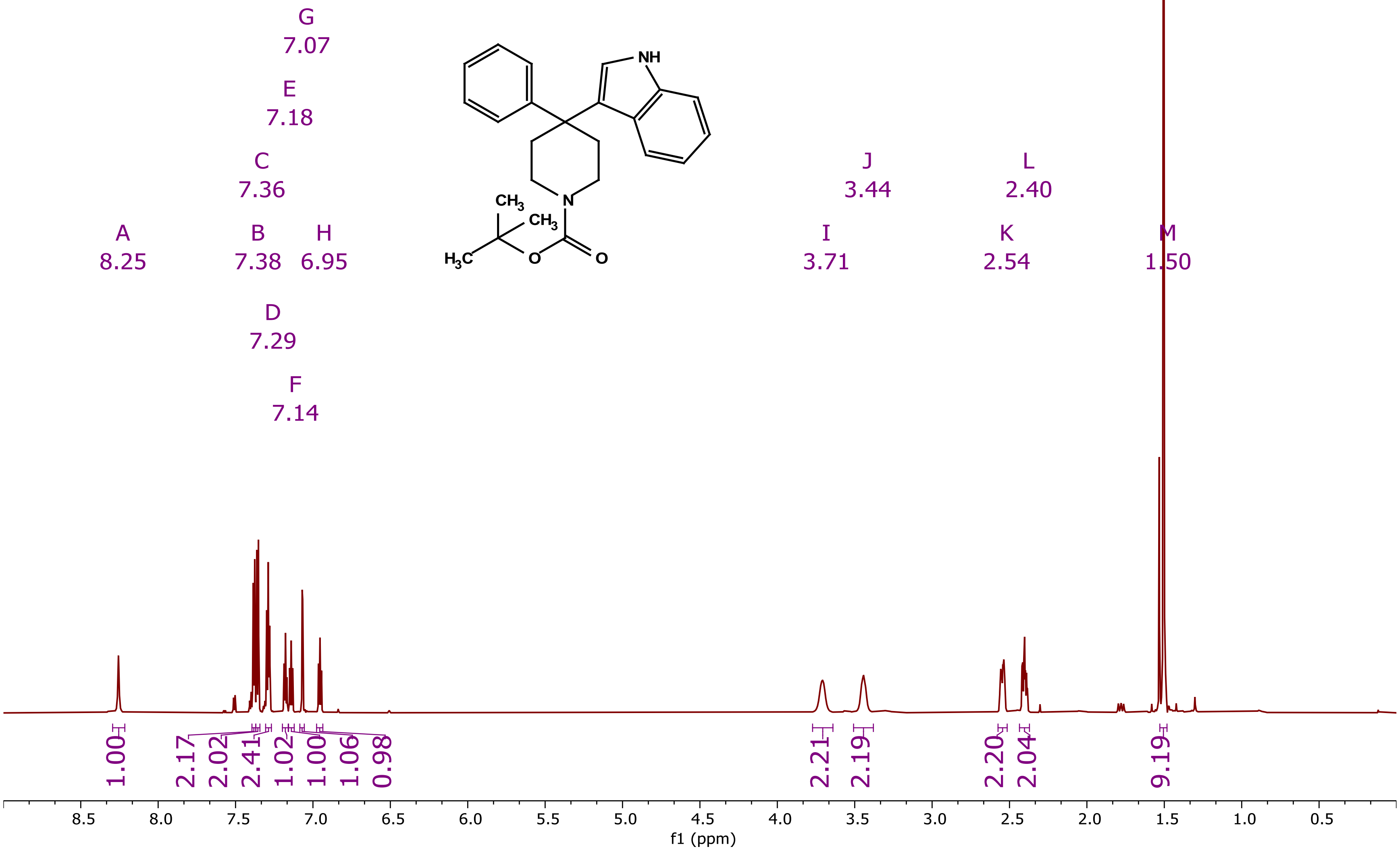

[16s] tert-butyl 4-(1H-indol-3-yl)-4-phenylpiperidine-1-carboxylate
13C NMR collected at 201.27 MHz in CDCl3

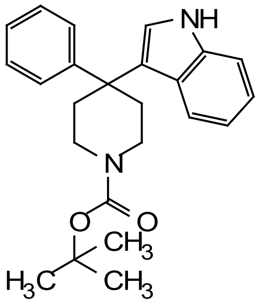

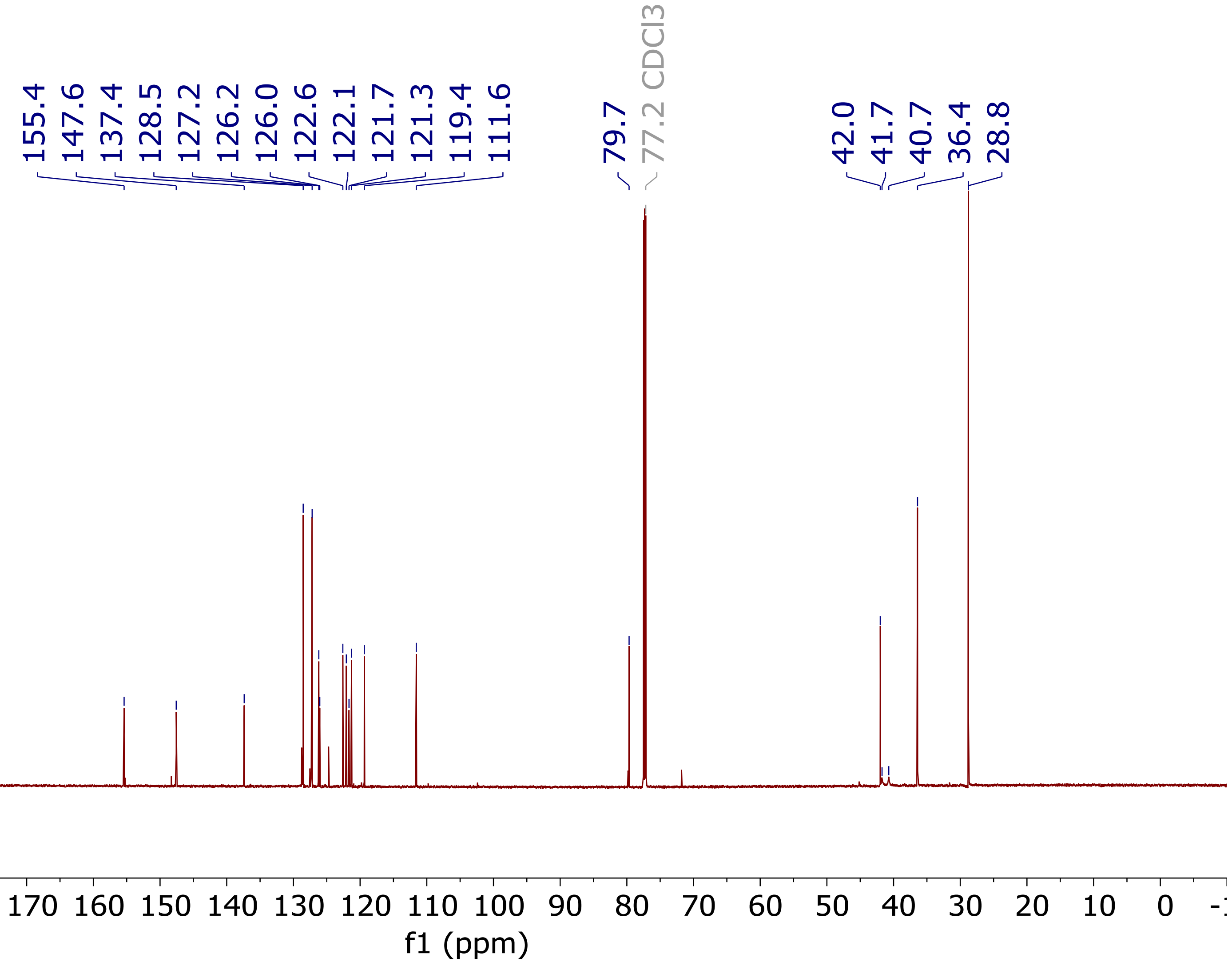

[11s] tert-butyl 4-phenyl-4-(2-phenylallyl)piperidine-1-carboxylate
1H NMR collected at 800.34 MHz in CDCl3

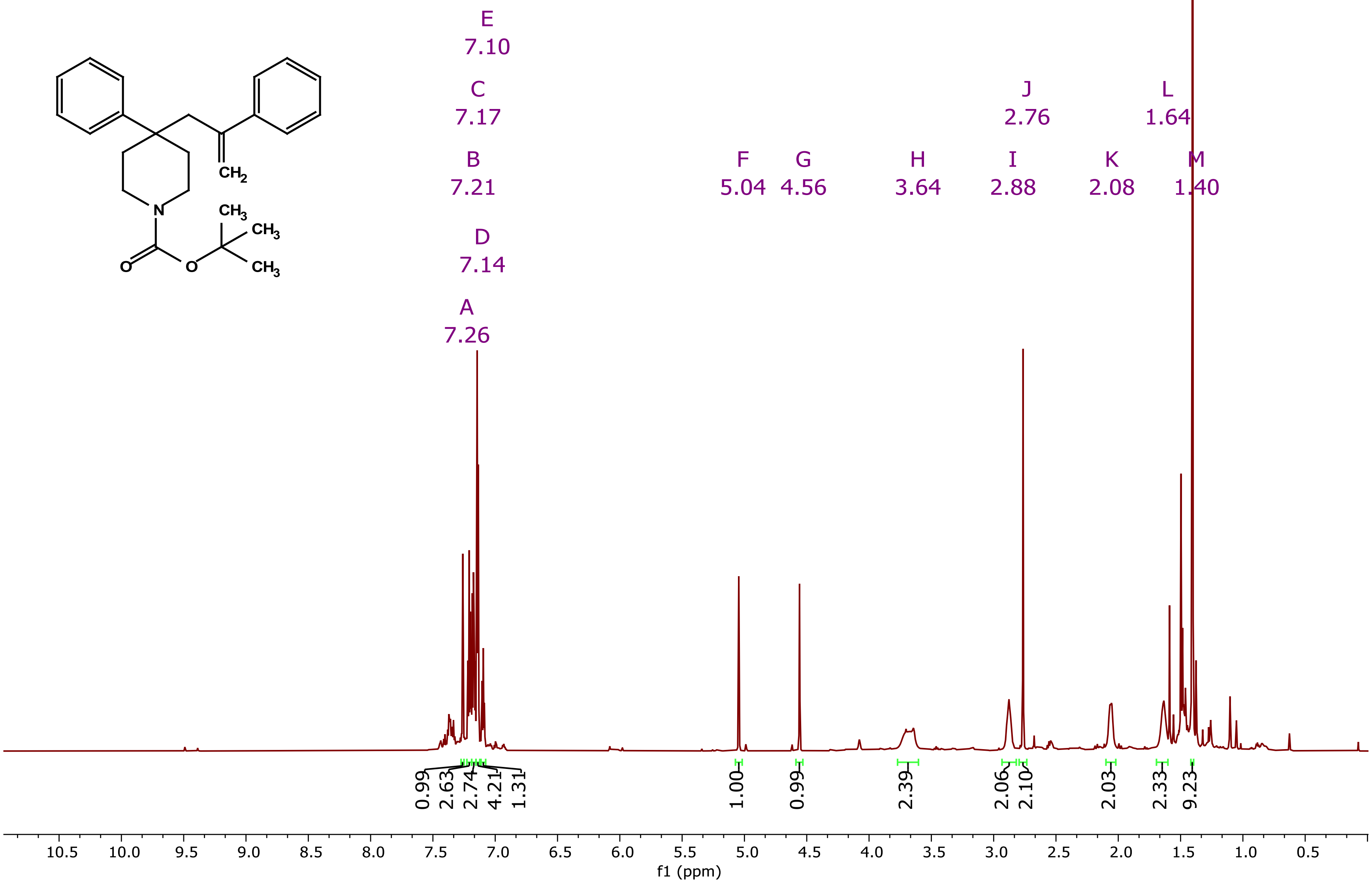

[11s] tert-butyl 4-phenyl-4-(2-phenylallyl)piperidine-1-carboxylate
13C NMR collected at 201.27 MHz in CDCl3

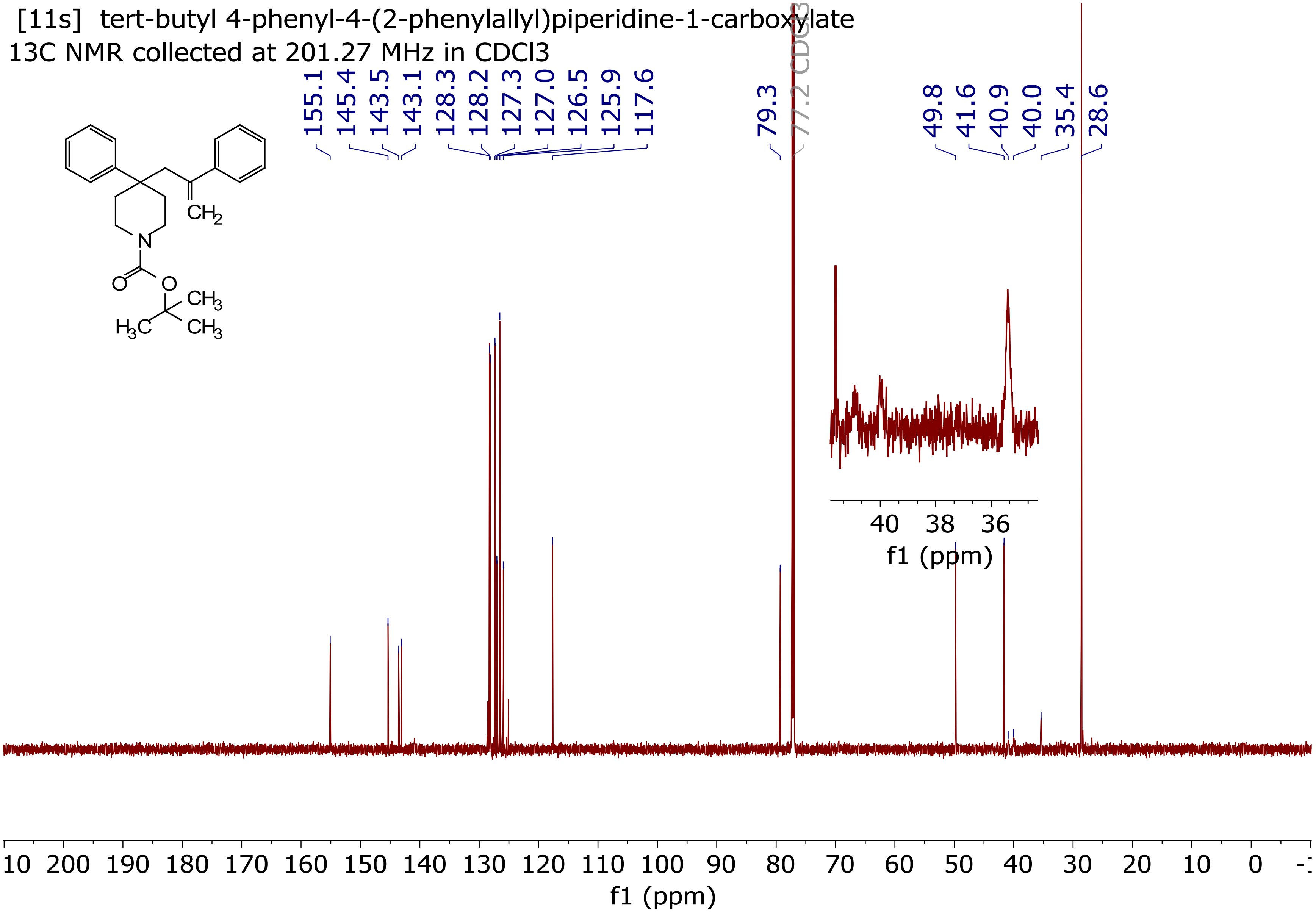

[2t] tert-butyl 4-(4-methoxyphenyl)-4-(5-methylfuran-2-yl)piperidine-1-carboxylate
1H NMR collected at 800.34 MHz in CDCl3

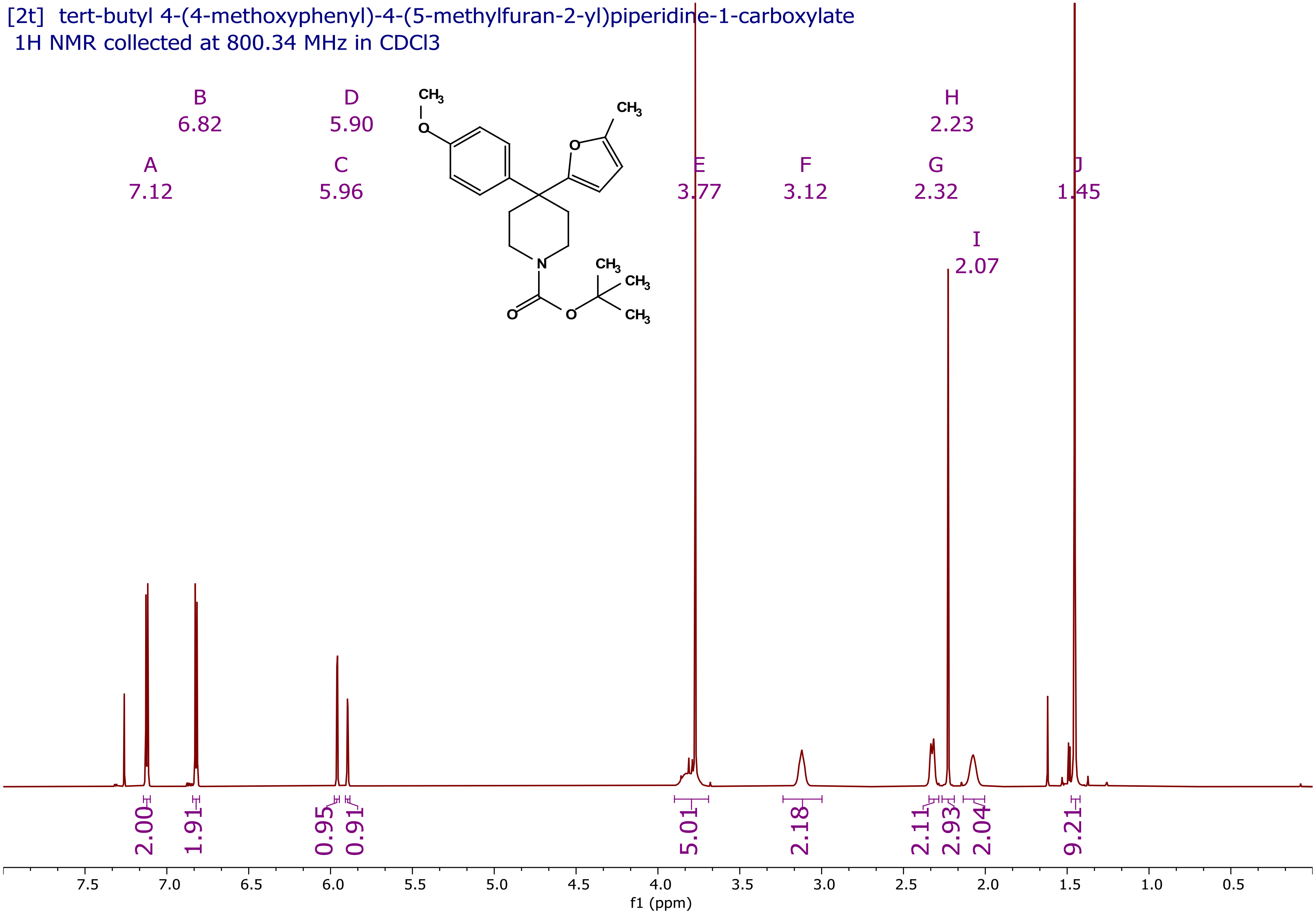

[2t] tert-butyl 4-(4-methoxyphenyl)-4-(5-methylfuran-2-yl)piperidine-1-carboxylate
13C NMR collected at 201.27 MHz in CDCl3

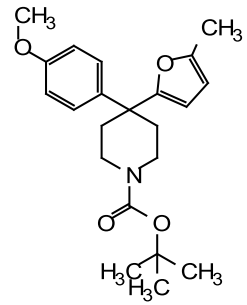

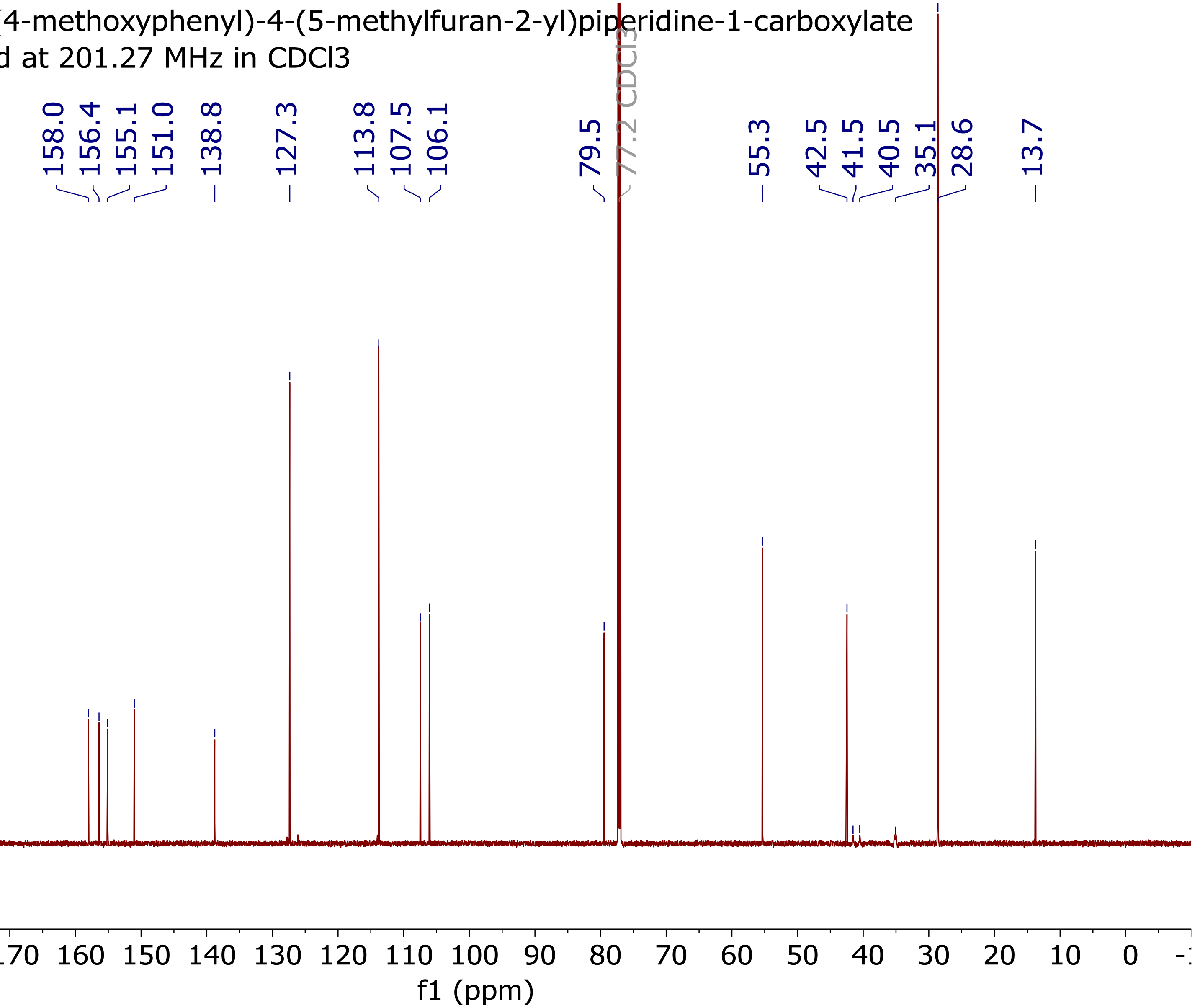

Supplement: Supplementary file 1 — jo5c00061_si_001.pdf [file jo5c00061_si_001.pdf]
