# Supplementary figures and images for: Dynamically Generated Carbenium Species via Photoisomerization of Cyclic Alkenes: Mild Friedel–Crafts Alkylation
Source: J Org Chem. 2025 Mar 5;90(10):3762–8. doi: 10.1021/acs.joc.5c00061 (PMC11915380; doi:10.1021/acs.joc.5c00061)

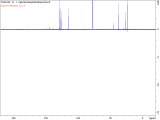

Supplement: Supplementary file 2 — jo5c00061_si_002.zip [file jo5c00061_si_002.zip › NMR/[10b-CC]/13C/pdata/1/thumb.png]

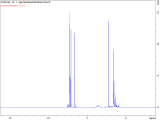

Supplement: Supplementary file 2 — jo5c00061_si_002.zip [file jo5c00061_si_002.zip › NMR/[10b-CC]/1H/pdata/1/thumb.png]

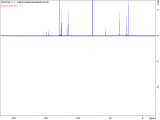

Supplement: Supplementary file 2 — jo5c00061_si_002.zip [file jo5c00061_si_002.zip › NMR/[10b-NC]/13C/pdata/1/thumb.png]

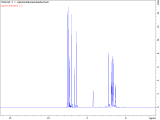

Supplement: Supplementary file 2 — jo5c00061_si_002.zip [file jo5c00061_si_002.zip › NMR/[10b-NC]/1H/pdata/1/thumb.png]

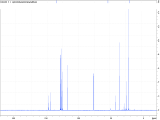

Supplement: Supplementary file 2 — jo5c00061_si_002.zip [file jo5c00061_si_002.zip › NMR/[11b]/13C/pdata/1/thumb.png]

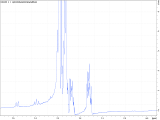

Supplement: Supplementary file 2 — jo5c00061_si_002.zip [file jo5c00061_si_002.zip › NMR/[11b]/1H/pdata/1/thumb.png]

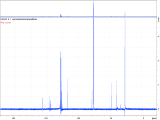

Supplement: Supplementary file 2 — jo5c00061_si_002.zip [file jo5c00061_si_002.zip › NMR/[11s]/13C/pdata/1/thumb.png]

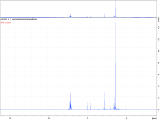

Supplement: Supplementary file 2 — jo5c00061_si_002.zip [file jo5c00061_si_002.zip › NMR/[11s]/1H/pdata/1/thumb.png]
