# Supplementary material for: Dynamically Generated Carbenium Species via Photoisomerization of Cyclic Alkenes: Mild Friedel–Crafts Alkylation
Source: J Org Chem. 2025 Mar 5;90(10):3762–8. doi: 10.1021/acs.joc.5c00061 (PMC11915380; doi:10.1021/acs.joc.5c00061)
Supplement: Supplementary file 2 — jo5c00061_si_002.zip [file jo5c00061_si_002.zip › NMR/[1k]/13C/pdata/1/email_TDS5009_11_1.pdf]

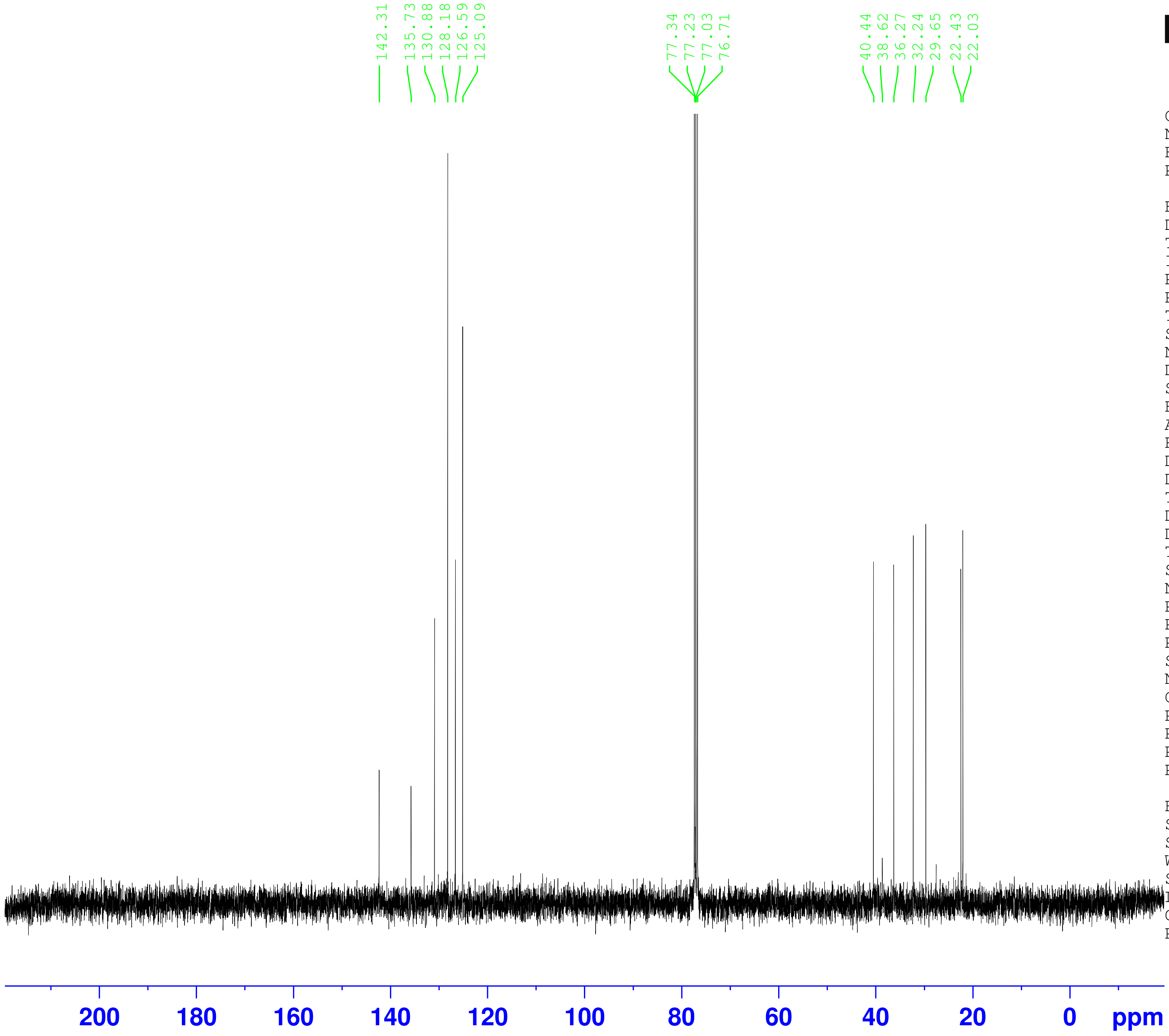


Current Data Parameters

| | |
|---|---|
| NAME | TDS5009 |
| EXPNO | 11 |
| PROCNO | 1 |

F2 - Acquisition Parameters

| | |
|---|---|
| Date_ | 20240514 |
| Time | 23.05 h |
| INSTRUM | spect |
| PROBHD | Z116098_0222 ( |
| PULPROG | zgpg30 |
| TD | 65536 |
| SOLVENT | CDCl3 |
| NS | 128 |
| DS | 4 |
| SWH | 24038.461 Hz |
| FIDRES | 0.733596 Hz |
| AQ | 1.3631488 sec |
| RG | 206.31 |
| DW | 20.800 usec |
| DE | 6.50 usec |
| TE | 298.0 K |
| D1 | 2.00000000 sec |
| D11 | 0.03000000 sec |
| TD0 | 1 |
| SFO1 | 100.6278593 MHz |
| NUC1 | 13C |
| P0 | 3.33 usec |
| P1 | 10.00 usec |
| PLW1 | 70.00000000 W |
| SFO2 | 400.1516006 MHz |
| NUC2 | 1H |
| CPDPRG[2 | waltz65 |
| PCPD2 | 90.00 usec |
| PLW2 | 13.89000034 W |
| PLW12 | 0.17148000 W |
| PLW13 | 0.08625400 W |

F2 - Processing parameters

| | |
|---|---|
| SI | 32768 |
| SF | 100.6177975 MHz |
| WDW | EM |
| SSB | 0 |
| LB | 1.00 Hz |
| GB | 0 |
| PC | 1.40 |
