# Supplementary material for: Dynamically Generated Carbenium Species via Photoisomerization of Cyclic Alkenes: Mild Friedel–Crafts Alkylation
Source: J Org Chem. 2025 Mar 5;90(10):3762–8. doi: 10.1021/acs.joc.5c00061 (PMC11915380; doi:10.1021/acs.joc.5c00061)
Supplement: Supplementary file 2 — jo5c00061_si_002.zip [file jo5c00061_si_002.zip › NMR/[1k]/1H/pdata/1/email_TDS5009_10_1.pdf]

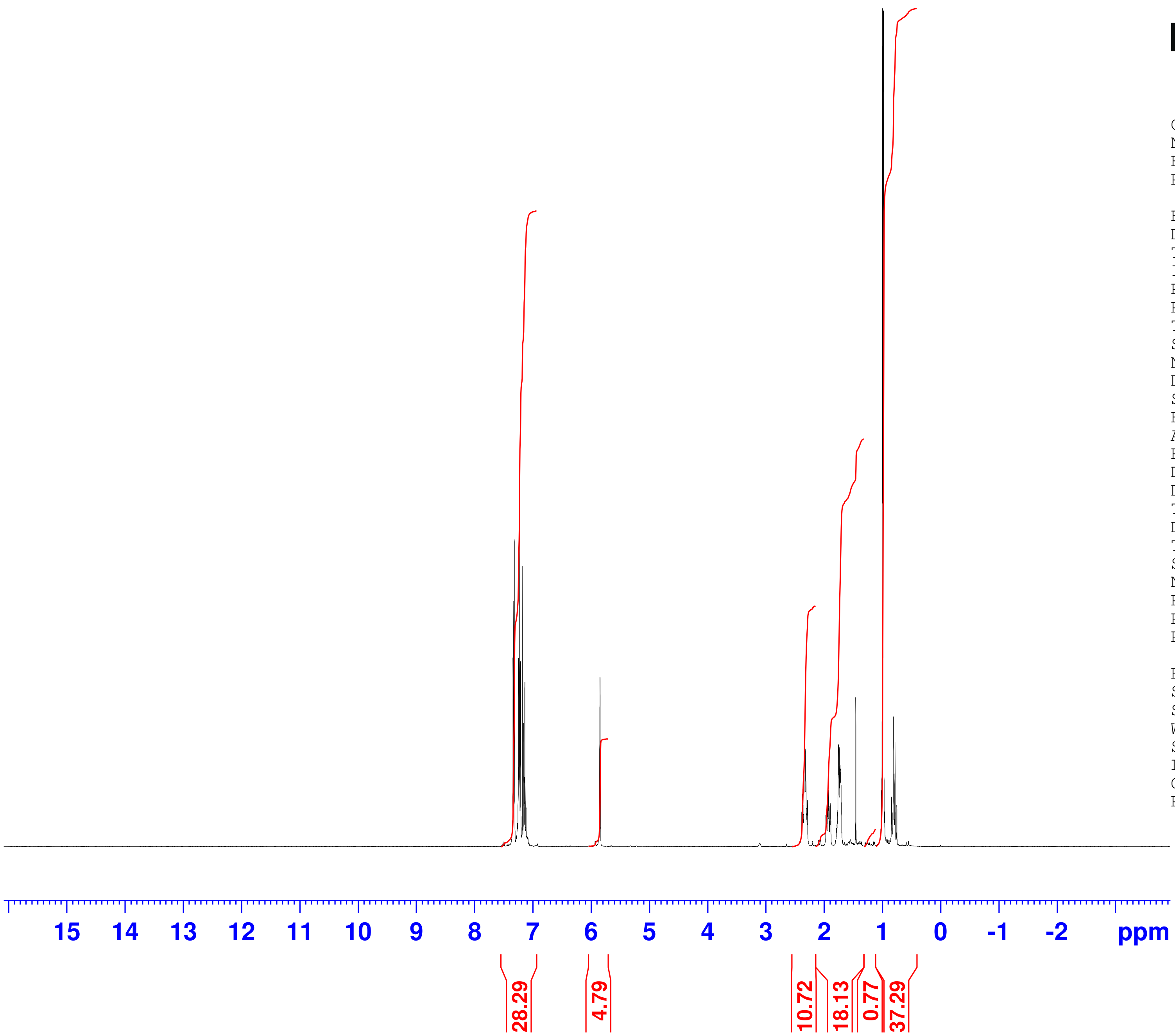


```
Current Data Parameters
NAME              TDS5009
EXPNO                  10
PROCNO                  1

F2 - Acquisition Parameters
Date_            20240514
Time                22.56 h
INSTRUM             spect
PROBHD   Z116098_0222 (
PULPROG              zg30
TD                  65536
SOLVENT             CDCl3
NS                     16
DS                      2
SWH              8012.820 Hz
FIDRES           0.244532 Hz
AQ              4.0894465 sec
RG                  91.67
DW                 62.400 usec
DE                   6.50 usec
TE                  298.0 K
D1             1.00000000 sec
TD0                     1
SFO1          400.1524709 MHz
NUC1                   1H
P0                   3.33 usec
P1                  10.00 usec
PLW1          13.89000034 W

F2 - Processing parameters
SI                  65536
SF            400.1500417 MHz
WDW                    EM
SSB      0
LB                   0.30 Hz
GB       0
PC                   1.00
```
