# Supplementary material for: Dynamically Generated Carbenium Species via Photoisomerization of Cyclic Alkenes: Mild Friedel–Crafts Alkylation
Source: J Org Chem. 2025 Mar 5;90(10):3762–8. doi: 10.1021/acs.joc.5c00061 (PMC11915380; doi:10.1021/acs.joc.5c00061)
Supplement: Supplementary file 2 — jo5c00061_si_002.zip [file jo5c00061_si_002.zip › NMR/[1l]/1H/pdata/1/email_TDS5011_10_1.pdf]

F2, tubes 13-41

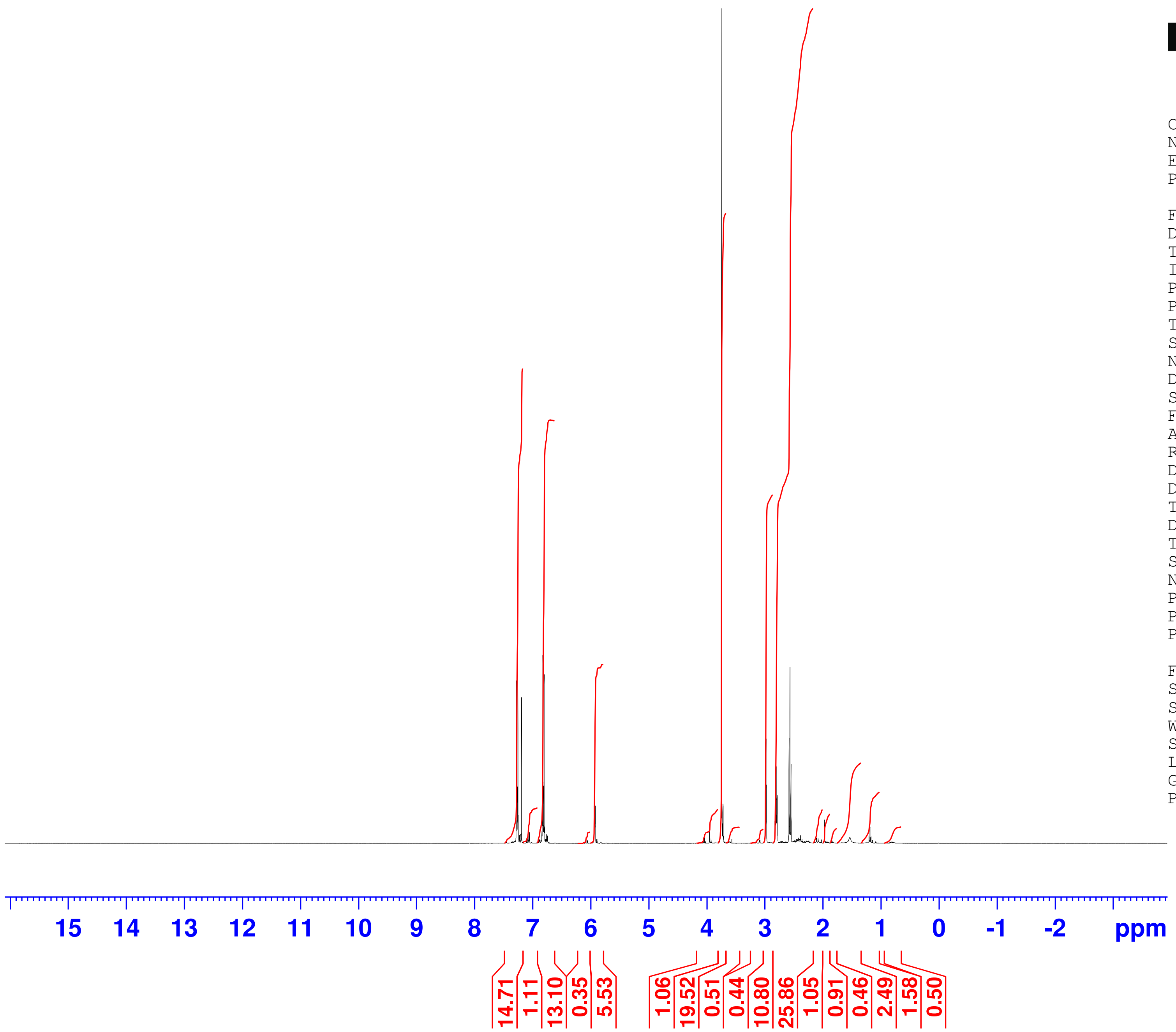

Current Data Parameters

NAME	TDS5011
EXPNO	10
PROCNO	1

F2 - Acquisition Parameters

Date_	20240516
Time	10.38 h
INSTRUM	spect
PROBHD	Z116098_0222 (
PULPROG	zg30
TD	65536
SOLVENT	CDCl3
NS	16
DS	2
SWH	8012.820 Hz
FIDRES	0.244532 Hz
AQ	4.0894465 sec
RG	49.53
DW	62.400 usec
DE	6.50 usec
TE	298.0 K
D1	1.00000000 sec
TD0	1
SFO1	400.1524709 MHz
NUC1	1H
P0	3.33 usec
P1	10.00 usec
PLW1	13.89000034 W

F2 - Processing parameters

SI	65536
SF	400.1500384 MHz
WDW	EM
SSB	0
LB	0.30 Hz
GB	0
PC	1.00
